# De Novo Hybrid Assembly of the *Tripterygium wilfordii* Mitochondrial Genome Provides the Chromosomal Mitochondrial DNA Structure and RNA Editing Events

**DOI:** 10.3390/ijms26157093

**Published:** 2025-07-23

**Authors:** Yisha Cai, Suxin Yang, Haimei Chen, Yang Ni, Jingling Li, Jinghong Zhang, Chang Liu

**Affiliations:** 1 School of Medicine, Huaqiao University, Quanzhou 362021, China; caiyisha198999@163.com (Y.C.); nievesyang11130@163.com (S.Y.); 2 Institute of Medicinal Plant Development, Chinese Academy of Medical Sciences and Peking Union Medical College, Beijing 100193, China; hmchen@implad.ac.cn (H.C.); ny_work@126.com (Y.N.); lijingling1997@163.com (J.L.)

**Keywords:** *Tripterygium wilfordii*, mitochondrial genome, RNA-editing events, MTPTs

## Abstract

*Tripterygium wilfordii* has extremely important pharmaceutical value in both traditional and modern medicine. The mitogenome of *T. wilfordii* was subjected to assembly and annotation with Nanopore long reads and Illumina short reads in this study. The mitogenome is 720,306 bp in length and is responsible for encoding 55 specific genes, including 35 protein-coding genes (PCGs), 17 transfer RNA (tRNA) genes, and 3 ribosomal RNA (rRNA) genes. Upon repetitive sequence analysis, 223 simple sequence repeats (SSRs), 24 long tandem repeats (LTRs), and 47 dispersed repetitive sequences (DRSs) were identified. The 24 common PCGs were used for phylogenetic analysis, which revealed that *T. wilfordii* is more closely related to *Euonymus alatus*. Moreover, mitochondrial plastid DNA (MTPT) analysis revealed eight MTPTs in the mitochondrial genome. Furthermore, 600 RNA-editing sites were detected in the protein-coding genes according to RNA-seq results. Among these genes, the ccmB gene contained the greatest number of sites, followed by the nad4 gene. This is the first study to report the *T. wilfordii* mitogenome and illustrate its linear structure. The findings of this study will help elucidate the evolution of the *T. wilfordii* mitogenome and facilitate its potential application in genetic breeding.

## 1. Introduction

*Tripterygium wilfordii Hook. f.* is a perennial twining shrub belonging to the Celastraceae family and is distributed widely across East Asia [1]. The species is recognized as a Chinese medicinal herb that is used for managing various autoimmune conditions, such as rheumatoid arthritis and systemic lupus erythematosus [2,3]. *T. wilfordii* has numerous secondary metabolites, including triptolide and celastrol, which possess anti-inflammatory, immuno-suppressive, and antitumor activities and, therefore, have great development potential in the field of modern medicine [4,5]. Moreover, triptolide and celastrol are important in managing central nervous system disorders, such as Parkinson’s disease and Alzheimer’s disease [6,7]. Celastrol can also be used for the treatment of metabolic disturbances, such as high-fat-diet-induced obesity and type 2 diabetes [8,9]. High-throughput sequencing substantially contributes to the study of plant transcriptomes and genome architecture and is also useful when exploring plant genetic variations and secondary metabolites at the molecular level. Many transcriptome and genome studies conducted to date have revealed the key enzyme-encoding genes in the mevalonate (MVA) and methylerythritol phosphate (MEP) pathways, which regulate the biosynthesis of triptolide and celastrol through transcriptome data, as well as various transcription factors or miRNAs that regulate the biosynthesis of secondary metabolites in *T. wilfordii,* such as the negative regulation of triptolide biosynthesis by MYC2 [10]. In addition, the high-quality reference *T. wilfordii* genome has been illustrated, highlighting the regulatory role of CYP450 in the biosynthesis of triptolide [11]. The elucidation of the complete *T. wilfordii* chloroplast genome will be useful for molecular-assisted breeding in future studies [1]. However, the *T. wilfordii* mitochondrial genome sequence remains unreported to date, greatly limiting subsequent studies on this species.

In plants, the mitochondrial genome, which has many kinds of rearrangements, can be quite complex, although it may be used for various beneficial applications. First, as the mitochondrial genome has an extremely high rate of evolution, it has been extensively used as a molecular marker in modern molecular biology research, such as for reconstructing the phylogenetic relationships of species. Second, as cellular organelles, mitochondria are responsible for generating ATP and various metabolites, and the mitochondrial release of the metabolites of the tricarboxylic acid (TCA) cycle controls cell activity and fate [12]. Third, the plant mitogenome is huge and has a complicated structure and genetic diversity, although the stability of a plant mitogenome is protected through certain DNA repair mechanisms, particularly homologous recombination (HR) and base excision repair (BER) [13]. Finally, RNA editing, such as the conversion of cytidines (C) to uridines (U), which is important for the normal functioning of protein-encoding genes, is frequently detected in plant mitochondria [14]. In addition, *T. wilfordii* has long been used for asexual reproduction, resulting in many problems, such as confusion in terms of variety, serious degradation, and a low content of active ingredients. Consequently, exploring the *T. wilfordii* mitochondrial genome is necessary. This work, therefore, involved the sequencing and assembly of the complete *T. wilfordii* mitochondrial genome; analysis of the potential *T. wilfordii* mtDNA structure; and comparative analyses of repetitive sequences, homologous recombination, phylogenetic relationships, and RNA editing for this species. The findings of this study are expected to contribute to the further exploration of scientific issues encountered in relevant research at the molecular level and lay a certain theoretical basis for the genetic engineering and breeding of medicinal plants.

## 2. Results

### 2.1. Graph-Based Mitogenome and General Genomic Features

**Figure 1:**
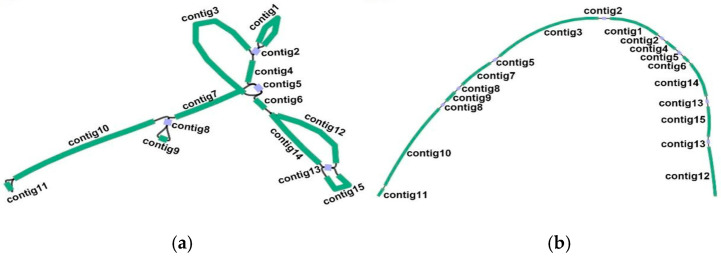
Unitig graph showing the *T. wilfordii* mitogenome. (**a**) Green and purple contigs are the mitochondrial chromosomes (MCs), whereas purple contigs are double-bifurcation structures (DBSs). (**b**) The graph after resolving the DBSs using long reads.

**Figure A1:**
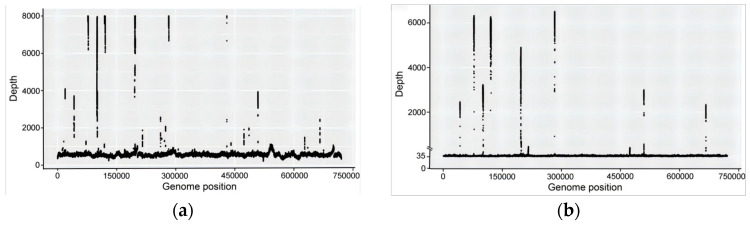
The coverage depth of the mitochondrial genome of *T. wilfordii.* The *x*-axis represents the genome position. The *y*-axis is the depth of those positions. (**a**) The coverage depth for Illumina short reads. (**b**) The coverage depth for Nanopore long read.

**Figure A2:**
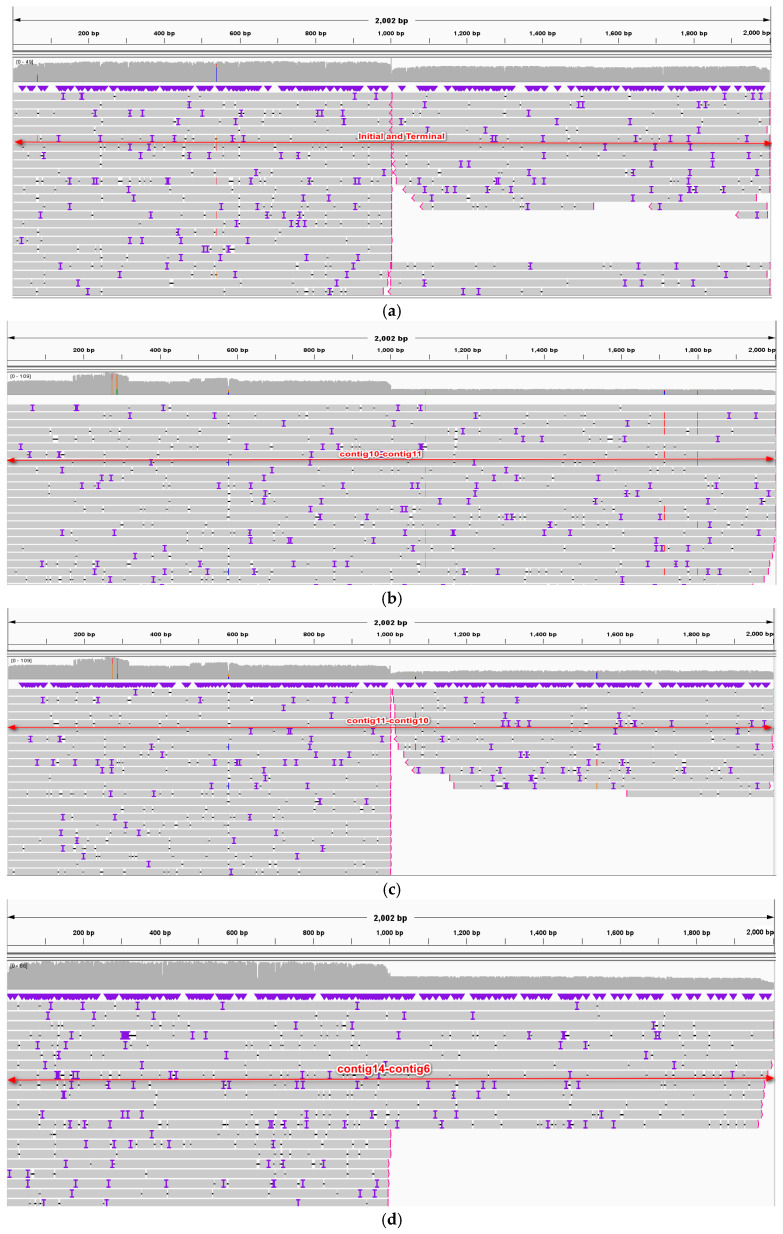

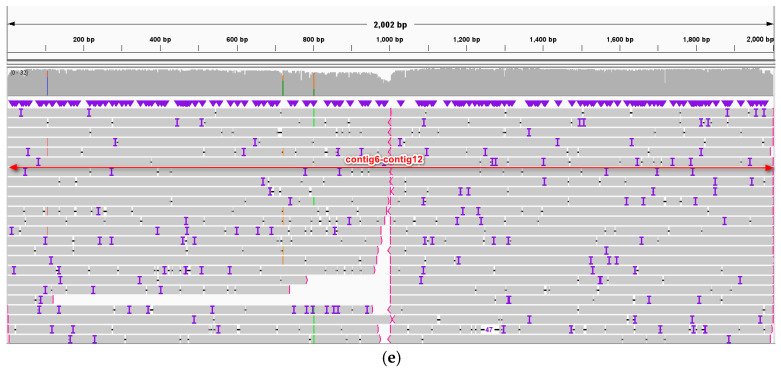
The validation of structure of the mitochondrial genome of *T. wilfordii*. (**a**) The junction between initial and terminal (2000 bp), The initial is the start of contig11 and terminal is the end of contig12 (**b**) the junction between contig10 and start of contig11 (2000 bp), (**c**) the junction between contig11 and end of contig10 (2000 bp), (**d**) the junction between contig14 and contig6 (2000 bp), and (**e**) the junction between contig6 and contig12 (2000 bp).

The hybrid assembly method was used to construct the *T. wilfordii* mitogenome by adopting long/short reads. First, 10 Gb of Illumina short reads and 7 Gb of Nanopore long reads of the total DNA were sequenced (Table A1). GetOrganelle software (v1.7.0) was then used for extracting and elongating the mitochondrial genome short reads, and SPAdes software (v3.8.2) in Unicycle was usyed for assembly into a unitig graph using the whole-genome sequencing results (Figure 1a). Moreover, Illumina short reads together with Nanopore long reads were used for calculating the sequencing depth at each genomic locus, thereby validating the accuracy of the assembly. The sequencing depth remained consistent among the genomic locations, without detectable gaps (Figure A1a,b). The *T. wilfordii* mitochondrial genome was revealed to have 15 contigs, including 4 double-bifurcation structures (Table 1 and Figure 1a). The longest contig was contig 3, with a length of 186,151,728 bp, and the shortest contig was contig 8, a double-bifurcation structure with a length of 1728 bp. Second, the Nanopore long reads resolved into double-bifurcation structures, with each contig merged using Bandage software (v0.8.1) (Figure 1b). Finally, one chromosome was obtained independently. The Nanopore long reads were then used to validate the *T. wilfordii* mitochondrial genome structure (Figure A2a–e).

**Figure 2:**
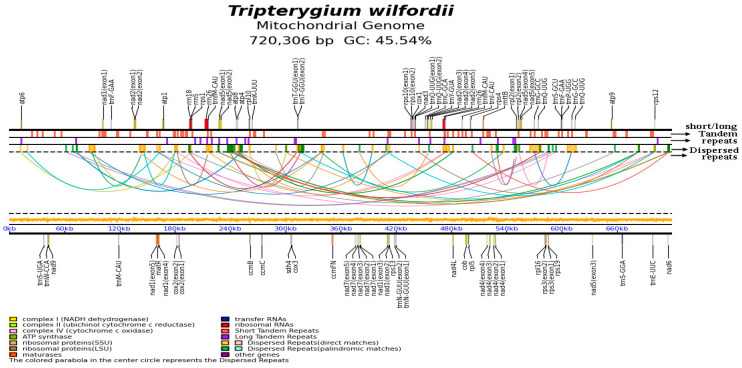
A sketch map showing the *T. wilfordii* mitogenome. PMGmap (http://www.1kmpg.cn/pmgmap, accessed on 14 July 2025) was used for graph creation. This graph represents (1) the genes on both positive and negative strands; (2) the short tandem repeat distribution on the chromosome; (3) the long tandem repeat distribution on the chromosome; (4) the relationship of the dispersed repeat sequences; (5) the dispersed repeat sequence distribution on the chromosome, with yellow and green indicating the direct and inverted dispersed repeat sequences, respectively; (6) the scale coordinate axis; and (7) the GC content of the chromosome.

The *T. wilfordii* mitochondrial genome was revealed to contain one chromosome 720,306 bp in length. The GC content was 45.54% (Figure 2), and it was revealed to be closely related to the *Euonymus alatus* mitochondrial genome in terms of size (genome size: 1,045,106 bp; NCBI accession number: NC_053921.1). In addition, the *T. wilfordii* mitochondrial genome was annotated as having a total of 61 genes (55 unique genes), including 35 PCGs, 22 tRNA genes (17 unique genes), and 4 rRNA genes (3 unique genes). The PCGs were classified into 10 functional groups (Table 2). The annotation of the core genes in the *T. wilfordii* mitochondrial genome was completed, revealing three variable gene types, including seven small subunit ribosome genes (*rps1*, *rps10*, *rps12*, *rps13*, *rps19*, *rps3*, and *rps4*), four large subunit ribosome genes (*rpl10*, *rpl16*, *rpl2*, and *rpl5*), and one subunit succinate dehydrogenase gene (*sdh4*).

### 2.2. Tandem Repeat Analysis

SSRs, also referred to as short tandem repeats or microsatellites, generally represent repetitive DNA sequences containing 1–6 nucleotides [15,16]. SSRs have been extensively used as genetic markers in forensics, disease diagnosis, and population genetics [17]. This work used the MISA online platform [18] for predicting the SSRs present in the *T. wilfordii* mitogenome. A total of 223 SSRs were identified on the chromosome of the mitogenome, including 66 monomers, 49 dimers, 29 trimers, 70 tetramers, 7 pentamers, and 2 hexamers (Table A2). These identified SSRs might be crucial molecular markers for subsequent analyses. LTRs, in which the repeat units contain ≥7 nucleotides, are crucial for numerous biological events, such as translation, transcription, promoter activity regulation, genome evolution, and chromosome stability [19,20,21]. The LTRs in the *T. wilfordii* mitochondrial genome were predicted using the Tandem Repeats Finder program [22] in this study. A total of 24 LTRs were predicted from the results (Table S1). LTR1 presented the greatest number of repeat units at 48 bp, whereas LTR16 was only 13 bp long.

### 2.3. Recombinations Mediated by Repetitive Sequences

Dispersed repetitive sequences (DRSs), or scattered repetitive sequences, are repeated at multiple positions across a genome and are used for determining biological evolution, individual identification, and disease diagnosis [23]. Furthermore, DRSs are crucial for determining mitogenome structural variations [24]. In this study, ROUS Finder 2.0.py [25] was used for predicting DRSs. A total of 47 DRSs were detected in the *T. wilfordii* mitochondrial genome, including 18 palindromic repetitive sequences and 29 direct repetitive sequences (Table 3). The DBSs were then compared to these DRSs, revealing four DBS sequences similar to DRS02–DRS05 (refer to Table 3, Figure S1, and Table S2). First, every repetitive sequence pair with 500 bp flanking sequences was isolated and used to create reference sequences representing the four possible conformations to be recombined. Second, the 47 DRSs were assessed for their recombination frequency with Nanopore long reads, revealing that no recombined conformation was supported by the Nanopore long reads (Table S2). Using the conformation as the baseline, it was determined that *T. wilfordii* has no four repetitive sequence-mediated recombinations.

### 2.4. Analysis of the Homologous Sequences Between Plastids and Mitochondria

**Figure 3:**
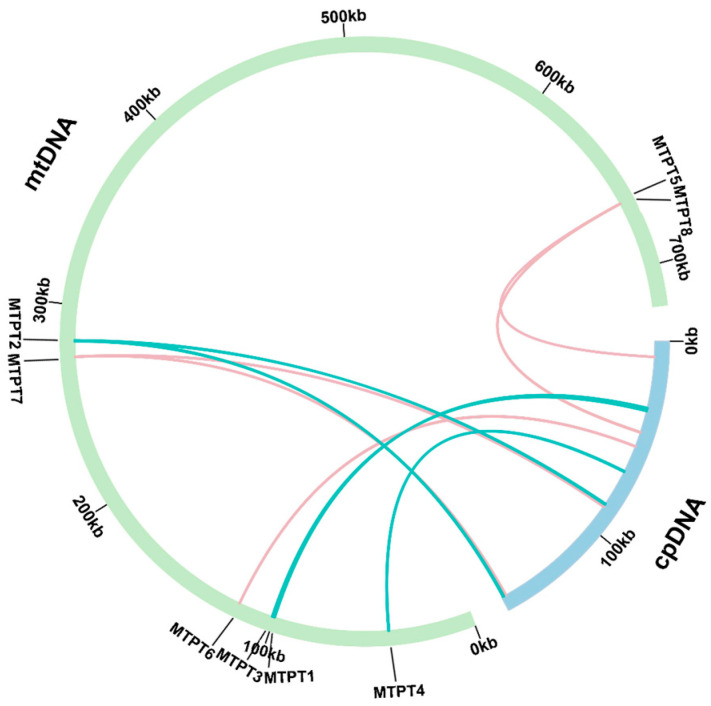
Mitochondrial plastid DNA MTPTs in the *T. wilfordii* mitogenome. The blue and green arcs represent the chloroplast and mitochondrial genomes, respectively. The green and pink arcs within the circle represent homologous regions in the chloroplast and mitochondrial genomes, respectively.

Mitochondrial plastid DNAs (MTPTs) refer to plastid-derived DNA fragments in mitochondrial genomes [26]. These sequences originate from rare inter-organellar DNA transfer events, where fragments of the chloroplast genome are inadvertently integrated into the mitochondrial genome. Such transfers can cause misattribution of organelle DNA in taxonomic studies and complicate genome assembly/annotation, owing to chimeric origins. Therefore, the rigorous identification of MTPTs is essential for resolving these complexities. MTPTs have been reported in numerous species, such as *Coffea arabica* [27], *Salvia miltiorrhiza* [28], and *Caragana spinosa* [29]. In this study, the dataset adopted in the mitochondrial genome (OR538545.1/NC_082972.1) assembly was used for assembling the *T. wilfordii* chloroplast genome (OR538544.1), which is 156,692 bp long, with a GC content of 37.47%. The chloroplast genome included 131 genes (111 unique genes), comprising 87 PCGs (78 unique genes), 36 tRNA genes (29 unique genes), and 8 rRNA genes (4 unique genes) (Table S3 and Figure 3).

**Figure 4:**
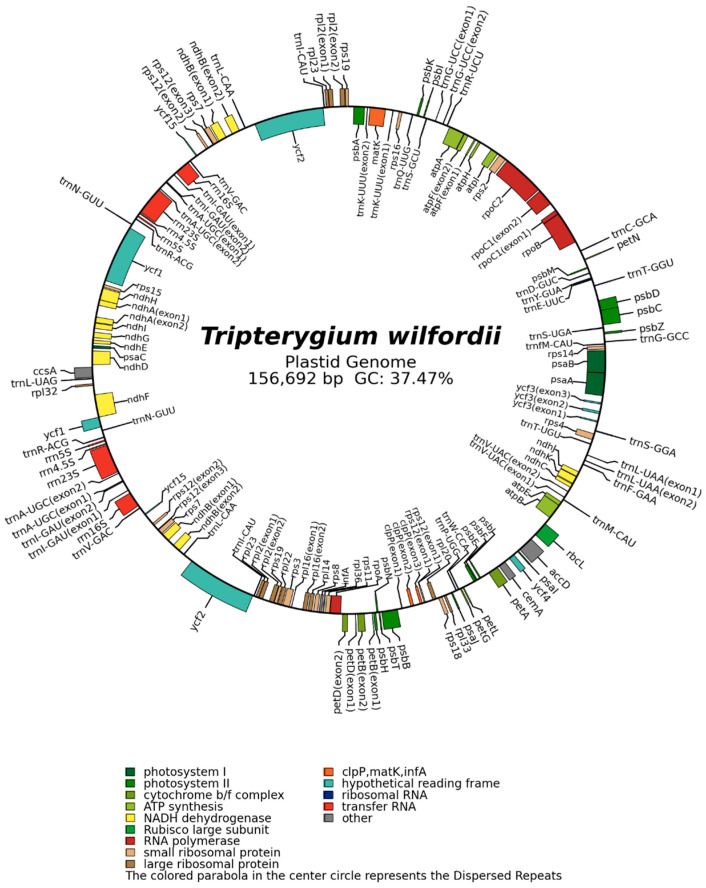
A schematic representation of the *T. wilfordii* chloroplast genome. The graph was drawn using PMGmap (http://www.1kmpg.cn/pmgmap, accessed on 14 July 2025). For the graph, the following are represented from inside-out: (1) the distribution of the GC content on the chromosome and (2) the genes located on the negative strand and the positive strand.

A total of eight MTPTs were identified in *T. wilfordii* by comparing the complete mitochondrial DNA with the chloroplast DNA from *T. wilfordii* using BLAST with default parameters [28]. These eight MTPTs were 3297 bp long, accounting for 0.46% of the entire mitochondrial genome. Among these, MTPT1 had the greatest length (1048 bp), whereas MTPT8 had the smallest length (30 bp). MTPT2 and MTPT7 were located in the inverted repeat (IR) region of the chloroplast genome (Figure S2). In addition, the locations of the eight MTPTs within the chloroplast and mitochondrial genomes are provided in Table S3 and Figure 3. In order to investigate the functions of these eight MTPTs, the DNA fragments were subjected to annotation, revealing that the MTPTs contained complete and functional ptDNA-encoded genes (trnW–CCA, trnP–UGG, trnS–GGA, and trnM–CAU) and partial-protein-encoding genes (psbD, psbC, and rpl2) (Figure 4 and Table S3). In order to further confirm that total MTPTs were present, Nanopore long reads were aligned to the reference sequences containing MTPTs as well as to the MTPT sequence and the corresponding 1000 bp upstream/downstream sequences. The mapping results revealed that the Nanopore long reads supported all eight MTPTs (Figure 4).

### 2.5. Phylogenetic Analysis

**Figure 5:**
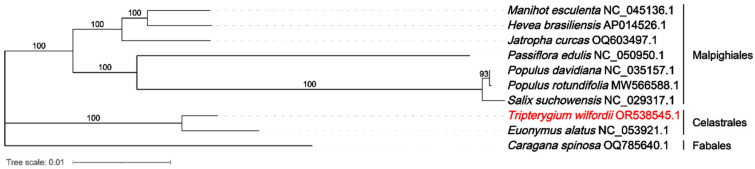
The NCBI nucleotide database has 136 mitochondrial genomes for Malpighiales, 2 for Celastrales, and 251 for Fabales. Phylogenetic relationships between *T. wilfordii* and nine additional species were inferred based on the sequences of 24 PCGs from their mitochondrial genomes. Phylogenetic trees were constructed using the maximum likelihood (ML) method. The numbers on the branches represent bootstrap values, indicating the support level for each node. GenBank accession numbers for the mitochondrial genomes are provided following the Latin names of the species, and the corresponding plant families are listed on the right side of the tree.

The NCBI nucleotide database has 136 mitochondrial genomes for Malpighiales, 2 for Celastrales, and 251 for Fabales. In order to analyze the phylogenetic relationships among the nine Celastrales and Malpighiales species, phylogenetic trees were created using the CDSs among the 24 common PCGs in the mitochondrial genomes (atp1, atp4, atp6, atp8, atp9, ccmB, ccmC, cox1, cox2, cox3, cob, matR, mttB, nad1, nad2, nad3, nad4, nad5, nad6, nad7,nad9, rps3, rps4, and rps12). It was found that *T. wilfordii* and *Euonymus alatus* were clustered under 100 bootstrap supports (Figure 5). These results showed that *T. wilfordii* and *Euonymus alatus* are more closely related. Additionally, each node showed a bootstrap support of >90, suggesting that these nine mitochondrial genomes of Celastrales and Malpighiales species have strongly reliable phylogenetic relationships.

### 2.6. RNA-Editing Event Analysis

**Figure 6:**
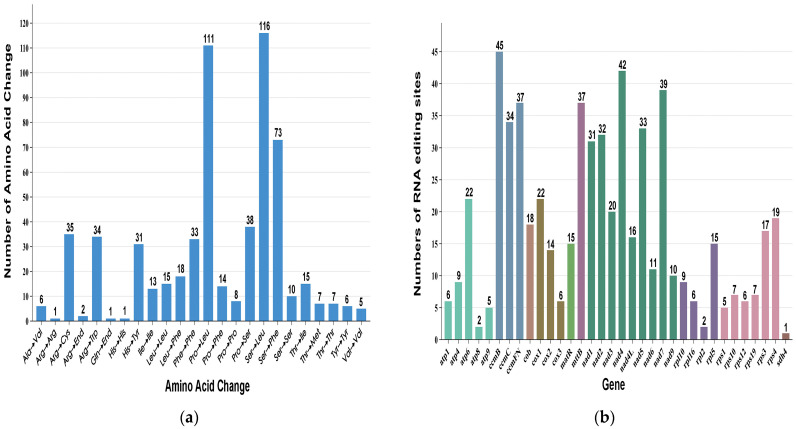
Numbers of the RNA-editing sites within the *T. wilfordii* mitogenome. (**a**) The *x*-axis and *y*-axis represent the amino acids altered under RNA editing and the number of changed residues for each amino acid, respectively. (**b**) The *x*-axis and *y*-axis represent the number of genes affected by RNA editing and the number of RNA edits, respectively. Different colors indicate various families of genes.

RNA editing is an important supplement to the central dogma, and it differs from the corresponding DNA templates and exerts critical effects on various processes, including chloroplast and mitochondrial biogenesis, hormone and stress responses, and seed growth [29,30]. C-to-U conversion within plant organelles is the major RNA-editing pattern [30]. In this study, a total of RNA editing sites were identified in the *T. wilfordii* mitogenome following RNA-seq analysis (Table 4 and Figure S3). The false positives in the analysis of mitochondrial genomic polymorphic sites were eliminated by identifying the SNPs using the DNA-seq analysis of the identical samples of RNA-seq data. Three overlaps, cox2–698, cox2–721, and nad6–26, were obtained through the comparison of the predicted RNA-editing sites and SNP sites (Table S4 and Figure S3). Consequently, 600 RNA-editing sites were obtained from the *T. wilfordii* mitogenome (Table S4), among which 99 (16.50%) and 501 (83.50%) sites were altered, resulting in 99 synonymous and 501 non-synonymous codons, respectively. Variations in the number of non-synonymous codons for the Ser, Pro, and Arg amino acids were observed, among which 116 (19.33%) RNA-editing sites had Ser altered to Leu, 112 (8.67%) had Pro altered to Leu, 72 (12.00%) had Ser altered to Phe, 38 (6.33%) had Pro altered to Ser, 35 (5.83%) had Arg altered to Cys, and 34 (5.67%) had Arg altered to Trp (Table S4 and Figure 6a). The RNA-editing events within *T. wilfordii* mitochondria occurred mostly at the first and second codon base positions, accounting for 30.83% (185) and 52.67% (316) of the overall RNA-editing sites, respectively. Additionally, the RNA-editing events occurred in the most unique PCGs within the *T. wilfordii* mitochondrial genome. Among these genes, ccmB represented the highest number of RNA-editing sites (*n* = 45), followed by nad4 with 42 sites (Figure 6b). Notably, RNA-editing events resulted in the generation of both start and stop codons. Specifically, codon changes at positions atp6-718, atp9-223, and rps19-7 led to the conversion of CAA to TAA and CGA to TGA, effectively producing stop codons.

## 3. Discussion

### 3.1. Overview of the T. wilfordii Mitochondrial Genome

*T. wilfordii* is a traditional Chinese medicine that exhibits excellent medicinal effects in terms of curing nephrotic syndrome, rheumatoid arthritis, and systemic lupus erythematosus; in addition, this species has been extensively adopted in China as a folk medicine [31]. Acquiring the genomic data of this species constitutes a crucial step toward comprehending its active constituents’ physiological traits and biosynthetic processes. In this study, using hybrid assembly and annotation methods involving Illumina short reads and Oxford Nanopore long reads, the complete *T. wilfordii* mitogenome was identified. Subsequently, the gene contents and numbers, SSRs, TRSs, DRSs, homologous sequences, and RNA-editing events of the genome were determined. Finally, the phylogenetic relationships of the genome with eight Celastrales and Malpighiales species were explored according to the conserved PCGs in the mitochondrial genomes. The mitogenome of *T. wilfordii* will serve as a reference for investigating the genomes of other plants in the Celastraceae family and for Tripterygium mitogenome evolution and diversity research.

### 3.2. Architecture of One Molecule of the T. wilfordii Mitogenome

Plant mitogenome assembly is generally achieved and presented in circular maps according to the extensively accepted concept among living scientists that plant mitochondrial DNA exists mainly as molecules of circular genomes [32]. However, the physical structure of the real mitochondrial genome may involve different circles, complex branching structures, and linear molecules [33]. The results of this study revealed that the *T. wilfordii* mitochondrial genome is composed of one molecule that is 720,306 bp in length (Figure 1a,b). The molecular configuration, as depicted in Figure 1a, is characterized by the presence of two distinct bubble structures, one large and one small. In contrast, Figure 1b illustrates a linear structure. However, the linear structure is supported by the nanopore long reads (Figure A1 and Figure A2). The mitochondrial genomes of plants usually exhibit diverse alternative conformations because of the presence of many repetitive sequences [34,35]. According to this study, the *T. wilfordii* mitochondrial genome does not undergo recombination mediated by repetitive sequences. By extracting repetitive elements along with their 500 bp flanking regions as reference sequences and mapping them onto Nanopore long reads, it was confirmed that no recombination conformations associated with repetitive sequences are present in the *T. wilfordii* mitochondrial genome.

### 3.3. Research Trends for the T. wilfordii Mitogenome

The mitogenomes of plants differ significantly in length compared to chloroplast genomes due to frequent exchange with nuclear and chloroplast DNA [36,37]. In this study, the MTPTs of *T. wilfordii* were identified. These MTPTs were then subjected to functional annotation. MTPTs in the *T. wilfordii* mitochondrial genome included four complete and functional ptDNA-encoded genes (trnW–CCA, trnP–UGG, trnS–GGA, and trnM–CAU) and three partial-protein-coding genes (psbD, psbC, and rpl2). Furthermore, MTPT2 and MTPT7 were localized to the inverted repeat (IR) region of the chloroplast genome. Nonetheless, MTPT2, the partial rpl2, could be detected within the chloroplast and mitochondrial genomes. Thus, it was impossible to precisely determine whether MTPT3 originated from the *T. wilfordii* chloroplast or mitochondrial genome.

RNA editing, an extensive phenomenon observed in the organelles of higher plants, is used in several processes with different mechanisms that change the nucleotide sequences of RNA molecules so that the RNAs are different from the corresponding gene sequences [38,39]. In addition, RNA-editing events frequently occur at the first and second codon positions within the plant mitochondrial genome. In the *T. wilfordii* mitochondrial genome, this study identified 600 RNA-editing sites with alterations in one base, producing synonymous and non-synonymous amino acid codons, which in turn affected the translation of proteins. Ser, Pro, and Arg were the most frequently altered amino acids resulting from non-synonymous codon changes. Among the identified RNA-editing sites, only 99 (16.50%) occurred at the third codon position, while 501 (83.50%) were located at the first or second positions, indicating a predominant localization of editing events at the first and second codon positions. Furthermore, most PCGs in *T. wilfordii* contained RNA-editing sites, with the exception of rps13. These RNA-editing events can modify and refine genetic information, enhance the diversity of gene products, and thereby contribute to plant evolutionary adaptation.

## 4. Materials and Methods

### 4.1. Plant Materials, DNA and RNA Isolation, and Sequencing

Fresh *T. wilfordii* leaf samples were obtained from the nursery of the medical school of Huaqiao University (Quanzhou, China). Thereafter, a DNA extraction kit and an RNAprep Pure Plant Plus Kit (Tiangen Biotech, Beijing, China) were used to extract the total DNA and RNA, respectively, which were preserved at −80 °C until use. Specifically, the extracted total DNA was used for Illumina sequencing and Oxford Nanopore sequencing (Table A1). For Illumina sequencing, 1 µg of the genomic DNA was used to construct a DNA library with the NEBNext library building kit, followed by Illumina 2500 platform sequencing (Illumina, San Diego, CA, USA). For Oxford Nanopore sequencing, a 10 kb DNA library was established prior to sequencing using a PromethION sequencer (Oxford Nanopore Technologies plc, Oxford, UK). The TruSeq Stranded mRNA Library Prep kit (Illumina) was used to construct a strand-specific RNA-seq library. Illumina sequencing was performed using PE150 sequencing for the quantified library.

### 4.2. Genome Assembly, Annotation, and Validation

GetOrganelle software (3.8.2)was used to complete the organelle genome assembly [40] In this study, the parameters “–R 15–k 21,45,65,85,105–F embplant_pt” were adopted for assembling the chloroplast genome. However, mitochondria-derived nuclear and plastid DNA can result in false positives during mitogenome polishing [41]. Thus, mitochondrial genome assembly was completed using the single hybrid assembly strategy. First, the parameters “–R 20 –k 21,45,65,85,105 –P 1000,000 –F embplant_mt” were used to obtain mitochondrial short reads using GetOrganelle (3.8.2) [40]. Second, long and short reads were assembled through de novo assembly into a unitig graph using SPAdes [42], miniasm, and Racon packages [43] included in Unicycler software (v0.5.0). Finally, using Unicycler software, the Nanopore long reads were adopted to address the double-bifurcating structures (DBSs) in the unitig graph [44]. The bandage software was applied to visualize the connections of contigs to allow for the manual removal of nodes derived from the chloroplasts and nuclei [45]. CPGAVAS2(v2.0) [46] was used for chloroplast genome annotation, whereas the CPGView(v1.0) web server [47] was used for potential annotation errors in the chloroplast genome”. However, the annotation of the mitogenome was completed using the GeSeq [48] and PMGA (http://www.1kmpg.cn/mgavas/, accessed on 14 July 2025) web servers. Thereafter, Apollo software (v1.11.8) [49] was used for the manual correction of the annotation results. The chloroplast and mitochondrial genome structures were subsequently drawn with PMGmap (http://www.1kmpg.cn/pmgmap, accessed on 14 July 2025). Finally, the organelle genome sequences and the annotations were deposited into GenBank (accession nos. OR538544.1 and OR538545.1 (NC_082972.1) for the chloroplast and mitochondrial genomes, respectively) [50].

### 4.3. Identification of Tandem Sequences

MISA [18] and Tandem Repeats Finder [22] are two web servers that are used to analyze simple sequence repeats (SSRs) and long tandem repeats (LTRs), respectively. SSRs, also referred to as short tandem repeats or microsatellites, were analyzed with MISA, using the thresholds of below 10, 5, 4, and 3 units for mononucleotides, dinucleotides, trinucleotides, and tetra-/penta-/hexa-nucleotides, respectively. Moreover, LTRs were predicted with Tandem Repeats Finder using the default parameters of 7 and 2 for mismatches/indels, which were matched, respectively, with a maximal period size of 500 and a minimal alignment score of 50.

### 4.4. Identification of Repeat-Mediated Recombination

In order to identify repeat-mediated recombination, the dispersed repeat sequences of the mitochondrial genome were detected with ROUS Finder 2.0.py [25]. Thereafter, the repetitive sequences and their bilateral flanking sequences (500 bp) were extracted; these two sequences corresponded to one conformation. Later, the two sequences were integrated in silico to generate the sequences associated with the other conformations that were recombined. Next, the Nanopore long reads were mapped to four DNA sequences in two conformations, followed by counting the number of mapped reads with BWA (v0.7.17) [51] and SAMtools (v1.17) [52]. Finally, IGV software (v.2.8) was adopted to visualize the read mapping results [53]. In order to further confirm the *T. wilfordii* mitochondrial genome structure, the initial and terminal 1000 bp sequences of the genome were extracted and concatenated. Next, 1000 bp sequences were extracted and concatenated from the junction of contig10 and contig11, the junction of contig6 and contig12, and the junction of contig14 in the *Tripterygium wilfordii* mitochondrial genome. Thereafter, the Nanopore long reads were mapped to these DNA sequences using BWA (v0.7.17) [51] and SAMtools (v1.17) [52]. Finally, IGV software (v.2.8) [53] was adopted to visualize the read mapping results and verify the *T. wilfordii* mitochondrial genome structure.

### 4.5. Identification of Mitochondrial Plastid Sequences (MTPTs)

Plastid genomes are transferred to plant mitogenomes to generate MTPTs, which can influence the complexity of mitochondrial genomes and can induce the false-positive DNA barcoding paradox [54,55]. Therefore, MTPTs were identified in this study by comparing the cpgenome (OR538544.1) with the mitogenome (OR53 8545.1/NC_08 2972.1) of *T. wilfordii* using BLASTN software (v2.2.30+) [56] with default parameters. In order to confirm the identified MTPTs, the MTPT sequence and its upstream/downstream sequences (1000 bp) were obtained to create the reference genome. Thereafter, using default parameters, the Nanopore long reads were aligned to these reference sequences with BWA software (v0.7.17) [52]. Finally, IGV software (v.2.8 ) was used to visualize the Nanopore long-read mapping results to the MTPT regions [53]. The Circos package of TB tools (V1.098) was then applied to visualize the MTPT results [57,58].

### 4.6. Phylogenetic Tree Construction

A total of 9 mitochondrial genomes of Celastrales and Malpighiales species were used in the phylogenetic analysis, and *Caragana spinosa* (OQ785640.1) was selected as the out group. The following mitochondrial genomes were downloaded from the NCBI GenBank database: *Tripterygium wilfordii* (OR538545.1/NC_082972.1), *Euonymus alatus* (N C_053921.1), *Populusdavidiana* (NC_035157.1), *Populusrotundifolia* (MW566588.1), *Salixsu chowensis* (NC_029317.1), *Passiflora edulis* (NC_050950.1), *Hevea brasiliensis* (AP014526.1), *Manihot esculenta* (NC_0451 36.1), and *Jatropha curcas* (OQ603497.1). For phylogenetic analysis, the common coding sequences (CDSs) among the afore-stated species were obtained with PhyloSuite software3 (v.1.2.1) [59] and then aligned using MAFFT software4 (v7.505) [60]. Using the maximum likelihood approach, the aligned sequences were subsequently used to construct a phylogenetic tree with IQ-Tree(v2.1.4-beta) [61]. UFBoot was then used for performing bootstrap analysis involving 1000 replicates [62]. Finally, iTOL (https://itol.embl.de/, accessed on 14 July 2025) was used for visualizing the resulting phylogenetic tree [63].

### 4.7. RNA-Editing Site Identification in the PCGs of the T. wilfordii Mitogenome

In this study, to identify the RNA-editing sites within the *T. wilfordii* mitogenome, strand-specific RNA-seq data were mapped to 100 bp of the sequences in 35 PCGs and the corresponding 3′and 5′ flanking regions using BWA (Burrows–Wheeler Alignment tool) [51]; moreover, reference sequence-matching alignment reads were obtained with SAMtools (v1.17) [52]. Thereafter, REDItools [64] was used to predict the RNA-editing sites using specific parameters of frequency ≥ 0.1 and coverage ≥ 5 [15]. In order to increase the prediction accuracy, the RNA-editing sites were visualized using IGV software (v.2.8 ) [53], with a special focus on sites with frequency > 0.2. In order to exclude the impact of single-nucleotide polymorphism (SNP) sites on the findings, SNPs were also predicted, and RNA-editing sites overlapping with the results of the SNP analysis were eliminated. Similarly, when the SNPs of the *T. wilfordii* mitogenome were identified, the Illumina short reads in the whole genome were mapped to 100 base pairs of CDSs in the PCGs, the corresponding 3′and 5′ flanking regions were mapped with BWA software (v0.7.17) [51], and isolated alignment reads matching the reference sequence were mapped with SAM tools (v1.17) [52]. Subsequently, the SNP sites were analyzed using REDItools (http://code.google.com/p/reditools/m accessed on 14 July 2025) [64], with parameters requiring frequency ≥ 0.1 and coverage ≥ 5.

## 5. Conclusions

This study is the first to report the *T. wilfordii* mitochondrial genome according to the sequences obtained from Illumina short reads and Nanopore long reads. The *T. wilfordii* mitogenome is a unique chromosome. This study revealed the general features of the *T. wilfordii* mitochondrial genome through various comparative analyses, including repetitive sequence analysis, recombination analysis, homologous sequence analysis, phylogenetic analysis, and RNA-editing event analysis. Furthermore, it was found that the *T. wilfordii* mitochondrial genome exhibits no recombination conformation mediated by repetitive sequences.

## Figures and Tables

**Table 1:** Attribution of contig assembly within the *T. wilfordii* mitogenome. DBS: double-bifurcating structure.

No.	Name	Length (bp)	Bifurcation Structures
1	contig1	71,533	No
2	contig2/DBS3	5483	Yes
3	contig3	151,728	No
4	contig4	17,421	No
5	contig5/DBS1	5937	Yes
6	contig6	10,857	No
7	contig7	51,730	No
8	contig8/DBS4	1728	Yes
9	contig9	18,187	No
10	contig10	140,923	No
11	contig11	18,808	No
12	contig12	86,897	No
13	contig13/DBS2	5600	Yes
14	contig14	55,721	No
15	contig15	49,130	No
16	contig16	9875	Yes

**Table 2:** Genes predicted within the *T. wilfordii* mitogenome.

Group of Genes	Names of Genes
Subunit of ATPase	atp1, atp4, atp6, atp8, atp9
Cytochrome c biogenesis	ccmB, ccmC, ccmFN
Apocytochrome b	cob
Subunit of cytochrome c oxidase	cox1, cox2, cox3
Maturase R	matR
Transport membrane protein	mttB
Subunit of NADH dehydrogenase	nad1, nad2, nad3, nad4, nad4L, nad5, nad6, nad7, nad9
Small subunit of ribosome	rps1, rps10, rps12, rps13, rps19, rps3, rps4
Large subunit of ribosome	rpl10, rpl16, rpl2, rpl5
Subunit of succinate dehydrogenase	sdh4
Transfer RNAs	trnF–GAA(×2), trnN–GUU, trnQ–UUG, trnT–GGU, trnC–GCA, trnE–UUC, trnG–GCC(×2), trnK–UUU, tr nM–CAU, trnI–CAU, trnM–CAU (×2), trnP–UGG ×3), trnS–GCU, trnS–GGA, trnS–UGA, trnW–CCA, trnY–GUA
Ribosomal RNAs	rrn18, rrn26(×2), rrn5

**Table 3:** Statistics of dispersed repetitive sequences in the *T. wilfordii* mitochondrial genome.

ID	Repeat Length	Repeat Type	Start1	End1	Start2	End2
DRS1	9875	F	565,608	575,482	605,947	615,821
DRS2	5937	F	213,806	219,742	471,391	477,327
DRS3	5600	F	86,898	92,497	141,628	147,227
DRS4	5483	P	237,164	242,646	319,662	314,180
DRS5	1728	P	529,058	530,785	550,700	548,973
DRS6	388	P	250,458	250,845	593,826	593,439
DRS7	295	F	289,376	289,670	341,859	342,153
DRS8	288	F	260,811	261,098	301,192	301,479
DRS9	241	P	73,190	73,430	252,355	252,115
DRS10	225	P	380,076	380,300	455,856	455,632
DRS11	200	F	338,753	338,952	536,718	536,917
DRS12	165	F	523,613	523,777	552,149	552,313
DRS13	138	F	444,909	445,046	682,863	683,000
DRS14	130	F	11,917	120,46	197,372	197,501
DRS15	125	F	19,468	19,592	163,717	163,841
DRS16	120	P	409,541	409,422	250,339	250,458
DRS17	111	F	503,030	503,140	716,487	716,377
DRS18	106	F	362,472	362,577	394,369	394,474
DRS19	101	F	159,986	160,086	700,671	700,771
DRS20	98	P	227,118	227,215	716,057	715,960
DRS21	85	P	701,099	701,115	300,221	300,305
DRS22	85	F	162,454	162,538	413,199	413,283
DRS23	73	P	553,929	554,001	586,127	586,055
DRS24	73	P	226,954	227,026	716,254	716,182
DRS25	73	P	61,726	61,798	590,179	590,107
DRS26	71	F	410,796	410,866	700,746	700,816
DRS27	70	F	248,538	248,607	482,163	482,232
DRS28	70	P	244,244	244,313	576,606	576,537
DRS29	67	F	87,186	87,252	141,916	141,982
DRS30	67	F	87,186	87,252	339,733	339,799
DRS31	67	F	141,916	141,982	339,733	339,799
DRS32	65	P	195,478	195,542	657,450	657,386
DRS33	64	F	91,720	91,783	146,450	146,513
DRS34	64	F	91,720	91,783	179,993	180,056
DRS35	64	F	146,450	146,513	179,993	180,056
DRS36	63	F	182,246	182,308	216,152	216,214
DRS37	63	F	182,246	182,308	473,737	473,799
DRS38	63	F	216,152	216,214	473,737	473,799
DRS39	61	P	291,156	291,216	504,253	504,193
DRS40	61	F	57,622	57,682	30,6075	30,6135
DRS41	59	F	495,093	495,151	528,531	528,589
DRS42	58	F	47,031	47,088	693,744	693,801
DRS43	55	P	167,131	167,185	710,277	710,223
DRS44	53	F	195,516	195,568	312,661	312,713
DRS45	51	F	244,667	244,717	576,983	577,033
DRS46	50	P	442,609	442,560	248,466	248,515
DRS47	50	P	226,067	226,116	441,070	441,021

**Table 4:** List of single-nucleotide polymorphisms (SNPs) detected in the PCGs of the *T. jasminoides* mitochondrial genome. “Position” means the position of the SNPs in the nucleotide sequences of the PCGs.

Region	Position	Reference	Coverage	Base Count [A, C, G, T]	AllSubs	Frequency
atp1	69	G	439	[56, 0, 383, 0]	GA	0.13
atp1	71	A	415	[352, 63, 0, 0]	AC	0.15
atp1	74	T	469	[0, 62, 0, 407]	TC	0.13
atp1	75	G	472	[0, 0, 410, 62]	GT	0.13
atp1	76	A	448	[386, 62, 0, 0]	AC	0.14
atp1	79	G	453	[59, 0, 394, 0]	GA	0.13
cox2	698	C	302	[0, 255, 0, 47]	CT	0.16
cox2	721	C	205	[0, 141, 0, 64]	CT	0.31
matR	1753	G	450	[43, 0, 407, 0]	GA	0.1
matR	1760	A	438	[396, 0, 42, 0]	AG	0.1
nad5	83	C	253	[0, 225, 28, 0]	CG	0.11
nad5	84	C	254	[28, 226, 0, 0]	CA	0.11
nad5	1295	A	441	[374, 0, 0, 67]	AT	0.15
nad5	1301	C	428	[62, 366, 0, 0]	CA	0.14
nad5	1312	C	417	[0, 366, 0, 51]	CT	0.12
nad5	1351	C	376	[0, 322, 0, 54]	CT	0.14
nad5	1353	G	378	[53, 0, 325, 0]	GA	0.14
nad5	1361	T	356	[0, 49, 0, 307]	TC	0.14
nad5	1363	C	353	[0, 304, 0, 49]	CT	0.14
nad5	1377	G	349	[58, 0, 291, 0]	GA	0.17
nad5	1509	T	279	[0, 0, 40, 239]	TG	0.14
nad5	1510	G	278	[40, 3, 235, 0]	GA GC	0.15
nad6	26	C	425	[0, 333, 0, 92]	CT	0.22
nad6	55	C	520	[0, 376, 0, 144]	CT	0.28
nad6	154	A	727	[487, 0, 240, 0]	AG	0.33
nad6	185	C	764	[0, 502, 261, 1]	CG CT	0.34
rps3	74	A	1	[0, 0, 0, 1]	AT	1
rps4	63	G	570	[0, 0, 448, 122]	GT	0.21
rps4	75	T	550	[0, 102, 0, 448]	TC	0.19
rps4	78	A	560	[467, 0, 0, 93]	AT	0.17
rps4	87	A	525	[437, 88, 0, 0]	AC	0.17
rps4	93	C	553	[0, 472, 0, 81]	CT	0.15
rps4	100	C	540	[75, 465, 0, 0]	CA	0.14
rps4	159	T	646	[0, 0, 121, 525]	TG	0.19
rps4	185	C	652	[109, 542, 0, 1]	CA CT	0.17

## Data Availability

The organelle sequences supporting the conclusions of this article are available in GenBank (https://www.ncbi.nlm.nih.gov/, accessed on 14 July 2025), with accession numbers: OR538545.1/NC_082972.1 (mitogenome) and OR538544.1 (plastome). The sample was deposited in the Institute of Medicinal Plant Development (Beijing, China), with accession number 2023050143. The raw data have been released through GenBank, with the following accession numbers: BioProject PRJNA1067867, BioSample SAMN 39554832, and SRA database SRR27708480 (Nanopore DNA reads) and SRR27708481 (Illumina DNA reads).

## Supplementary Materials

The following supporting information can be downloaded at https://www.mdpi.com/article/10.3390/ijms26157093/s1.

## Abbreviations

The following abbreviations are used in this manuscript:

PCG	protein-coding gene
tRNA	transfer RNA
rRNA	ribosomal RNA
SSR	simple sequence repeat
LTR	long tandem repeat
DRS	dispersed repetitive sequence
MTPT	mitochondrial plastid DNA
CDS	coding sequence
MVA	mevalonate
MEP	methylerythritol phosphate
TCA	tricarboxylic acid
HR	homologous recombination
BER	base excision repair
C	cytidines
U	uridines
DBS	double-bifurcating structure
SNP	single-nucleotide polymorphism
MC	mitochondrial chromosome
IR	inverted repeat

## Appendix A

**Table A1:** Statistic summary of the sequencing data generated by Nanopore and Illumina platforms.

Sequencing Descriptor	Sequencing Platform	
	Illumina	Nanopore
Total number of nucleotides	13,177,504,500	7,231,843,596
Total number of reads	87,850,030	316,356
Mean read length (bp)	150	22,859.8

**Table A2:** Statistics of simple sequence repeats in the *T. wilfordii* mitochondrial genome.

ID	SSR Type	SSR	Size	Start	End
SSR1	p4	(TCAT)3	12	3904	3915
SSR2	p3	(TGC)4	12	14,552	14,563
SSR3	p2	(GA)5	10	18,258	18,267
SSR4	p3	(TTC)4	12	21,603	21,614
SSR5	p4	(TTCA)3	12	22,669	22,680
SSR6	p1	(T)10	10	24,476	24,485
SSR7	p2	(GA)5	10	26,947	26,956
SSR8	p1	(A)11	11	30,368	30,378
SSR9	p1	(A)10	10	36,140	36,149
SSR10	p4	(CTTA)3	12	42,128	42,139
SSR11	p2	(TA)6	12	49,292	49,303
SSR12	p2	(AT)5	10	55,853	55,862
SSR13	p1	(A)12	12	56,245	56,256
SSR14	p2	(CT)5	10	56,509	56,518
SSR15	p4	(ATCA)3	12	59,470	59,481
SSR16	p3	(CTT)4	12	63,294	63,305
SSR17	p1	(T)12	12	97,610	97,621
SSR18	p2	(TA)6	12	101,015	101,026
SSR19	p2	(TA)5	10	102,475	102,484
SSR20	p1	(T)10	10	102,491	102,500
SSR21	p1	(A)13	13	102,503	102,515
SSR22	p1	(T)10	10	103,640	103,649
SSR23	p1	(A)10	10	104,561	104,570
SSR24	p5	(TTTCT)3	15	108,551	108,565
SSR25	p4	(ATAG)3	12	108,894	108,905
SSR26	p4	(AGAA)3	12	114,292	114,303
SSR27	p4	(TAAG)3	12	114,945	114,956
SSR28	p1	(A)11	11	116,756	116,766
SSR29	p1	(T)10	10	118,096	118,105
SSR30	p2	(CT)5	10	118,358	118,367
SSR31	p2	(CT)5	10	122,012	122,021
SSR32	p3	(CAA)4	12	125,275	125,286
SSR33	p4	(CTGT)3	12	127,624	127,635
SSR34	p2	(CT)5	10	128,277	128,286
SSR35	p1	(T)10	10	130,920	130,929
SSR36	p3	(TAT)4	12	140,781	140,792
SSR37	p2	(TA)5	10	149,059	149,068
SSR38	p1	(T)12	12	149,539	149,550
SSR39	p1	(T)10	10	149,968	149,977
SSR40	p4	(GGCA)3	12	151,469	151,480
SSR41	p1	(A)12	12	160,470	160,481
SSR42	p5	(ACTAG)3	15	160,484	160,498
SSR43	p4	(TTCT)3	12	162,501	162,512
SSR44	p1	(A)10	10	163,156	163,165
SSR45	p4	(TCTT)3	12	170,024	170,035
SSR46	p2	(TC)5	10	172,086	172,095
SSR47	p2	(AG)5	10	173,151	173,160
SSR48	p4	(ATTT)3	12	177,791	177,802
SSR49	p2	(AT)6	12	178,785	178,796
SSR50	p4	(CTTT)3	12	179,931	179,942
SSR51	p1	(T)10	10	179,992	180,001
SSR52	p1	(C)11	11	182,801	182,811
SSR53	p2	(CT)5	10	186,353	186,362
SSR54	p1	(A)10	10	186,457	186,466
SSR55	p4	(TTCT)3	12	187,055	187,066
SSR56	p1	(A)10	10	188,497	188,506
SSR57	p1	(T)10	10	191,228	191,237
SSR58	p1	(T)11	11	192,751	192,761
SSR59	p4	(GGCG)3	12	197,572	197,583
SSR60	p3	(CTT)4	12	198,276	198,287
SSR61	p1	(A)11	11	198,567	198,577
SSR62	p4	(AGCC)3	12	200,534	200,545
SSR63	p4	(AGAC)3	12	204,756	204,767
SSR64	p3	(CTT)4	12	205,202	205,213
SSR65	p1	(G)10	10	215,294	215,303
SSR66	p1	(A)10	10	218,839	218,848
SSR67	p4	(AAAG)3	12	226,751	226,762
SSR68	p4	(GCCG)3	12	231,013	231,024
SSR69	p4	(CAAG)3	12	234,577	234,588
SSR70	p2	(GA)5	10	239,681	239,690
SSR71	p4	(GCAA)3	12	250,096	250,107
SSR72	p4	(TCTT)3	12	250,875	250,886
SSR73	p3	(CTT)4	12	253,352	253,363
SSR74	p4	(TACT)3	12	253,737	253,748
SSR75	p2	(AT)8	16	261,274	261,289
SSR76	p4	(GTGA)3	12	262,669	262,680
SSR77	p2	(AT)7	14	265,650	265,663
SSR78	p1	(A)10	10	266,208	266,217
SSR79	p1	(A)11	11	266,771	266,781
SSR80	p3	(TTA)4	12	275,552	275,563
SSR81	p1	(A)10	10	276,586	276,595
SSR82	p2	(TC)5	10	291,363	291,372
SSR83	p4	(AAAG)3	12	291,575	291,586
SSR84	p3	(CAA)4	12	293,222	293,233
SSR85	p4	(AAAC)3	12	296,155	296,166
SSR86	p4	(TCTT)3	12	307,547	307,558
SSR87	p2	(TC)5	10	317,136	317,145
SSR88	p3	(TCT)4	12	319,730	319,741
SSR89	p4	(TCTT)3	12	320,202	320,213
SSR90	p4	(TCAA)3	12	325,791	325,802
SSR91	p2	(AT)5	10	329,975	329,984
SSR92	p2	(CT)5	10	335,795	335,804
SSR93	p2	(TC)5	10	336,109	336,118
SSR94	p2	(TC)6	12	340,581	340,592
SSR95	p3	(GAT)4	12	340,901	340,912
SSR96	p2	(TA)6	12	342,602	342,613
SSR97	p4	(TCAG)3	12	344,772	344,783
SSR98	p3	(TCT)4	12	347,832	347,843
SSR99	p4	(TTCT)3	12	348,543	348,554
SSR100	p4	(AGAA)3	12	354,135	354,146
SSR101	p2	(AG)5	10	354,778	354,787
SSR102	p3	(GGA)4	12	357,969	357,980
SSR103	p2	(TA)5	10	360,398	360,407
SSR104	p4	(CCTT)3	12	362,330	362,341
SSR105	p3	(CTT)4	12	366,171	366,182
SSR106	p4	(GAGT)3	12	366,513	366,524
SSR107	p2	(TA)5	10	376,772	376,781
SSR108	p4	(GAAT)3	12	383,703	383,714
SSR109	p4	(CTTT)3	12	384,018	384,029
SSR110	p2	(CT)5	10	389,799	389,808
SSR111	p5	(TTAGA)3	15	390,482	390,496
SSR112	p3	(CTG)5	15	391,010	391,024
SSR113	p1	(T)10	10	395,390	395,399
SSR114	p3	(CTT)4	12	402,562	402,573
SSR115	p3	(AGA)4	12	403,830	403,841
SSR116	p2	(CT)5	10	404,825	404,834
SSR117	p4	(CTAT)3	12	408,871	408,882
SSR118	p4	(GCTA)3	12	409,575	409,586
SSR119	p1	(A)10	10	411,626	411,635
SSR120	p1	(A)13	13	412,695	412,707
SSR121	p4	(TTCT)3	12	413,246	413,257
SSR122	p1	(A)10	10	413,768	413,777
SSR123	p4	(ATAG)3	12	415,985	415,996
SSR124	p2	(CT)5	10	416,396	416,405
SSR125	p2	(CT)5	10	423,720	423,729
SSR126	p2	(AT)9	18	429,578	429,595
SSR127	p2	(GA)5	10	433,555	433,564
SSR128	p2	(AG)5	10	433,863	433,872
SSR129	p1	(A)11	11	437,201	437,211
SSR130	p1	(T)14	14	440,277	440,290
SSR131	p5	(AAAGA)3	15	450,941	450,955
SSR132	p4	(GGAT)3	12	452,636	452,647
SSR133	p4	(GGAG)3	12	453,553	453,564
SSR134	p4	(GAAA)3	12	455,204	455,215
SSR135	p5	(TCTAT)3	15	456,202	456,216
SSR136	p1	(A)14	14	456,223	456,236
SSR137	p2	(AT)5	10	458,251	458,260
SSR138	p2	(AT)5	10	458,267	458,276
SSR139	p3	(TAA)4	12	463,152	463,163
SSR140	p4	(CTTT)3	12	470,520	470,531
SSR141	p1	(G)10	10	472,879	472,888
SSR142	p1	(A)10	10	476,424	476,433
SSR143	p3	(ACT)4	12	480,790	480,801
SSR144	p1	(A)10	10	481,798	481,807
SSR145	p3	(TCC)4	12	485,671	485,682
SSR146	p1	(A)11	11	487,453	487,463
SSR147	p2	(AT)6	12	491,773	491,784
SSR148	p1	(A)11	11	495,422	495,432
SSR149	p1	(T)10	10	495,583	495,592
SSR150	p2	(TA)5	10	497,184	497,193
SSR151	p1	(T)10	10	501,990	501,999
SSR152	p4	(TTTC)3	12	510,724	510,735
SSR153	p4	(AAGA)3	12	514,161	514,172
SSR154	p1	(T)12	12	514,486	514,497
SSR155	p2	(AT)5	10	514,761	514,770
SSR156	p3	(TTA)4	12	516,658	516,669
SSR157	p1	(A)10	10	519,246	519,255
SSR158	p1	(A)11	11	520,785	520,795
SSR159	p1	(A)10	10	521,711	521,720
SSR160	p2	(TC)5	10	527,030	527,039
SSR161	p4	(TCTA)3	12	527,038	527,049
SSR162	p4	(TAGA)3	12	527,050	527,061
SSR163	p3	(CTA)4	12	527,121	527,132
SSR164	p1	(A)10	10	528,215	528,224
SSR165	p4	(AGCC)3	12	528,751	528,762
SSR166	p4	(AAAG)3	12	537,670	537,681
SSR167	p1	(T)10	10	553,153	553,162
SSR168	p4	(AAGC)3	12	556,534	556,545
SSR169	p4	(AACA)3	12	563,478	563,489
SSR170	p1	(T)10	10	566,427	566,436
SSR171	p1	(A)13	13	570,844	570,856
SSR172	p6	(ATCTAT)3	18	574,227	574,244
SSR173	p1	(A)11	11	578,831	578,841
SSR174	p1	(A)12	12	581,826	581,837
SSR175	p4	(AATG)3	12	583,827	583,838
SSR176	p2	(TA)6	12	584,679	584,690
SSR177	p4	(CTTT)3	12	592,151	592,162
SSR178	p2	(TC)5	10	593,996	594,005
SSR179	p1	(T)10	10	596,355	596,364
SSR180	p5	(GTAAT)3	15	599,235	599,249
SSR181	p1	(A)11	11	600,150	600,160
SSR182	p5	(GCCCA)3	15	603,387	603,401
SSR183	p1	(T)10	10	606,766	606,775
SSR184	p1	(A)13	13	611,183	611,195
SSR185	p6	(ATCTAT)3	18	614,566	614,583
SSR186	p4	(ATTC)3	12	623,581	623,592
SSR187	p4	(TTAG)3	12	625,006	625,017
SSR188	p4	(TTTC)3	12	625,633	625,644
SSR189	p1	(T)10	10	626,533	626,542
SSR190	p1	(T)10	10	626,867	626,876
SSR191	p1	(T)12	12	627,314	627,325
SSR192	p1	(T)11	11	627,327	627,337
SSR193	p3	(GCT)4	12	627,388	627,399
SSR194	p4	(TCGA)3	12	630,121	630,132
SSR195	p4	(TTTC)3	12	632,172	632,183
SSR196	p4	(GAAT)3	12	636,280	636,291
SSR197	p4	(AGAA)3	12	647,143	647,154
SSR198	p2	(AG)5	10	656,323	656,332
SSR199	p1	(A)10	10	656,397	656,406
SSR200	p3	(GGT)4	12	657,497	657,508
SSR201	p2	(GA)6	12	657,832	657,843
SSR202	p3	(GTT)5	15	658,242	658,256
SSR203	p1	(T)11	11	658,267	658,277
SSR204	p4	(CTTG)3	12	668,106	668,117
SSR205	p1	(T)10	10	670,382	670,391
SSR206	p4	(AAGA)3	12	670,430	670,441
SSR207	p3	(CTT)4	12	676,856	676,867
SSR208	p2	(CT)5	10	684,284	684,293
SSR209	p4	(CTTT)3	12	686,396	686,407
SSR210	p4	(ATTC)3	12	689,697	689,708
SSR211	p4	(AAAG)3	12	694,379	694,390
SSR212	p1	(C)11	11	697,246	697,256
SSR213	p2	(CT)5	10	697,892	697,901
SSR214	p1	(A)10	10	698,667	698,676
SSR215	p1	(T)10	10	699,203	699,212
SSR216	p4	(TTAA)3	12	699,408	699,419
SSR217	p4	(AAGA)3	12	700,384	700,395
SSR218	p4	(GATA)3	12	700,546	700,557
SSR219	p3	(TCT)4	12	701,847	701,858
SSR220	p3	(TCC)4	12	706,852	706,863
SSR221	p4	(AAGC)3	12	709,507	709,518
SSR222	p2	(CT)5	10	709,834	709,843
SSR223	p2	(TC)5	10	715,387	715,396
