# Supplementary material for: De Novo Hybrid Assembly of the Tripterygium wilfordii Mitochondrial Genome Provides the Chromosomal Mitochondrial DNA Structure and RNA Editing Events
Source: Int J Mol Sci. 2025 Jul 23;26(15):7093. doi: 10.3390/ijms26157093 (PMC12346498; doi:10.3390/ijms26157093)

SUPPLEMENTARY MATERIALS

De Novo Hybrid Assembly of the *Tripterygium wilfordii* Mitochondrial Genome Provides the Chromosomal Mitochondrial DNA Structure and RNA Editing Events

Yisha Cai [1,2,†], Suxin Yang [1,3†], Haimei Chen [2], Yang Ni [2], Jingling Li [2], Jinghong Zhang [1,*] and Chang Liu [2,*]
1 School of Medicine, Huaqiao University, Quanzhou 362021, China; caiyisha198999@163.com (Y.C.); nievesyang11130@163.com (S.Y.)
2 Institute of Medicinal Plant Development, Chinese Academy of Medical Sciences and Peking Union Medical College, Beijing 100193, China; hmchen@implad.ac.cn (H.C.); ny_work@126.com (Y.N.); lijingling1997@163.com (J.L.)
* Correspondence: zjh@hqu.edu.cn (J.Z.); cliu@implad.ac.cn (C.L.)
† These authors contributed equally to this work.

Table S1 Statistics of tandem repeats in the *T. wilfordii* mitochondrial genome.

ID	Indices	Period Size	Copy Number	Consensus Size	Percent Matches	Percent Indels	Score	Bases number				Entropy (0-2)
								A	C	G	T	
LTR1	12553-12650	48	2	48	98	0	187	28	27	26	17	1.98
LTR2	72422-72461	21	1.9	21	94	0	71	20	37	10	32	1.85
LTR3	72503-72556	24	2.2	25	86	6	83	24	37	9	29	1.86
LTR4	171467-171517	25	2	25	92	0	84	33	9	29	27	1.89
LTR5	201744-201786	21	2.2	19	83	8	50	16	32	9	41	1.8
LTR6	207517-207547	15	2.1	15	93	0	53	16	35	12	35	1.87
LTR7	214608-214650	18	2.4	18	84	0	59	18	20	20	39	1.93
LTR8	220048-220078	15	2.1	15	93	0	53	19	35	12	32	1.9
LTR9	227520-227580	24	2.5	24	100	0	122	9	44	31	14	1.78
LTR10	236742-236790	24	2	24	100	0	98	36	0	34	28	1.58
LTR11	246409-246448	19	2.1	19	100	0	80	22	20	7	50	1.73
LTR12	256328-256371	22	2	22	100	0	88	18	22	27	31	1.97
LTR13	259424-259459	18	2.1	17	89	5	54	19	22	16	41	1.9
LTR14	310196-310225	14	2.1	14	93	0	51	46	16	16	20	1.84
LTR15	311333-311392	18	3.6	16	72	12	57	11	36	13	38	1.81
LTR16	410726-410751	13	2	13	100	0	52	38	23	23	15	1.92
LTR17	444663-444703	18	2.3	18	83	8	57	26	17	17	39	1.91
LTR18	456933-456966	17	2	17	94	0	59	23	20	5	50	1.7
LTR19	472193-472235	18	2.4	18	84	0	59	18	20	20	39	1.93
LTR20	511475-511518	22	2	22	86	0	61	15	13	18	52	1.75
LTR21	530412-530488	38	2.1	37	92	7	129	31	24	12	31	1.93

LTR22	546948-546993	23	2	23	91	0	74	30	30	21	17	1.96
LTR23	549270-549346	38	2.1	37	92	7	129	31	12	24	31	1.93
LTR24	703967-704004	17	2.2	18	85	4	51	26	5	23	44	1.74

Table S2 Statistics of Dispersed repeats in the *T. wilfordii* mitochondrial genome. DBS: double bifurcating structures.

ID	Alignment Length (nt)	Positions of Repeat Copy 1		Positions of Repeat Copy 2		Type	Numbers of Long Reads Mapped to Each Conformation				Recombination Frequency (%)
		start	end	start	end		c1	c2	c3	c4	
DRS1	9875	565,608	575,482	605,947	615,821	F	15	11	0	0	0
DRS2/DBS1	5937	213,806	219,742	47,1391	477,327	F	21	24	0	0	0
DRS3/DBS2	5600	86,898	92,497	141,628	147,227	F	5	13	0	0	0
DRS4/DBS3	5483	237,164	242,646	319,662	314,180	P	11	12	0	0	0
DRS5/DBS4	1728	529,058	530,785	550,700	548,973	P	19	19	0	0	0
DRS6	388	250,458	250,845	593,826	593,439	P	17	14	0	0	0
DRS7	295	289,376	289,670	341,859	342,153	F	20	16	0	0	0
DRS8	288	260,811	261,098	301,192	301,479	F	20	17	0	0	0
DRS9	241	731,90	734,30	252,355	252,115	P	3	27	0	0	0
DRS10	225	380,076	380,300	455,856	455,632	P	24	20	0	0	0
DRS11	200	338,753	338,952	536,718	536,917	F	15	22	0	0	0
DRS12	165	523,613	523,777	552,149	552,313	F	17	9	0	0	0
DRS13	138	444,909	445,046	682,863	683,000	F	18	5	0	0	0
DRS14	130	11,917	12,046	197,372	197,501	F	33	24	0	0	0
DRS15	125	194,68	195,92	163,717	163,841	F	22	18	0	0	0
DRS16	120	409,541	409,422	250,339	250,458	P	18	1	0	0	0
DRS17	111	503,030	503,140	716,487	716,377	P	32	21	0	0	0
DRS18	106	362,472	362,577	394,369	394,474	F	21	19	0	0	0
DRS19	101	159,986	160,086	700,671	700,771	F	20	12	0	0	0
DRS20	98	227,118	227,215	716,057	715,960	P	6	3	0	0	0
DRS21	85	701,099	701,015	300,221	300,305	P	5	11	0	0	0
DRS22	85	162,454	162,538	413,199	413,283	F	16	13	0	0	0
DRS23	73	553,929	554,001	586,127	586,055	P	18	11	0	0	0
DRS24	73	226,954	227,026	716,254	716,182	P	4	1	0	0	0
DRS25	73	61,726	61,798	59,0179	590,107	P	4	21	0	0	0

DRS26	71	410,796	410,866	700,746	700,816	F	15	4	0	0	0
DRS27	70	248,538	248,607	482,163	482,232	F	9	33	0	0	0
DRS28	70	244,244	244,313	576,606	576,537	P	21	15	0	0	0
DRS29	67	87,186	87,252	141,916	141,982	F	4	7	0	0	0
DRS30	67	87,186	87,252	339,733	339,799	F	3	15	0	0	0
DRS31	67	141,916	141,982	339,733	339,799	F	8	17	0	0	0
DRS32	65	195,478	195,542	657,450	657,386	P	22	15	0	0	0
DRS33	64	91,720	91,783	146,450	146,513	F	17	18	0	0	0
DRS34	64	91,720	91,783	179,993	180,056	F	22	22	0	0	0
DRS35	64	146,450	146,513	179,993	180,056	F	18	22	0	0	0
DRS36	63	182,246	182,308	216,152	216,214	F	23	16	0	0	0
DRS37	63	182,246	182,308	473,737	473,799	F	30	25	0	0	0
DRS38	63	216,152	216,214	473,737	473,799	F	16	25	0	0	0
DRS39	61	291,156	291,216	504,253	504,193	P	12	34	0	0	0
DRS40	61	57,622	57,682	306,075	306,135	F	15	16	0	0	0
DRS41	59	495,093	495,151	528,531	528,589	F	24	7	0	0	0
DRS42	58	47,031	47,088	693,744	693,801	F	19	11	0	0	0
DRS43	55	167,131	167,185	710,277	710,223	P	18	44	0	0	0
DRS44	53	195,516	195,568	312,661	312,713	F	4	15	0	0	0
DRS45	51	244,667	244,717	576,983	577,033	F	0	12	0	0	0
DRS46	50	442,609	442,560	248,466	248,515	P	24	4	0	0	0
DRS47	50	226,067	226,116	441,070	441,021	P	19	22	0	0	0

Table S3 List of mitochondrial plastid DNA (MTPT) in the mitochondrial genome of *T. wilfordii*.

MTPT Number	Cpgenome Alignment		Mtgenome Alignment		Alignment Length	Identity(%)	Mismatches	Gap Openings	E-value	Bit Score	MTPT annotation
	Start	End	Start	End							
MTPT1	33,570	34,628	99,637	100,684	1048	91.407	80	7	0	1441	*partial (psbD, psbC)*
MTPT2	15,5431	155,992	282,455	281,893	563	99.467	2	1	0.00E+00	1022	partial (*rpl2*)
MTPT2	86,103	86,664	281,893	282,455	563	99.467	2	1	0.00E+00	1022	partial (*rpl2*)
MTPT3	34,751	35,073	100,667	100,996	330	87.273	35	4	3.43E-101	370	partial (*psbC*)
MTPT4	67,269	67,636	41,682	42,019	338	80.488	40	20	3.61E-66	254	complete (*trn*W-CCA, *trn*P-UGG)
MTPT5	46,106	46,287	666,089	665,909	181	86.957	19	5	1.33E-50	202	complete (*trn*S-GGA)
MTPT6	53,630	53,701	118,660	118,589	72	94.444	4	0	2.30E-23	111	complete (*trn*M-CAU)
MTPT7	154,273	154,347	274,194	274,109	86	82.558	4	7	1.81E-09	65.8	partial (*trn*I-CAU)
MTPT7	87,748	87,822	274,109	274,194	86	82.558	4	7	1.81E-09	65.8	partial (*trn*I-CAU)
MTPT8	7639	7668	665,944	665,973	30	100	0	0	1.09E-06	56.5	partial (*trn*S-GCU)

Table S4 List of the predicted RNA editing sites in the *T. wilfordii* mitochondrial genome.“CDS Position” and “AA Position” means the position of the RNA editing sites in the nucleotide and amino acid sequences of the PCGs.

Region	Position	Reference	Coverage	BaseCount[A,C,G,T]	AllSubs	Frequency	Effect	AA Effect
atp1	246	C	171,57	[3, 14255, 1, 2898]	CT CA CG	0.17	ACC→ACU	Thr→Thr
atp1	1039	C	12,837	[0, 52, 6, 12,779]	CT CG	1	CCC→UCC	Pro→Ser
atp1	1178	C	19,563	[12, 48, 6, 19,497]	CT CA CG	1	UCA→UUA	Ser→Leu
atp1	1292	C	16,517	[0, 54, 1, 16,462]	CT CG	1	CCG→CUG	Pro→Leu
atp1	1415	C	20,083	[2, 38, 2, 20,041]	CT CA CG	1	CCA→CUA	Pro→Leu
atp1	1490	C	15,124	[0, 36, 3, 15,085]	CT CG	1	CCA→CUA	Pro→Leu
atp4	23	C	4983	[1, 772, 3, 4207]	CT CG CA	0.84	UCU→UUU	Ser→Phe
atp4	53	C	5524	[1, 104, 0, 5419]	CT CA	0.98	UCA→UUA	Ser→Leu
atp4	82	C	5849	[1, 29, 1, 5818]	CT CA CG	1	CGU→UGU	Arg→Cys
atp4	179	C	8393	[0, 61, 4, 8328]	CT CG	0.99	UCG→UUG	Ser→Leu
atp4	191	C	8780	[1, 115, 2, 8662]	CT CG CA	0.99	CCC→CUC	Pro→Leu
atp4	212	C	7717	[0, 111, 1, 7605]	CT CG	0.99	CCU→CUU	Pro→Leu
atp4	215	C	8839	[0, 131, 6, 8702]	CT CG	0.99	CCG→CUG	Pro→Leu
atp4	359	C	9335	[0, 83, 3, 9249]	CT CG	0.99	UCA→UUA	Ser→Leu
atp4	380	C	8747	[0, 139, 2, 8606]	CT CG	0.98	ACU→AUU	Thr→Ile
atp6	37	C	4311	[2, 15, 3, 4291]	CT CG CA	1	CCA→UCA	Pro→Ser
atp6	116	C	6001	[0, 102, 1, 5898]	CT CG	0.98	UCA→UUA	Ser→Leu
atp6	167	C	6153	[0, 53, 2, 6098]	CT CG	0.99	CCG→CUG	Pro→Leu
atp6	173	C	6014	[7, 60, 6, 5941]	CT CA CG	0.99	CCG→CUG	Pro→Leu
atp6	201	C	6326	[3, 5634, 1688]	CT CA CG	0.11	UCC→UCU	Ser→Ser
atp6	229	C	6025	[2, 67, 2, 5954]	CT CA CG	0.99	CGC→UGC	Arg→Cys
atp6	236	C	6270	[0, 101, 5, 6164]	CT CG	0.98	UCG→UUG	Ser→Leu
atp6	254	C	6736	[0, 35, 2, 6699]	CT CG	0.99	UCG→UUG	Ser→Leu
atp6	262	C	7738	[0, 97, 4, 7637]	CT CG	0.99	CGU→UGU	Arg→Cys
atp6	270	C	7774	[0, 6678, 0, 1096]	CT	0.14	CUC→CUU	Leu→Leu

atp6	315	C	9529	[0, 7927, 1, 1601]	CT CG	0.17	AUC→AUU	Ile→Ile
atp6	401	C	12,329	[0, 49, 0, 12,280]	CT	1	UCA→UUA	Ser→Leu
atp6	460	C	9367	[0, 74, 0, 9293]	CT	0.99	CCU→UCU	Pro→Ser
atp6	463	C	8878	[0, 86, 3, 8789]	CT CG	0.99	CAU→UAU	His→Tyr
atp6	485	C	8786	[0, 105, 3, 8678]	CT CG	0.99	UCA→UUA	Ser→Leu
atp6	527	C	7683	[0, 58, 0, 7625]	CT	0.99	UCA→UUA	Ser→Leu
atp6	548	C	6795	[1, 43, 6, 6745]	CT CG CA	0.99	UCC→UUC	Ser→Phe
atp6	599	C	6882	[1, 63, 0, 6818]	CT CA	0.99	CCU→CUU	Pro→Leu
atp6	664	C	10,751	[1, 80, 0, 10,670]	CT CA	0.99	CAU→UAU	His→Tyr
atp6	671	C	10,488	[0, 72, 0, 10,416]	CT	0.99	UCU→UUU	Ser→Phe
atp6	707	C	8358	[1, 107, 1, 8249]	CT CA CG	0.99	ACA→AUA	Thr→Ile
atp6	718	C	7083	[1, 84, 0, 6998]	CT CA	0.99	CAA→UAA	Gln→End
atp8	21	C	3070	[0, 1892, 1, 1177]	CT CG	0.38	UUC→UUU	Phe→Phe
atp8	57	C	2569	[0, 2273, 0, 296]	CT	0.12	UUC→UUU	Phe→Phe
atp9	20	C	101,927	[5, 380, 53, 101,489]	CT CG CA	1	UCA→UUA	Ser→Leu
atp9	50	C	106,130	[2, 329, 18, 105,781]	CT CG CA	1	UCA→UUA	Ser→Leu
atp9	205	C	101,184	[1, 3336, 4, 97,843]	CT CG CA	0.97	CUG→UUG	Leu→Leu
atp9	212	C	98,890	[5, 457, 4, 98,424]	CT CA CG	1	UCA→UUA	Ser→Leu
atp9	223	C	96,242	[16, 477, 5, 95,744]	CT CA CG	1	CGA→UGA	Arg→End
ccmB	16	C	1397	[0, 1200, 0, 197]	CT	0.14	CUU→UUU	Leu→Phe
ccmB	28	C	1484	[0, 182, 0, 1302]	CT	0.88	CAU→UAU	His→Tyr
ccmB	43	C	1373	[0, 173, 0, 1200]	CT	0.87	CCC→UCC	Pro→Ser
ccmB	70	C	1244	[0, 1015, 0, 229]	CT	0.18	CCA→UUA	Pro→Leu
ccmB	71	C	1275	[0, 197, 0, 1078]	CT	0.85	CCA→UUA	Pro→Leu
ccmB	80	C	1090	[0, 166, 1, 923]	CT CG	0.85	UCG→UUG	Ser→Leu
ccmB	87	C	1167	[0, 403, 1, 763]	CT CG	0.65	AUC→AUU	Ile→Ile
ccmB	90	C	1237	[0, 963, 0, 274]	CT	0.22	GUC→GUU	Val→Val
ccmB	99	C	1260	[1, 791, 0, 468]	CT CA	0.37	CCC→CCU	Pro→Pro
ccmB	128	C	1465	[0, 252, 0, 1213]	CT	0.83	UCA→UUA	Ser→Leu

ccmB	137	C	1508	[0, 266, 0, 1242]	CT	0.82	UCC→UUC	Ser→Phe
ccmB	148	C	1376	[0, 290, 0, 1086]	CT	0.79	CCG→UUG	Pro→Leu
ccmB	149	C	1458	[0, 292, 0, 1166]	CT	0.8	CCG→UUG	Pro→Leu
ccmB	154	C	1530	[0, 312, 0, 1218]	CT	0.8	CGG→UGG	Arg→Trp
ccmB	160	C	1549	[0, 306, 1, 1242]	CT CG	0.8	CCU→UCU	Pro→Ser
ccmB	164	C	1527	[0, 325, 0, 1202]	CT	0.79	CCG→CUG	Pro→Leu
ccmB	179	C	1698	[0, 320, 0, 1378]	CT	0.81	CCU→CUU	Pro→Leu
ccmB	181	C	1712	[0, 302, 1, 1409]	CT CG	0.82	CCU→UCU	Pro→Ser
ccmB	193	C	1658	[0, 256, 0, 1402]	CT	0.85	CCU→UUU	Pro→Phe
ccmB	194	C	1703	[0, 280, 0, 1423]	CT	0.84	CCU→UUU	Pro→Phe
ccmB	286	C	2206	[0, 290, 1, 1915]	CT CG	0.87	CGG→UGG	Arg→Trp
ccmB	338	C	2430	[0, 318, 1, 2111]	CT CG	0.87	CCG→CUG	Pro→Leu
ccmB	367	C	2741	[0, 316, 0, 2425]	CT	0.88	CGG→UGG	Arg→Trp
ccmB	380	C	2680	[1, 339, 0, 2340]	CT CA	0.87	CCA→CUA	Pro→Leu
ccmB	424	C	2162	[0, 730, 1, 1431]	CT CG	0.66	CGU→UGU	Arg→Cys
ccmB	428	C	2074	[0, 288, 1, 1785]	CT CG	0.86	UCG→UUG	Ser→Leu
ccmB	441	C	1940	[0, 1569, 0, 371]	CT	0.19	AUC→AUU	Ile→Ile
ccmB	467	C	1724	[0, 310, 0, 1414]	CT	0.82	UCG→UUG	Ser→Leu
ccmB	475	C	1553	[0, 269, 1, 1283]	CT CG	0.83	CCA→UUA	Pro→Leu
ccmB	476	C	1552	[0, 282, 0, 1270]	CT	0.82	CCA→UUA	Pro→Leu
ccmB	485	C	1651	[0, 266, 0, 1385]	CT	0.84	UCA→UUA	Ser→Leu
ccmB	501	C	2264	[2, 1979, 0, 283]	CT CA	0.13	CCC→CCU	Pro→Pro
ccmB	502	C	2109	[8, 1735, 1, 365]	CT CA CG	0.17	CCA→UUA	Pro→Leu
ccmB	503	C	1986	[8, 260, 1, 1717]	CT CA CG	0.87	CCA→UUA	Pro→Leu
ccmB	512	C	1862	[0, 223, 0, 1639]	CT	0.88	UCU→UUU	Ser→Phe
ccmB	514	C	1880	[0, 194, 0, 1686]	CT	0.9	CGU→UGU	Arg→Cys
ccmB	551	C	2128	[0, 221, 0, 1907]	CT	0.9	UCA→UUA	Ser→Leu
ccmB	554	C	2153	[0, 329, 0, 1824]	CT	0.85	UCG→UUG	Ser→Leu
ccmB	566	C	2332	[0, 238, 0, 2094]	CT	0.9	UCC→UUC	Ser→Phe

ccmB	569	C	2069	[0, 189, 0, 1880]	CT	0.91	UCU→UUU	Ser→Phe
ccmB	572	C	2174	[0, 184, 0, 1990]	CT	0.92	CCG→CUG	Pro→Leu
ccmB	576	C	2244	[0, 1356, 0, 888]	CT	0.4	UUC→UUU	Phe→Phe
ccmB	596	C	2223	[0, 222, 0, 2001]	CT	0.9	UCG→UUG	Ser→Leu
ccmB	600	C	2228	[0, 563, 0, 1665]	CT	0.75	GUC→GUU	Val→Val
ccmB	601	C	2276	[0, 250, 0, 2026]	CT	0.89	CCG→UCG	Pro→Ser
ccmC	5	C	8269	[1, 640, 1, 7627]	CT CA CG	0.92	UCC→UUC	Ser→Phe
ccmC	76	C	5356	[0, 154, 3, 5199]	CT CG	0.97	CGG→UGG	Arg→Trp
ccmC	103	C	5948	[1, 205, 0, 5742]	CT CA	0.97	CAU→UAU	His→Tyr
ccmC	115	C	6540	[0, 186, 2, 6352]	CT CG	0.97	CGG→UGG	Arg→Trp
ccmC	133	C	7817	[0, 253, 2, 7562]	CT CG	0.97	CUU→UUU	Leu→Phe
ccmC	179	C	6797	[7, 115, 8, 6667]	CT CG CA	0.98	GCG→GUG	Ala→Val
ccmC	184	C	7408	[0, 103, 5, 7300]	CT CG	0.99	CGG→UGG	Arg→Trp
ccmC	281	C	6901	[0, 480, 0, 6421]	CT	0.93	ACA→AUA	Thr→Ile
ccmC	306	C	6869	[0, 3805, 3, 3061]	CT CG	0.45	UUC→UUU	Phe→Phe
ccmC	331	C	8299	[0, 159, 2, 8138]	CT CG	0.98	CGG→UGG	Arg→Trp
ccmC	395	C	9942	[0, 391, 3, 9548]	CT CG	0.96	UCG→UUG	Ser→Leu
ccmC	399	C	9720	[0, 93, 2, 9625]	CT CG	0.99	UUC→UUU	Phe→Phe
ccmC	400	C	9594	[0, 290, 0, 9304]	CT	0.97	CUU→UUU	Leu→Phe
ccmC	421	C	10,604	[0, 432, 1, 10,171]	CT CG	0.96	CGU→UGU	Arg→Cys
ccmC	436	C	11,563	[1, 227, 4, 11,331]	CT CG CA	0.98	CCU→UCU	Pro→Ser
ccmC	446	C	10,531	[1, 356, 3, 10,171]	CT CG CA	0.97	CCG→CUG	Pro→Leu
ccmC	458	C	11,294	[5, 687, 1, 10,601]	CT CA CG	0.94	UCA→UUA	Ser→Leu
ccmC	463	C	11,348	[1, 630, 1, 10,716]	CT CA CG	0.94	CGU→UGU	Arg→Cys
ccmC	467	C	11,626	[1, 1030, 2, 10,593]	CT CG CA	0.91	GCU→GUU	Ala→Val
ccmC	473	C	12,234	[0, 324, 6, 11,904]	CT CG	0.97	CCG→CUG	Pro→Leu
ccmC	497	C	11,227	[1, 856, 2, 10,368]	CT CG CA	0.92	UCU→UUU	Ser→Phe
ccmC	521	C	8838	[0, 98, 2, 8738]	CT CG	0.99	UCG→UUG	Ser→Leu
ccmC	548	C	7561	[0, 658, 0, 6903]	CT	0.91	UCU→UUU	Ser→Phe

ccmC	568	C	12,226	[0, 456, 1, 11,769]	CT CG	0.96	CCU→UCU	Pro→Ser
ccmC	575	C	12,514	[0, 688, 1, 11,825]	CT CG	0.95	CCC→CUC	Pro→Leu
ccmC	605	C	11,869	[1, 402, 3, 11,463]	CT CG CA	0.97	UCC→UUU	Ser→Phe
ccmC	606	C	12,084	[0, 10119, 0, 1965]	CT	0.16	UCC→UUU	Ser→Phe
ccmC	614	C	11,536	[0, 203, 2, 11,331]	CT CG	0.98	UCA→UUA	Ser→Leu
ccmC	618	C	10,925	[1, 9661, 1, 1262]	CT CA CG	0.12	ACC→ACU	Thr→Thr
ccmC	619	C	10,470	[1, 425, 4, 10,040]	CT CG CA	0.96	CGU→UGU	Arg→Cys
ccmC	624	C	10,518	[0, 651, 0, 9867]	CT	0.94	AUC→AUU	Ile→Ile
ccmC	650	C	9201	[2, 375, 0, 8824]	CT CA	0.96	CCU→CUU	Pro→Leu
ccmC	656	C	8967	[0, 144, 0, 8823]	CT	0.98	CCA→CUA	Pro→Leu
ccmC	673	C	8618	[3, 415, 0, 8200]	CT CA	0.95	CCU→UCU	Pro→Ser
ccmFN	38	C	1009	[0, 45, 2, 962]	CT CG	0.96	CCG→CUG	Pro→Leu
ccmFN	98	C	1038	[0, 15, 1, 1022]	CT CG	0.99	CCU→CUU	Pro→Leu
ccmFN	137	C	725	[0, 19, 0, 706]	CT	0.97	UCG→UUG	Ser→Leu
ccmFN	142	C	743	[0, 7, 0, 736]	CT	0.99	CGU→UGU	Arg→Cys
ccmFN	248	C	1121	[0, 2, 1, 1118]	CT CG	1	UCA→UUA	Ser→Leu
ccmFN	256	C	1213	[0, 11, 0, 1202]	CT	0.99	CGG→UGG	Arg→Trp
ccmFN	263	C	1284	[0, 20, 0, 1264]	CT	0.98	CCA→CUA	Pro→Leu
ccmFN	283	C	1334	[0, 18, 0, 1316]	CT	0.99	CUU→UUU	Leu→Phe
ccmFN	375	C	1362	[0, 510, 0, 852]	CT	0.63	UUC→UUU	Phe→Phe
ccmFN	520	C	1178	[0, 820, 0, 358]	CT	0.3	CUU→UUU	Leu→Phe
ccmFN	539	C	1194	[4, 948, 0, 242]	CT CA	0.2	CCG→CUG	Pro→Leu
ccmFN	546	C	1176	[0, 900, 0, 276]	CT	0.23	UAC→UAU	Tyr→Tyr
ccmFN	619	C	1166	[0, 959, 0, 207]	CT	0.18	CAU→UAU	His→Tyr
ccmFN	719	C	947	[0, 16, 0, 931]	CT	0.98	CCU→CUU	Pro→Leu
ccmFN	728	C	922	[0, 10, 3, 909]	CT CG	0.99	UCA→UUA	Ser→Leu
ccmFN	738	C	935	[0, 821, 1, 113]	CT CG	0.12	UCC→UCU	Ser→Ser
ccmFN	766	C	996	[0, 15, 0, 981]	CT	0.98	CGU→UGU	Arg→Cys
ccmFN	771	C	909	[2, 787, 0, 120]	CT CA	0.13	ACC→ACU	Thr→Thr

ccmFN	788	C	935	[0, 8, 0, 927]	CT	0.99	UCA→UUA	Ser→Leu
ccmFN	800	C	1046	[0, 22, 0, 1024]	CT	0.98	CCA→CUA	Pro→Leu
ccmFN	815	C	1029	[0, 12, 2, 1015]	CT CG	0.99	UCA→UUA	Ser→Leu
ccmFN	913	C	1586	[0, 1419, 0, 167]	CT	0.11	CAC→UAC	His→Tyr
ccmFN	964	C	1462	[0, 15, 2, 1445]	CT CG	0.99	CGC→UGC	Arg→Cys
ccmFN	1199	C	1552	[1, 169, 0, 1382]	CT CA	0.89	UCG→UUG	Ser→Leu
ccmFN	1282	C	1126	[1, 9, 1, 1115]	CT CA CG	0.99	CGG→UGG	Arg→Trp
ccmFN	1310	C	1232	[0, 9, 0, 1223]	CT	0.99	CCA→CUA	Pro→Leu
ccmFN	1327	C	1334	[0, 27, 0, 1307]	CT	0.98	CAU→UAU	His→Tyr
ccmFN	1342	C	1407	[0, 20, 0, 1387]	CT	0.99	CGG→UGG	Arg→Trp
ccmFN	1360	C	1793	[0, 15, 0, 1778]	CT	0.99	CGG→UGG	Arg→Trp
ccmFN	1393	C	1853	[1, 21, 0, 1831]	CT CA	0.99	CGG→UGG	Arg→Trp
ccmFN	1411	C	2214	[0, 50, 4, 2160]	CT CG	0.98	CGU→UGU	Arg→Cys
ccmFN	1435	C	2227	[1, 1428, 1, 797]	CT CA CG	0.36	CUU→UUU	Leu→Phe
ccmFN	1454	C	2151	[0, 1755, 0, 396]	CT	0.18	UCC→UUC	Ser→Phe
ccmFN	1474	C	2139	[0, 12, 0, 2127]	CT	0.99	CUU→UUU	Leu→Phe
ccmFN	1478	C	2165	[0, 10, 0, 2155]	CT	1	CCA→CUA	Pro→Leu
ccmFN	1490	C	2251	[0, 12, 1, 2238]	CT CG	0.99	UCA→UUA	Ser→Leu
ccmFN	1525	C	2243	[0, 31, 2, 2210]	CT CG	0.99	CCC→UCC	Pro→Ser
cob	47	C	4899	[0, 239, 2, 4658]	CT CG	0.95	UCC→UUC	Ser→Phe
cob	114	C	6462	[0, 5319, 2, 1141]	CT CG	0.18	UUC→UUU	Phe→Phe
cob	180	C	6594	[1, 5289, 1, 1303]	CT CA CG	0.2	UAC→UAU	Tyr→Tyr
cob	286	C	8524	[0, 97, 7, 8420]	CT CG	0.99	CUC→UUC	Leu→Phe
cob	298	C	9299	[2, 121, 6, 9170]	CT CG CA	0.99	CAC→UAC	His→Tyr
cob	325	C	9402	[0, 92, 1, 9309]	CT CG	0.99	CAU→UAU	His→Tyr
cob	381	C	11,919	[3, 10022, 2, 1892]	CT CA CG	0.16	UUC→UUU	Phe→Phe
cob	407	C	12,135	[0, 81, 2, 12,052]	CT CG	0.99	ACA→AUA	Thr→Ile
cob	568	C	9771	[0, 107, 0, 9664]	CT	0.99	CAU→UAU	His→Tyr
cob	853	C	13,725	[0, 69, 2, 13,654]	CT CG	0.99	CAU→UAU	His→Tyr

cob	908	C	11,423	[1, 43, 2, 11,377]	CT CG CA	1	CCA→CUA	Pro→Leu
cob	982	C	9589	[0, 356, 1, 9232]	CT CG	0.96	CAC→UAC	His→Tyr
cob	1081	C	9828	[1, 57, 2, 9768]	CT CG CA	0.99	CCU→UCU	Pro→Ser
cob	1084	C	9977	[0, 53, 3, 9921]	CT CG	0.99	CCU→UCU	Pro→Ser
cob	1098	C	14,811	[1, 13358, 3, 1449]	CT CG CA	0.1	UUC→UUU	Phe→Phe
cob	1124	C	14,796	[2, 72, 2, 14,720]	CT CA CG	1	CCG→CUG	Pro→Leu
cob	1169	C	12,795	[0, 1576, 1, 11,218]	CT CG	0.88	ACU→AUU	Thr→Ile
cob	1179	C	12,073	[1, 10474, 0, 1598]	CT CA	0.13	ACC→ACU	Thr→Thr
cox1	11	C	2877	[0, 74, 0, 2803]	CT	0.97	CCG→CUG	Pro→Leu
cox1	15	C	2984	[0, 2685, 1, 298]	CT CG	0.1	GUC→GUU	Val→Val
cox1	242	C	7691	[0, 34, 3, 7654]	CT CG	1	UCU→UUU	Ser→Phe
cox1	254	C	8538	[0, 62, 3, 8473]	CT CG	0.99	UCU→UUU	Ser→Phe
cox1	443	C	9713	[3, 66, 2, 9642]	CT CA CG	0.99	UCA→UUA	Ser→Leu
cox1	452	C	9873	[0, 39, 3, 9831]	CT CG	1	UCU→UUU	Ser→Phe
cox1	515	C	13,420	[4, 66, 4, 13,346]	CT CA CG	1	UCC→UUU	Ser→Phe
cox1	516	C	13,033	[0, 11737, 1, 1295]	CT CG	0.1	UCC→UUU	Ser→Phe
cox1	551	C	12,521	[1, 61, 1, 12,458]	CT CA CG	1	UCA→UUA	Ser→Leu
cox1	555	C	11,822	[1, 10630, 5, 1186]	CT CG CA	0.1	CCC→CCU	Pro→Pro
cox1	570	C	11,122	[1, 9160, 4, 1957]	CT CG CA	0.18	UCC→UCU	Ser→Ser
cox1	590	C	11,554	[1, 69, 0, 11,484]	CT CA	0.99	CCA→CUA	Pro→Leu
cox1	668	C	11,033	[0, 34, 5, 10,994]	CT CG	1	UCU→UUU	Ser→Phe
cox1	715	C	10,573	[0, 30, 1, 10,542]	CT CG	1	CGG→UGG	Arg→Trp
cox1	746	C	10,667	[1, 33, 1, 10,632]	CT CA CG	1	CCC→CUC	Pro→Leu
cox1	761	C	10,121	[0, 27, 3, 10,091]	CT CG	1	UCC→UUC	Ser→Phe
cox1	1038	C	6496	[4, 5049, 2, 1441]	CT CA CG	0.22	UUC→UUU	Phe→Phe
cox1	1186	C	12,955	[1, 38, 1, 12,915]	CT CA CG	1	CAC→UAC	His→Tyr
cox1	1405	C	10,006	[0, 36, 0, 9970]	CT	1	CGU→UGU	Arg→Cys
cox1	1413	C	9465	[0, 7358, 2, 2105]	CT CG	0.22	UUC→UUU	Phe→Phe
cox1	1433	C	9302	[0, 28, 1, 9273]	CT CG	1	UCA→UUA	Ser→Leu

cox1	1489	C	16,971	[0, 43, 0, 16,928]	CT	1	CCA→UCA	Pro→Ser
cox2	71	C	7558	[0, 62, 3, 7493]	CT CG	0.99	UCU→UUU	Ser→Phe
cox2	138	C	8748	[0, 7866, 1, 881]	CT CG	0.1	CUC→CUU	Leu→Leu
cox2	163	C	9928	[2, 143, 4, 9779]	CT CG CA	0.99	CGG→UGG	Arg→Trp
cox2	253	C	14,275	[1, 309, 2, 13,963]	CT CG CA	0.98	CGG→UGG	Arg→Trp
cox2	278	C	13,621	[2, 178, 1, 13,440]	CT CA CG	0.99	CCG→CUG	Pro→Leu
cox2	379	C	11,524	[0, 84, 4, 11,436]	CT CG	0.99	CGG→UGG	Arg→Trp
cox2	426	C	12,484	[1, 9716, 7, 2760]	CT CG CA	0.22	CUC→CUU	Leu→Leu
cox2	443	C	12,145	[0, 129, 3, 12,013]	CT CG	0.99	ACG→AUG	Thr→Met
cox2	476	C	12,531	[0, 78, 3, 12,450]	CT CG	0.99	UCA→UUA	Ser→Leu
cox2	544	C	10,516	[0, 38, 3, 10,475]	CT CG	1	CCU→UCU	Pro→Ser
cox2	557	C	10,173	[3, 33, 0, 10,137]	CT CA	1	CCU→CUU	Pro→Leu
cox2	581	C	8425	[0, 28, 3, 8394]	CT CG	1	UCA→UUA	Ser→Leu
cox2	632	C	10,443	[1, 21, 3, 10,418]	CT CG CA	1	UCG→UUG	Ser→Leu
cox2	698	C	10,175	[0, 25, 1, 10,149]	CT CG	1	ACG→AUG	Thr→Met
cox2	721	C	10,611	[0, 156, 1, 10,454]	CT CG	0.99	CCU→UCU	Pro→Ser
cox2	742	C	10,639	[1, 19, 2, 10,617]	CT CG CA	1	CGG→UGG	Arg→Trp
cox3	289	C	7833	[0, 118, 1, 7714]	CT CG	0.98	CUU→UUU	Leu→Phe
cox3	311	C	8041	[0, 152, 1, 7888]	CT CG	0.98	UCU→UUU	Ser→Phe
cox3	413	C	9710	[13, 91, 20, 9586]	CT CG CA	0.99	CCU→CUU	Pro→Leu
cox3	512	C	12,849	[0, 2417, 4, 10,428]	CT CG	0.81	UCA→UUA	Ser→Leu
cox3	527	C	13,046	[0, 138, 2, 12,906]	CT CG	0.99	UCC→UUC	Ser→Phe
cox3	754	C	6589	[0, 41, 1, 6547]	CT CG	0.99	CGG→UGG	Arg→Trp
matR	32	C	674	[0, 159, 0, 515]	CT	0.76	UCC→UUC	Ser→Phe
matR	43	C	644	[0, 185, 0, 459]	CT	0.71	CCC→UCC	Pro→Ser
matR	326	C	995	[0, 208, 0, 787]	CT	0.79	CCA→CUA	Pro→Leu
matR	413	C	987	[0, 101, 1, 885]	CT CG	0.9	UCG→UUG	Ser→Leu
matR	850	C	1436	[0, 1105, 0, 331]	CT	0.23	CUG→UUG	Leu→Leu
matR	1545	C	1725	[9, 339, 5, 1372]	CT CA CG	0.8	CCC→CCU	Pro→Pro

matR	1679	C	2085	[0, 191, 1, 1893]	CT CG	0.91	UCC→UUC	Ser→Phe
matR	1700	C	1996	[0, 362, 0, 1634]	CT	0.82	CCU→CUU	Pro→Leu
matR	1720	C	2081	[0, 401, 0, 1680]	CT	0.81	CGC→UGC	Arg→Cys
matR	1734	C	2141	[0, 453, 1, 1687]	CT CG	0.79	UAC→UAU	Tyr→Tyr
matR	1756	C	1783	[0, 183, 2, 1598]	CT CG	0.9	CAC→UAC	His→Tyr
matR	1826	C	1593	[0, 110, 0, 1483]	CT	0.93	CCA→CUA	Pro→Leu
matR	1844	C	1478	[0, 152, 1, 1325]	CT CG	0.9	UCA→UUA	Ser→Leu
matR	1874	C	1450	[0, 1166, 1, 283]	CT CG	0.2	ACC→AUU	Thr→Ile
matR	1875	C	1477	[0, 1005, 0, 472]	CT	0.32	ACC→AUU	Thr→Ile
mttB	5	C	879	[0, 160, 0, 719]	CT	0.82	UCC→UUC	Ser→Phe
mttB	16	C	1002	[0, 280, 0, 722]	CT	0.72	CAU→UAU	His→Tyr
mttB	25	C	944	[0, 415, 0, 529]	CT	0.56	CCG→UUG	Pro→Leu
mttB	26	C	1002	[0, 117, 0, 885]	CT	0.88	CCG→UUG	Pro→Leu
mttB	37	C	1104	[0, 709, 0, 395]	CT	0.36	CUA→UUA	Leu→Leu
mttB	59	C	1440	[0, 287, 1, 1152]	CT CG	0.8	UCC→UUC	Ser→Phe
mttB	64	C	1500	[0, 30, 0, 1470]	CT	0.98	CGG→UGG	Arg→Trp
mttB	75	C	1715	[0, 1020, 0, 695]	CT	0.41	AUC→AUU	Ile→Ile
mttB	111	C	2086	[0, 1748, 0, 338]	CT	0.16	UUC→UUU	Phe→Phe
mttB	112	C	2112	[1, 273, 1, 1837]	CT CA CG	0.87	CCG→UCG	Pro→Ser
mttB	130	C	2143	[0, 1896, 0, 247]	CT	0.12	CCA→UUA	Pro→Leu
mttB	131	C	2167	[0, 30, 0, 2137]	CT	0.99	CCA→UUA	Pro→Leu
mttB	178	C	2411	[0, 61, 0, 2350]	CT	0.97	CGU→UGU	Arg→Cys
mttB	188	C	2326	[2, 106, 2, 2216]	CT CA CG	0.95	UCA→UUA	Ser→Leu
mttB	202	C	2302	[0, 456, 0, 1846]	CT	0.8	CCG→UCG	Pro→Ser
mttB	262	C	2350	[0, 110, 0, 2240]	CT	0.95	CAU→UAU	His→Tyr
mttB	331	C	2906	[0, 568, 0, 2338]	CT	0.8	CAU→UAU	His→Tyr
mttB	346	C	3025	[0, 170, 0, 2855]	CT	0.94	CGC→UGC	Arg→Cys
mttB	353	C	2915	[0, 912, 0, 2003]	CT	0.69	UCC→UUU	Ser→Phe
mttB	354	C	2788	[0, 2370, 0, 418]	CT	0.15	UCC→UUU	Ser→Phe

mttB	373	C	2500	[0, 575, 2, 1923]	CT CG	0.77	CCU→UUU	Pro→Phe
mttB	374	C	2612	[1, 574, 0, 2037]	CT CA	0.78	CCU→UUU	Pro→Phe
mttB	376	C	2626	[0, 30, 0, 2596]	CT	0.99	CCC→UCC	Pro→Ser
mttB	379	C	2624	[0, 643, 0, 1981]	CT	0.75	CGG→UGG	Arg→Trp
mttB	472	C	2795	[0, 189, 0, 2606]	CT	0.93	CAU→UAU	His→Tyr
mttB	497	C	3834	[0, 77, 4, 3753]	CT CG	0.98	UCG→UUG	Ser→Leu
mttB	505	C	3928	[0, 88, 1, 3839]	CT CG	0.98	CCA→UCA	Pro→Ser
mttB	541	C	3298	[1, 64, 1, 3232]	CT CA CG	0.98	CGU→UGU	Arg→Cys
mttB	548	C	3266	[0, 300, 1, 2965]	CT CG	0.91	CCA→CUA	Pro→Leu
mttB	554	C	3082	[0, 50, 2, 3030]	CT CG	0.98	CCA→CUA	Pro→Leu
mttB	578	C	2606	[0, 240, 2, 2364]	CT CG	0.91	UCC→UUC	Ser→Phe
mttB	610	C	2876	[0, 98, 1, 2777]	CT CG	0.97	CCG→UCG	Pro→Ser
mttB	616	C	2731	[0, 110, 0, 2621]	CT	0.96	CUC→UUC	Leu→Phe
mttB	660	C	3076	[0, 201, 0, 2875]	CT	0.93	AUC→AUU	Ile→Ile
mttB	667	C	3048	[0, 47, 1, 3000]	CT CG	0.98	CGU→UGU	Arg→Cys
mttB	672	C	3278	[0, 156, 0, 3122]	CT	0.95	UUC→UUU	Phe→Phe
mttB	713	C	3018	[0, 45, 0, 2973]	CT	0.99	UCG→UUG	Ser→Leu
nad1	2	C	2988	[1, 982, 1, 2004]	CT CA CG	0.67	ACG→AUG	Thr→Met
nad1	40	C	3177	[0, 2526, 1, 650]	CT CG	0.2	CUA→UUA	Leu→Leu
nad1	167	C	4438	[1, 94, 0, 4343]	CT CA	0.98	UCG→UUG	Ser→Leu
nad1	215	C	4820	[1, 47, 1, 4771]	CT CA CG	0.99	UCC→UUU	Ser→Phe
nad1	216	C	4617	[1, 4060, 0, 556]	CT CA	0.12	UCC→UUU	Ser→Phe
nad1	265	C	4840	[0, 55, 1, 4784]	CT CG	0.99	CGG→UGG	Arg→Trp
nad1	307	C	5163	[0, 99, 0, 5064]	CT	0.98	CCG→UUG	Pro→Leu
nad1	308	C	5089	[0, 99, 0, 4990]	CT	0.98	CCG→UUG	Pro→Leu
nad1	368	C	4181	[0, 88, 1, 4092]	CT CG	0.98	ACA→AUA	Thr→Ile
nad1	376	C	4660	[0, 35, 4, 4621]	CT CG	0.99	CGG→UGG	Arg→Trp
nad1	436	C	5969	[2, 102, 2, 5863]	CT CA CG	0.98	CCU→UCU	Pro→Ser
nad1	490	C	4989	[0, 53, 3, 4933]	CT CG	0.99	CCC→UCU	Pro→Ser

nad1	492	C	4578	[0, 76, 1, 4501]	CT CG	0.98	CCC→UCU	Pro→Ser
nad1	493	C	4604	[0, 76, 4, 4524]	CT CG	0.98	CGU→UGU	Arg→Cys
nad1	500	C	5787	[0, 107, 0, 5680]	CT	0.98	UCG→UUG	Ser→Leu
nad1	536	C	5815	[1, 115, 0, 5699]	CT CA	0.98	UCU→UUU	Ser→Phe
nad1	573	C	6348	[0, 64, 0, 6284]	CT	0.99	UUC→UUU	Phe→Phe
nad1	580	C	6260	[0, 25, 3, 6232]	CT CG	1	CGU→UGU	Arg→Cys
nad1	635	C	7014	[0, 106, 1, 6907]	CT CG	0.98	UCA→UUA	Ser→Leu
nad1	725	C	6758	[4, 91, 5, 6658]	CT CG CA	0.99	CCA→CUA	Pro→Leu
nad1	734	C	7007	[0, 42, 3, 6962]	CT CG	0.99	UCG→UUG	Ser→Leu
nad1	738	C	6867	[0, 5897, 1, 969]	CT CG	0.14	CUC→CUU	Leu→Leu
nad1	740	C	6765	[0, 84, 4, 6677]	CT CG	0.99	UCU→UUU	Ser→Phe
nad1	743	C	6443	[0, 90, 3, 6350]	CT CG	0.99	CCA→CUA	Pro→Leu
nad1	755	C	8481	[2, 120, 3, 8356]	CT CG CA	0.99	CCG→CUG	Pro→Leu
nad1	802	C	7098	[0, 30, 1, 7067]	CT CG	1	CGG→UGG	Arg→Trp
nad1	823	C	6690	[0, 56, 0, 6634]	CT	0.99	CUC→UUC	Leu→Phe
nad1	898	C	8797	[1, 46, 1, 8749]	CT CA CG	0.99	CGG→UGG	Arg→Trp
nad1	928	C	8704	[8, 53, 9, 8634]	CT CG CA	0.99	CGG→UGG	Arg→Trp
nad1	937	C	8770	[1, 38, 1, 8730]	CT CA CG	1	CCC→UCU	Pro→Ser
nad1	939	C	8683	[0, 3295, 0, 5388]	CT	0.62	CCC→UCU	Pro→Ser
nad2	26	C	1578	[0, 54, 1, 1523]	CT CG	0.97	UCC→UUC	Ser→Phe
nad2	56	C	1514	[0, 21, 0, 1493]	CT	0.99	UCC→UUU	Ser→Phe
nad2	57	C	1635	[0, 59, 0, 1576]	CT	0.96	UCC→UUU	Ser→Phe
nad2	223	C	3123	[0, 10, 0, 3113]	CT	1	CUU→UUU	Leu→Phe
nad2	252	C	3242	[0, 1785, 0, 1457]	CT	0.45	UUC→UUU	Phe→Phe
nad2	303	C	2819	[0, 474, 3, 2342]	CT CG	0.83	UUC→UUU	Phe→Phe
nad2	308	C	2780	[0, 31, 0, 2749]	CT	0.99	UCU→UUU	Ser→Phe
nad2	311	C	2919	[0, 11, 1, 2907]	CT CG	1	UCC→UUC	Ser→Phe
nad2	367	C	3983	[2, 68, 2, 3911]	CT CA CG	0.98	CGC→UGC	Arg→Cys
nad2	394	C	3799	[1, 94, 0, 3704]	CT CA	0.98	CAU→UAU	His→Tyr

nad2	401	C	4121	[0, 62, 1, 4058]	CT CG	0.98	UCA→UUA	Ser→Leu
nad2	428	C	4166	[0, 25, 2, 4139]	CT CG	0.99	CCU→CUU	Pro→Leu
nad2	497	C	3785	[0, 19, 3, 3763]	CT CG	0.99	UCG→UUG	Ser→Leu
nad2	523	C	3730	[0, 13, 1, 3716]	CT CG	1	CCC→UCC	Pro→Ser
nad2	609	C	4600	[1, 1434, 2, 3163]	CT CG CA	0.69	ACC→ACU	Thr→Thr
nad2	662	C	4378	[0, 62, 0, 4316]	CT	0.99	UCU→UUU	Ser→Phe
nad2	800	C	5812	[0, 24, 2, 5786]	CT CG	1	UCA→UUA	Ser→Leu
nad2	809	C	5406	[0, 20, 1, 5385]	CT CG	1	UCU→UUU	Ser→Phe
nad2	855	C	5520	[1, 4862, 1, 656]	CT CA CG	0.12	UUC→UUU	Phe→Phe
nad2	920	C	6702	[0, 48, 1, 6653]	CT CG	0.99	CCU→CUU	Pro→Leu
nad2	928	C	7185	[0, 49, 3, 7133]	CT CG	0.99	CAU→UAU	His→Tyr
nad2	958	C	8258	[0, 37, 2, 8219]	CT CG	1	CGU→UGU	Arg→Cys
nad2	962	C	7249	[0, 56, 2, 7191]	CT CG	0.99	ACU→AUU	Thr→Ile
nad2	1014	C	8588	[0, 6640, 1, 1947]	CT CG	0.23	CUC→CUU	Leu→Leu
nad2	1058	C	9775	[0, 27, 3, 9745]	CT CG	1	UCA→UUA	Ser→Leu
nad2	1127	C	8941	[0, 31, 0, 8910]	CT	1	UCG→UUG	Ser→Leu
nad2	1298	C	9271	[0, 37, 0, 9234]	CT	1	GCG→GUG	Ala→Val
nad2	1400	C	7358	[0, 19, 1, 7338]	CT CG	1	UCA→UUA	Ser→Leu
nad2	1403	C	6893	[0, 37, 1, 6855]	CT CG	0.99	UCC→UUC	Ser→Phe
nad2	1408	C	6411	[0, 3827, 0, 2584]	CT	0.4	CUA→UUA	Leu→Leu
nad2	1416	C	5058	[1, 53, 0, 5004]	CT CA	0.99	CCC→CCU	Pro→Pro
nad2	1457	C	2936	[0, 15, 0, 2921]	CT	0.99	UCA→UUA	Ser→Leu
nad3	5	C	1975	[0, 17, 1, 1957]	CT CG	0.99	UCA→UUA	Ser→Leu
nad3	39	C	1897	[0, 43, 1, 1853]	CT CG	0.98	AUC→AUU	Ile→Ile
nad3	43	C	1773	[0, 711, 0, 1062]	CT	0.6	CCG→UUG	Pro→Leu
nad3	44	C	1946	[0, 34, 0, 1912]	CT	0.98	CCG→UUG	Pro→Leu
nad3	61	C	1981	[0, 234, 0, 1747]	CT	0.88	CCA→UUA	Pro→Leu
nad3	62	C	2014	[0, 87, 0, 1927]	CT	0.96	CCA→UUA	Pro→Leu
nad3	80	C	2073	[0, 33, 1, 2039]	CT CG	0.98	CCA→CUA	Pro→Leu

nad3	102	C	2152	[2, 1827, 0, 323]	CT CA	0.15	ACC→ACU	Thr→Thr
nad3	126	C	1762	[0, 1543, 1, 218]	CT CG	0.12	UAC→UAU	Tyr→Tyr
nad3	146	C	1808	[0, 11, 0, 1797]	CT	0.99	UCC→UUC	Ser→Phe
nad3	168	C	1652	[0, 1417, 1, 234]	CT CG	0.14	UUC→UUU	Phe→Phe
nad3	208	C	1518	[0, 35, 0, 1483]	CT	0.98	CCU→UUU	Pro→Phe
nad3	209	C	1557	[0, 25, 2, 1530]	CT CG	0.98	CCU→UUU	Pro→Phe
nad3	230	C	1898	[0, 23, 1, 1874]	CT CG	0.99	UCC→UUC	Ser→Phe
nad3	247	C	2340	[1, 121, 0, 2218]	CT CA	0.95	CCU→UCU	Pro→Ser
nad3	251	C	2198	[0, 184, 0, 2014]	CT	0.92	CCC→CUC	Pro→Leu
nad3	266	C	2418	[0, 132, 0, 2286]	CT	0.95	CCG→CUG	Pro→Leu
nad3	317	C	2000	[0, 16, 0, 1984]	CT	0.99	UCU→UUU	Ser→Phe
nad3	344	C	1938	[0, 21, 0, 1917]	CT	0.99	UCG→UUG	Ser→Leu
nad3	349	C	1913	[0, 56, 0, 1857]	CT	0.97	CGG→UGG	Arg→Trp
nad4	29	C	2243	[0, 85, 0, 2158]	CT	0.96	UCU→UUU	Ser→Phe
nad4	44	C	2710	[0, 67, 1, 2642]	CT CG	0.98	CCU→CUU	Pro→Leu
nad4	57	C	2717	[0, 1785, 0, 932]	CT	0.34	CCC→CCU	Pro→Pro
nad4	74	C	2838	[1, 211, 0, 2626]	CT CA	0.93	ACU→AUU	Thr→Ile
nad4	77	C	2888	[0, 52, 1, 2835]	CT CG	0.98	CCU→CUU	Pro→Leu
nad4	84	C	2948	[0, 2457, 1, 490]	CT CG	0.17	UUC→UUU	Phe→Phe
nad4	107	C	2653	[0, 65, 0, 2588]	CT	0.98	CCG→CUG	Pro→Leu
nad4	158	C	2995	[2, 97, 1, 2895]	CT CA CG	0.97	CCU→CUU	Pro→Leu
nad4	164	C	2938	[0, 83, 0, 2855]	CT	0.97	CCU→CUU	Pro→Leu
nad4	166	C	3036	[0, 96, 0, 2940]	CT	0.97	CGG→UGG	Arg→Trp
nad4	177	C	3344	[0, 2898, 0, 446]	CT	0.13	UUC→UUU	Phe→Phe
nad4	197	C	3304	[0, 64, 0, 3240]	CT	0.98	UCU→UUU	Ser→Phe
nad4	362	C	3993	[1, 46, 0, 3946]	CT CA	0.99	ACA→AUA	Thr→Ile
nad4	368	C	3940	[0, 53, 1, 3886]	CT CG	0.99	UCG→UUG	Ser→Leu
nad4	376	C	4082	[0, 54, 0, 4028]	CT	0.99	CGU→UGU	Arg→Cys
nad4	403	C	3888	[0, 38, 0, 3850]	CT	0.99	CGC→UGC	Arg→Cys

nad4	433	C	3365	[0, 66, 0, 3299]	CT	0.98	CUU→UUU	Leu→Phe
nad4	436	C	3286	[0, 45, 0, 3241]	CT	0.99	CCC→UUC	Pro→Phe
nad4	437	C	3364	[0, 53, 0, 3311]	CT	0.98	CCC→UUC	Pro→Phe
nad4	449	C	4007	[1, 46, 1, 3959]	CT CA CG	0.99	CCA→CUA	Pro→Leu
nad4	608	C	4860	[0, 26, 3, 4831]	CT CG	0.99	UCA→UUA	Ser→Leu
nad4	659	C	4412	[1, 61, 4, 4346]	CT CG CA	0.99	UCU→UUU	Ser→Phe
nad4	767	C	4799	[0, 41, 2, 4756]	CT CG	0.99	CCU→CUU	Pro→Leu
nad4	770	C	4839	[5, 31, 0, 4803]	CT CA	0.99	UCA→UUA	Ser→Leu
nad4	784	C	4825	[0, 67, 1, 4757]	CT CG	0.99	CAC→UAC	His→Tyr
nad4	836	C	5222	[0, 310, 0, 4912]	CT	0.94	UCC→UUC	Ser→Phe
nad4	857	C	5192	[0, 123, 0, 5069]	CT	0.98	UCA→UUA	Ser→Leu
nad4	977	C	5932	[1, 1578, 8, 4345]	CT CG CA	0.73	CCG→CUG	Pro→Leu
nad4	1006	C	6062	[0, 53, 5, 6004]	CT CG	0.99	CUA→UUA	Leu→Leu
nad4	1010	C	5620	[0, 50, 5, 5565]	CT CG	0.99	CCG→CUG	Pro→Leu
nad4	1033	C	7009	[0, 112, 0, 6897]	CT	0.98	CCU→UCU	Pro→Ser
nad4	1101	C	8604	[0, 346, 2, 8256]	CT CG	0.96	UAC→UAU	Tyr→Tyr
nad4	1129	C	9815	[0, 28, 6, 9781]	CT CG	1	CUC→UUC	Leu→Phe
nad4	1148	C	10,363	[1, 75, 1, 10,286]	CT CA CG	0.99	UCU→UUU	Ser→Phe
nad4	1151	C	10,264	[0, 96, 1, 10,167]	CT CG	0.99	UCU→UUU	Ser→Phe
nad4	1172	C	9529	[0, 99, 4, 9426]	CT CG	0.99	UCA→UUA	Ser→Leu
nad4	1205	C	11,622	[1, 74, 4, 11,543]	CT CG CA	0.99	CCC→CUC	Pro→Leu
nad4	1355	C	7253	[0, 21, 0, 7232]	CT	1	UCA→UUA	Ser→Leu
nad4	1373	C	11,365	[0, 17, 2, 11,346]	CT CG	1	UCC→UUC	Ser→Phe
nad4	1405	C	10,058	[0, 55, 0, 10,003]	CT	0.99	CGG→UGG	Arg→Trp
nad4	1417	C	10,193	[0, 86, 0, 10,107]	CT	0.99	CAC→UAC	His→Tyr
nad4	1433	C	9903	[4, 87, 4, 9808]	CT CA CG	0.99	CCG→CUG	Pro→Leu
nad4L	2	C	4638	[0, 317, 3, 4318]	CT CG	0.93	ACG→AUG	Thr→Met
nad4L	21	C	5197	[0, 4644, 1, 552]	CT CG	0.11	UUC→UUU	Phe→Phe
nad4L	39	C	4653	[1, 3443, 2, 1207]	CT CG CA	0.26	AUC→AUU	Ile→Ile

nad4L	41	C	5036	[0, 40, 4, 4992]	CT CG	0.99	UCU→UUU	Ser→Phe
nad4L	55	C	5465	[0, 90, 1, 5374]	CT CG	0.98	CGG→UGG	Arg→Trp
nad4L	86	C	5927	[0, 44, 1, 5882]	CT CG	0.99	CCU→CUU	Pro→Leu
nad4L	95	C	5326	[0, 78, 0, 5248]	CT	0.99	UCA→UUA	Ser→Leu
nad4L	100	C	5091	[0, 105, 2, 4984]	CT CG	0.98	CCA→UCA	Pro→Ser
nad4L	110	C	4822	[1, 70, 2, 4749]	CT CG CA	0.99	UCA→UUA	Ser→Leu
nad4L	131	C	4458	[0, 80, 0, 4378]	CT	0.98	UCG→UUG	Ser→Leu
nad4L	149	C	5183	[0, 4008, 1, 1174]	CT CG	0.23	UCU→UUU	Ser→Phe
nad4L	158	C	4322	[0, 77, 0, 4245]	CT	0.98	UCG→UUG	Ser→Leu
nad4L	179	C	6837	[1, 108, 0, 6728]	CT CA	0.98	UCA→UUA	Ser→Leu
nad4L	188	C	7024	[0, 328, 0, 6696]	CT	0.95	UCA→UUA	Ser→Leu
nad4L	196	C	6995	[0, 5767, 0, 1228]	CT	0.18	CCA→UUA	Pro→Leu
nad4L	197	C	6815	[0, 1174, 0, 5641]	CT	0.83	CCA→UUA	Pro→Leu
nad5	155	C	3017	[0, 57, 0, 2960]	CT	0.98	CCG→CUG	Pro→Leu
nad5	242	C	3585	[0, 55, 0, 3530]	CT	0.98	CCG→CUG	Pro→Leu
nad5	272	C	3745	[0, 47, 0, 3698]	CT	0.99	UCC→UUC	Ser→Phe
nad5	315	C	3279	[2, 1028, 0, 2249]	CT CA	0.69	UCC→UCU	Ser→Ser
nad5	358	C	3133	[0, 34, 1, 3098]	CT CG	0.99	CUU→UUU	Leu→Phe
nad5	374	C	3234	[0, 36, 1, 3197]	CT CG	0.99	CCA→CUA	Pro→Leu
nad5	398	C	3560	[1, 74, 3, 3482]	CT CG CA	0.98	UCU→UUU	Ser→Phe
nad5	494	C	5412	[0, 29, 4, 5379]	CT CG	0.99	ACA→AUA	Thr→Ile
nad5	539	C	5740	[0, 124, 1, 5615]	CT CG	0.98	CCU→CUU	Pro→Leu
nad5	548	C	5981	[0, 247, 5, 5729]	CT CG	0.96	UCG→UUG	Ser→Leu
nad5	553	C	6008	[0, 174, 2, 5832]	CT CG	0.97	CGU→UGU	Arg→Cys
nad5	598	C	4906	[0, 109, 0, 4797]	CT	0.98	CGU→UGU	Arg→Cys
nad5	608	C	4776	[0, 75, 0, 4701]	CT	0.98	GCC→GUU	Ala→Val
nad5	609	C	4846	[0, 4168, 1, 677]	CT CG	0.14	GCC→GUU	Ala→Val
nad5	629	C	4985	[0, 731, 1, 4253]	CT CG	0.85	UCU→UUU	Ser→Phe
nad5	713	C	4847	[1, 81, 2, 4763]	CT CG CA	0.98	UCG→UUG	Ser→Leu

nad5	725	C	5008	[0, 65, 0, 4943]	CT	0.99	UCA→UUA	Ser→Leu
nad5	764	C	5499	[3, 103, 1, 5392]	CT CA CG	0.98	UCG→UUG	Ser→Leu
nad5	801	C	5378	[0, 4784, 1, 593]	CT CG	0.11	UUC→UUU	Phe→Phe
nad5	835	C	5586	[0, 128, 1, 5457]	CT CG	0.98	CCA→UCA	Pro→Ser
nad5	863	C	4659	[0, 81, 0, 4578]	CT	0.98	UCU→UUU	Ser→Phe
nad5	875	C	5029	[1, 269, 1, 4758]	CT CA CG	0.95	ACG→AUG	Thr→Met
nad5	1310	C	3846	[0, 35, 1, 3810]	CT CG	0.99	UCA→UUA	Ser→Leu
nad5	1318	C	4015	[2, 2643, 1, 1369]	CT CA CG	0.34	CUA→UUA	Leu→Leu
nad5	1490	C	9613	[7, 48, 7, 9551]	CT CA CG	0.99	CCC→CUC	Pro→Leu
nad5	1550	C	8741	[1, 17, 4, 8719]	CT CG CA	1	ACC→AUC	Thr→Ile
nad5	1580	C	8112	[0, 28, 2, 8082]	CT CG	1	UCA→UUA	Ser→Leu
nad5	1589	C	8692	[0, 34, 2, 8656]	CT CG	1	UCU→UUU	Ser→Phe
nad5	1610	C	9278	[2, 22, 2, 9252]	CT CA CG	1	CCC→CUC	Pro→Leu
nad5	1895	C	12,069	[0, 128, 5, 11936]	CT CG	0.99	UCA→UUA	Ser→Leu
nad5	1916	C	8797	[0, 93, 1, 8703]	CT CG	0.99	UCU→UUU	Ser→Phe
nad5	1918	C	8833	[0, 37, 0, 8796]	CT	1	CGU→UGU	Arg→Cys
nad5	1958	C	14,179	[0, 42, 0, 14137]	CT	1	UCG→UUG	Ser→Leu
nad6	26	C	3995	[1, 16, 4, 3974]	CT CG CA	1	CCU→CUU	Pro→Leu
nad6	53	C	5399	[2, 173, 0, 5224]	CT CA	0.97	GCA→GUA	Ala→Val
nad6	88	C	5528	[0, 117, 0, 5411]	CT	0.98	CCC→UUC	Pro→Phe
nad6	89	C	5528	[0, 182, 1, 5345]	CT CG	0.97	CCC→UUC	Pro→Phe
nad6	103	C	5731	[0, 121, 2, 5608]	CT CG	0.98	CGC→UGC	Arg→Cys
nad6	144	C	6638	[0, 5893, 5, 740]	CT CG	0.11	UUC→UUU	Phe→Phe
nad6	159	C	6795	[2, 5853, 2, 938]	CT CA CG	0.14	UUC→UUU	Phe→Phe
nad6	169	C	6829	[2, 86, 2, 6739]	CT CA CG	0.99	CAU→UAU	His→Tyr
nad6	191	C	6753	[1, 91, 0, 6661]	CT CA	0.99	UCA→UUA	Ser→Leu
nad6	446	C	7431	[1, 44, 3, 7383]	CT CG CA	0.99	UCC→UUC	Ser→Phe
nad6	463	C	8226	[0, 63, 0, 8163]	CT	0.99	CCU→UCU	Pro→Ser
nad6	620	C	17	[0, 14, 0, 3]	CT	0.18	ACG→AUG	Thr→Met

nad7	33	C	2923	[0, 2384, 0, 539]	CT	0.18	UUC→UUU	Phe→Phe
nad7	38	C	3162	[1, 95, 0, 3066]	CT CA	0.97	UCG→UUG	Ser→Leu
nad7	45	C	3200	[0, 2564, 0, 636]	CT	0.2	UUC→UUU	Phe→Phe
nad7	77	C	3429	[0, 32, 3, 3394]	CT CG	0.99	UCA→UUA	Ser→Leu
nad7	83	C	3045	[0, 20, 0, 3025]	CT	0.99	UCA→UUA	Ser→Leu
nad7	200	C	3829	[0, 134, 0, 3695]	CT	0.97	UCU→UUU	Ser→Phe
nad7	209	C	3795	[0, 125, 3, 3667]	CT CG	0.97	UCA→UUA	Ser→Leu
nad7	224	C	5064	[0, 37, 1, 5026]	CT CG	0.99	ACG→AUG	Thr→Met
nad7	244	C	5947	[0, 66, 0, 5881]	CT	0.99	CAU→UAU	His→Tyr
nad7	251	C	5908	[0, 78, 2, 5828]	CT CG	0.99	UCA→UUA	Ser→Leu
nad7	315	C	5538	[0, 2966, 2, 2570]	CT CG	0.46	UUC→UUU	Phe→Phe
nad7	316	C	5836	[0, 33, 1, 5802]	CT CG	0.99	CGU→UGU	Arg→Cys
nad7	335	C	6941	[0, 41, 3, 6897]	CT CG	0.99	UCA→UUA	Ser→Leu
nad7	344	C	6995	[0, 77, 3, 6915]	CT CG	0.99	UCA→UUA	Ser→Leu
nad7	383	C	6885	[1, 60, 2, 6822]	CT CG CA	0.99	UCA→UUA	Ser→Leu
nad7	533	C	8705	[0, 127, 1, 8577]	CT CG	0.99	UCC→UUC	Ser→Phe
nad7	578	C	9543	[0, 59, 1, 9483]	CT CG	0.99	UCA→UUA	Ser→Leu
nad7	679	C	11,023	[8, 541, 5, 10,469]	CT CA CG	0.95	CCA→UCA	Pro→Ser
nad7	724	C	13,535	[0, 57, 8, 13,470]	CT CG	1	CAU→UAU	His→Tyr
nad7	734	C	11,784	[1, 29, 5, 11,749]	CT CG CA	1	UCG→UUG	Ser→Leu
nad7	739	C	11,188	[1, 277, 1, 10,909]	CT CA CG	0.98	CCU→UUU	Pro→Phe
nad7	740	C	11,748	[0, 102, 2, 11,644]	CT CG	0.99	CCU→UUU	Pro→Phe
nad7	769	C	13,236	[5, 29, 2, 13,200]	CT CA CG	1	CGC→UGC	Arg→Cys
nad7	789	C	14,240	[1, 4346, 8, 9885]	CT CG CA	0.69	AUC→AUU	Ile→Ile
nad7	795	C	14,597	[2, 5847, 1, 8747]	CT CA CG	0.6	AUC→AUU	Ile→Ile
nad7	836	C	15,717	[0, 30, 4, 15,683]	CT CG	1	CCU→CUU	Pro→Leu
nad7	915	C	13,759	[2, 7420, 4, 6333]	CT CG CA	0.46	UCC→UCU	Ser→Ser
nad7	926	C	14,818	[1, 1068, 1, 13,748]	CT CA CG	0.93	UCA→UUA	Ser→Leu
nad7	944	C	14,445	[1, 122, 7, 14,315]	CT CG CA	0.99	CCU→CUU	Pro→Leu

nad7	963	C	13,156	[4, 11,235, 1, 1916]	CT CA CG	0.15	UCC→UCU	Ser→Ser
nad7	973	C	13,409	[0, 67, 2, 13,340]	CT CG	1	CCU→UCU	Pro→Ser
nad7	1050	C	10,105	[1, 1005, 2, 9097]	CT CG CA	0.9	CCC→CCU	Pro→Pro
nad7	1057	C	10,625	[2, 246, 4, 10,373]	CT CG CA	0.98	CGU→UGU	Arg→Cys
nad7	1079	C	17,580	[2, 105, 1, 17,472]	CT CA CG	0.99	UCU→UUU	Ser→Phe
nad7	1088	C	17,817	[0, 169, 4, 17,644]	CT CG	0.99	UCA→UUA	Ser→Leu
nad7	1103	C	18,823	[0, 65, 0, 18,758]	CT	1	UCU→UUU	Ser→Phe
nad7	1119	C	17,427	[0, 10,520, 1, 6906]	CT CG	0.4	CAC→CAU	His→His
nad7	1124	C	16,068	[2, 162, 11, 15,893]	CT CG CA	0.99	CCA→CUA	Pro→Leu
nad7	1166	C	14,400	[0, 48, 1, 14351]	CT CG	1	UCU→UUU	Ser→Phe
nad9	14	C	2704	[0, 162, 0, 2542]	CT	0.94	UCC→UUC	Ser→Phe
nad9	92	C	4152	[0, 212, 2, 3938]	CT CG	0.95	UCU→UUU	Ser→Phe
nad9	113	C	4592	[1, 130, 3, 4458]	CT CG CA	0.97	CCA→CUA	Pro→Leu
nad9	167	C	7012	[0, 100, 4, 6908]	CT CG	0.99	UCG→UUG	Ser→Leu
nad9	190	C	7120	[0, 574, 2, 6544]	CT CG	0.92	CAU→UAU	His→Tyr
nad9	298	C	7221	[0, 153, 1, 7067]	CT CG	0.98	CCG→UCG	Pro→Ser
nad9	328	C	5602	[0, 84, 4, 5514]	CT CG	0.98	CGG→UGG	Arg→Trp
nad9	368	C	6161	[0, 122, 0, 6039]	CT	0.98	UCC→UUC	Ser→Phe
nad9	398	C	5043	[0, 55, 0, 4988]	CT	0.99	UCA→UUA	Ser→Leu
nad9	439	C	5049	[1, 159, 2, 4887]	CT CG CA	0.97	CUU→UUU	Leu→Phe
rpl10	83	C	4433	[0, 261, 1, 4171]	CT CG	0.94	UCA→UUA	Ser→Leu
rpl10	101	C	4938	[0, 707, 1, 4230]	CT CG	0.86	UCG→UUG	Ser→Leu
rpl10	133	C	5357	[0, 3998, 0, 1359]	CT	0.25	CCA→UUA	Pro→Leu
rpl10	134	C	5409	[0, 208, 4, 5197]	CT CG	0.96	CCA→UUA	Pro→Leu
rpl10	155	C	5248	[1, 486, 2, 4759]	CT CG CA	0.91	CCA→CUA	Pro→Leu
rpl10	239	C	5354	[0, 423, 0, 4931]	CT	0.92	UCG→UUG	Ser→Leu
rpl10	314	C	5605	[0, 661, 0, 4944]	CT	0.88	UCA→UUA	Ser→Leu
rpl10	330	C	4597	[1, 4023, 1, 572]	CT CA CG	0.12	UAC→UAU	Tyr→Tyr
rpl10	397	C	5783	[0, 5154, 0, 629]	CT	0.11	CUU→UUU	Leu→Phe

rpl16	104	C	4734	[0, 99, 0, 4635]	CT	0.98	ACU→AUU	Thr→Ile
rpl16	164	C	5254	[1, 376, 3, 4874]	CT CG CA	0.93	ACA→AUA	Thr→Ile
rpl16	232	C	9287	[0, 172, 2, 9113]	CT CG	0.98	CUC→UUC	Leu→Phe
rpl16	321	C	9044	[1, 5938, 1, 3104]	CT CA CG	0.34	UCC→UCU	Ser→Ser
rpl16	401	C	7704	[0, 43, 3, 7658]	CT CG	0.99	CCA→CUA	Pro→Leu
rpl16	407	C	8352	[0, 62, 0, 8290]	CT	0.99	UCG→UUG	Ser→Leu
rpl2	215	C	5576	[0, 250, 0, 5326]	CT	0.96	CCA→CUA	Pro→Leu
rpl2	984	C	4092	[0, 3626, 2, 464]	CT CG	0.11	CCC→CCU	Pro→Pro
rpl5	7	C	2327	[0, 474, 0, 1853]	CT	0.8	CCG→UCG	Pro→Ser
rpl5	19	C	2305	[0, 1425, 1, 879]	CT CG	0.38	CAU→UAU	His→Tyr
rpl5	35	C	2594	[0, 1563, 1, 1030]	CT CG	0.4	UCA→UUA	Ser→Leu
rpl5	47	C	3062	[0, 319, 1, 2742]	CT CG	0.9	CCG→CUG	Pro→Leu
rpl5	59	C	3615	[0, 166, 1, 3448]	CT CG	0.95	CCG→CUG	Pro→Leu
rpl5	64	C	3713	[1, 128, 2, 3582]	CT CG CA	0.97	CAC→UAC	His→Tyr
rpl5	92	C	3953	[0, 254, 4, 3695]	CT CG	0.94	UCG→UUG	Ser→Leu
rpl5	169	C	4628	[0, 895, 1, 3732]	CT CG	0.81	CCG→UUG	Pro→Leu
rpl5	170	C	4776	[0, 209, 1, 4566]	CT CG	0.96	CCG→UUG	Pro→Leu
rpl5	320	C	4231	[2, 216, 2, 4011]	CT CA CG	0.95	UCG→UUG	Ser→Leu
rpl5	332	C	4179	[0, 3167, 0, 1012]	CT	0.24	UCG→UUG	Ser→Leu
rpl5	346	C	4068	[1, 2287, 0, 1780]	CT CA	0.44	CUG→UUG	Leu→Leu
rpl5	420	C	4623	[2, 4163, 0, 458]	CT CA	0.1	UCC→UCU	Ser→Ser
rpl5	515	C	3458	[0, 398, 0, 3060]	CT	0.88	CCA→CUA	Pro→Leu
rpl5	518	C	3359	[0, 212, 0, 3147]	CT	0.94	CCG→CUG	Pro→Leu
rps1	23	C	148	[0, 24, 0, 124]	CT	0.84	UCA→UUA	Ser→Leu
rps1	51	C	170	[0, 26, 0, 144]	CT	0.85	UUC→UUU	Phe→Phe
rps1	158	C	462	[0, 19, 1, 442]	CT CG	0.96	CCU→CUU	Pro→Leu
rps1	209	C	582	[0, 55, 0, 527]	CT	0.91	UCC→UUC	Ser→Phe
rps1	581	C	1119	[2, 887, 0, 230]	CT CA	0.21	CCA→CUA	Pro→Leu
rps10	7	C	4371	[0, 151, 3, 4217]	CT CG	0.97	CUG→UUG	Leu→Leu

rps10	12	C	4293	[0, 2293, 3, 1997]	CT CG	0.47	AUC→AUU	Ile→Ile
rps10	62	C	4679	[0, 387, 2, 4290]	CT CG	0.92	ACG→AUG	Thr→Met
rps10	76	C	4727	[0, 834, 2, 3891]	CT CG	0.82	CGU→UGU	Arg→Cys
rps10	294	C	3803	[0, 3362, 0, 441]	CT	0.12	UUC→UUU	Phe→Phe
rps10	298	C	4516	[0, 61, 0, 4455]	CT	0.99	CGG→UGG	Arg→Trp
rps10	362	C	1856	[0, 31, 5, 1820]	CT CG	0.98	UCG→UUG	Ser→Leu
rps12	100	C	718	[0, 137, 0, 581]	CT	0.81	CGC→UGC	Arg→Cys
rps12	104	C	726	[0, 119, 0, 607]	CT	0.84	CCG→CUG	Pro→Leu
rps12	196	C	541	[0, 52, 0, 489]	CT	0.9	CAC→UAC	His→Tyr
rps12	221	C	696	[0, 195, 0, 501]	CT	0.72	UCG→UUG	Ser→Leu
rps12	269	C	776	[0, 65, 0, 711]	CT	0.92	UCG→UUG	Ser→Leu
rps12	284	C	756	[0, 90, 0, 666]	CT	0.88	UCC→UUC	Ser→Phe
rps19	7	C	1118	[0, 962, 155, 1]	CG CT	0.14	CGA→UGA	Arg→End
rps19	116	C	810	[0, 76, 0, 734]	CT	0.91	UCG→UUG	Ser→Leu
rps19	126	C	845	[0, 287, 0, 558]	CT	0.66	UUC→UUU	Phe→Phe
rps19	163	C	932	[0, 126, 0, 806]	CT	0.86	CCU→UUU	Pro→Phe
rps19	164	C	904	[97, 56, 0, 751]	CT CA	0.93	CCU→UUU	Pro→Phe
rps19	180	C	962	[0, 547, 1, 414]	CT CG	0.43	AUC→AUU	Ile→Ile
rps19	260	C	748	[0, 642, 0, 106]	CT	0.14	CCG→CUG	Pro→Leu
rps3	64	C	450	[0, 3, 0, 447]	CT	0.99	CGG→UGG	Arg→Trp
rps3	69	C	459	[0, 391, 0, 68]	CT	0.15	UUC→UUU	Phe→Phe
rps3	92	C	626	[0, 24, 0, 602]	CT	0.96	UCA→UUA	Ser→Leu
rps3	126	C	689	[0, 298, 0, 391]	CT	0.57	UUC→UUU	Phe→Phe
rps3	255	C	857	[0, 738, 0, 119]	CT	0.14	CGC→CGU	Arg→Arg
rps3	512	C	1546	[0, 99, 0, 1447]	CT	0.94	UCA→UUA	Ser→Leu
rps3	642	C	2326	[0, 2011, 0, 315]	CT	0.14	UCC→UCU	Ser→Ser
rps3	713	C	1989	[0, 69, 0, 1920]	CT	0.97	UCG→UUG	Ser→Leu
rps3	890	C	2114	[0, 231, 0, 1883]	CT	0.89	UCG→UUG	Ser→Leu
rps3	1031	C	1210	[0, 26, 0, 1184]	CT	0.98	CCA→CUA	Pro→Leu

rps3	1332	C	2042	[0, 748, 2, 1292]	CT CG	0.63	GUC→GUU	Val→Val
rps3	1364	C	2017	[0, 20, 0, 1997]	CT	0.99	CCG→CUG	Pro→Leu
rps3	1502	C	1784	[0, 90, 0, 1694]	CT	0.95	UCA→UUA	Ser→Leu
rps3	1555	C	1744	[0, 40, 0, 1704]	CT	0.98	CGU→UGU	Arg→Cys
rps3	1588	C	1956	[0, 25, 0, 1931]	CT	0.99	CCU→UCU	Pro→Ser
rps3	1619	C	1976	[0, 45, 1, 1930]	CT CG	0.98	UCA→UUA	Ser→Leu
rps3	1626	C	1917	[1, 1513, 0, 403]	CT CA	0.21	GUC→GUU	Val→Val
rps4	61	C	3241	[0, 77, 1, 3163]	CT CG	0.98	CGG→UGG	Arg→Trp
rps4	145	C	3778	[0, 80, 2, 3696]	CT CG	0.98	CCG→UCG	Pro→Ser
rps4	176	C	2974	[0, 100, 0, 2874]	CT	0.97	UCA→UUA	Ser→Leu
rps4	205	C	3023	[0, 116, 0, 2907]	CT	0.96	CAU→UAU	His→Tyr
rps4	273	C	3300	[0, 2953, 1, 346]	CT CG	0.1	CUC→CUU	Leu→Leu
rps4	278	C	3207	[0, 60, 1, 3146]	CT CG	0.98	CCA→CUA	Pro→Leu
rps4	302	C	3875	[0, 49, 0, 3826]	CT	0.99	CCG→CUG	Pro→Leu
rps4	319	C	3962	[0, 88, 0, 3874]	CT	0.98	CGU→UGU	Arg→Cys
rps4	347	C	3949	[3, 80, 4, 3862]	CT CG CA	0.98	CCG→CUG	Pro→Leu
rps4	476	C	3760	[0, 98, 0, 3662]	CT	0.97	UCU→UUU	Ser→Phe
rps4	494	C	3612	[0, 45, 0, 3567]	CT	0.99	UCA→UUA	Ser→Leu
rps4	681	C	3543	[1, 2826, 0, 716]	CT CA	0.2	UCC→UCU	Ser→Ser
rps4	747	C	3317	[0, 1953, 2, 1362]	CT CG	0.41	UUC→UUU	Phe→Phe
rps4	852	C	3761	[2, 2936, 2, 821]	CT CA CG	0.22	ACC→ACU	Thr→Thr
rps4	906	C	3381	[0, 1321, 3, 2057]	CT CG	0.61	AUC→AUU	Ile→Ile
rps4	926	C	3301	[0, 166, 0, 3135]	CT	0.95	UCG→UUG	Ser→Leu
rps4	937	C	3184	[0, 64, 1, 3119]	CT CG	0.98	CAU→UAU	His→Tyr
rps4	1022	C	2336	[0, 24, 3, 2309]	CT CG	0.99	CCU→CUU	Pro→Leu
rps4	1027	C	2290	[0, 68, 0, 2222]	CT	0.97	CGG→UGG	Arg→Trp
sdh4	29	C	4564	[0, 18, 1, 4545]	CT CG	1	UCG→UUG	Ser→Leu

Figure S1 The validation of large repeat units (DRS02-05). The base sequence above represents the expected sequences in the mitochondrial genome. The base sequence below represents the Sanger sequencing result, and the bottom represents the chromatogram of Sanger sequencing. The black vertical line represents the boundary of repeating sequence.

A. The boundary regions: DRS02-c1

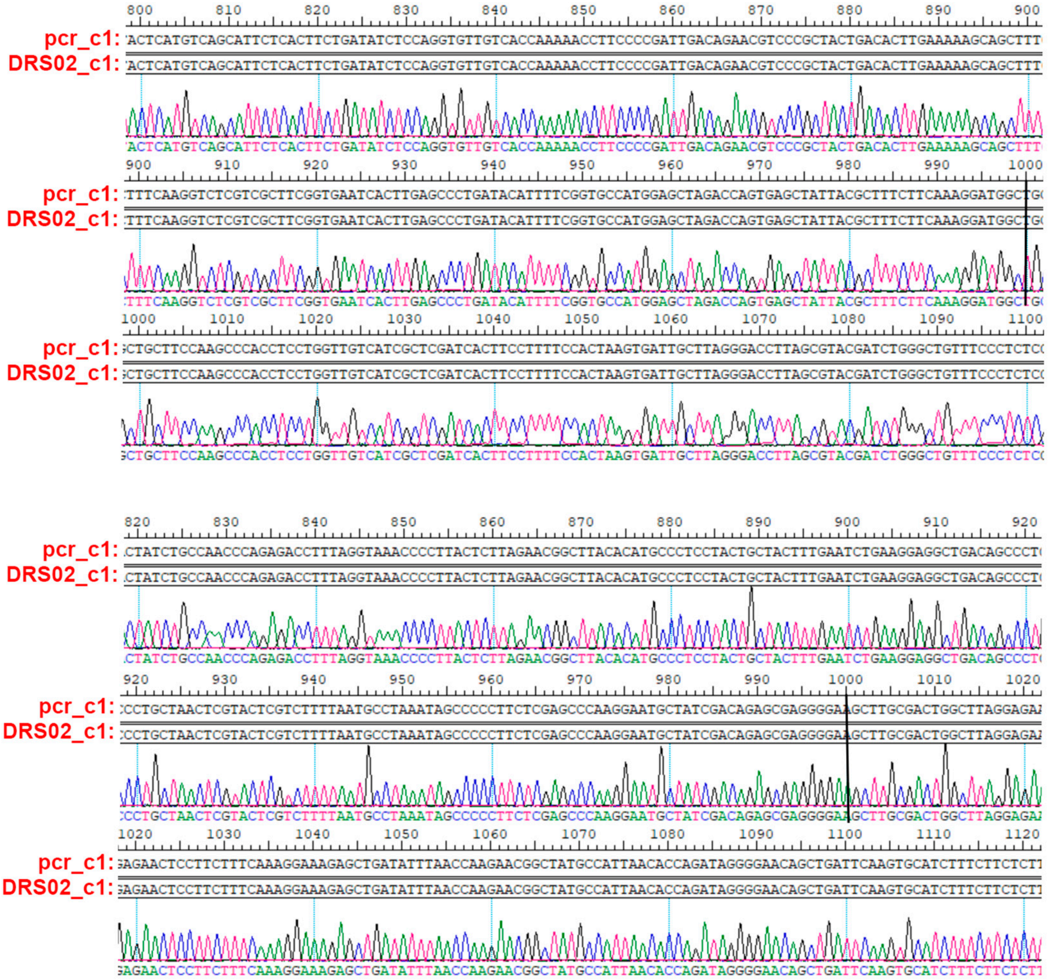

B. The boundary regions: DRS02-c2

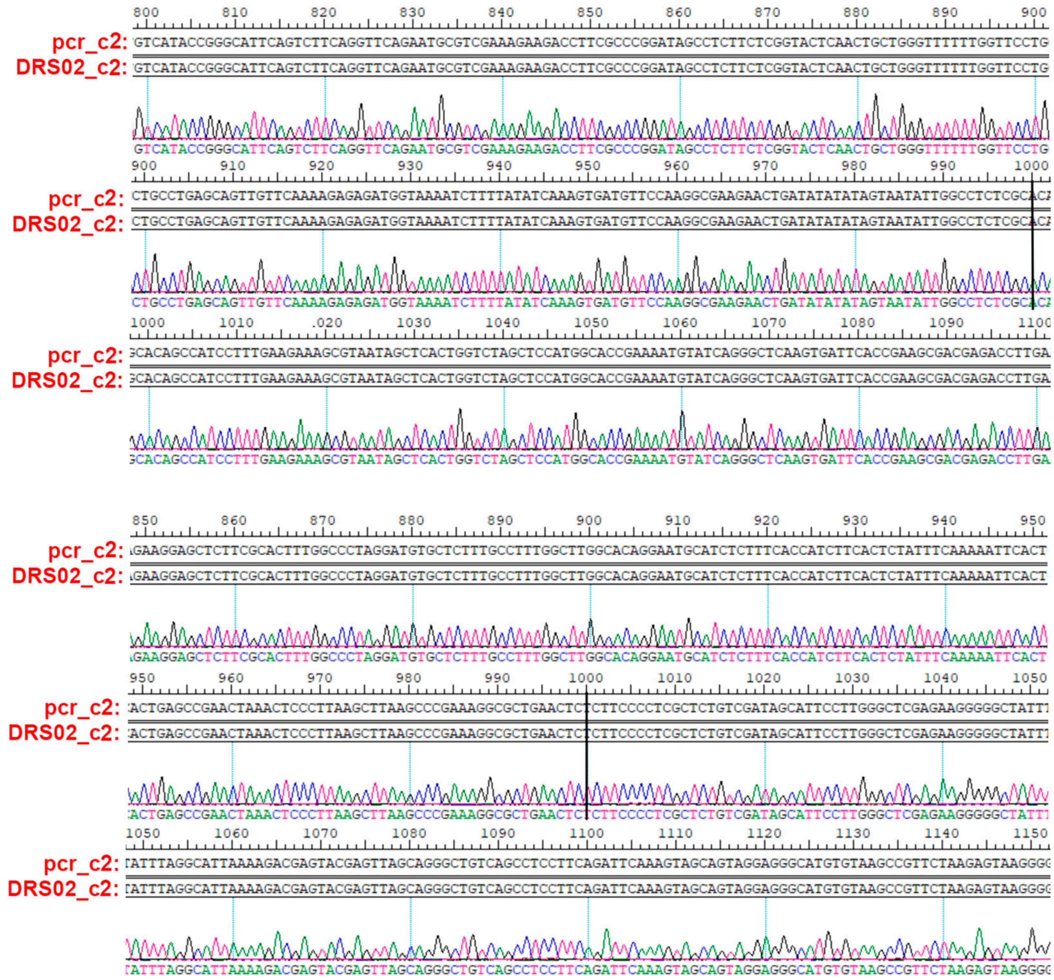

C. The boundary regions: DRS03-c1

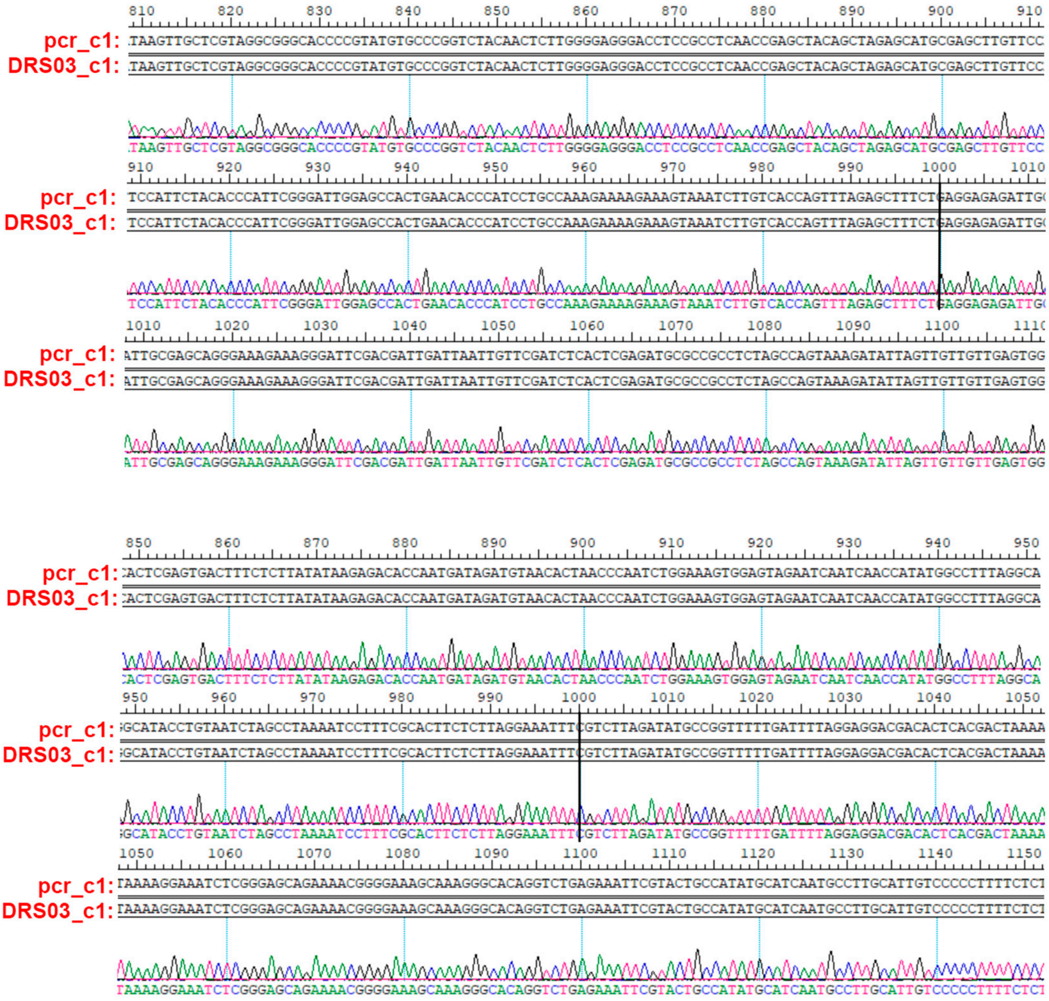

D. The boundary regions: DRS03-c2

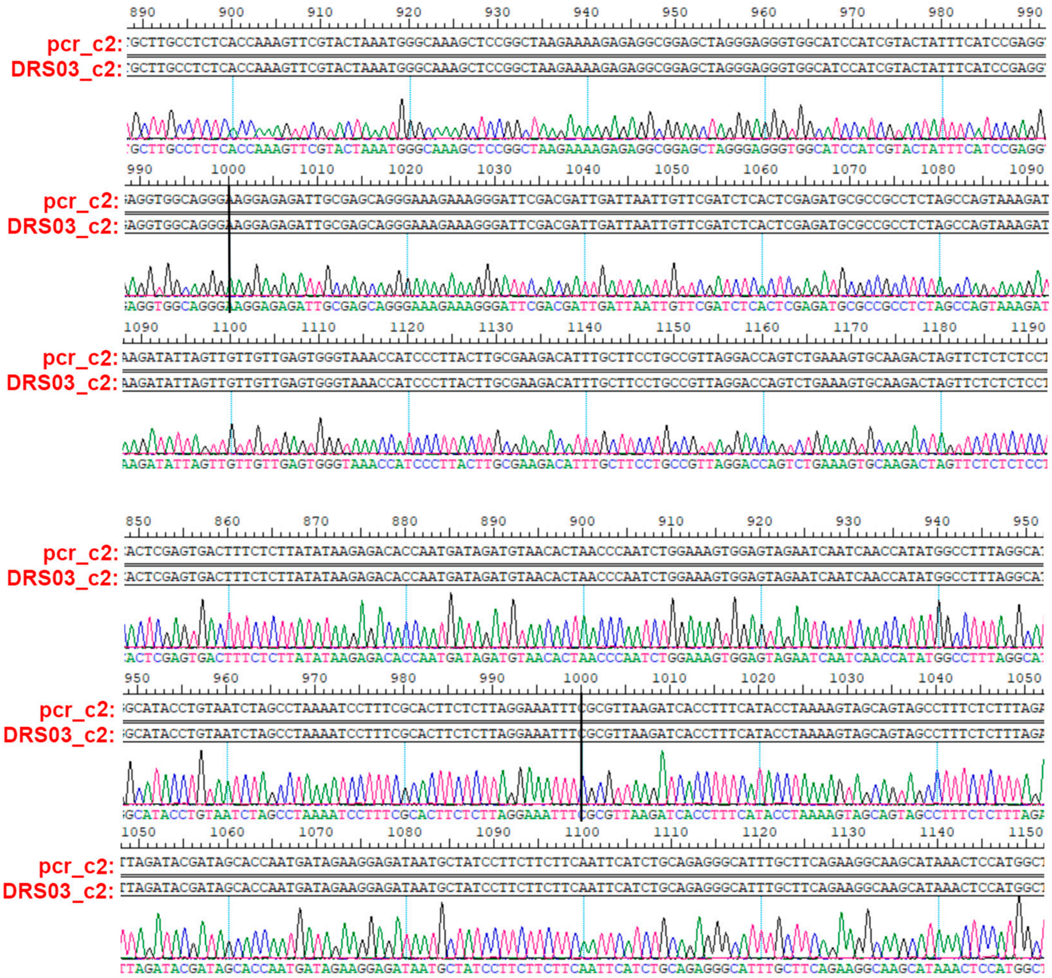

E. The boundary regions: DRS04-c1

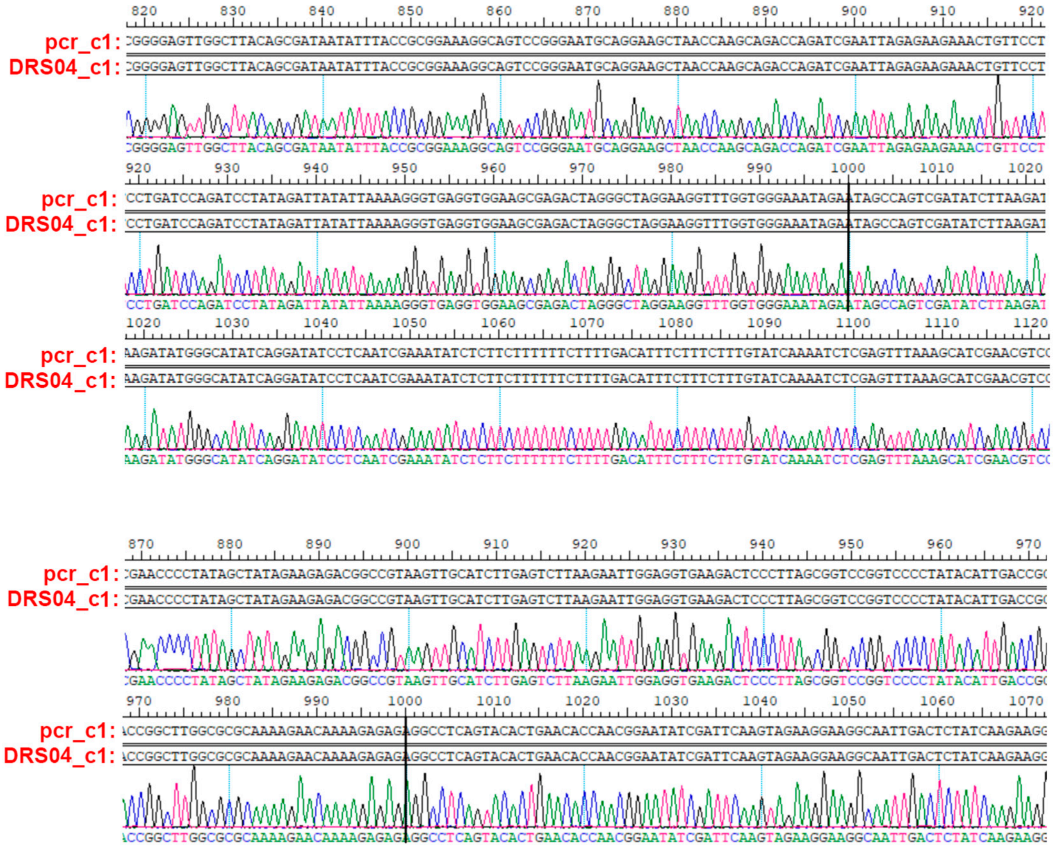

F. The boundary regions: DRS04-c2

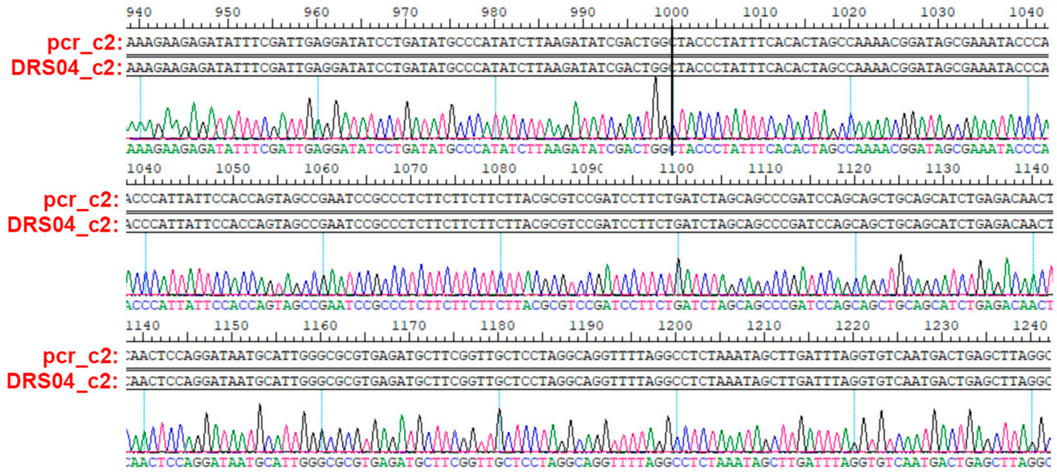

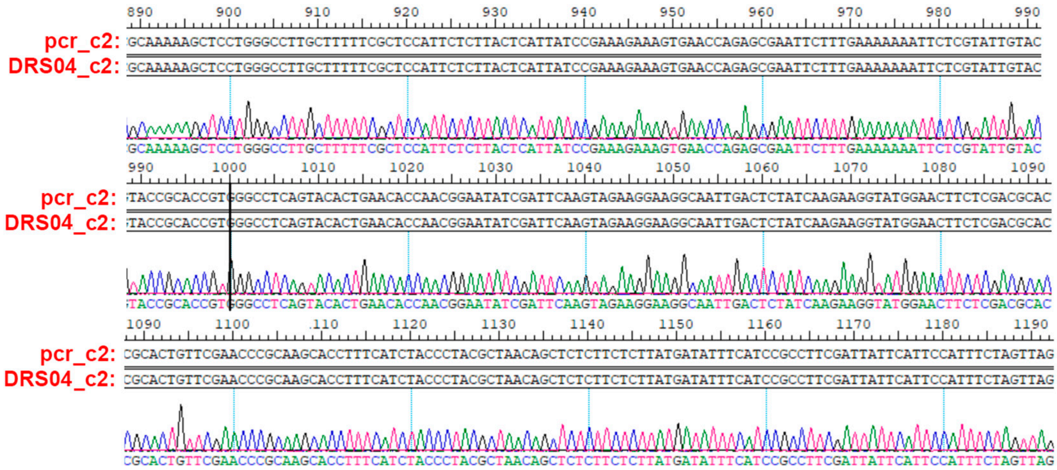

G. The boundary regions: DRS05-c1

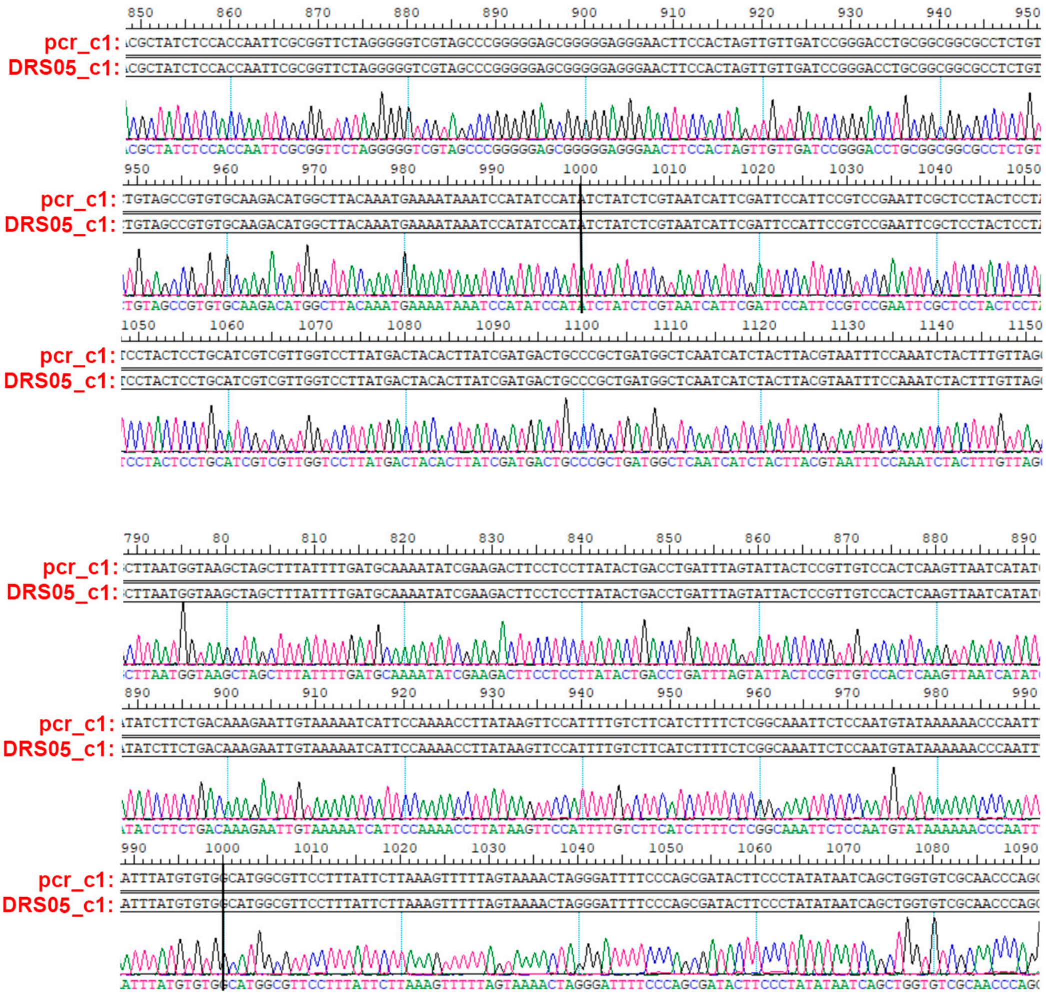

H. The boundary regions: DRS05-c2

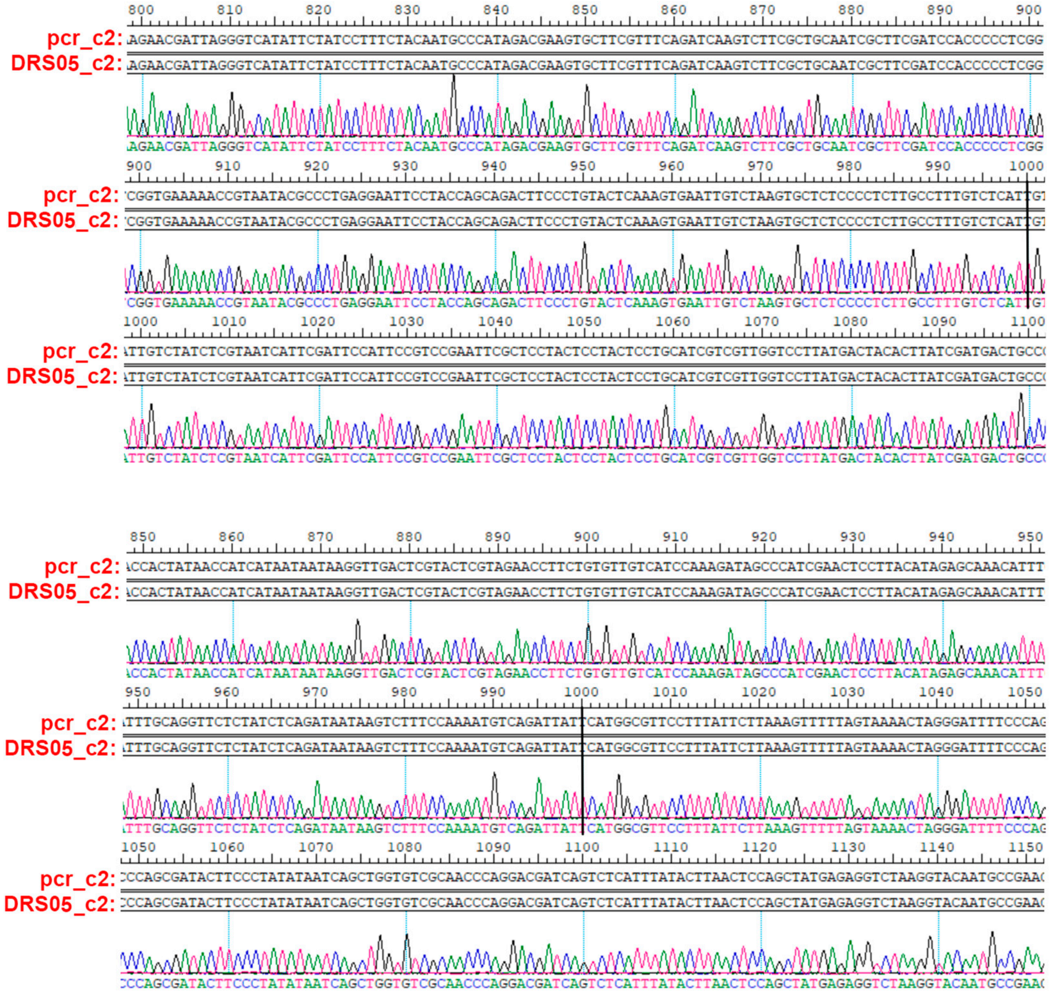

Figure S2 Alignment of the Nanopore long reads to the MTPTs and their flanking sequences in the mitogenome of T. wilfordii. The MTPT sequence was shown on the top of each figure with its coordinates. The reads were represented with gray lines. The regions in gray represent a match. The purple regions represent small indels. The MTPT sequences are indicated with red lines that have arrowheads at each end. The 1000 bp long flanking sequence refers to both ends of the MTPT1.

A. alignment of Nanopore long reads to MTPT1 and its flanking sequences.

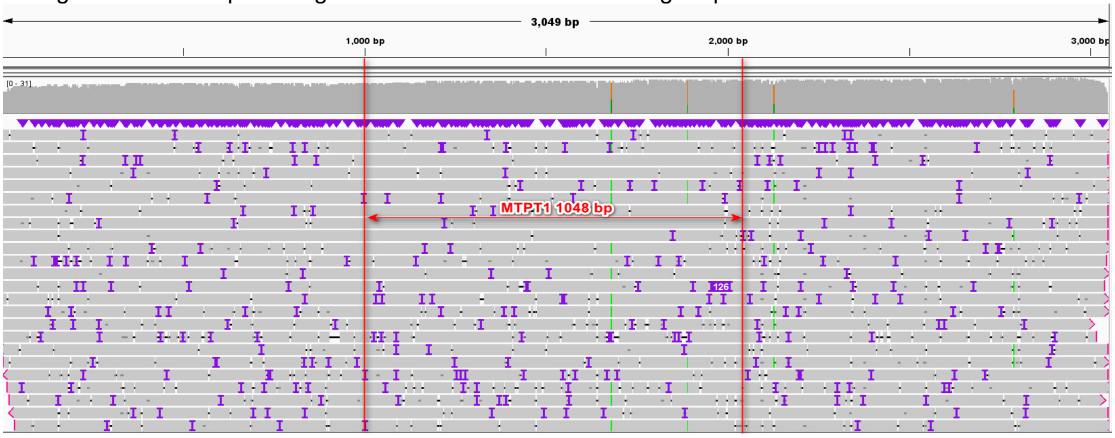

B. alignment of Nanopore long reads to MTPT2 and its flanking sequences.

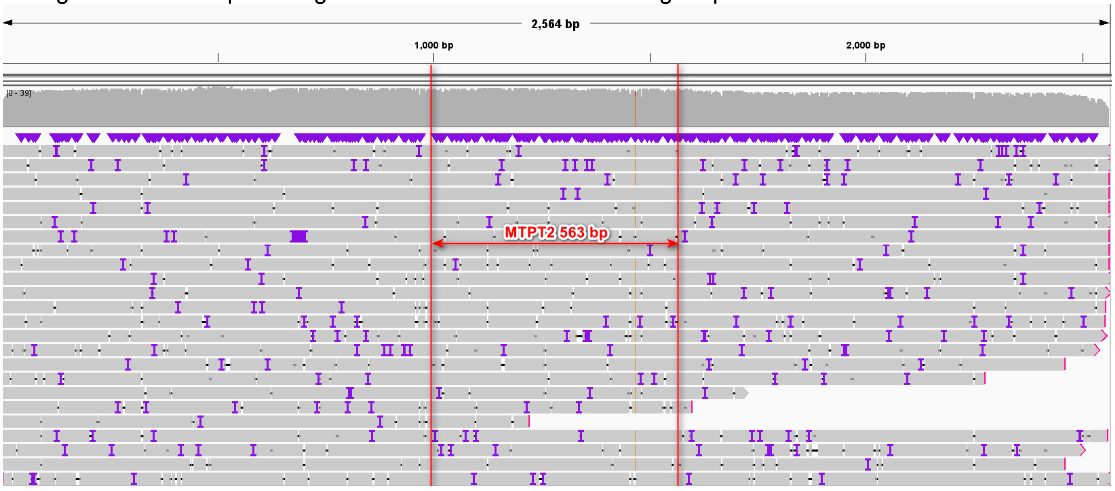

C. alignment of Nanopore long reads to MTPT3 and its flanking sequences.

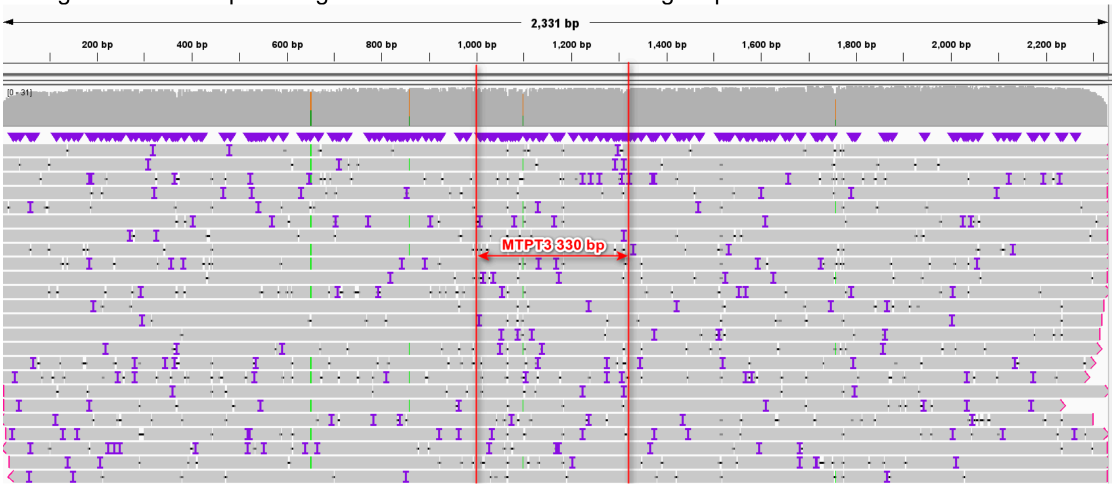

D. alignment of Nanopore long reads to MTPT4 and its flanking sequences.

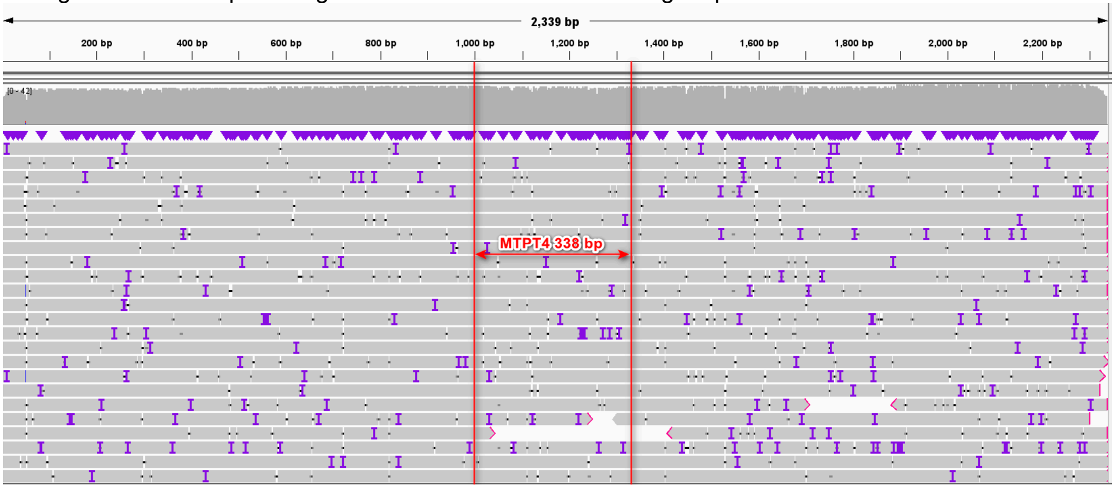

E. alignment of Nanopore long reads to MTPT5 and its flanking sequences.

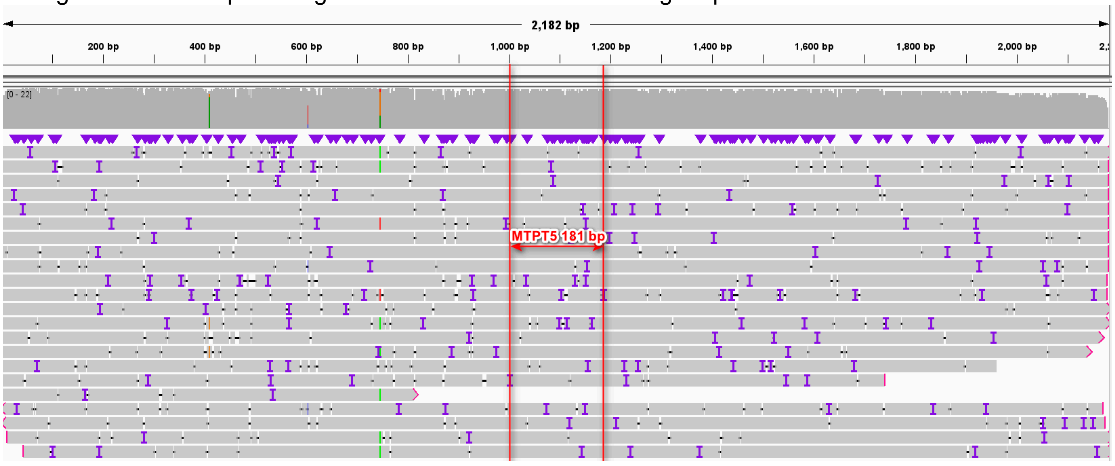

F. alignment of Nanopore long reads to MTPT6 and its flanking sequences.

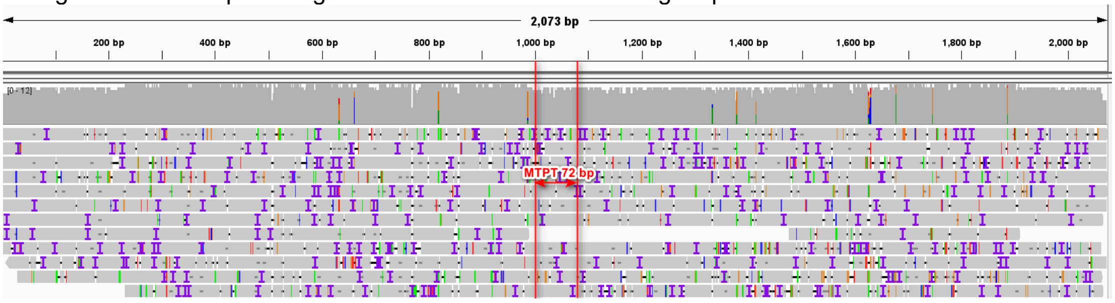

G. alignment of Nanopore long reads to MTPT6 and its flanking sequences.

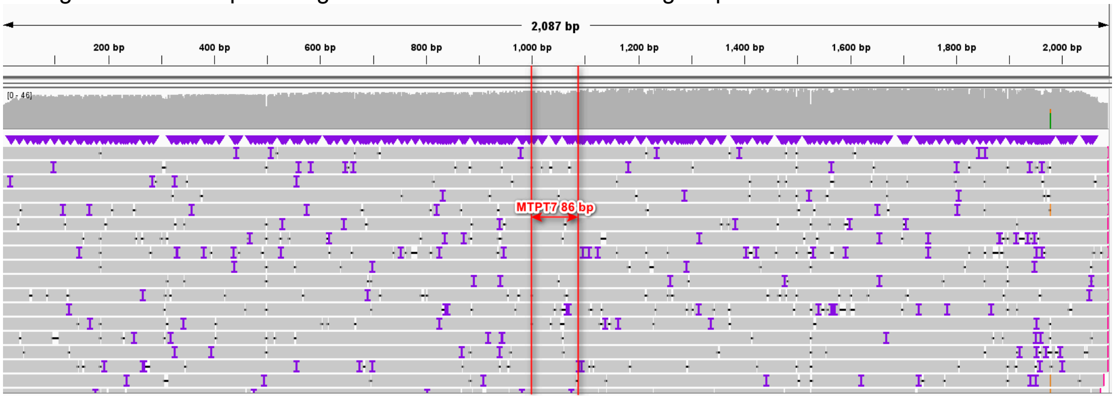

H. alignment of Nanopore long reads to MTPT6 and its flanking sequences.

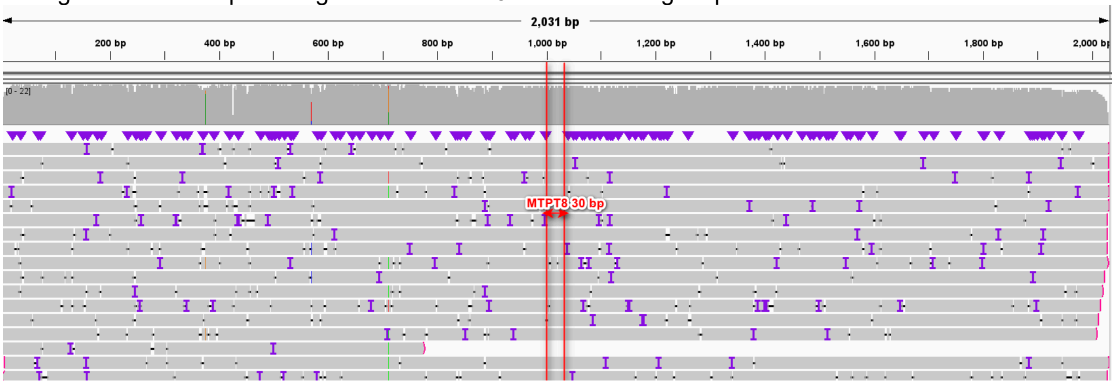

Figure S3 Mapping of the RNA sequencing reads to the coding sequences of PCGs in *T. wilfordii* mitogenome. The reference sequences are shown at the bottom. The gray and color lines above represent the reads mapped to the reference sequence. Bases not matching those in the reference sequences are shown in green for “A”, brown for “G”, blue for “C”, and red for “T”. The RNA editing sites are shown in the red squares.

A. Mapping of RNA-seq reads to the coding sequence of *atp1*.

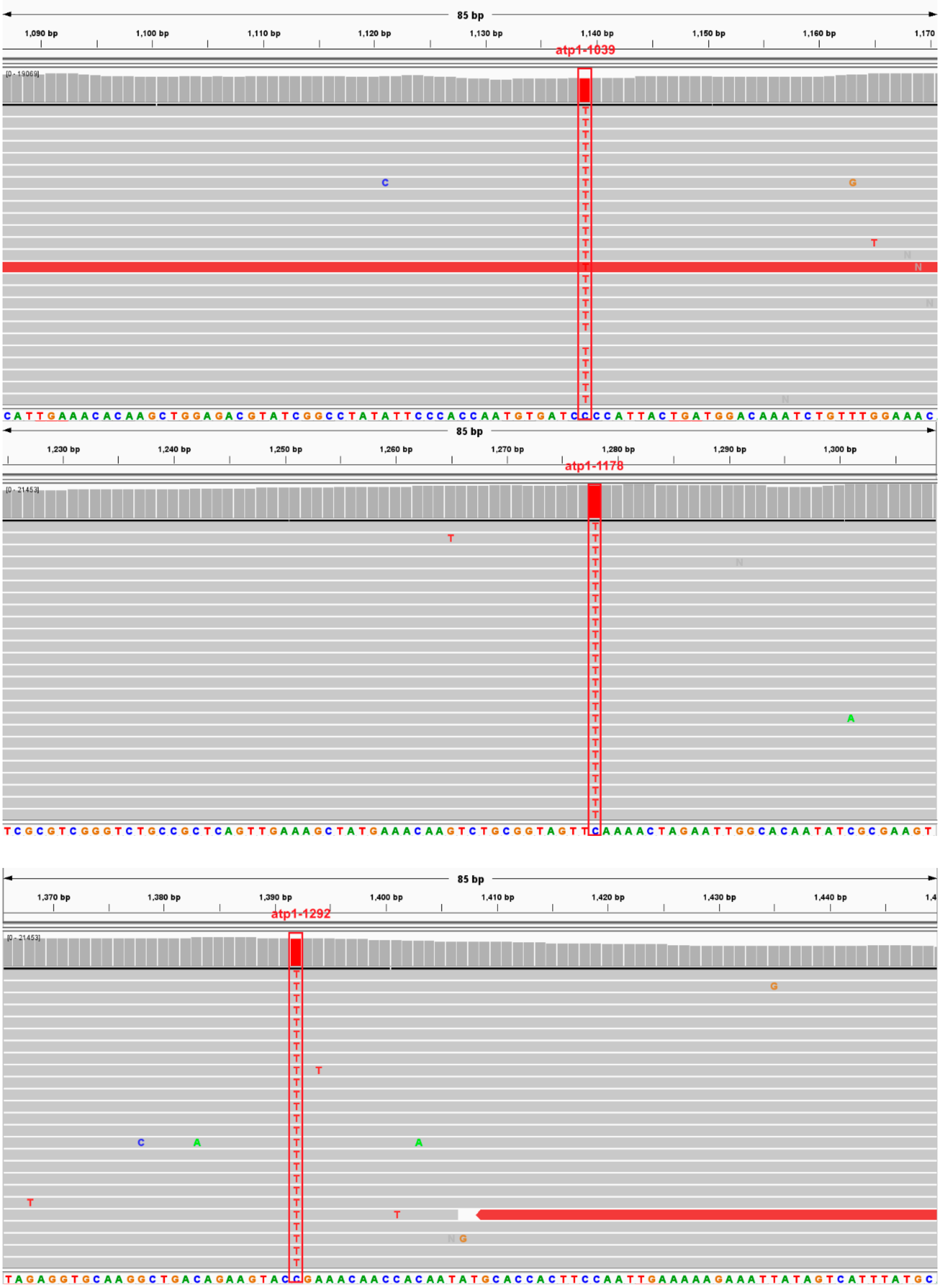

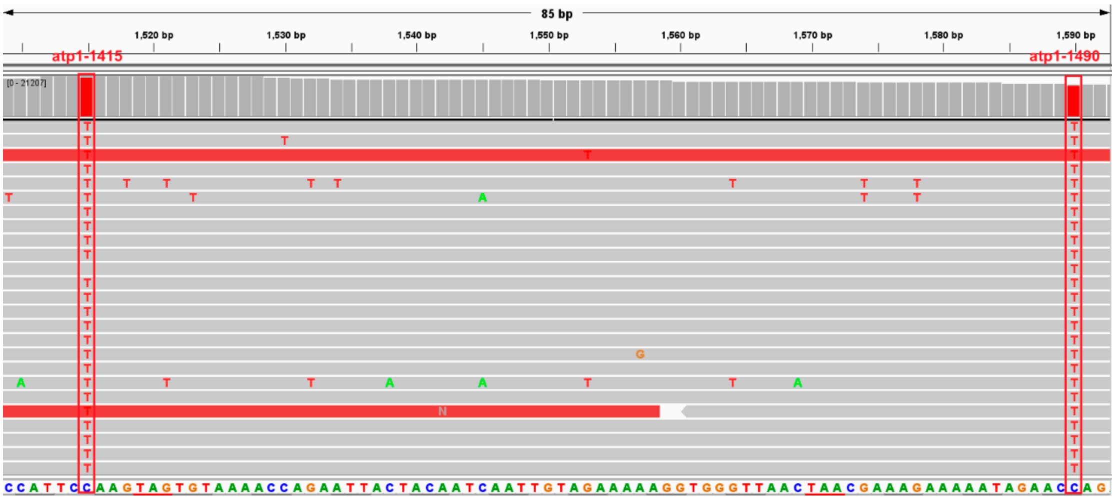

B. Mapping of RNA-seq reads to the coding sequence of *atp4*.

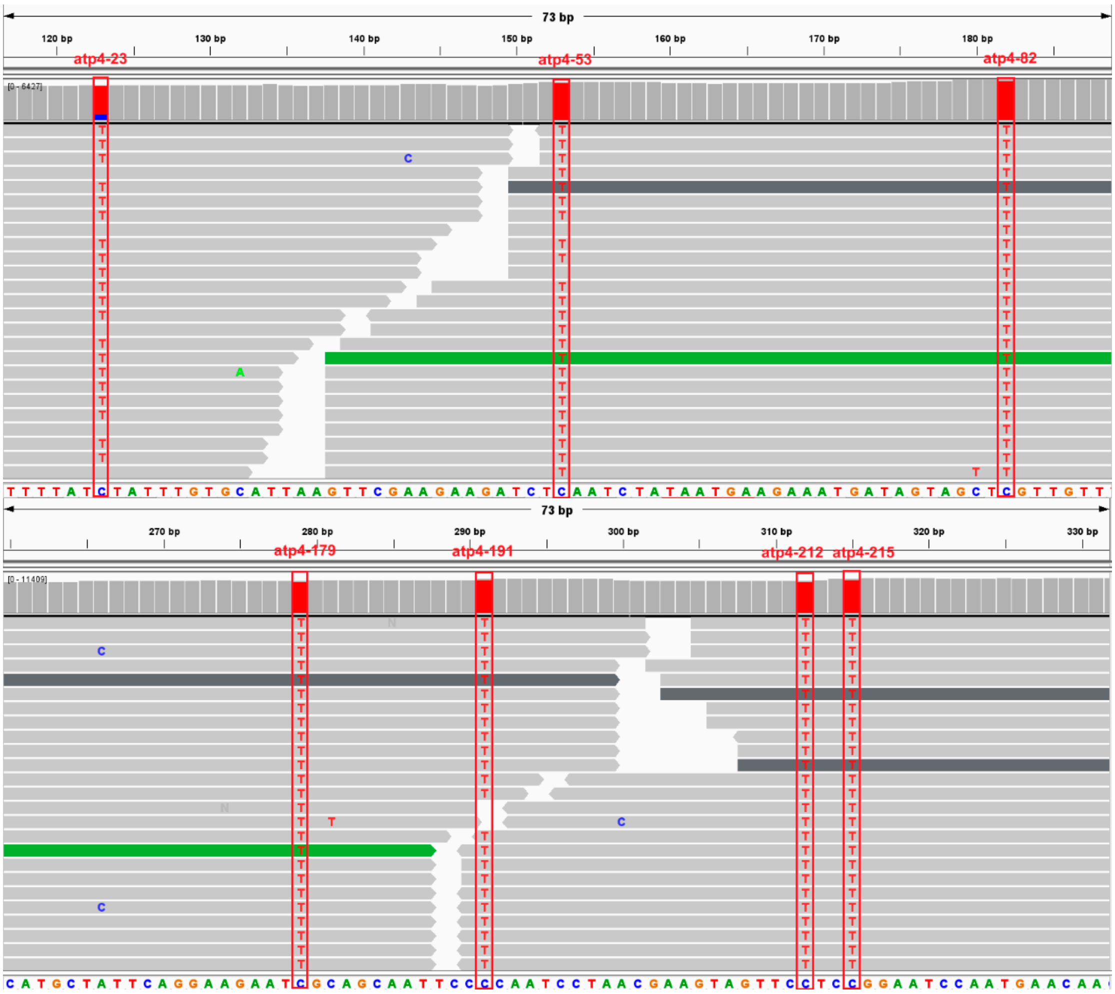

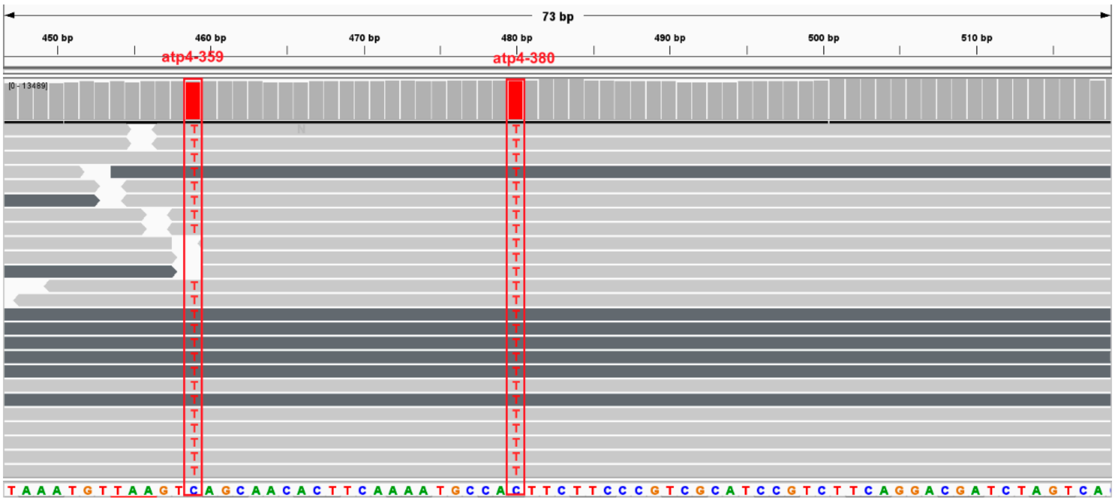

C. Mapping of RNA-seq reads to the coding sequence of *atp6*.

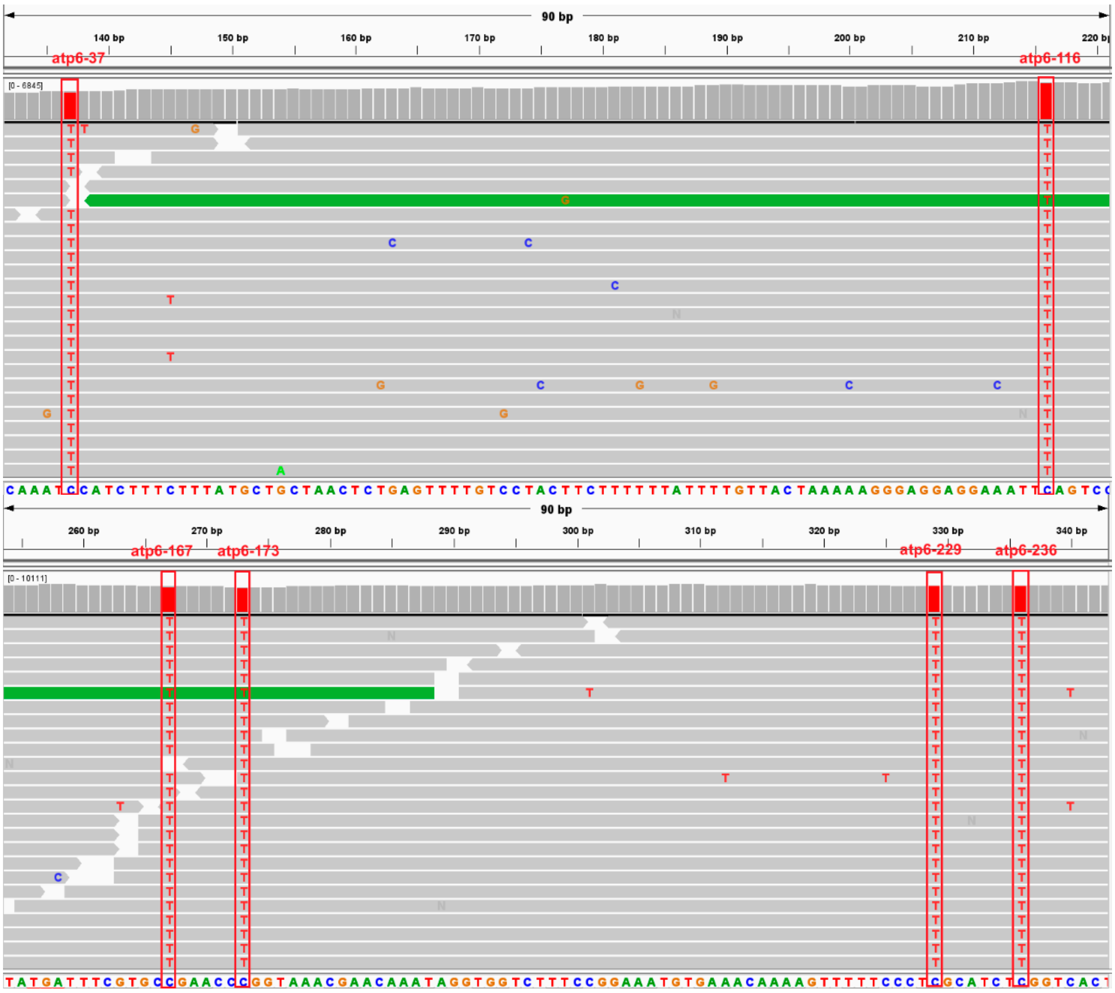

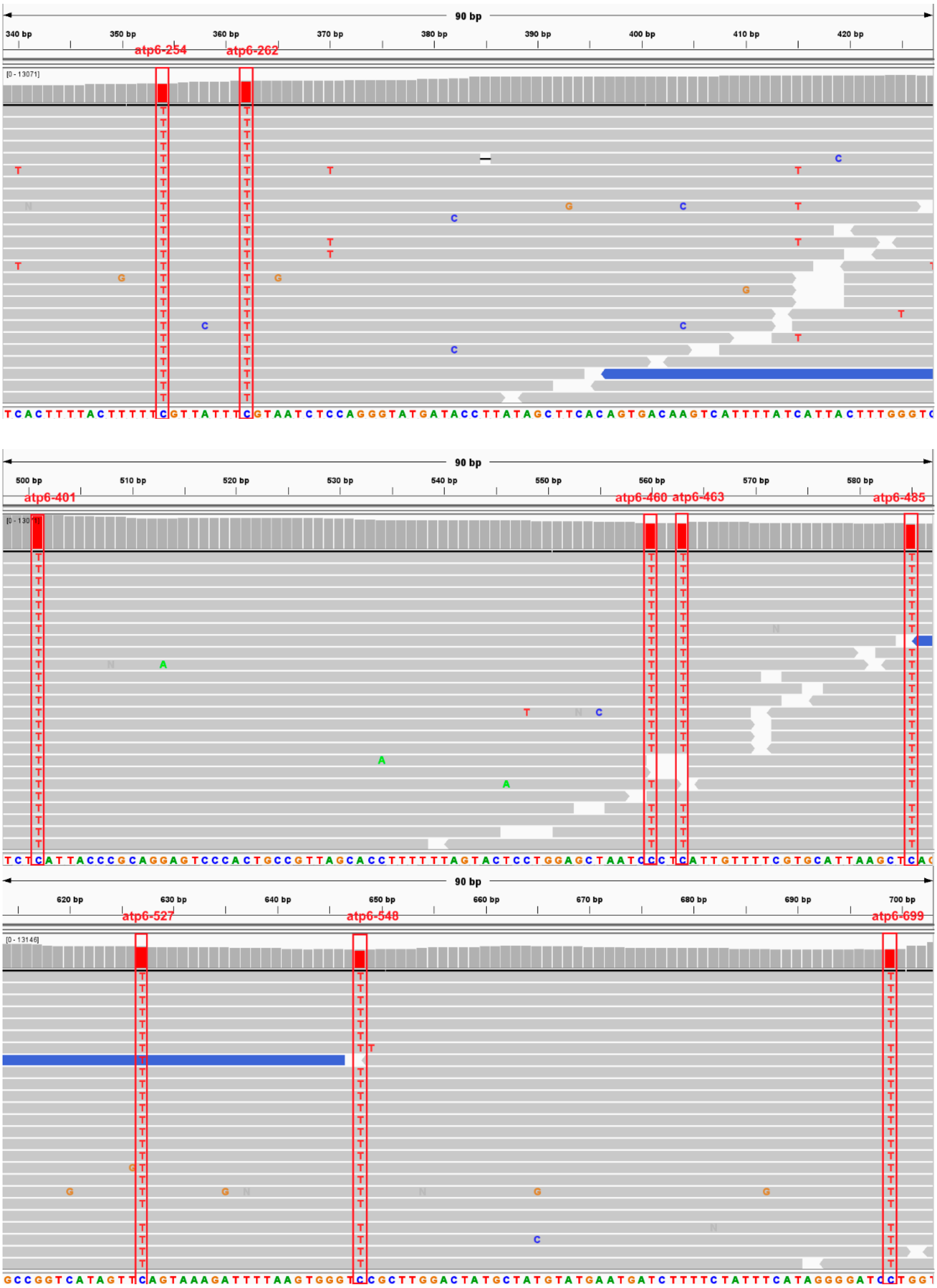
90 bp
340 bp
350 bp
360 bp
370 bp
380 bp
390 bp
400 bp
410 bp
420 bp
atp6-254
atp6-262
[0 - 13071]
90 bp
500 bp
510 bp
520 bp
530 bp
540 bp
550 bp
560 bp
570 bp
580 bp
atp6-401
atp6-460
atp6-463
atp6-485
90 bp
620 bp
630 bp
640 bp
650 bp
660 bp
670 bp
680 bp
690 bp
700 bp
atp6-527
atp6-548
atp6-699
[0 - 13146]

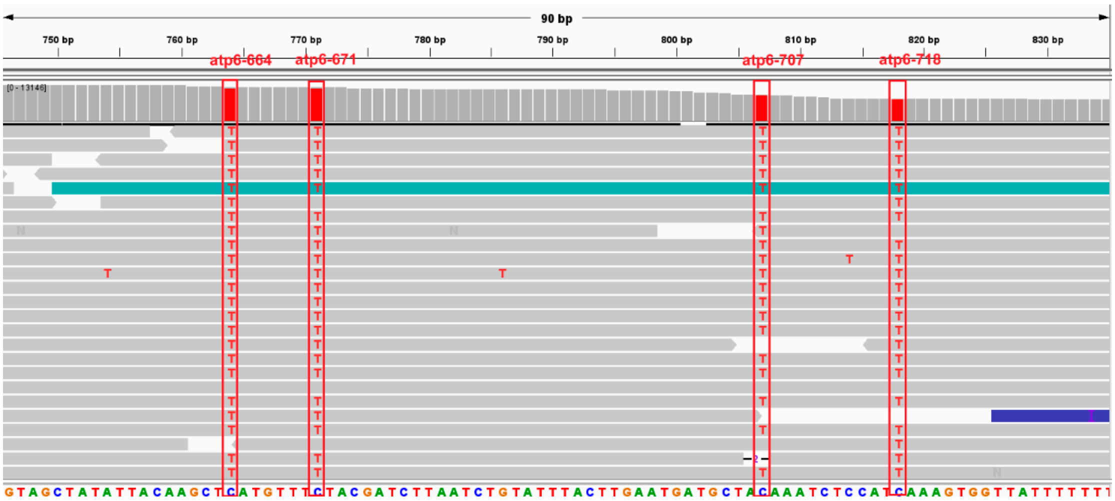

D. Mapping of RNA-seq reads to the coding sequence of *atp8*.

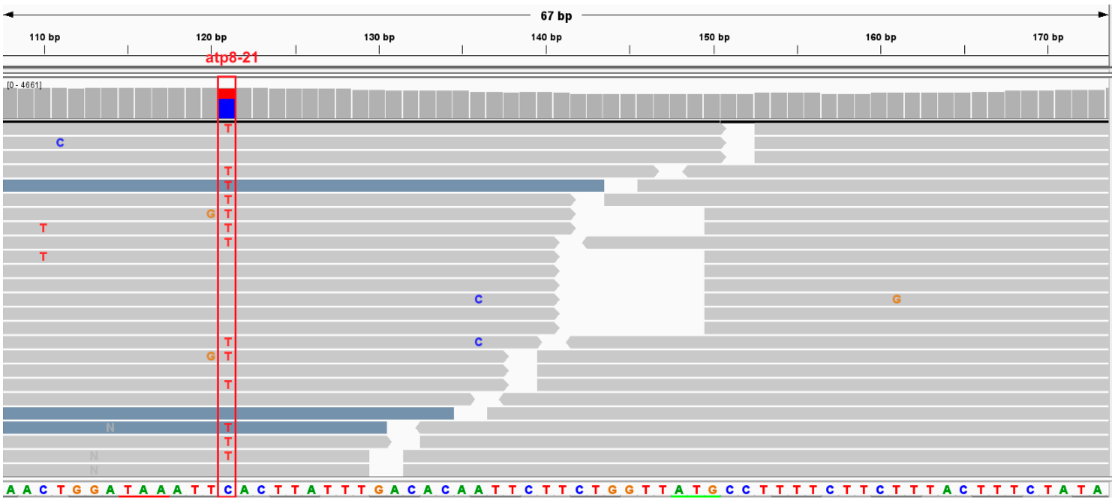

E. Mapping of RNA-seq reads to the coding sequence of *atp9*.

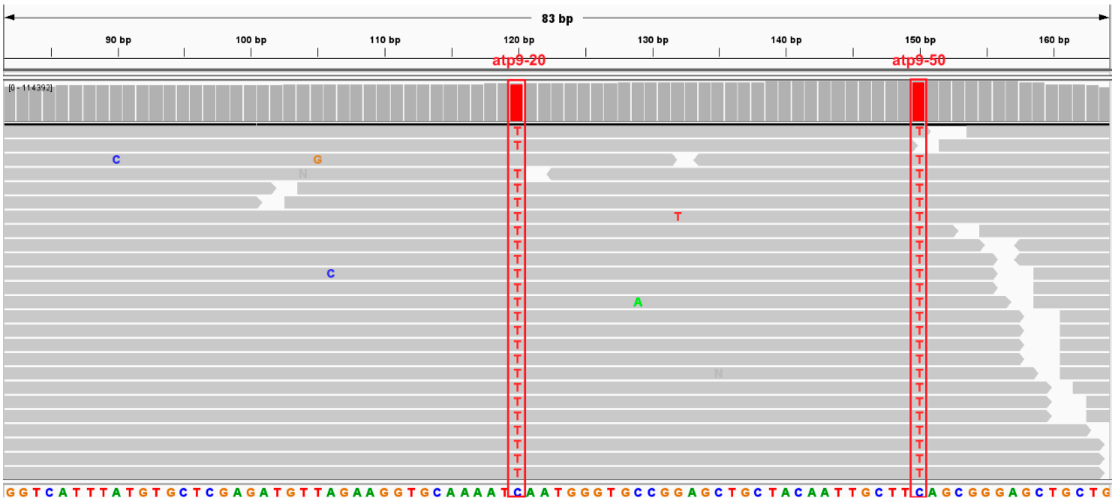

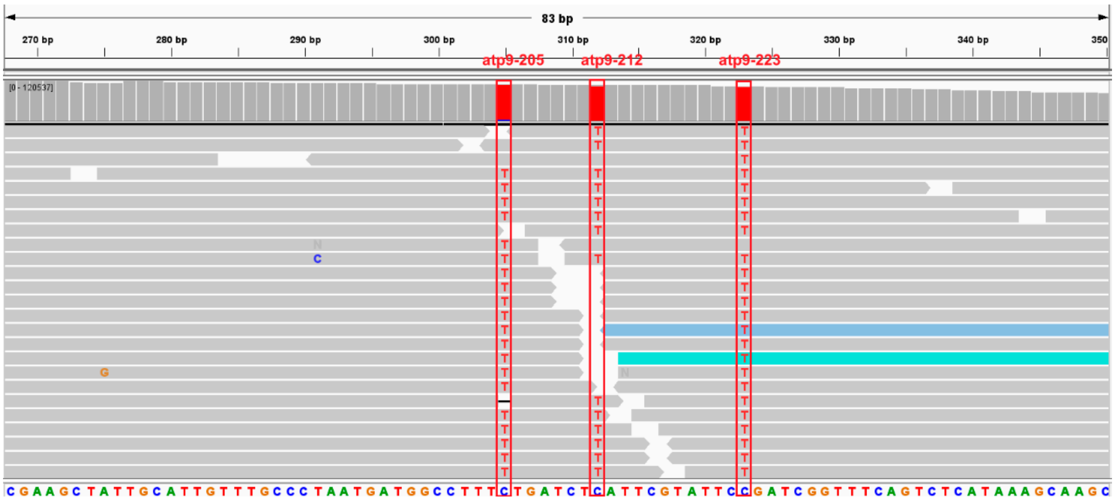

F. Mapping of RNA-seq reads to the coding sequence of *ccmB*.

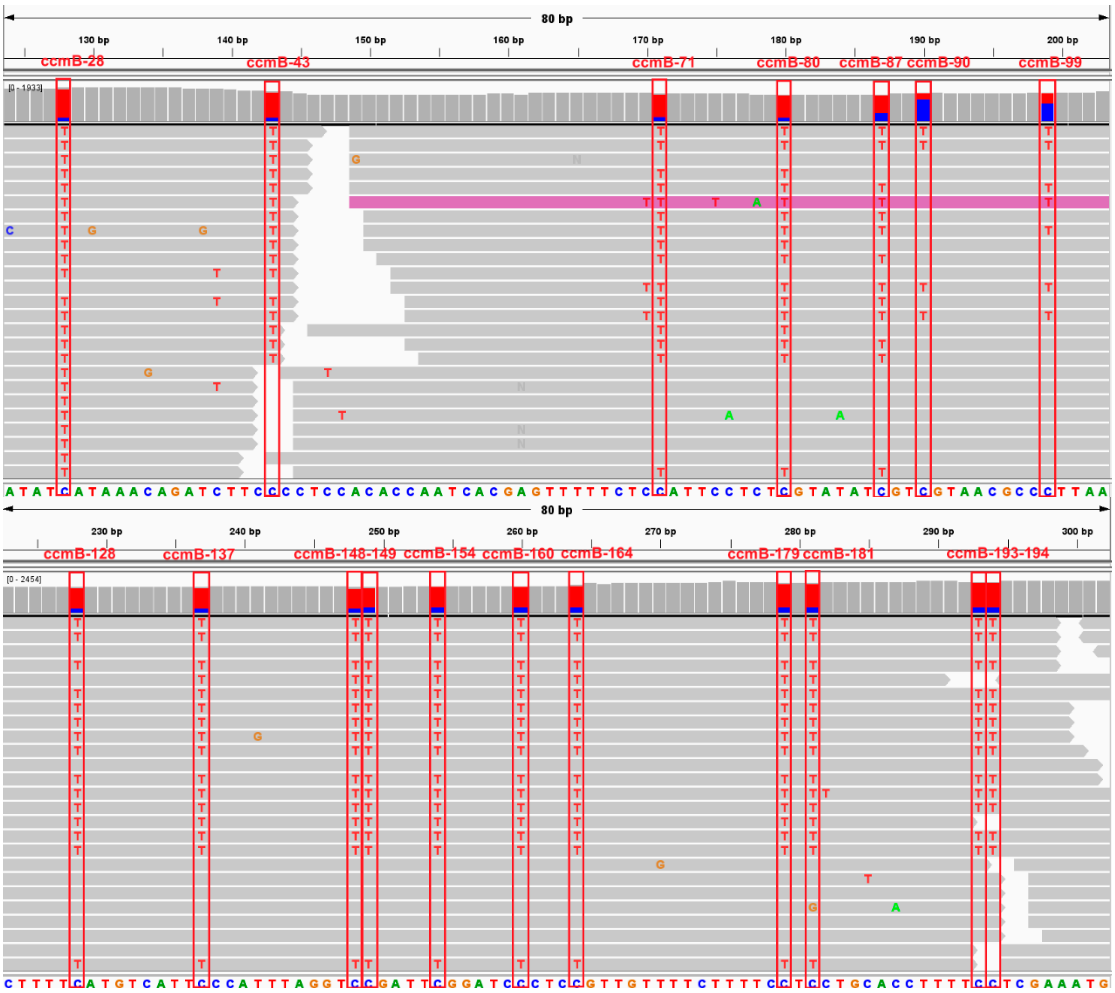

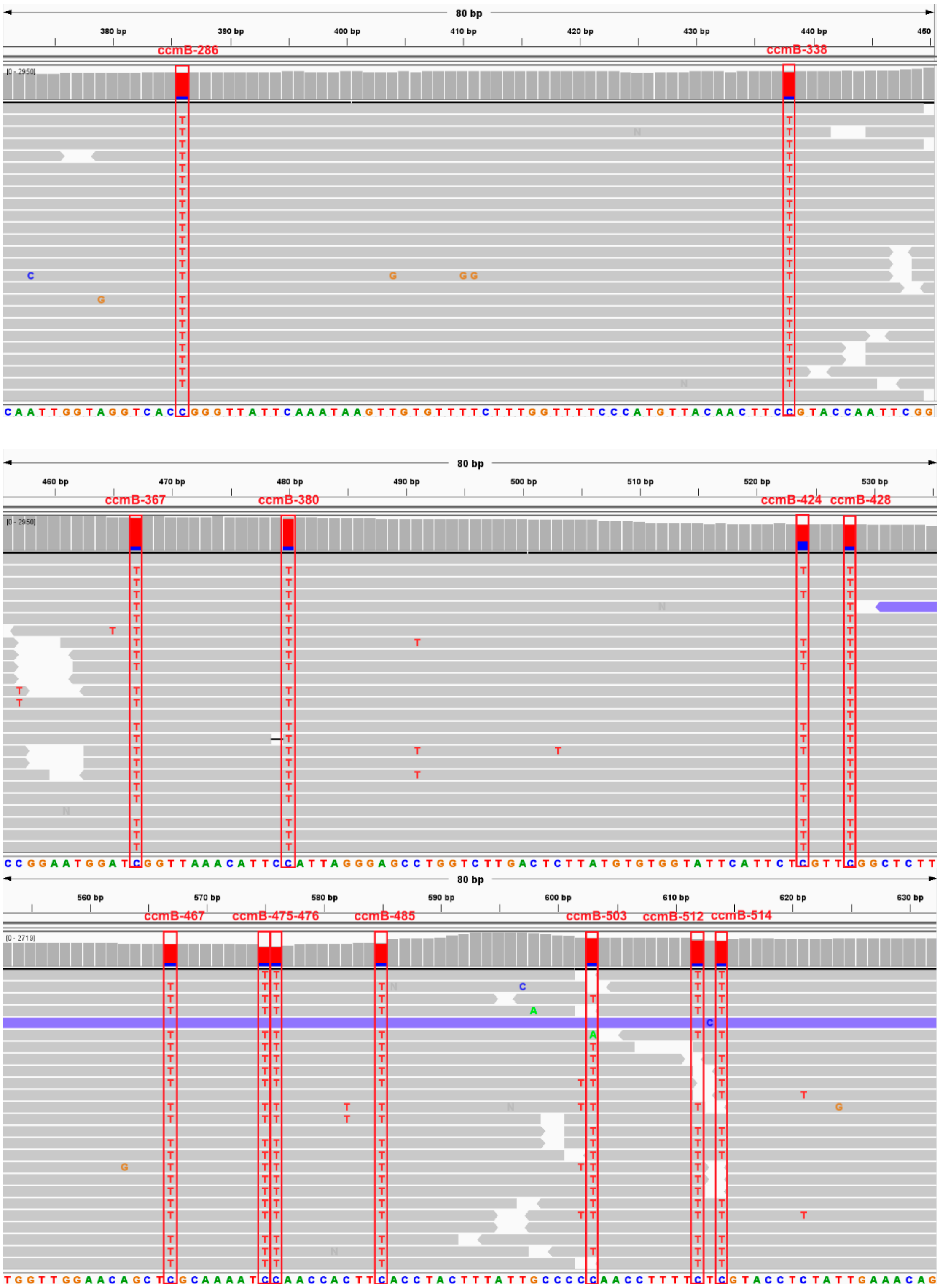

80 bp
380 bp
390 bp
400 bp
410 bp
420 bp
430 bp
440 bp
450
ccmB-286
ccmB-338
[0 - 2950]
80 bp
460 bp
470 bp
480 bp
490 bp
500 bp
510 bp
520 bp
530 bp
ccmB-367
ccmB-380
ccmB-424
ccmB-428
[0 - 2950]
80 bp
560 bp
570 bp
580 bp
590 bp
600 bp
610 bp
620 bp
630 bp
ccmB-467
ccmB-475-476
ccmB-485
ccmB-503
ccmB-512
ccmB-514
[0 - 2719]

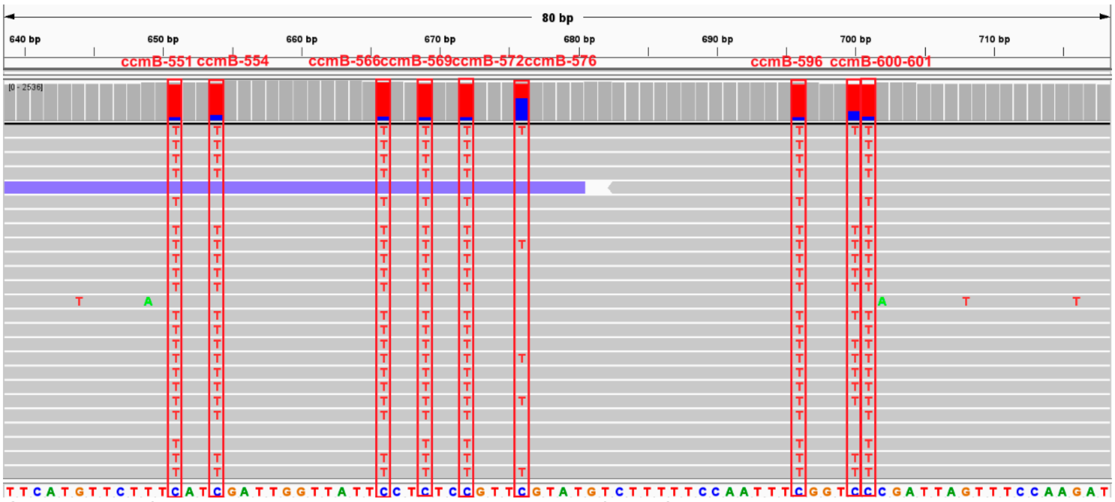

G. Mapping of RNA-seq reads to the coding sequence of *ccmC*.

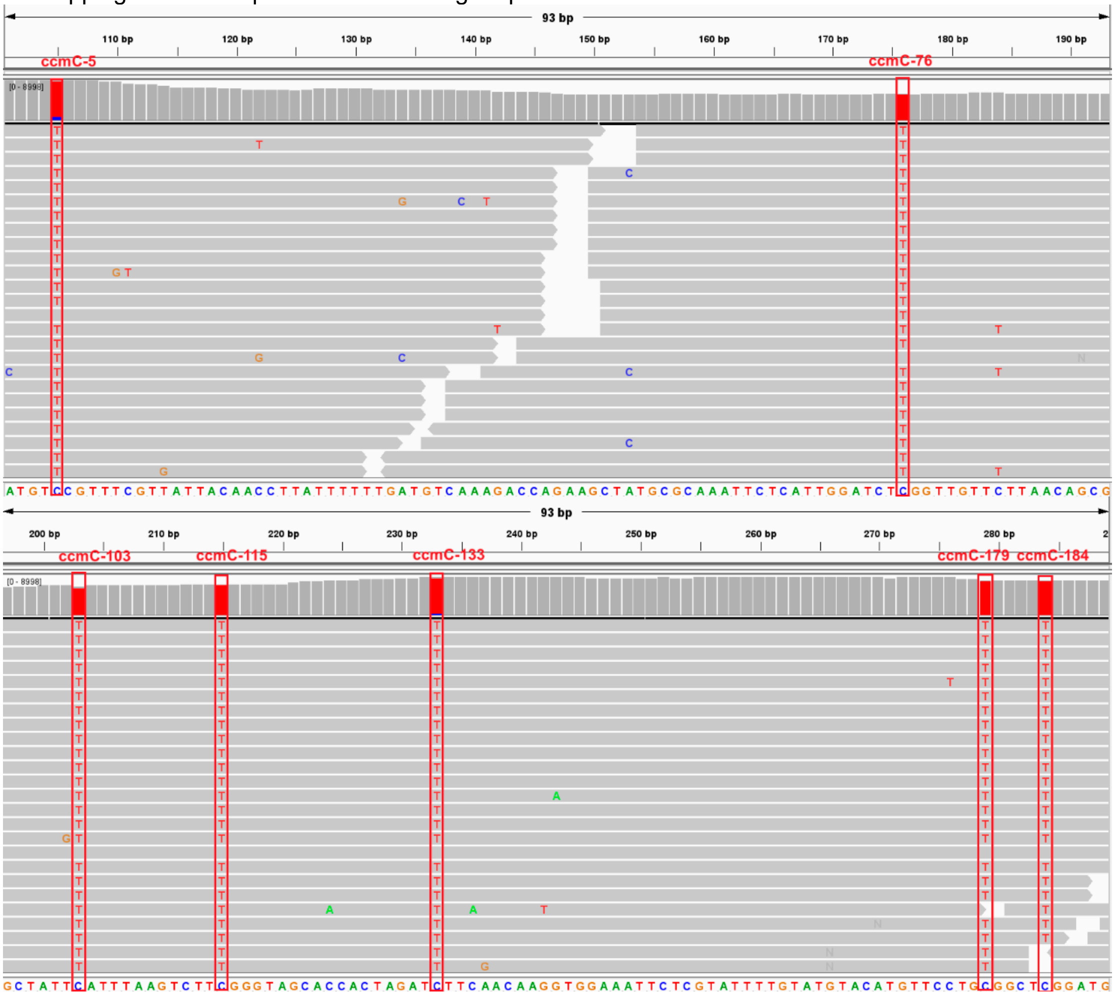

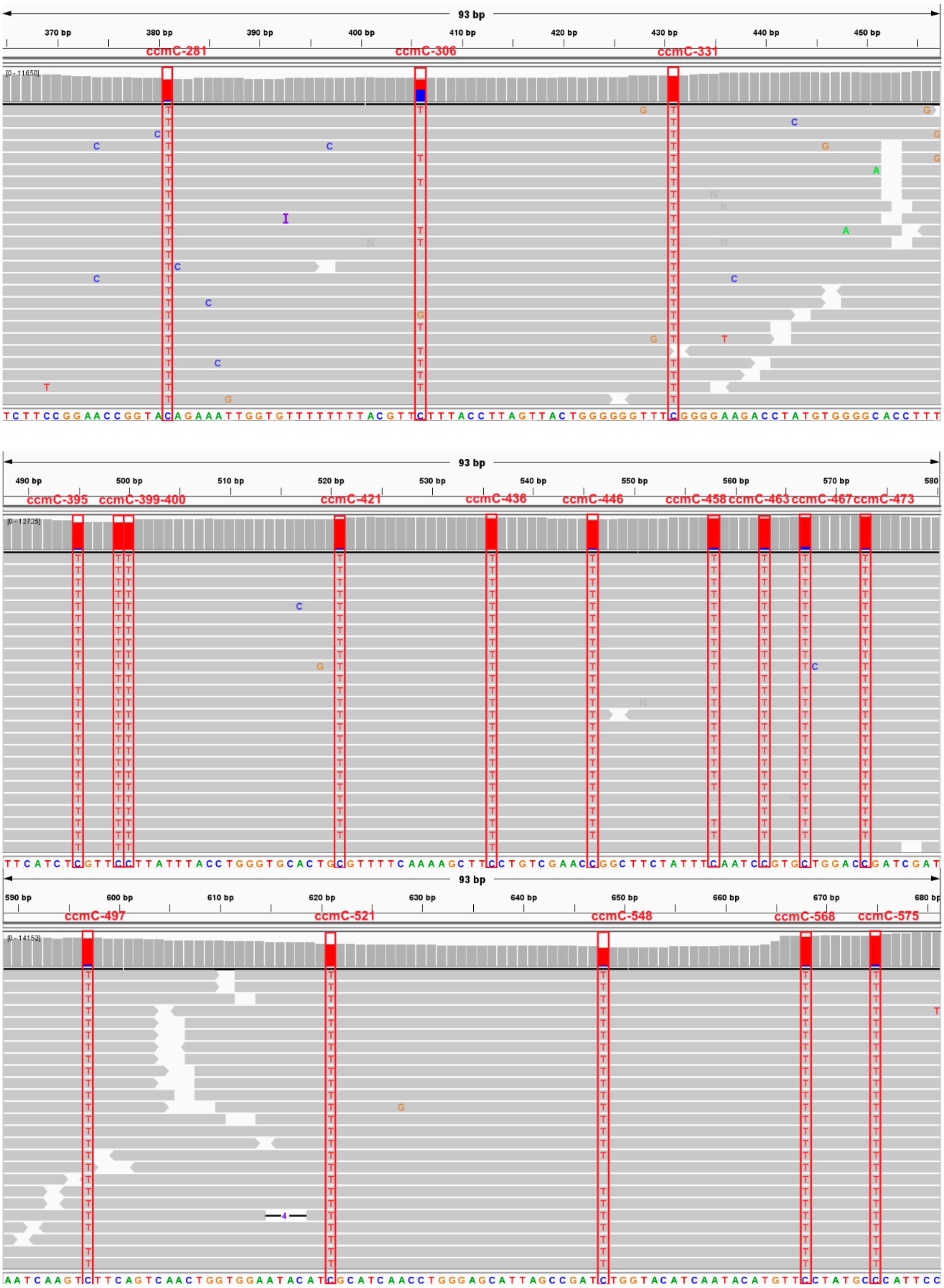
93 bp
370 bp
380 bp
390 bp
400 bp
410 bp
420 bp
430 bp
440 bp
450 bp
ccmC-281
ccmC-306
ccmC-331
93 bp
490 bp
500 bp
510 bp
520 bp
530 bp
540 bp
550 bp
560 bp
570 bp
580
ccmC-395
ccmC-399-400
ccmC-421
ccmC-436
ccmC-446
ccmC-458
ccmC-463
ccmC-467
ccmC-473
93 bp
590 bp
600 bp
610 bp
620 bp
630 bp
640 bp
650 bp
660 bp
670 bp
680 bp
ccmC-497
ccmC-521
ccmC-548
ccmC-568
ccmC-575

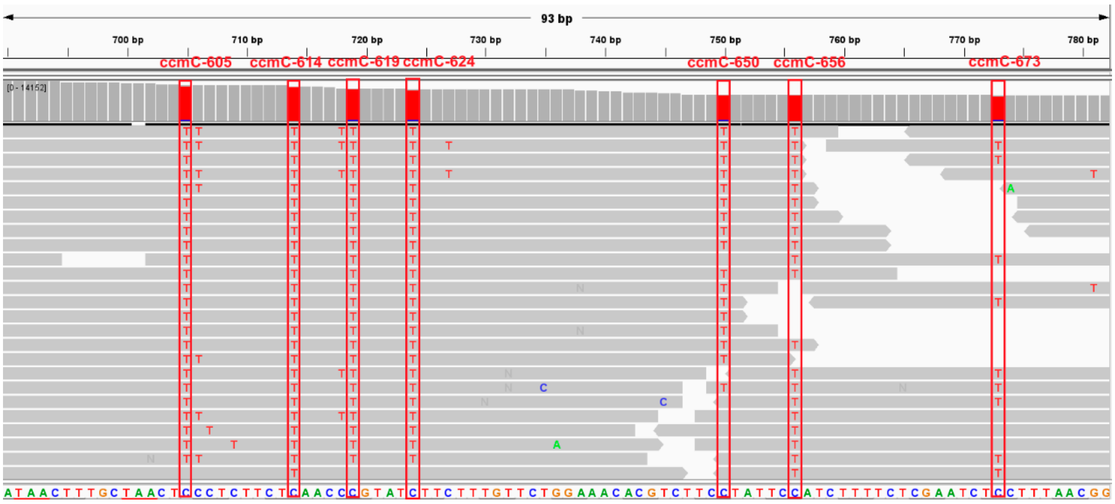

H.Mapping of RNA-seq reads to the coding sequence of *ccmFN*.

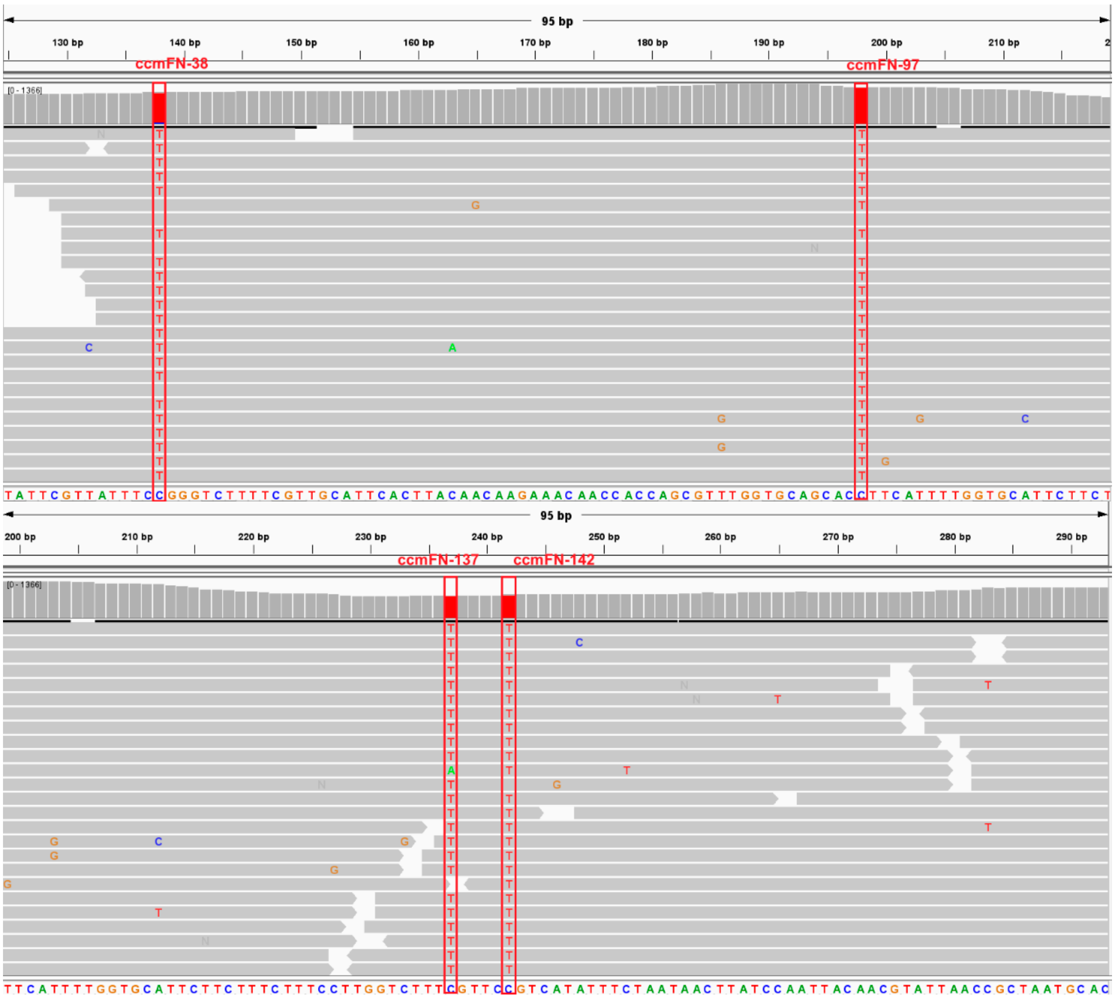

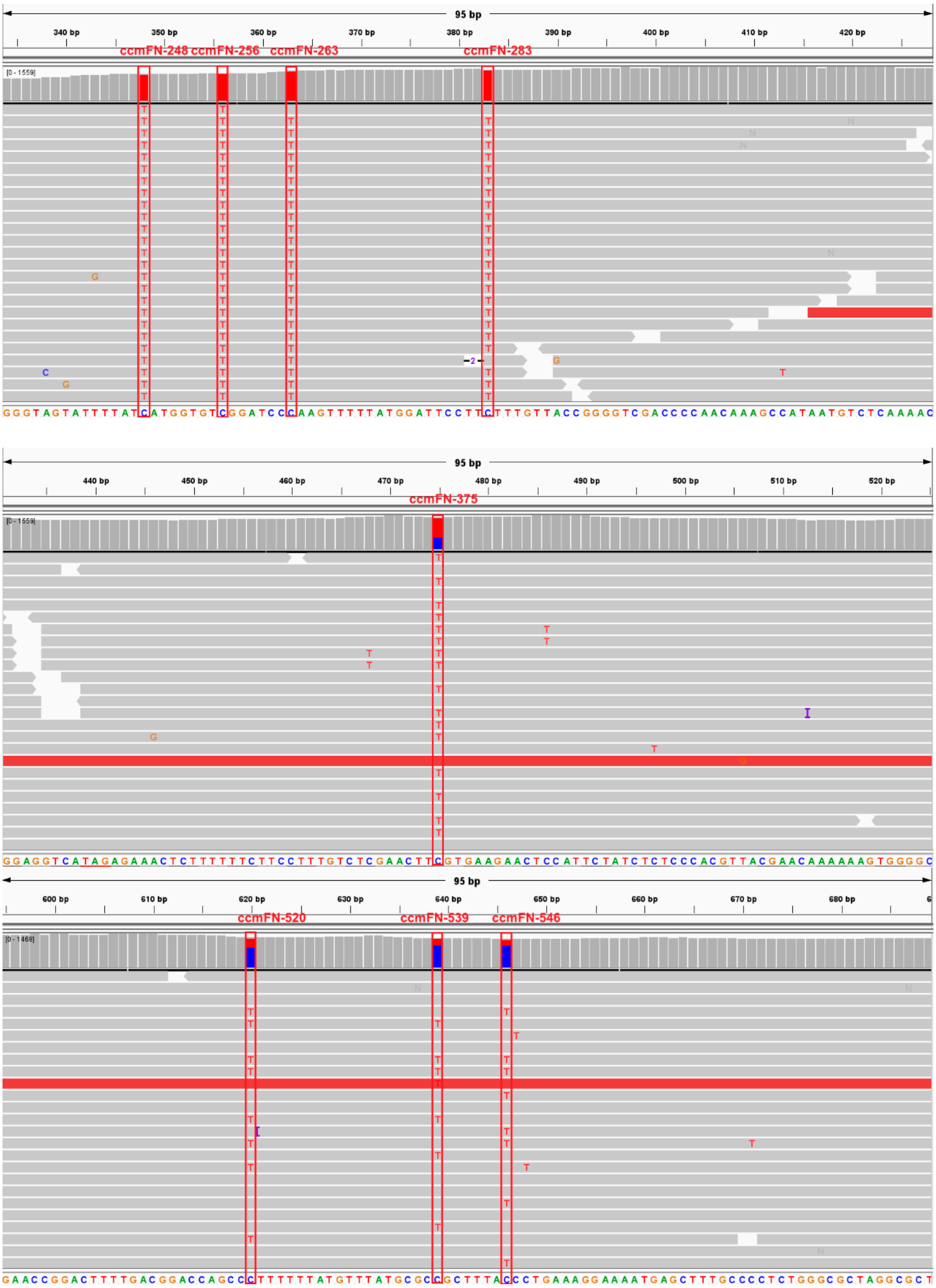

95 bp
340 bp
350 bp
360 bp
370 bp
380 bp
390 bp
400 bp
410 bp
420 bp
ccmFN-248
ccmFN-256
ccmFN-263
ccmFN-283
[0 - 1559]
95 bp
440 bp
450 bp
460 bp
470 bp
480 bp
490 bp
500 bp
510 bp
520 bp
ccmFN-375
[0 - 1559]
95 bp
600 bp
610 bp
620 bp
630 bp
640 bp
650 bp
660 bp
670 bp
680 bp
ccmFN-520
ccmFN-539
ccmFN-546
[0 - 1468]

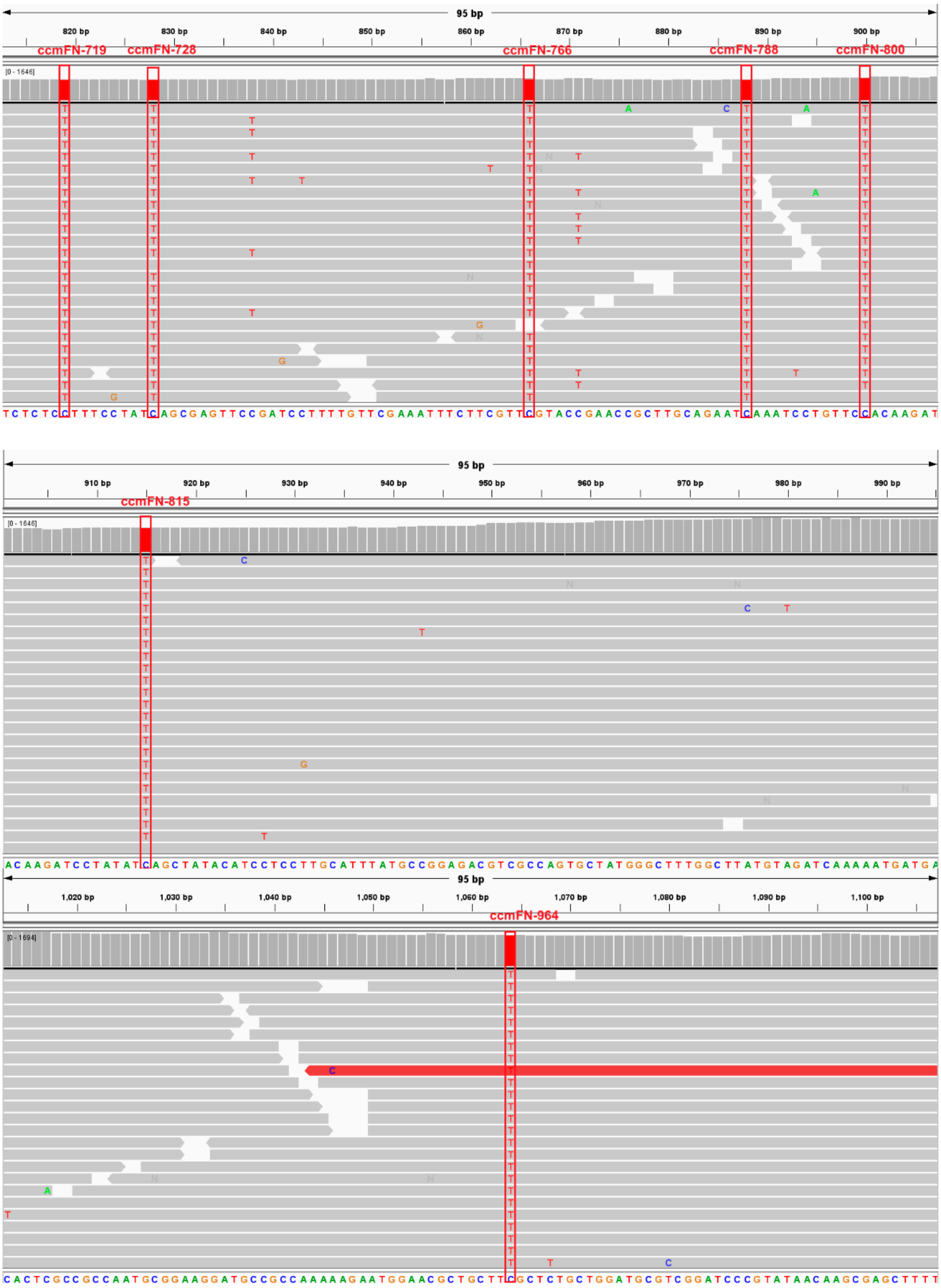

95 bp
820 bp
830 bp
840 bp
850 bp
860 bp
870 bp
880 bp
890 bp
900 bp
ccmFN-719
ccmFN-728
ccmFN-766
ccmFN-788
ccmFN-800
[0 - 1646]
95 bp
910 bp
920 bp
930 bp
940 bp
950 bp
960 bp
970 bp
980 bp
990 bp
ccmFN-815
[0 - 1646]
95 bp
1,020 bp
1,030 bp
1,040 bp
1,050 bp
1,060 bp
1,070 bp
1,080 bp
1,090 bp
1,100 bp
ccmFN-964
[0 - 1694]

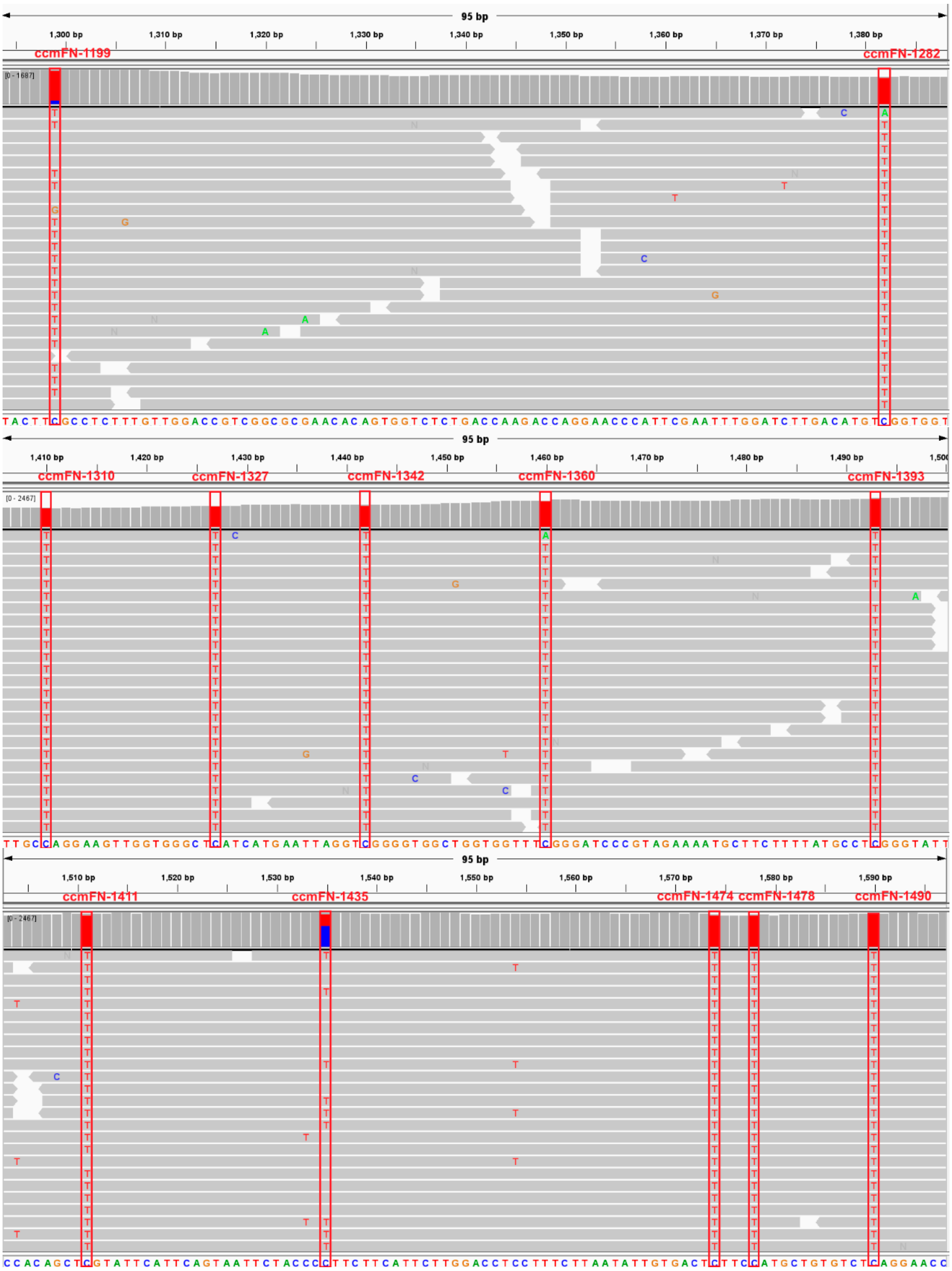
95 bp
1,300 bp
1,310 bp
1,320 bp
1,330 bp
1,340 bp
1,350 bp
1,360 bp
1,370 bp
1,380 bp
ccmFN-1199
ccmFN-1282
[0 - 1687]
95 bp
1,410 bp
1,420 bp
1,430 bp
1,440 bp
1,450 bp
1,460 bp
1,470 bp
1,480 bp
1,490 bp
ccmFN-1310
ccmFN-1327
ccmFN-1342
ccmFN-1360
ccmFN-1393
[0 - 2467]
95 bp
1,510 bp
1,520 bp
1,530 bp
1,540 bp
1,550 bp
1,560 bp
1,570 bp
1,580 bp
1,590 bp
ccmFN-1411
ccmFN-1435
ccmFN-1474
ccmFN-1478
ccmFN-1490
[0 - 2467]

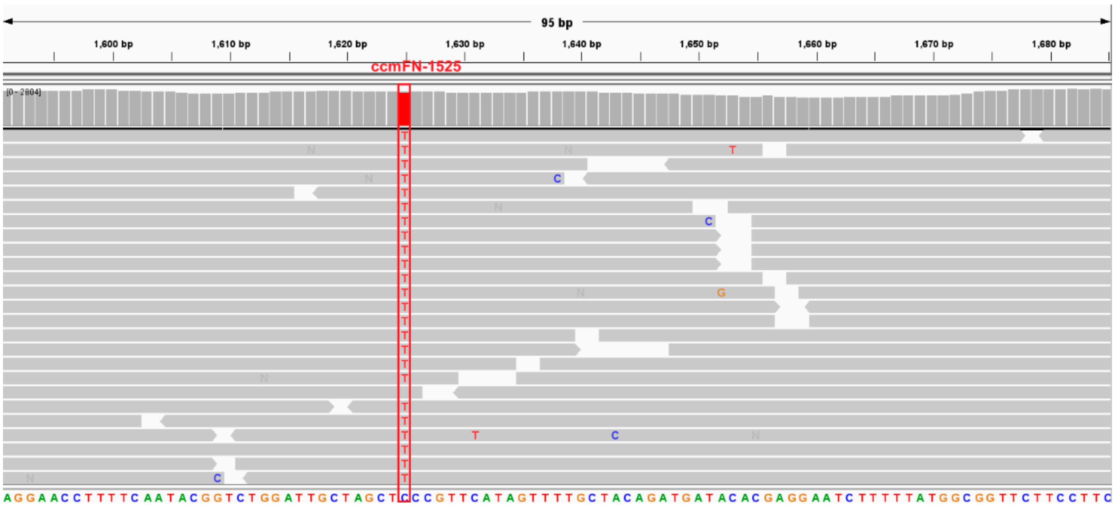

I.Mapping of RNA-seq reads to the coding sequence of *cob*.

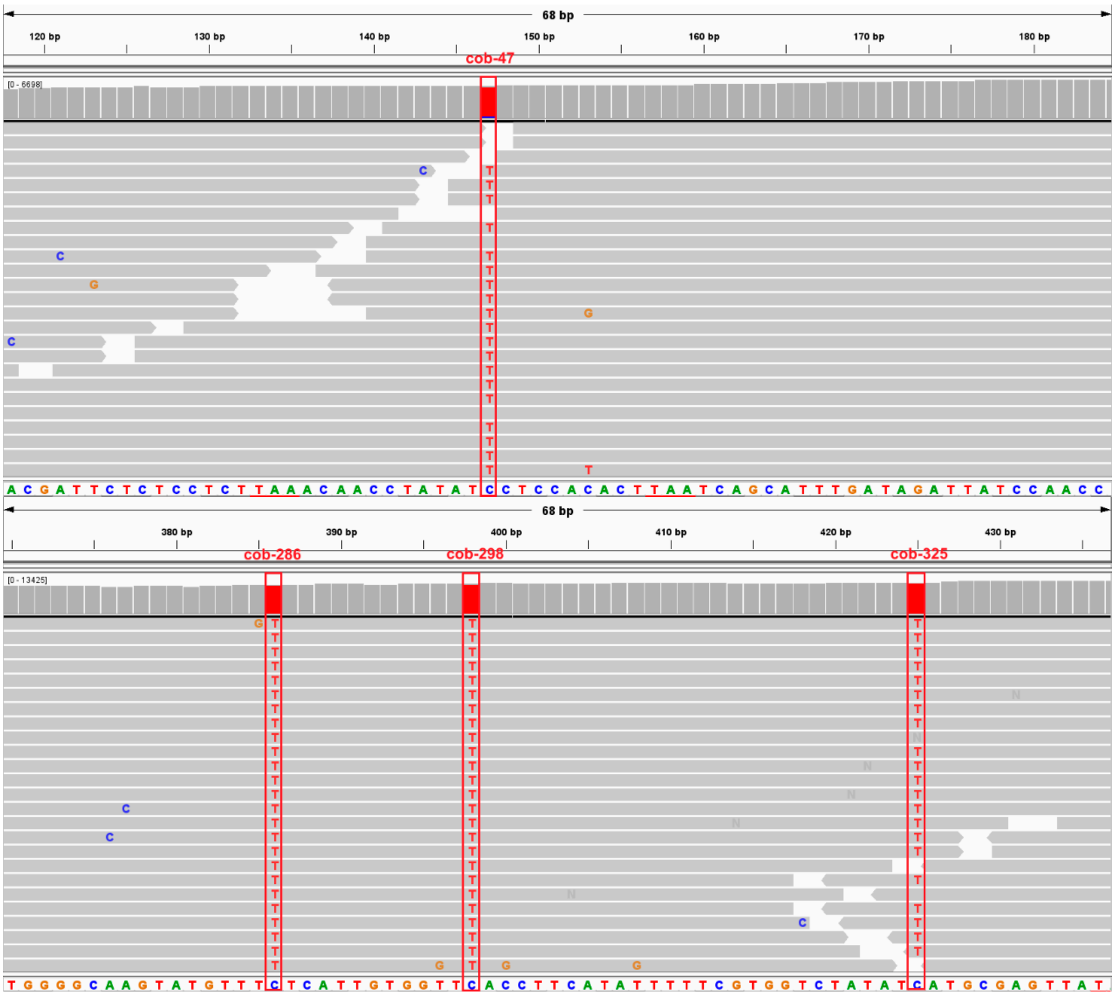

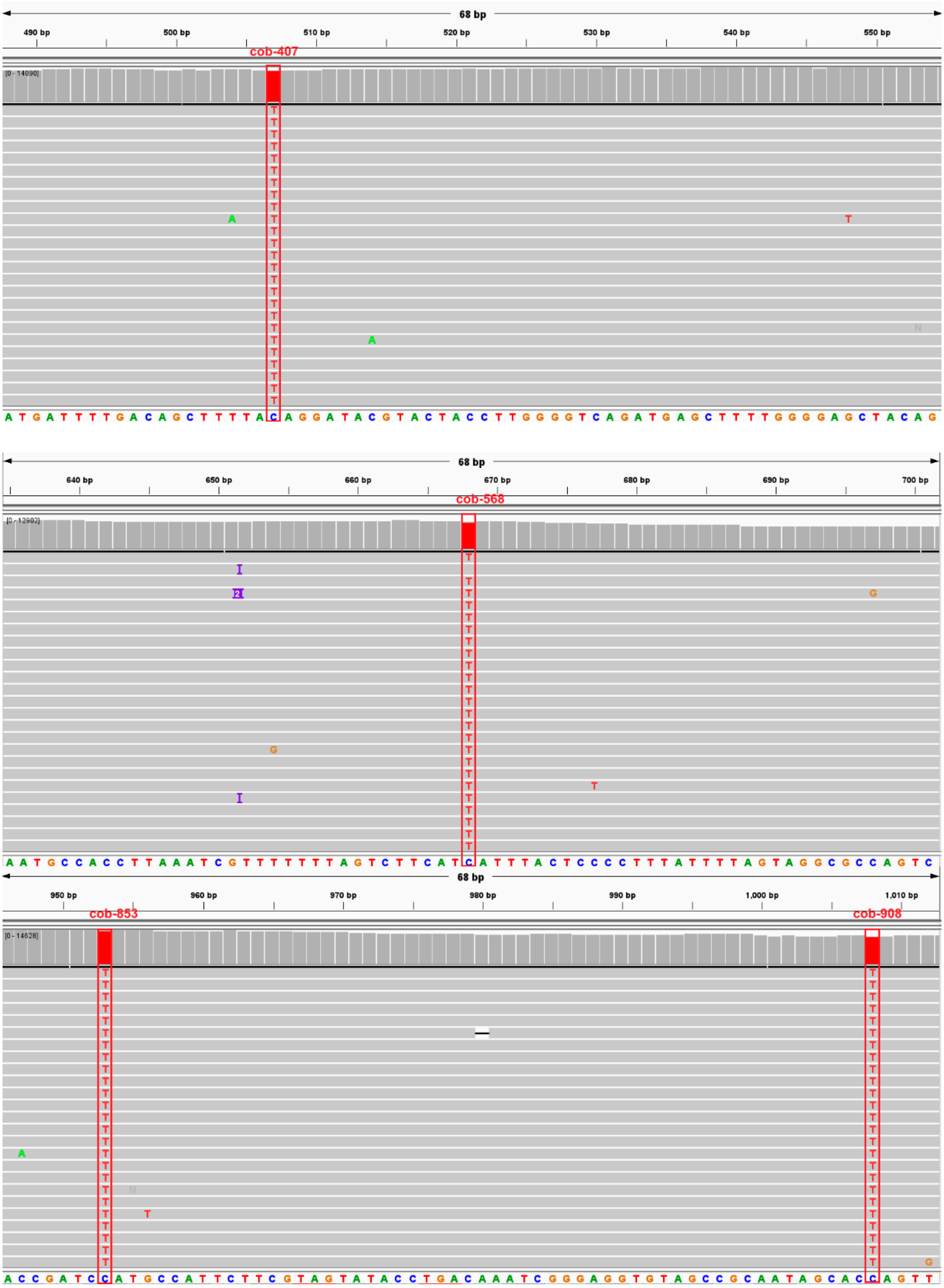

68 bp
490 bp
500 bp
510 bp
520 bp
530 bp
540 bp
550 bp
cob-407
[0 - 14090]
68 bp
640 bp
650 bp
660 bp
670 bp
680 bp
690 bp
700 bp
cob-568
[0 - 12902]
68 bp
950 bp
960 bp
970 bp
980 bp
990 bp
1,000 bp
1,010 bp
cob-853
cob-908
[0 - 14628]

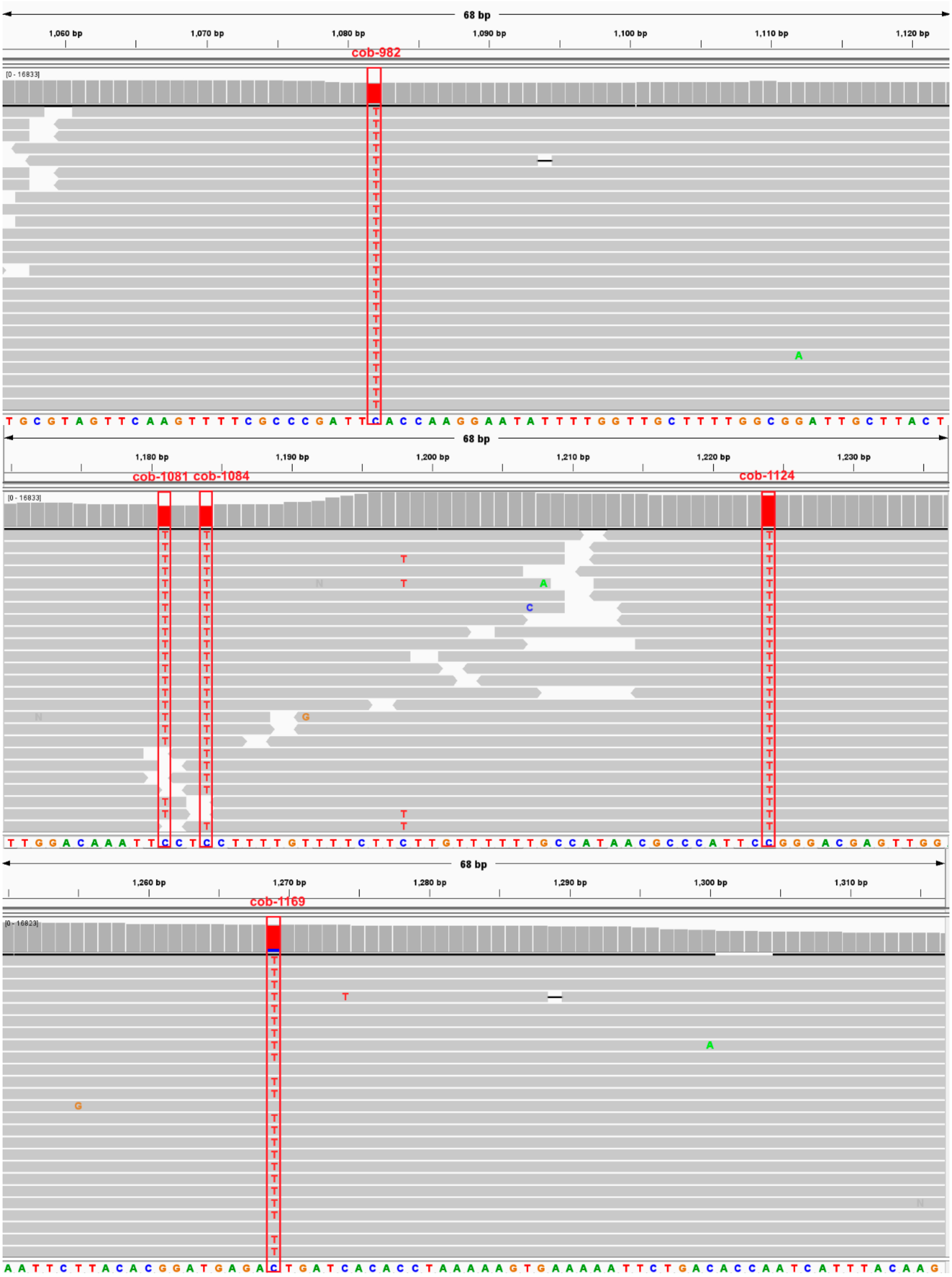

J. Mapping of RNA-seq reads to the coding sequence of *cox1*.

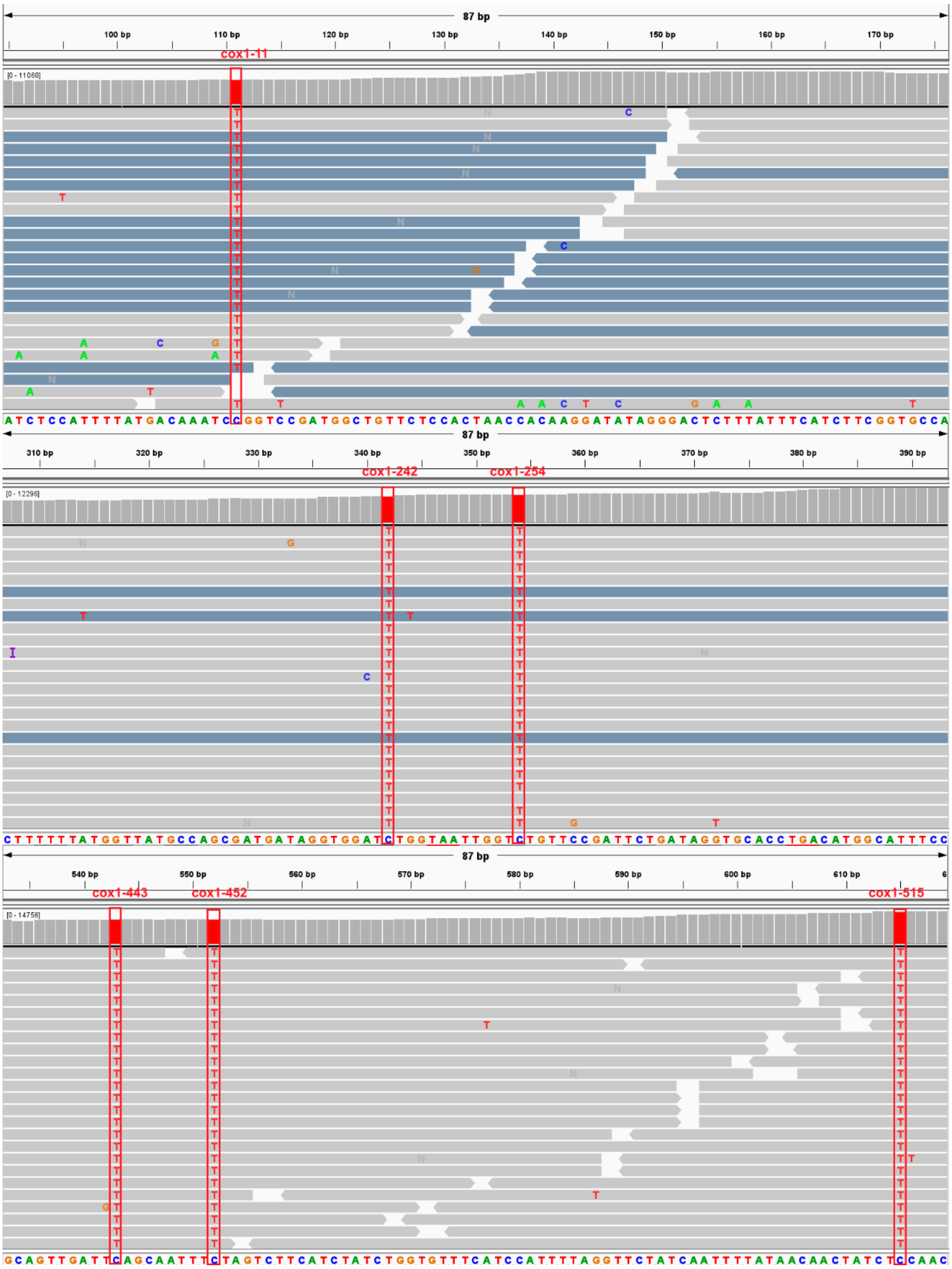
87 bp
100 bp
110 bp
120 bp
130 bp
140 bp
150 bp
160 bp
170 bp
cox1-11
[0 - 11060]
87 bp
310 bp
320 bp
330 bp
340 bp
350 bp
360 bp
370 bp
380 bp
390 bp
cox1-242
cox1-254
[0 - 12296]
87 bp
540 bp
550 bp
560 bp
570 bp
580 bp
590 bp
600 bp
610 bp
cox1-443
cox1-452
cox1-515
[0 - 14758]

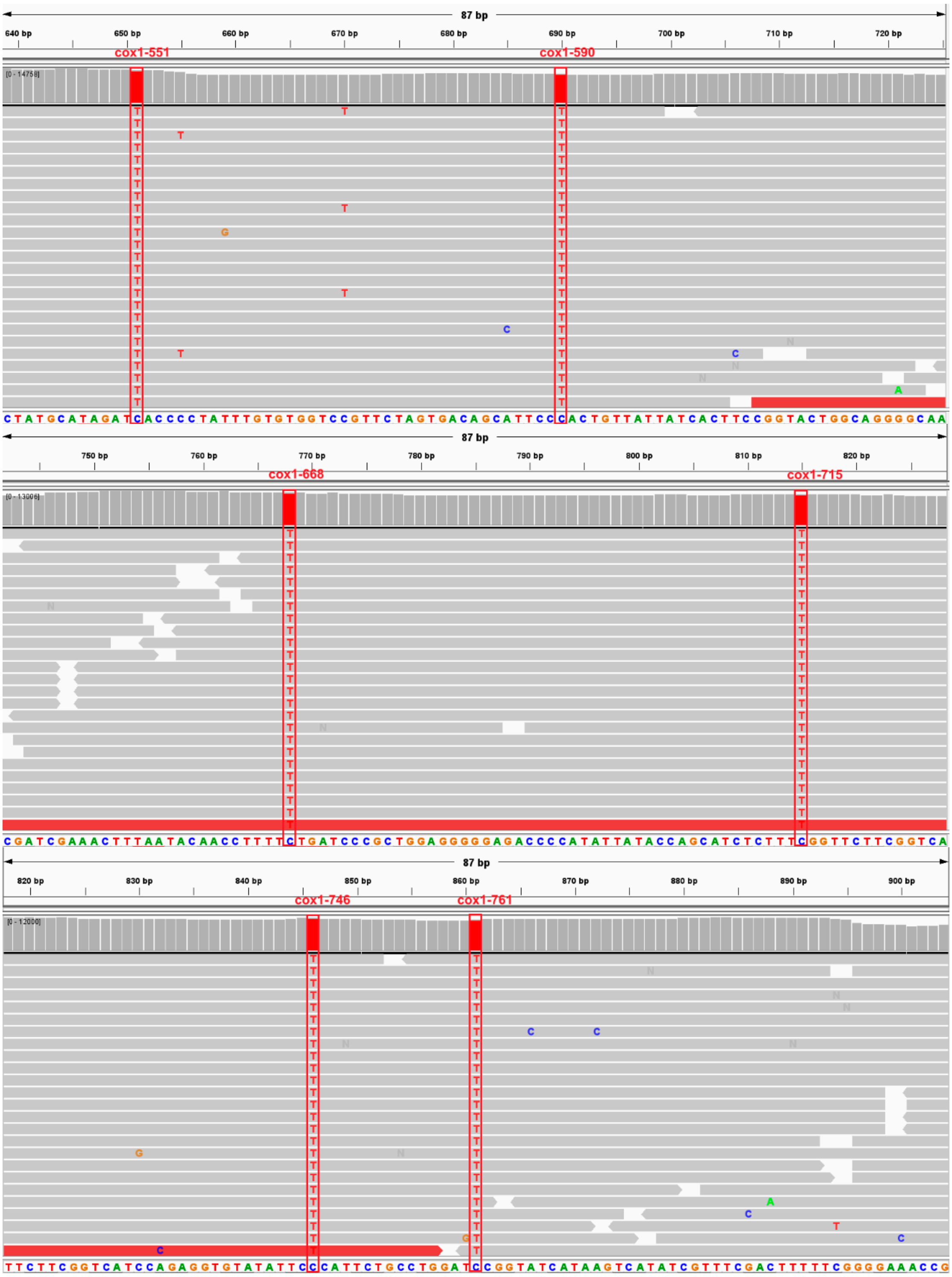
87 bp
640 bp
650 bp
660 bp
670 bp
680 bp
690 bp
700 bp
710 bp
720 bp
cox1-551
cox1-590
87 bp
750 bp
760 bp
770 bp
780 bp
790 bp
800 bp
810 bp
820 bp
cox1-668
cox1-715
87 bp
820 bp
830 bp
840 bp
850 bp
860 bp
870 bp
880 bp
890 bp
900 bp
cox1-746
cox1-761

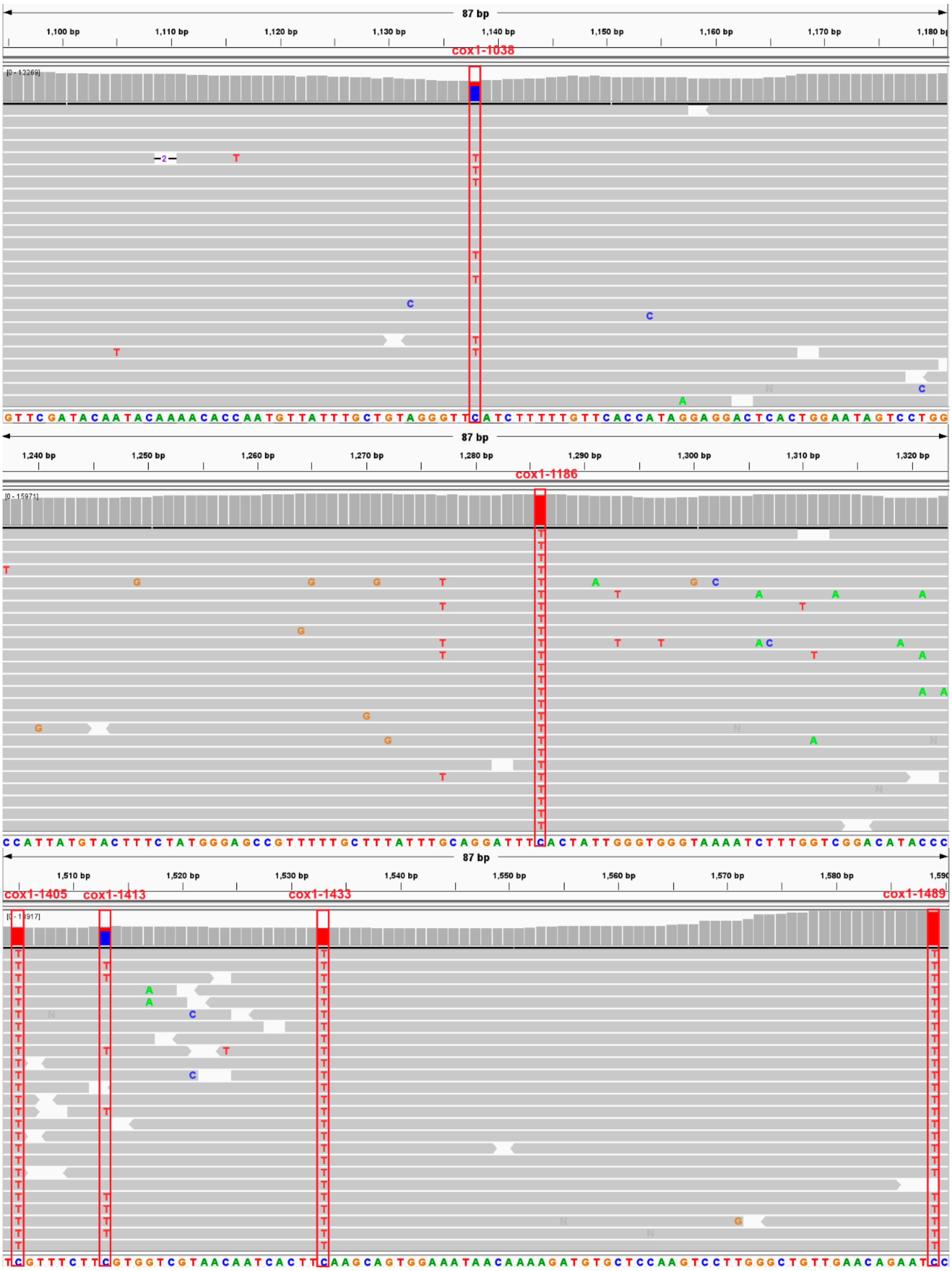

87 bp
1,100 bp
1,110 bp
1,120 bp
1,130 bp
1,140 bp
1,150 bp
1,160 bp
1,170 bp
cox1-1038
87 bp
1,240 bp
1,250 bp
1,260 bp
1,270 bp
1,280 bp
1,290 bp
1,300 bp
1,310 bp
1,320 bp
cox1-1186
87 bp
1,510 bp
1,520 bp
1,530 bp
1,540 bp
1,550 bp
1,560 bp
1,570 bp
1,580 bp
cox1-1405
cox1-1413
cox1-1433
cox1-1489

K. Mapping of RNA-seq reads to the coding sequence of *cox2*.

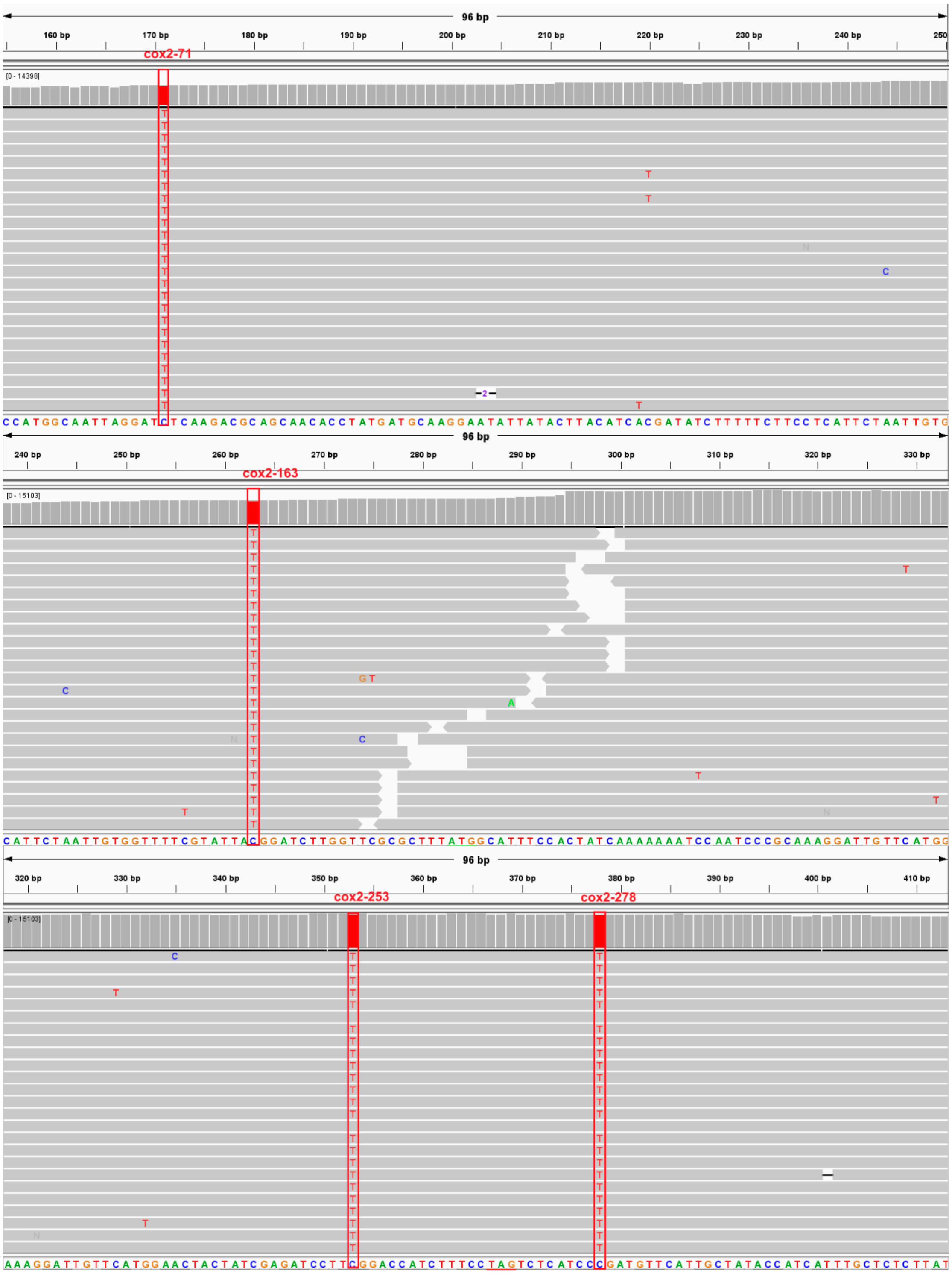

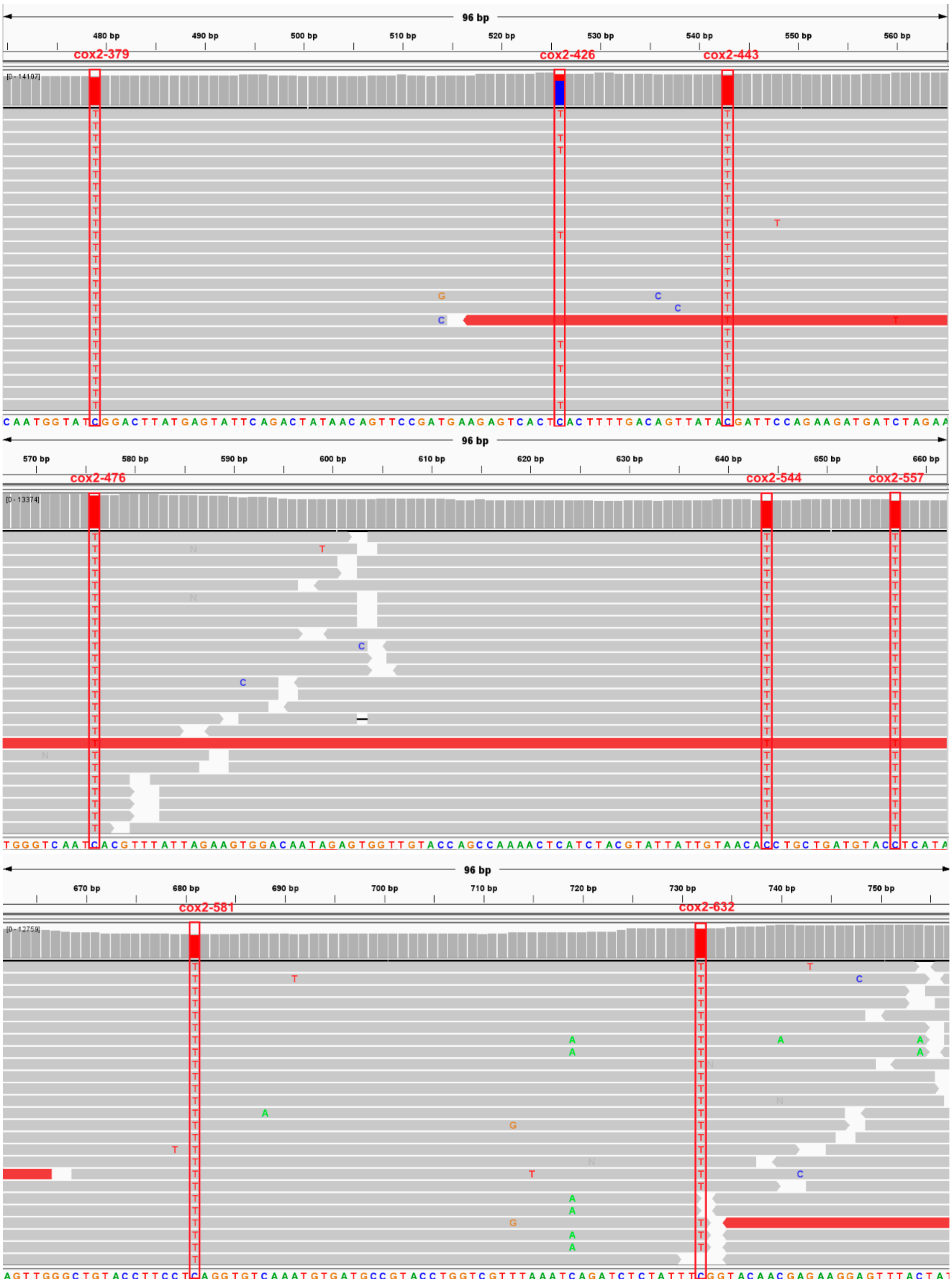
96 bp
480 bp
490 bp
500 bp
510 bp
520 bp
530 bp
540 bp
550 bp
560 bp
cox2-379
cox2-426
cox2-443
[0 - 14107]
96 bp
570 bp
580 bp
590 bp
600 bp
610 bp
620 bp
630 bp
640 bp
650 bp
660 bp
cox2-476
cox2-544
cox2-557
[0 - 13374]
96 bp
670 bp
680 bp
690 bp
700 bp
710 bp
720 bp
730 bp
740 bp
750 bp
cox2-581
cox2-632
[0 - 12759]

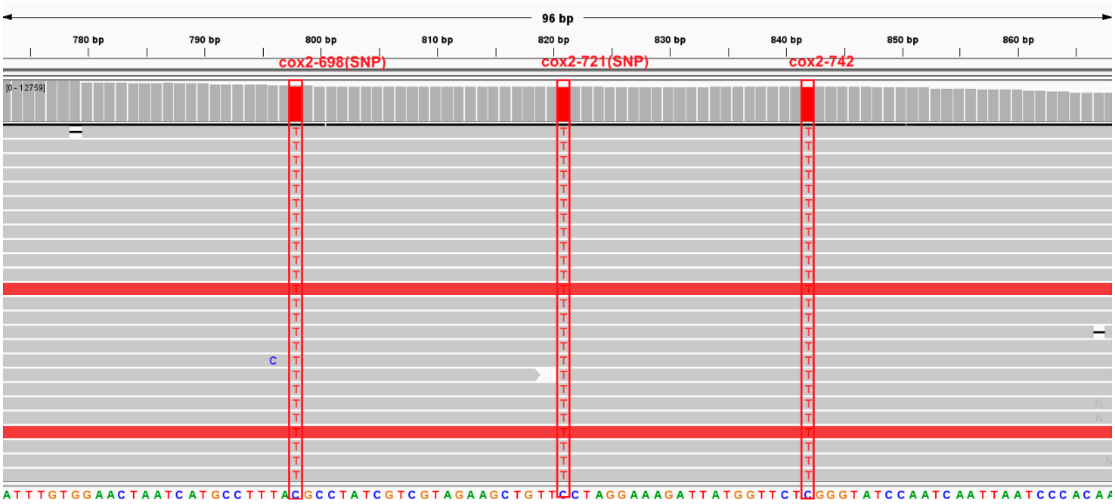

L.Mapping of RNA-seq reads to the coding sequence of *cox3*.

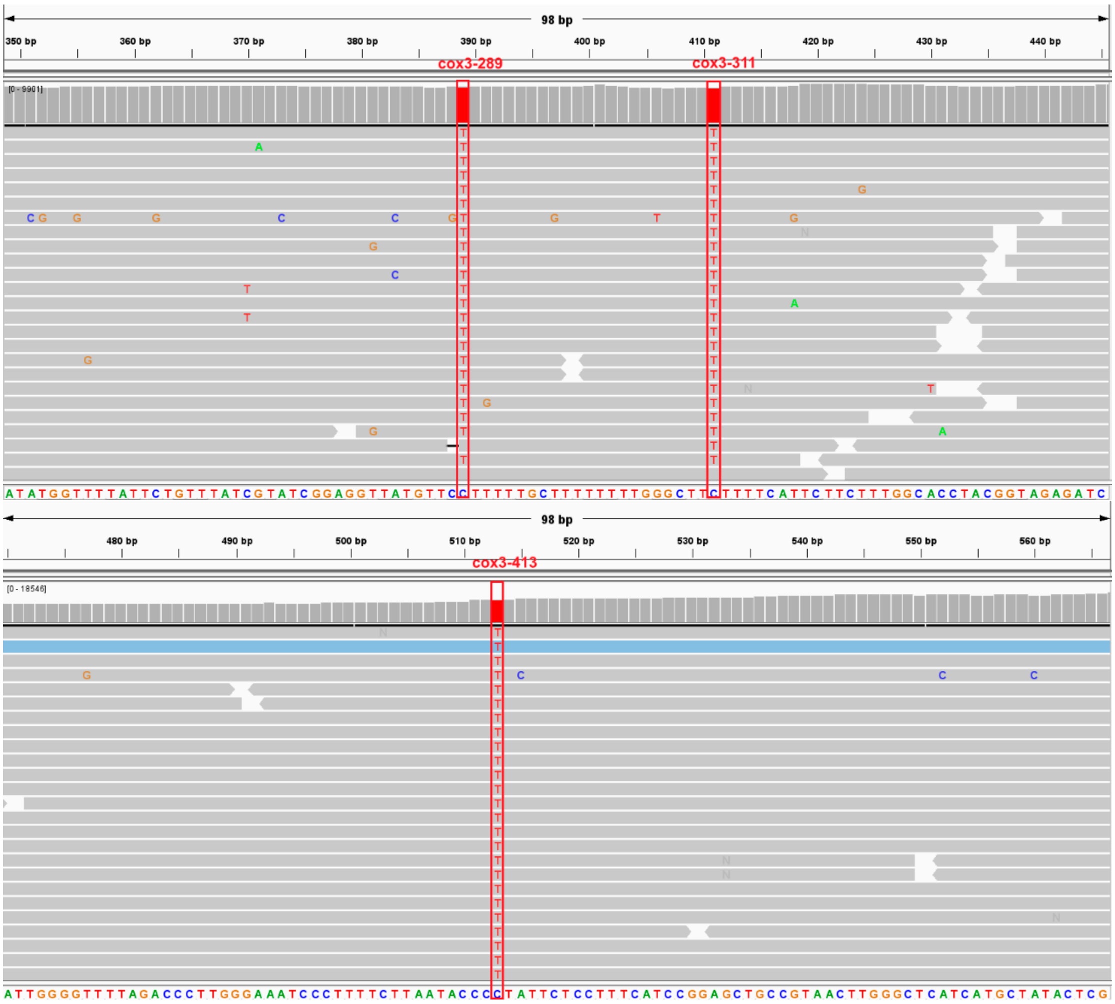

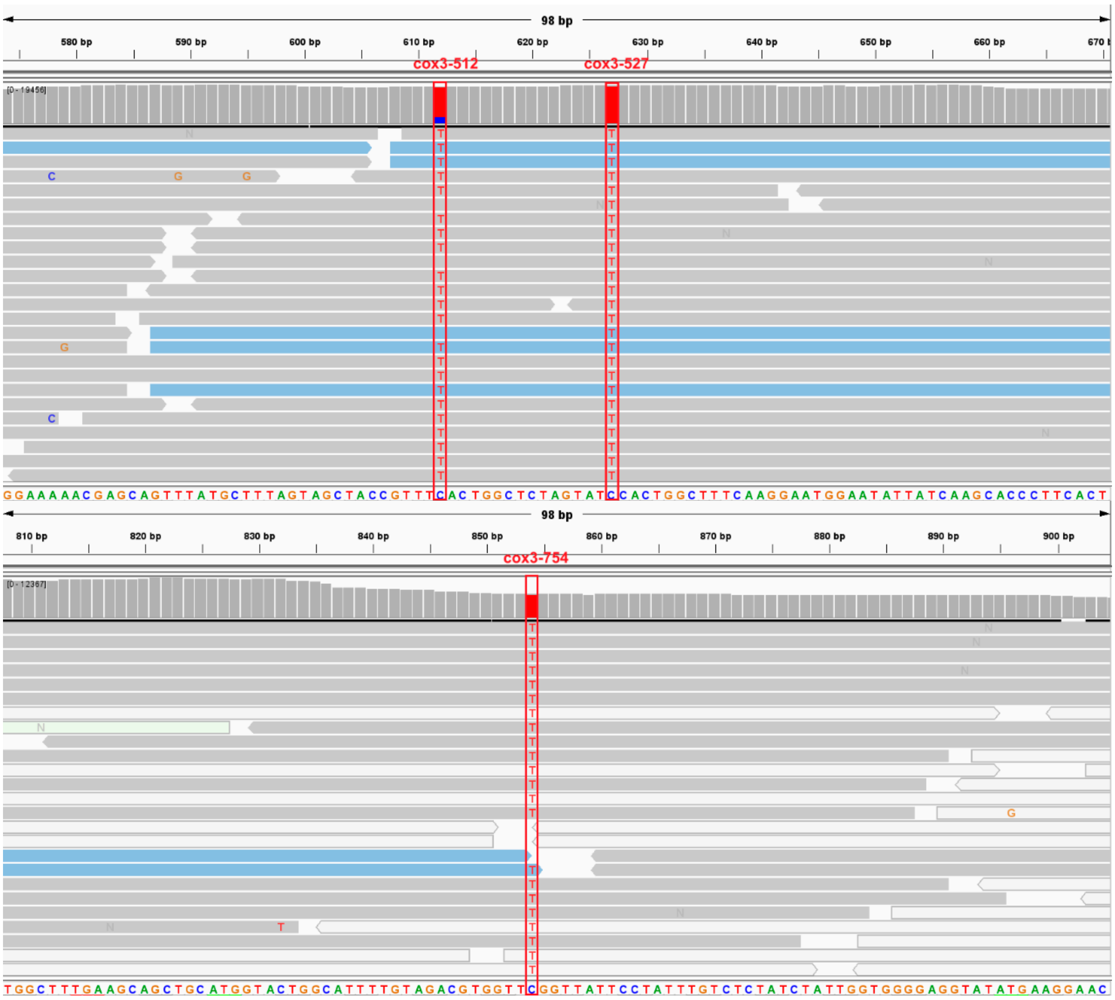

98 bp
580 bp
590 bp
600 bp
610 bp
620 bp
630 bp
640 bp
650 bp
660 bp
cox3-512
cox3-527
98 bp
810 bp
820 bp
830 bp
840 bp
850 bp
860 bp
870 bp
880 bp
890 bp
900 bp
cox3-754

M. Mapping of RNA-seq reads to the coding sequence of *matR*.

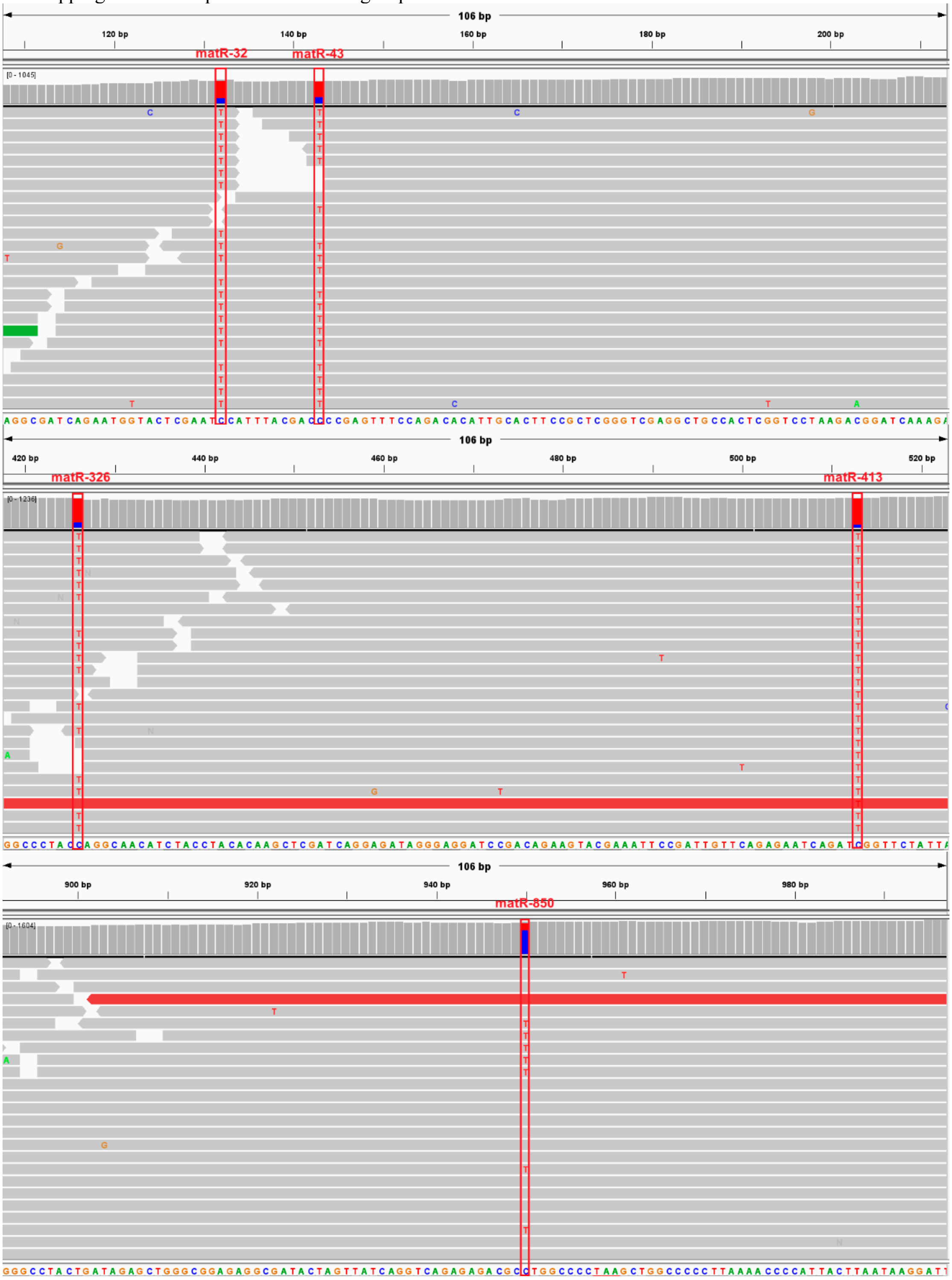

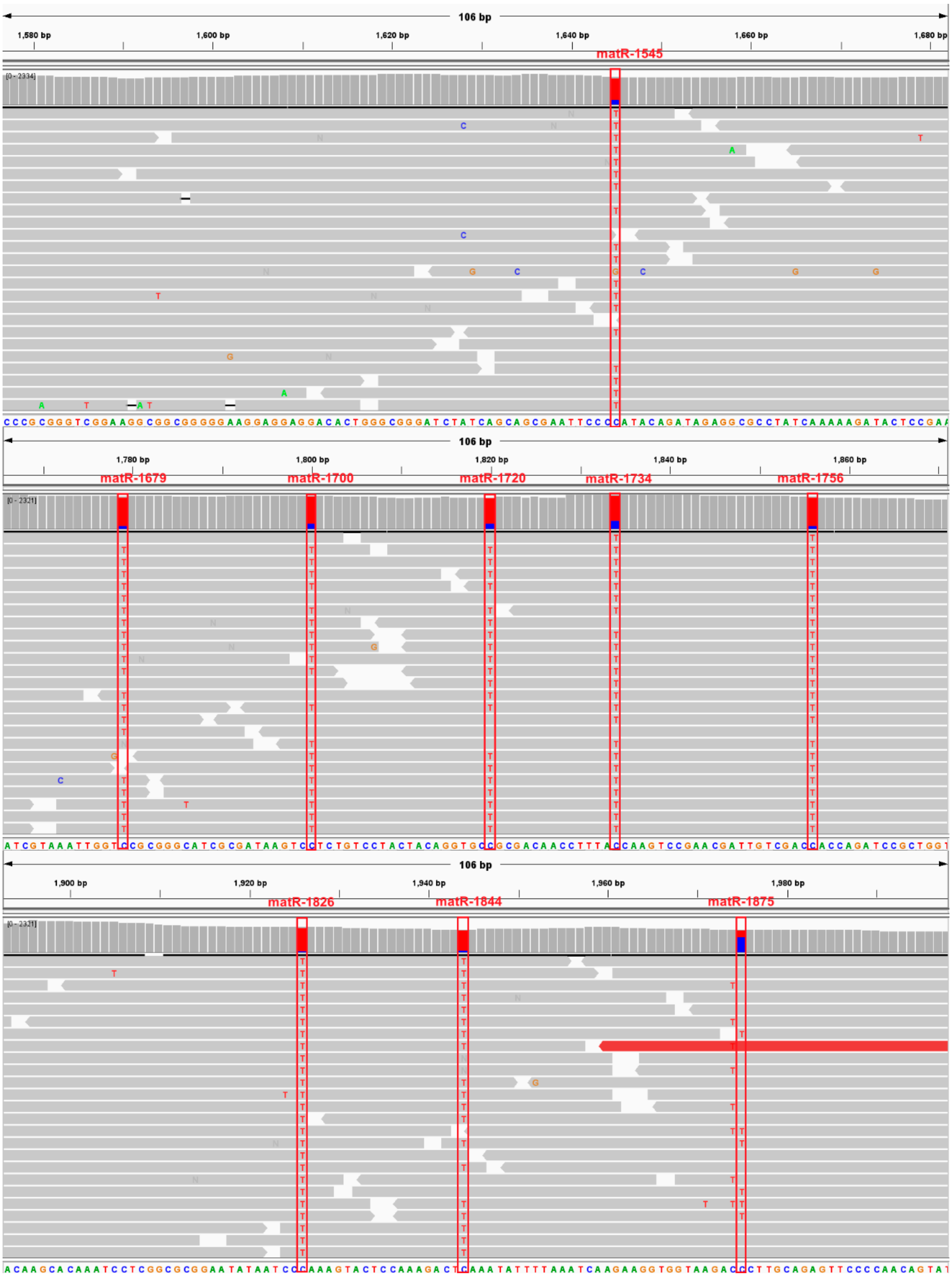

N. Mapping of RNA-seq reads to the coding sequence of *mttB*.

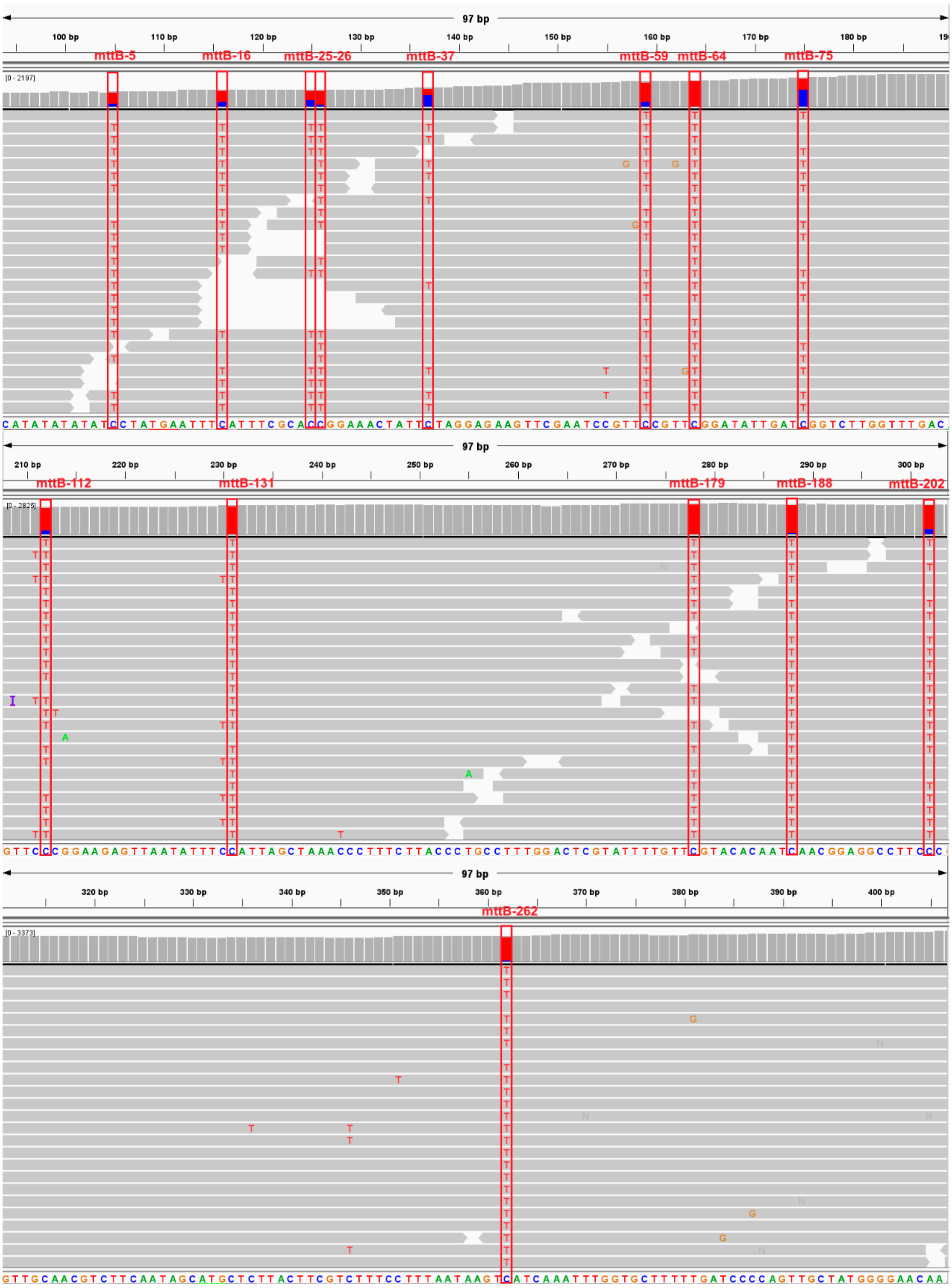
97 bp
100 bp
110 bp
120 bp
130 bp
140 bp
150 bp
160 bp
170 bp
180 bp
mttB-5
mttB-16
mttB-25-26
mttB-37
mttB-59
mttB-64
mttB-75
[0 - 2197]
97 bp
210 bp
220 bp
230 bp
240 bp
250 bp
260 bp
270 bp
280 bp
290 bp
300 bp
mttB-112
mttB-131
mttB-179
mttB-188
mttB-202
[0 - 2825]
97 bp
320 bp
330 bp
340 bp
350 bp
360 bp
370 bp
380 bp
390 bp
400 bp
mttB-262
[0 - 3373]

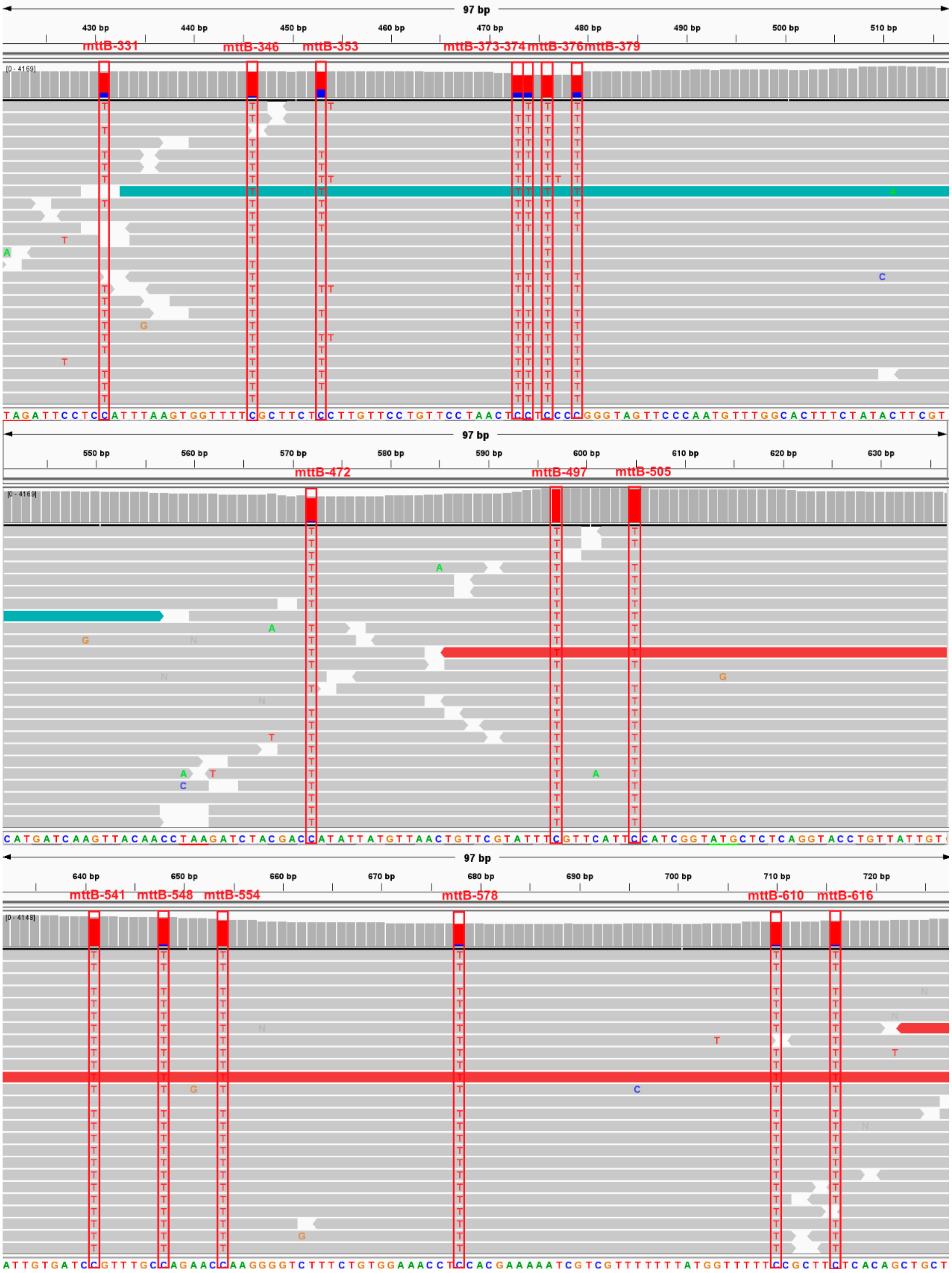
97 bp
430 bp
440 bp
450 bp
460 bp
470 bp
480 bp
490 bp
500 bp
510 bp
mttB-331
mttB-346
mttB-353
mttB-373-374
mttB-376
mttB-379
97 bp
550 bp
560 bp
570 bp
580 bp
590 bp
600 bp
610 bp
620 bp
630 bp
mttB-472
mttB-497
mttB-505
97 bp
640 bp
650 bp
660 bp
670 bp
680 bp
690 bp
700 bp
710 bp
720 bp
mttB-541
mttB-548
mttB-554
mttB-578
mttB-610
mttB-616

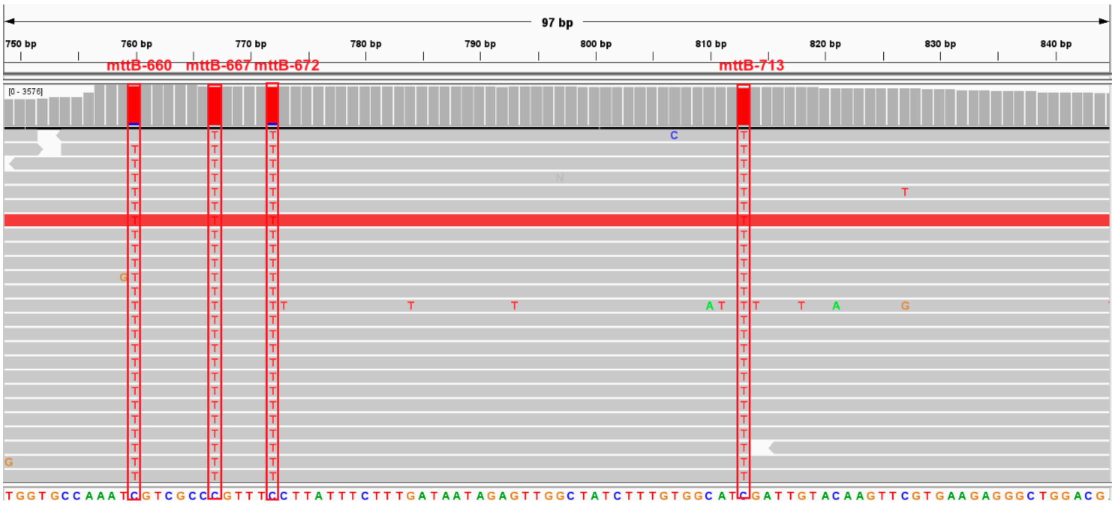

O. Mapping of RNA-seq reads to the coding sequence of *nad1*.

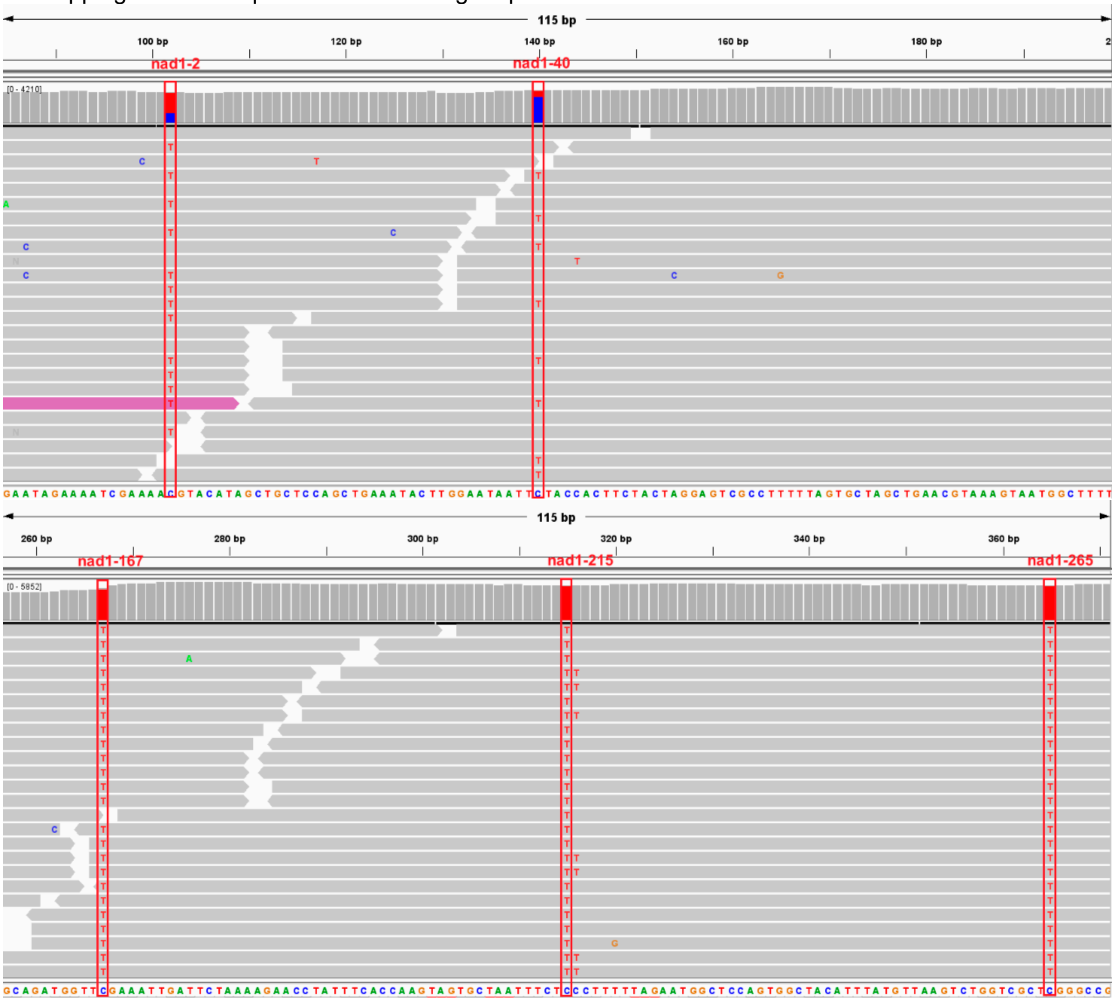

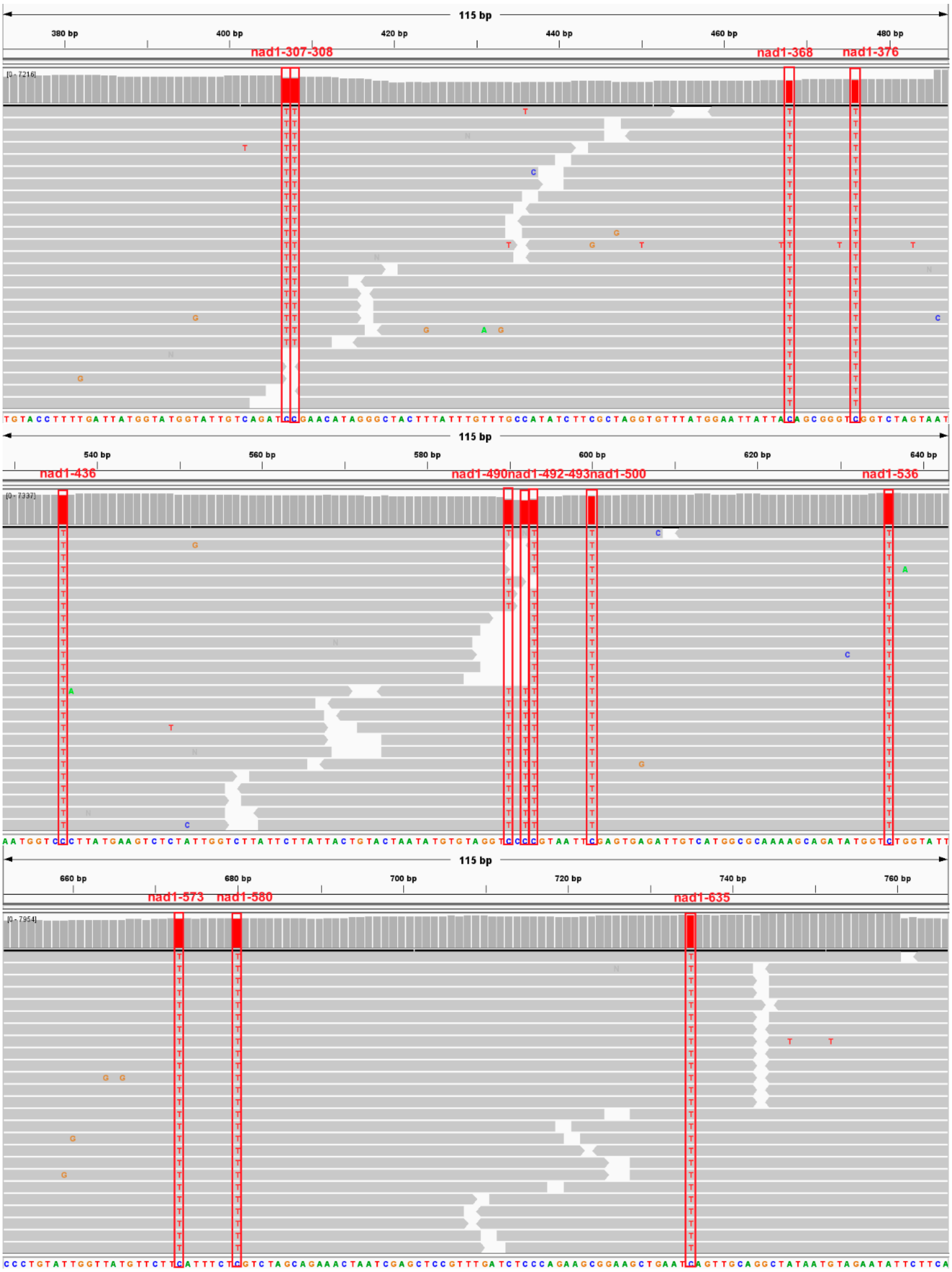
115 bp
380 bp
400 bp
420 bp
440 bp
460 bp
480 bp
nad1-307-308
nad1-368
nad1-376
115 bp
540 bp
560 bp
580 bp
600 bp
620 bp
640 bp
nad1-436
nad1-490
nad1-492-493
nad1-500
nad1-536
115 bp
660 bp
680 bp
700 bp
720 bp
740 bp
760 bp
nad1-573
nad1-580
nad1-635

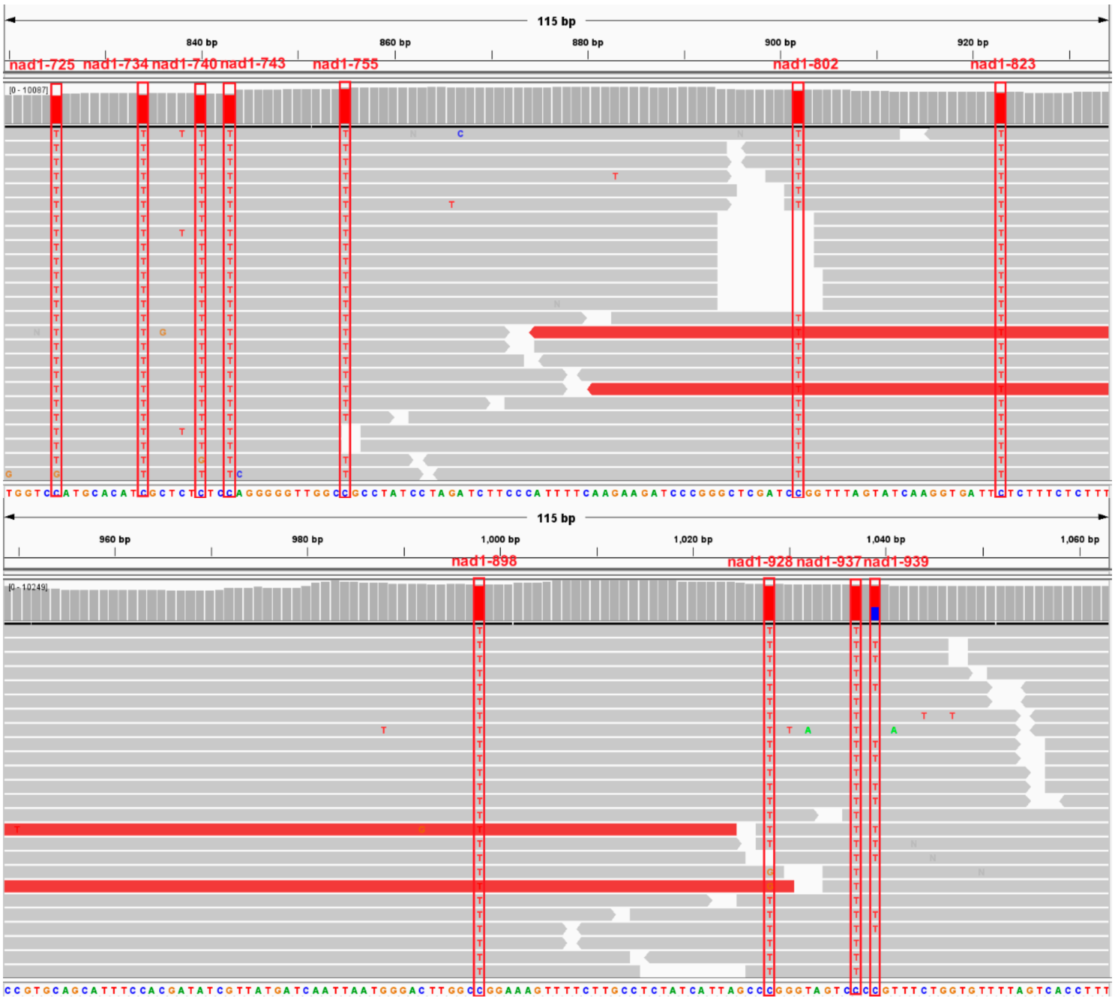

P.Mapping of RNA-seq reads to the coding sequence of *nad2*.

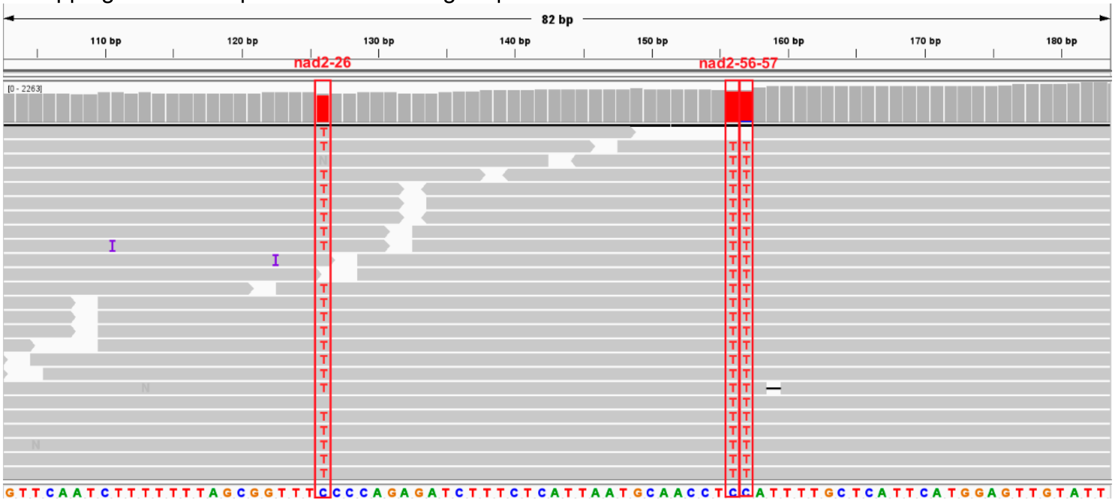

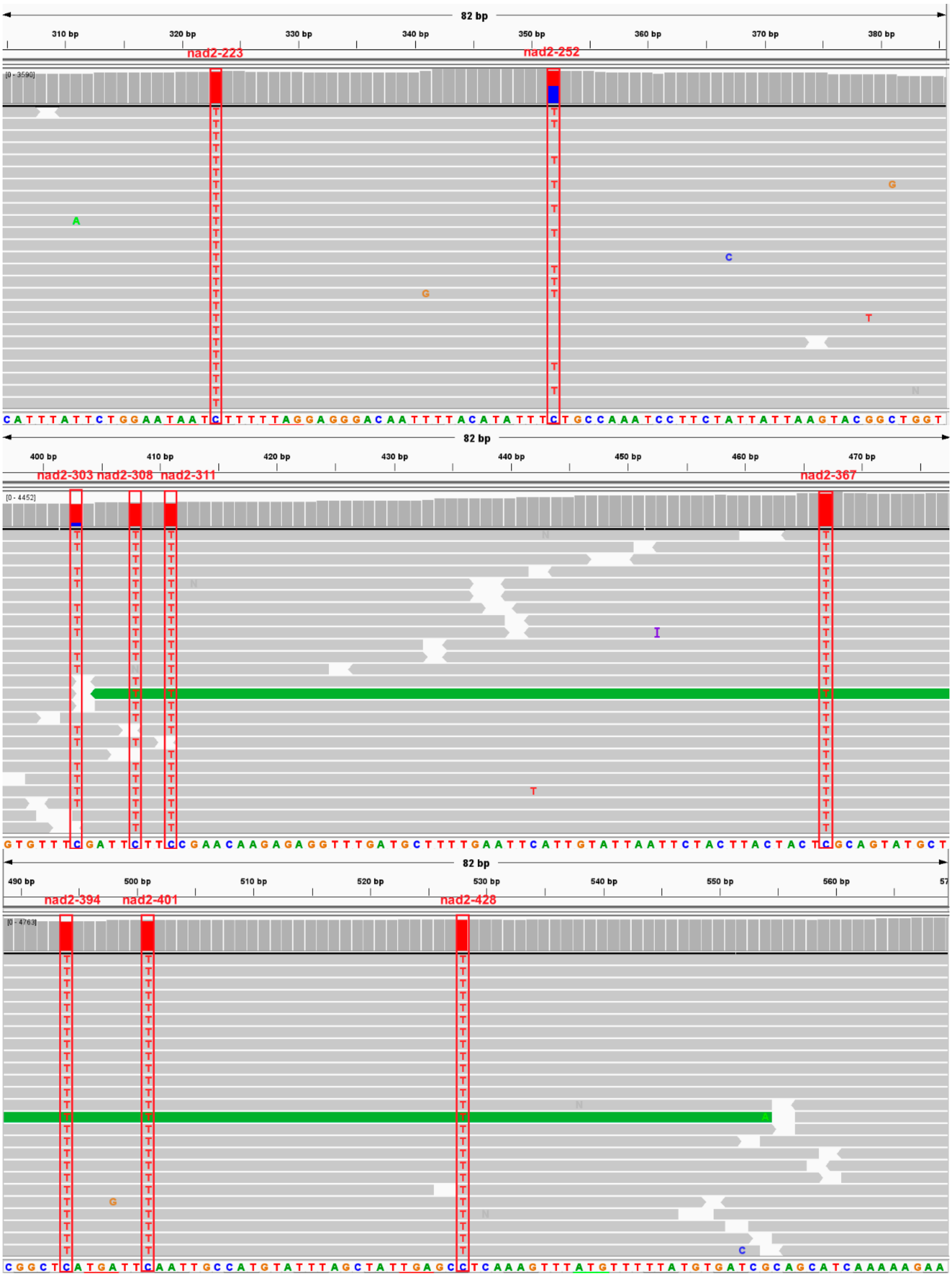
82 bp
310 bp
320 bp
330 bp
340 bp
350 bp
360 bp
370 bp
380 bp
nad2-223
nad2-252
82 bp
400 bp
410 bp
420 bp
430 bp
440 bp
450 bp
460 bp
470 bp
nad2-303
nad2-308
nad2-311
nad2-367
82 bp
490 bp
500 bp
510 bp
520 bp
530 bp
540 bp
550 bp
560 bp
nad2-394
nad2-401
nad2-428

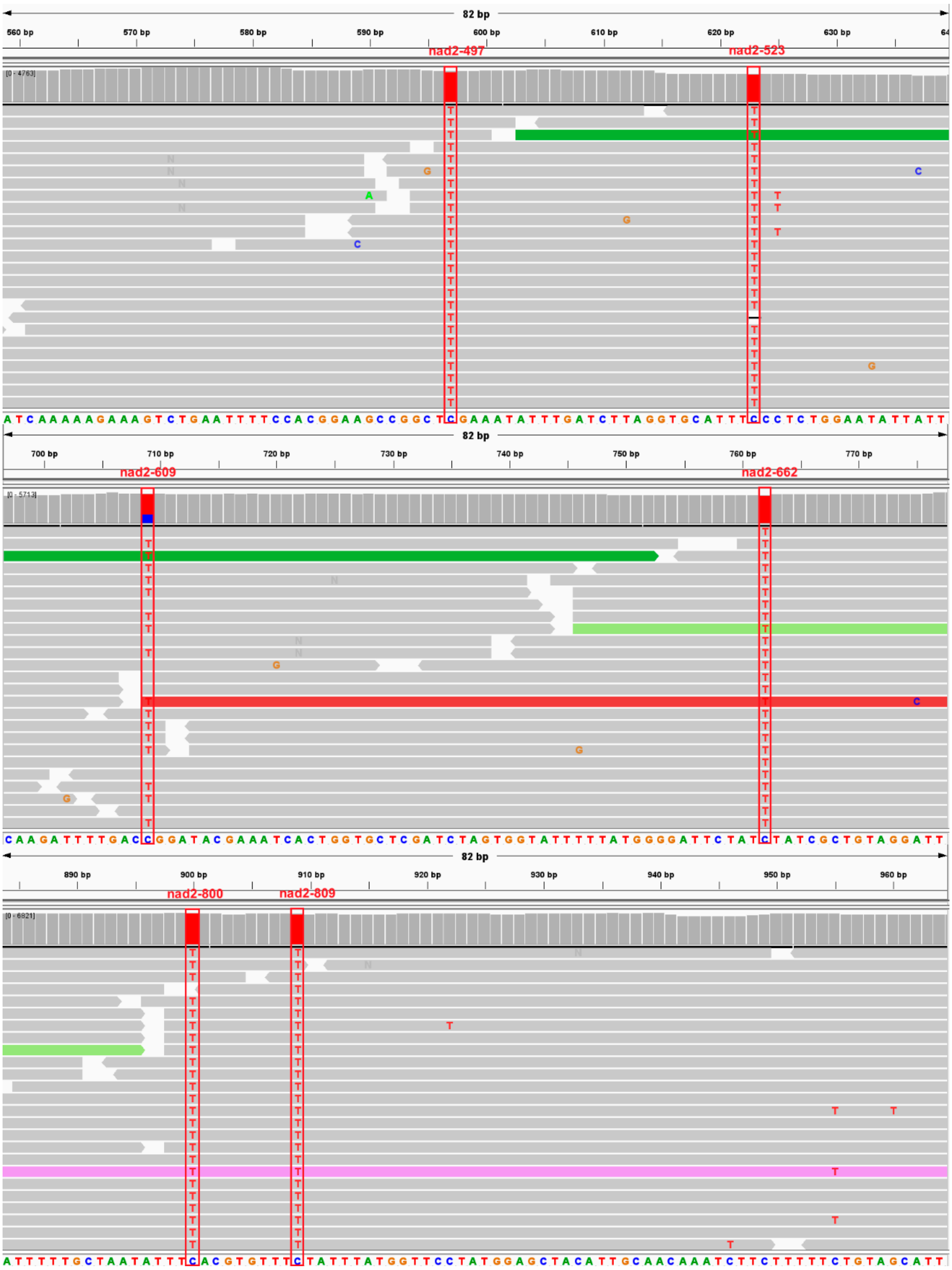
82 bp
560 bp
570 bp
580 bp
590 bp
600 bp
610 bp
620 bp
630 bp
nad2-497
nad2-523
82 bp
700 bp
710 bp
720 bp
730 bp
740 bp
750 bp
760 bp
770 bp
nad2-609
nad2-662
82 bp
890 bp
900 bp
910 bp
920 bp
930 bp
940 bp
950 bp
960 bp
nad2-800
nad2-809

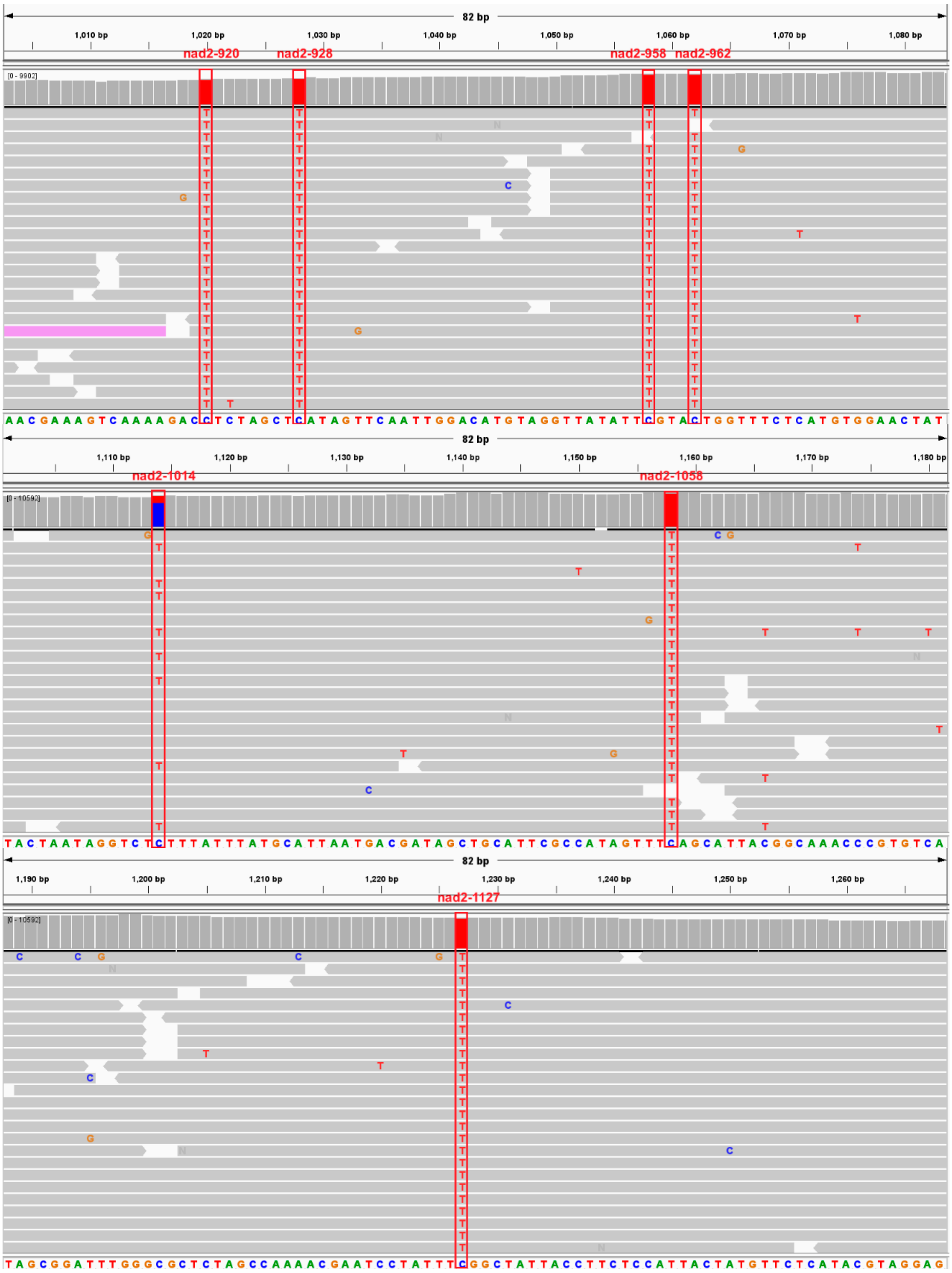
82 bp
1,010 bp
1,020 bp
1,030 bp
1,040 bp
1,050 bp
1,060 bp
1,070 bp
1,080 bp
nad2-920
nad2-928
nad2-958
nad2-962
[0 - 9902]
82 bp
1,110 bp
1,120 bp
1,130 bp
1,140 bp
1,150 bp
1,160 bp
1,170 bp
1,180 bp
nad2-1014
nad2-1058
[0 - 10592]
82 bp
1,190 bp
1,200 bp
1,210 bp
1,220 bp
1,230 bp
1,240 bp
1,250 bp
1,260 bp
nad2-1127
[0 - 10592]

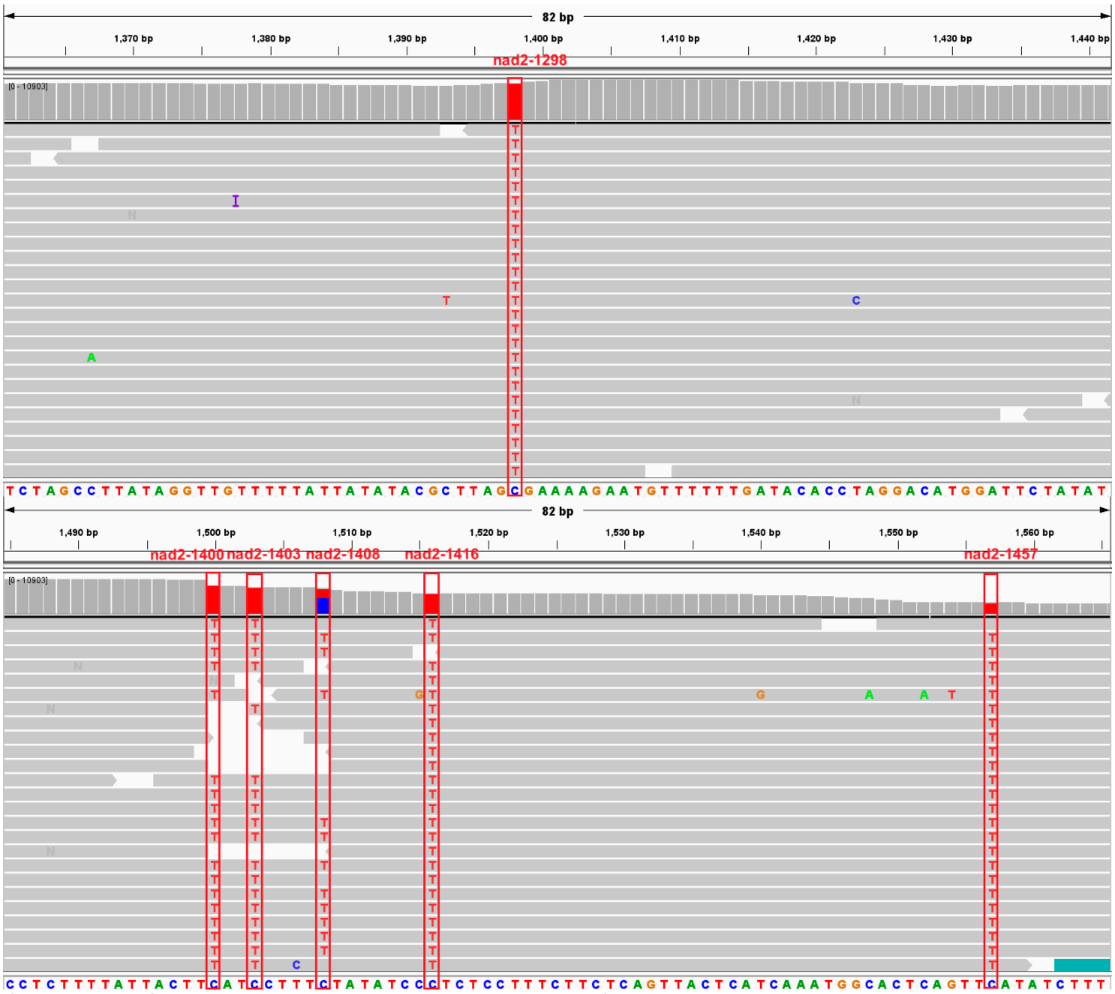

Q. Mapping of RNA-seq reads to the coding sequence of *nad3*.

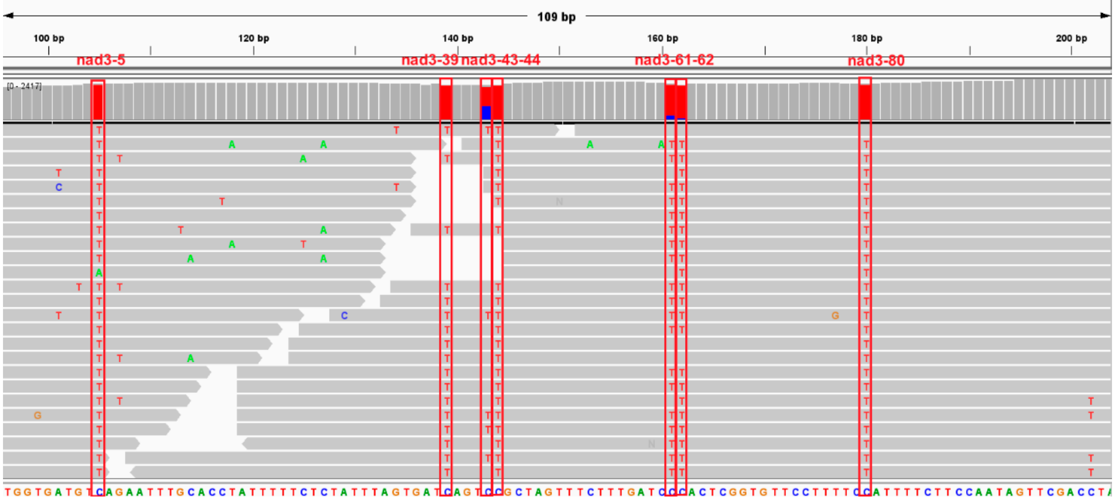

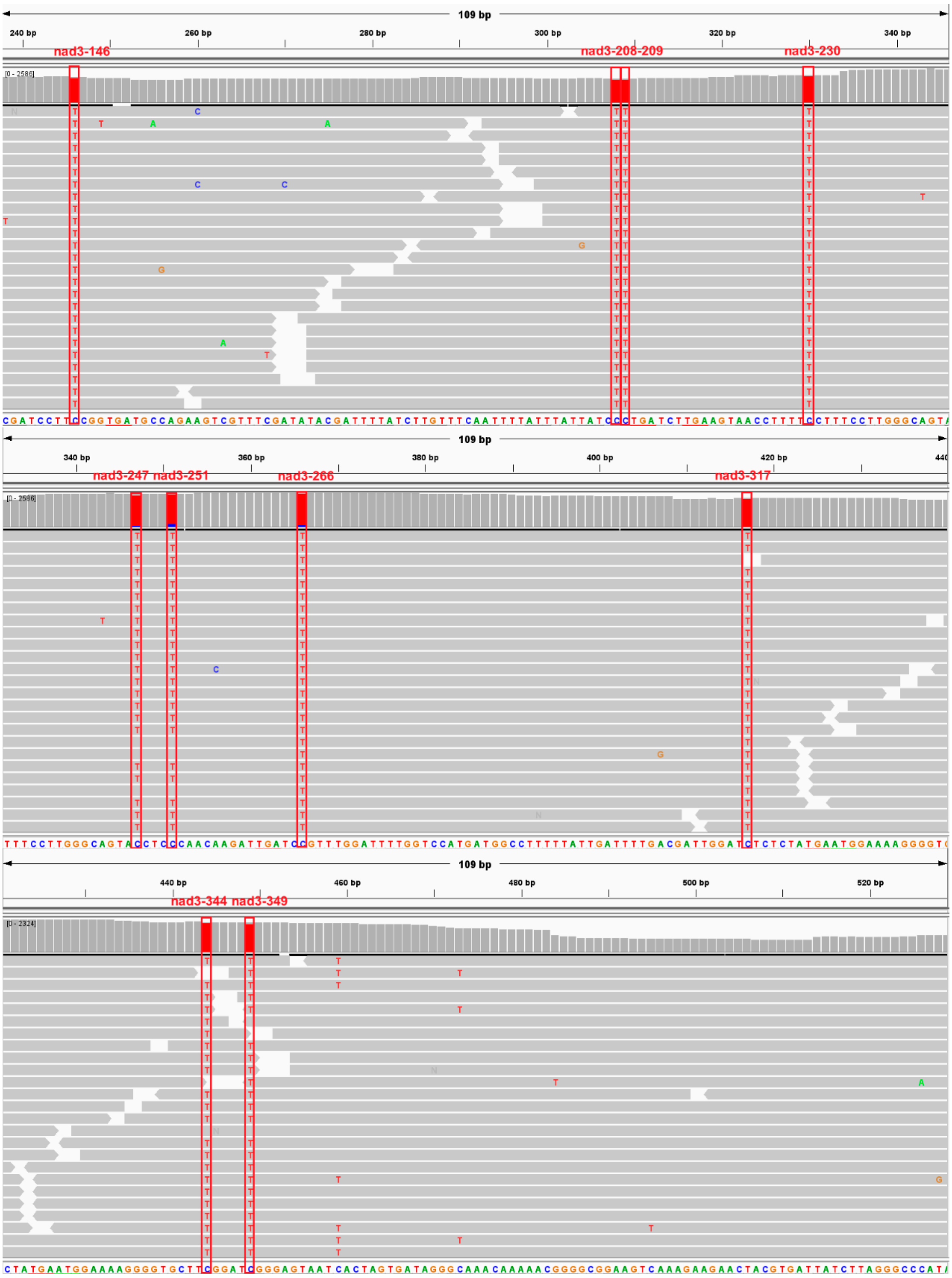

R.Mapping of RNA-seq reads to the coding sequence of *nad4*.

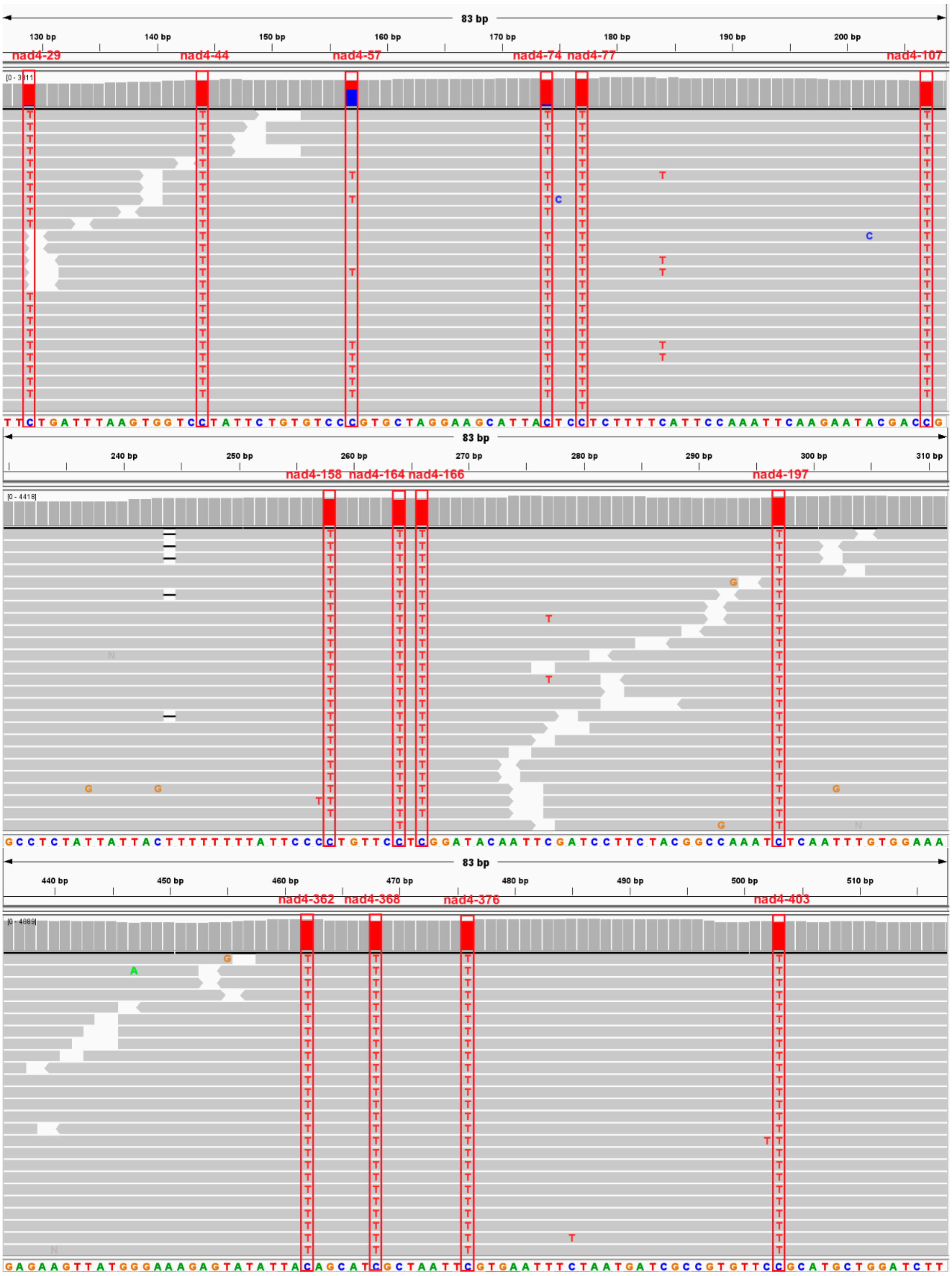
83 bp
130 bp
140 bp
150 bp
160 bp
170 bp
180 bp
190 bp
200 bp
nad4-29
nad4-44
nad4-57
nad4-74
nad4-77
nad4-107
83 bp
240 bp
250 bp
260 bp
270 bp
280 bp
290 bp
300 bp
310 bp
nad4-158
nad4-164
nad4-166
nad4-197
83 bp
440 bp
450 bp
460 bp
470 bp
480 bp
490 bp
500 bp
510 bp
nad4-362
nad4-368
nad4-376
nad4-403

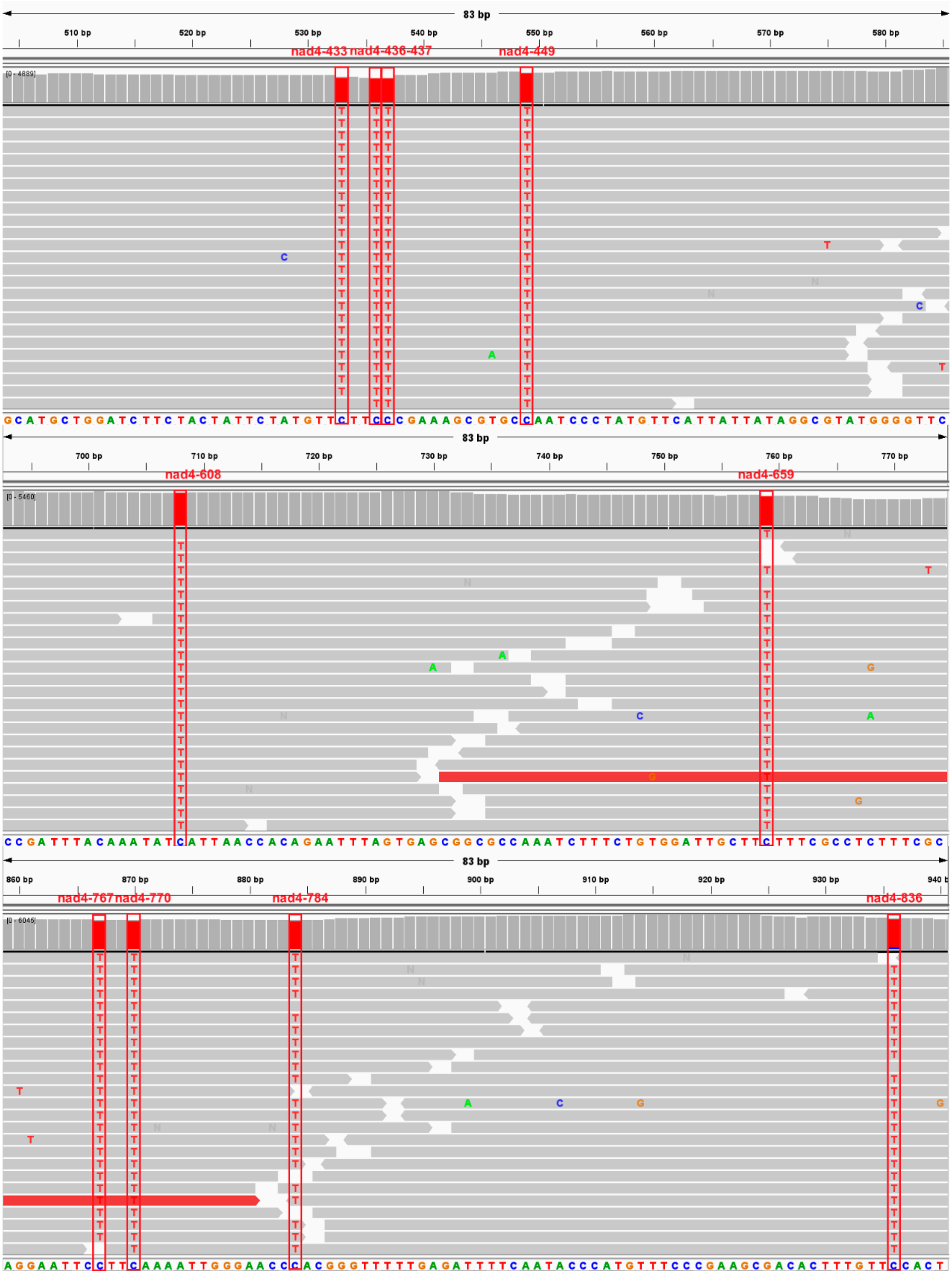

83 bp
510 bp
520 bp
530 bp
540 bp
550 bp
560 bp
570 bp
580 bp
nad4-433
nad4-436-437
nad4-449
83 bp
700 bp
710 bp
720 bp
730 bp
740 bp
750 bp
760 bp
770 bp
nad4-608
nad4-659
83 bp
860 bp
870 bp
880 bp
890 bp
900 bp
910 bp
920 bp
930 bp
nad4-767
nad4-770
nad4-784
nad4-836

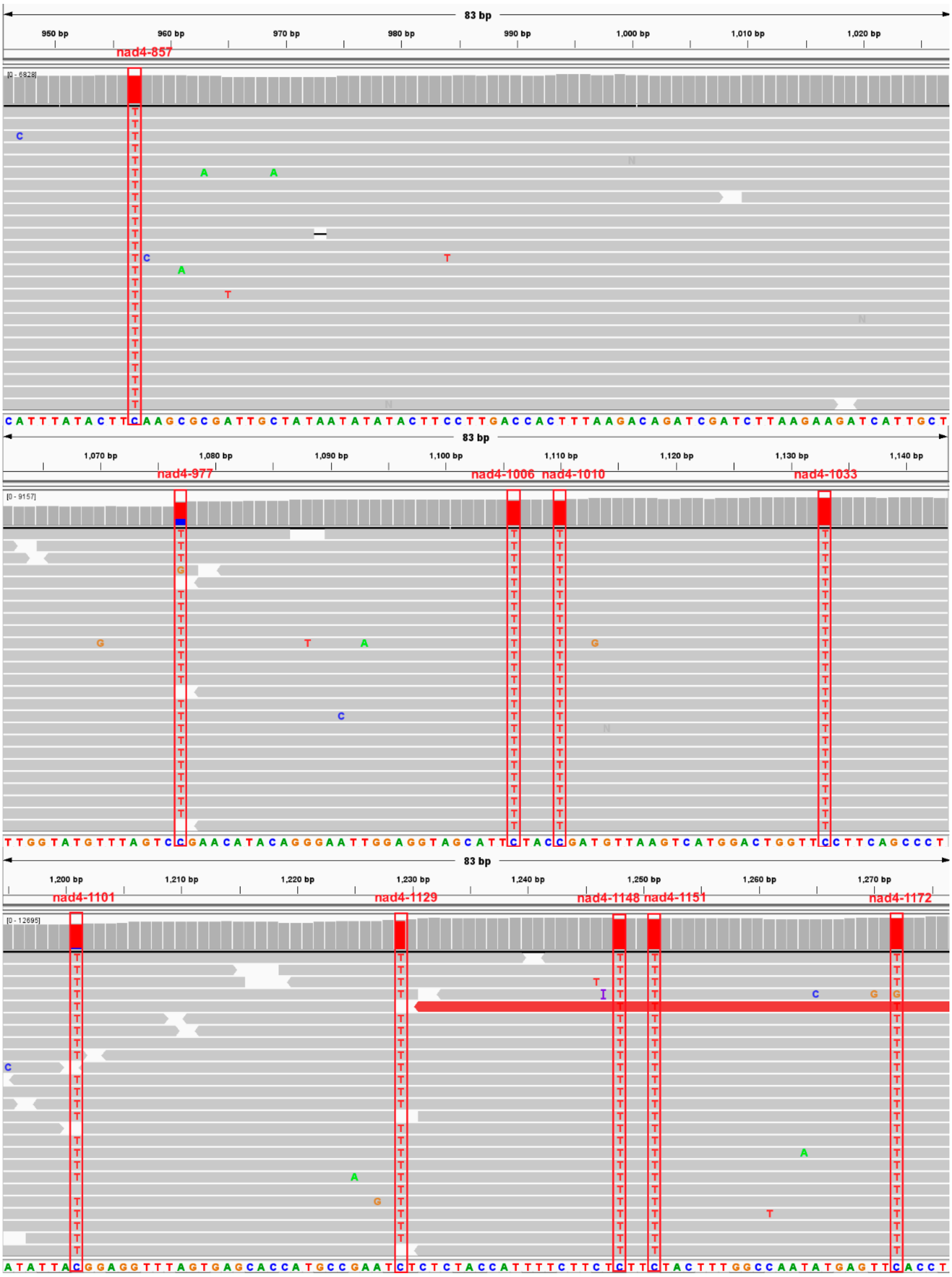

83 bp
nad4-857
nad4-977
nad4-1006
nad4-1010
nad4-1033
nad4-1101
nad4-1129
nad4-1148
nad4-1151
nad4-1172

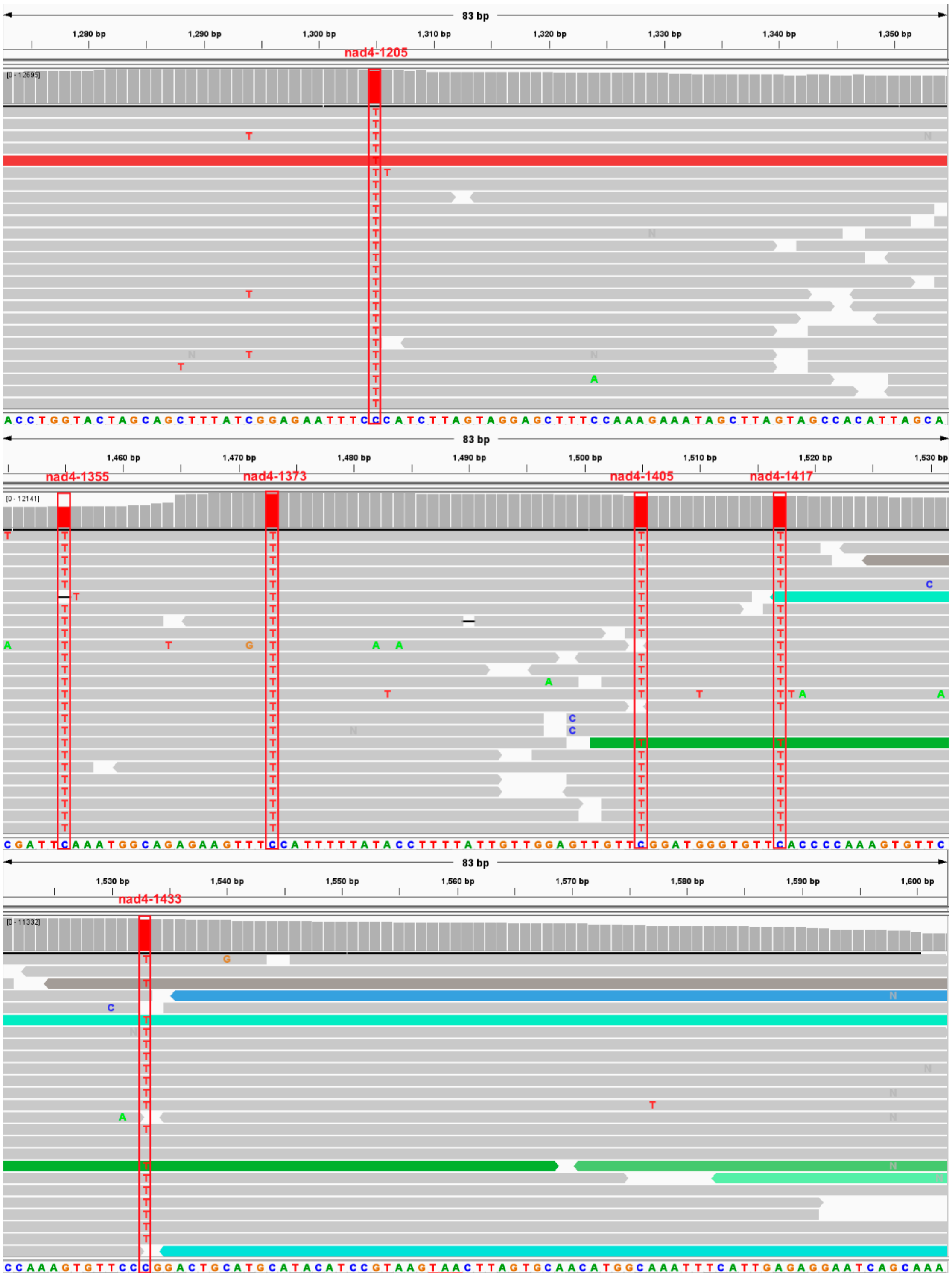
83 bp
1,280 bp
1,290 bp
1,300 bp
1,310 bp
1,320 bp
1,330 bp
1,340 bp
1,350 bp
nad4-1205
[0 - 12695]
83 bp
1,460 bp
1,470 bp
1,480 bp
1,490 bp
1,500 bp
1,510 bp
1,520 bp
1,530 bp
nad4-1355
nad4-1373
nad4-1405
nad4-1417
[0 - 12141]
83 bp
1,530 bp
1,540 bp
1,550 bp
1,560 bp
1,570 bp
1,580 bp
1,590 bp
1,600 bp
nad4-1433
[0 - 11332]

S. Mapping of RNA-seq reads to the coding sequence of *nad4L*.

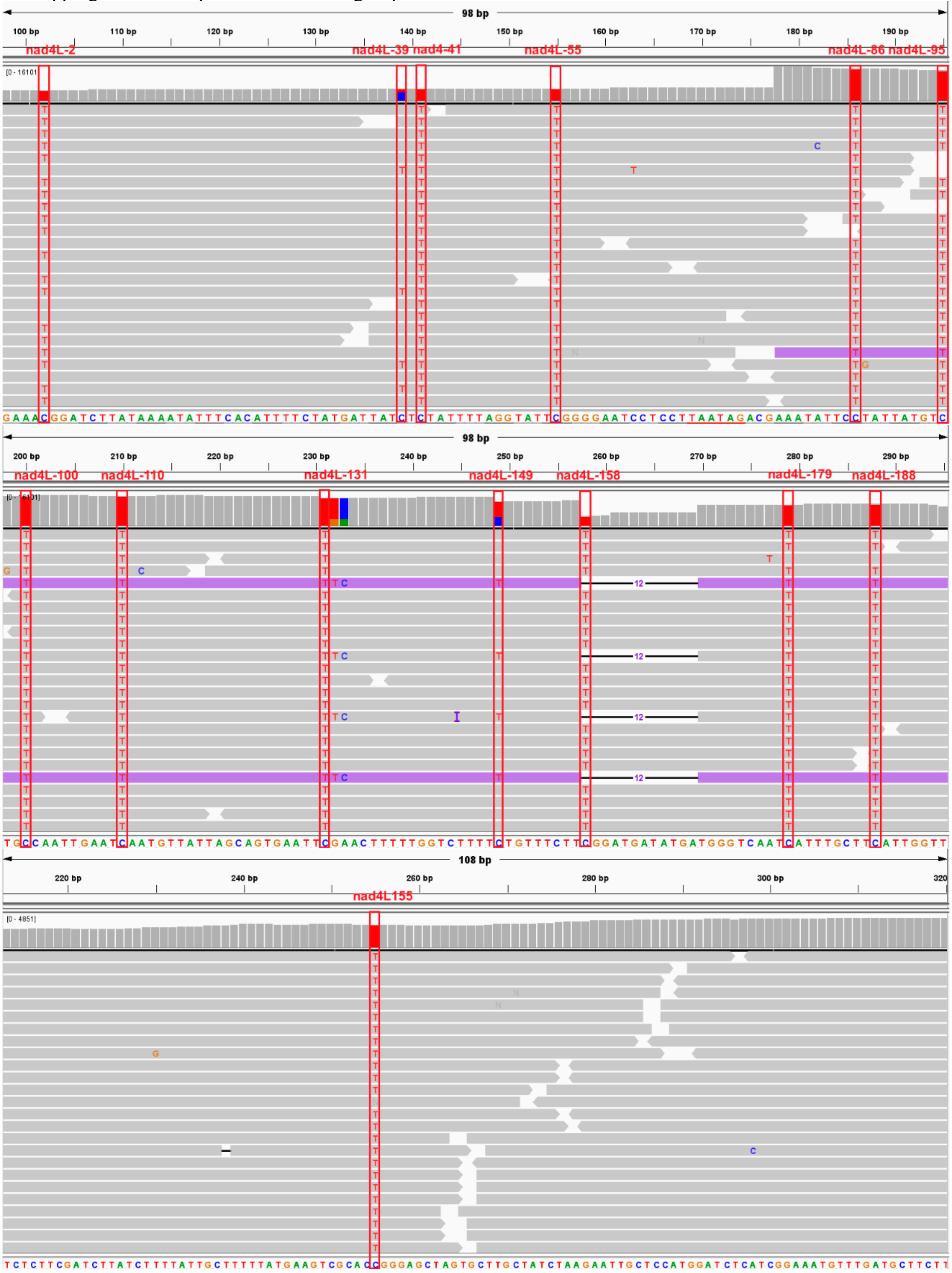

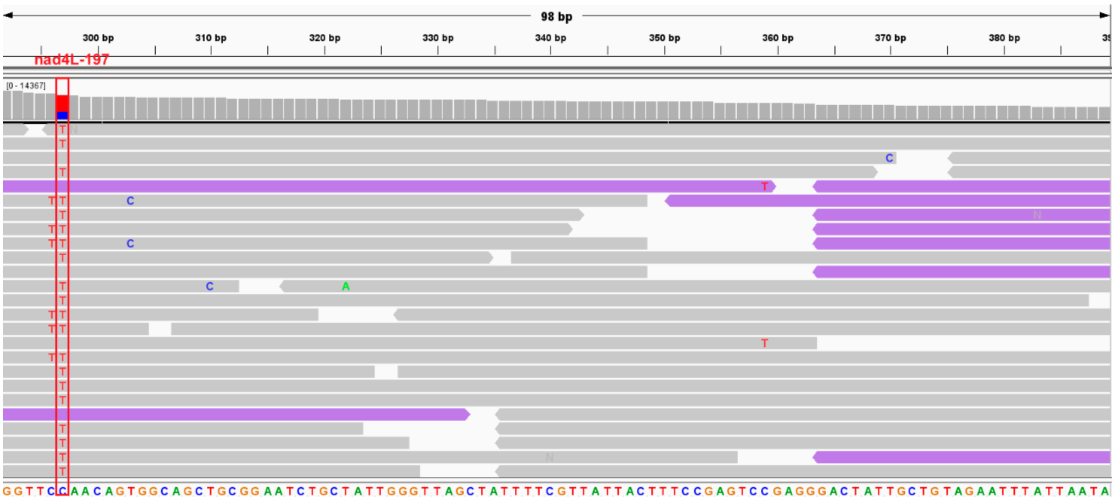
98 bp
300 bp
310 bp
320 bp
330 bp
340 bp
350 bp
360 bp
370 bp
380 bp
nad4L-197
[0 - 14367]

T.Mapping of RNA-seq reads to the coding sequence of *nad5*.

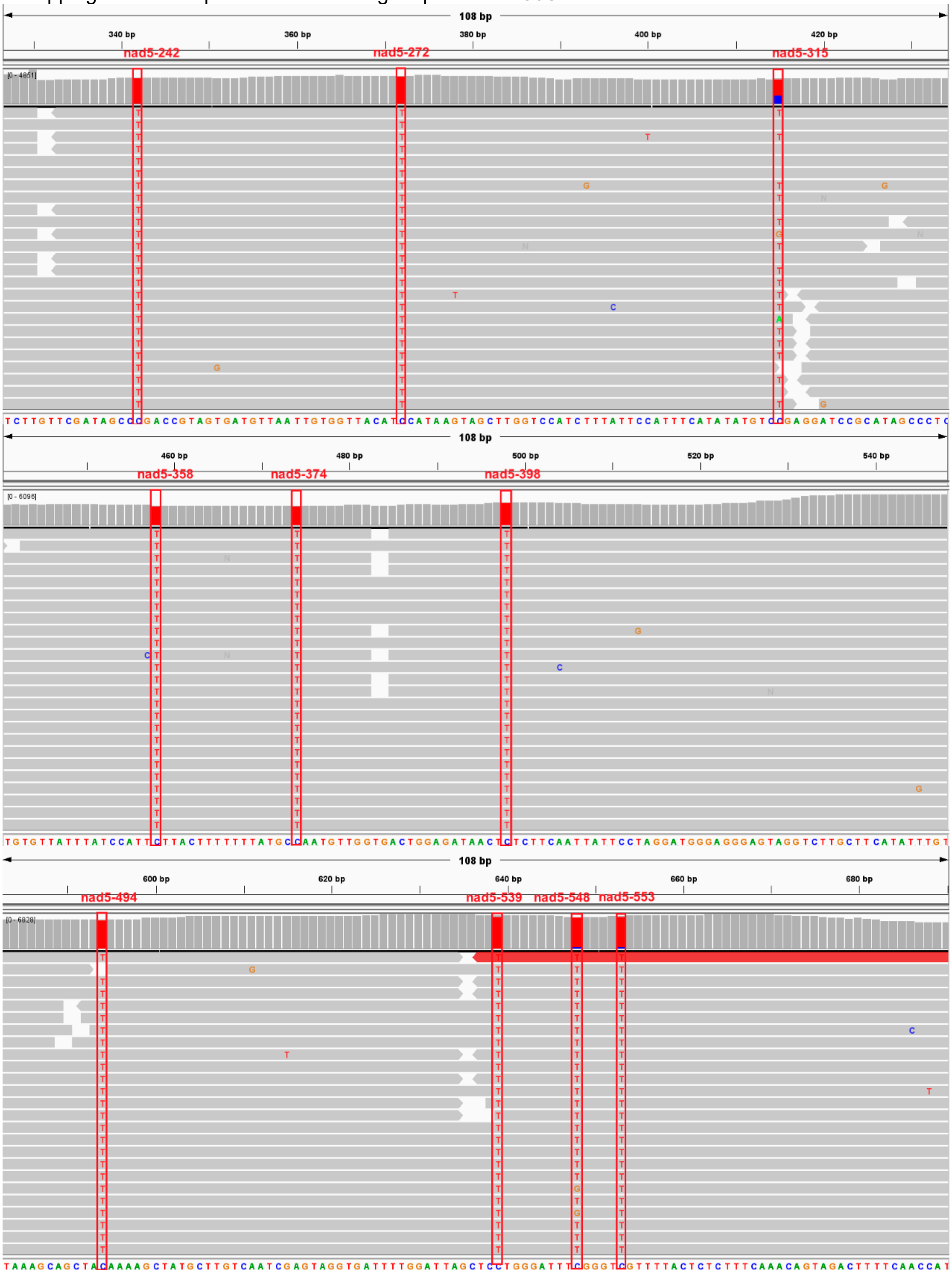

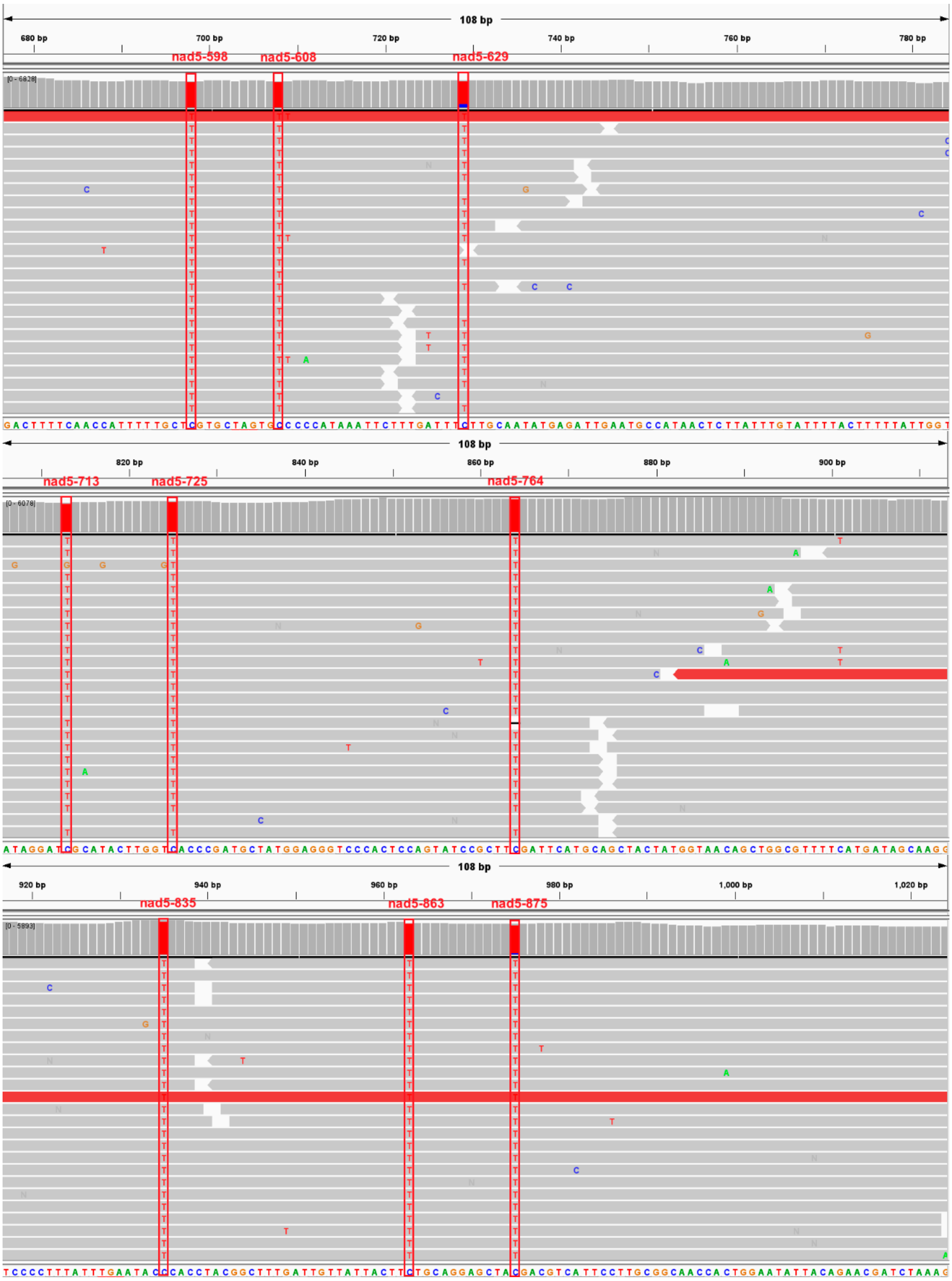
108 bp
680 bp
700 bp
720 bp
740 bp
760 bp
780 bp
nad5-598
nad5-608
nad5-629
108 bp
820 bp
840 bp
860 bp
880 bp
900 bp
nad5-713
nad5-725
nad5-764
108 bp
920 bp
940 bp
960 bp
980 bp
1,000 bp
1,020 bp
nad5-835
nad5-863
nad5-875

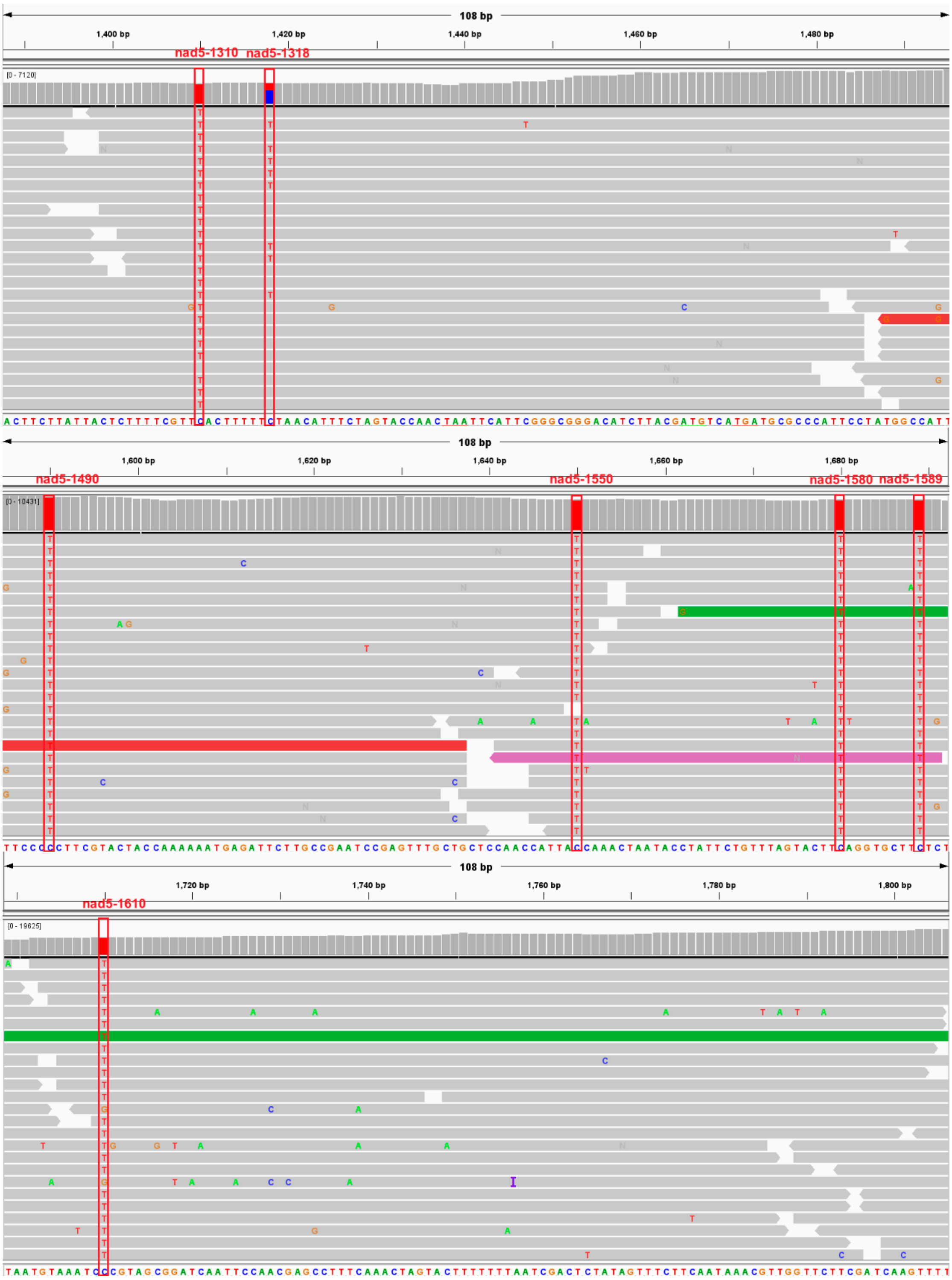
108 bp
1,400 bp
1,420 bp
1,440 bp
1,460 bp
1,480 bp
nad5-1310
nad5-1318
[0 - 7120]
108 bp
1,600 bp
1,620 bp
1,640 bp
1,660 bp
1,680 bp
nad5-1490
nad5-1550
nad5-1580
nad5-1589
[0 - 10431]
108 bp
1,720 bp
1,740 bp
1,760 bp
1,780 bp
1,800 bp
nad5-1610
[0 - 19625]

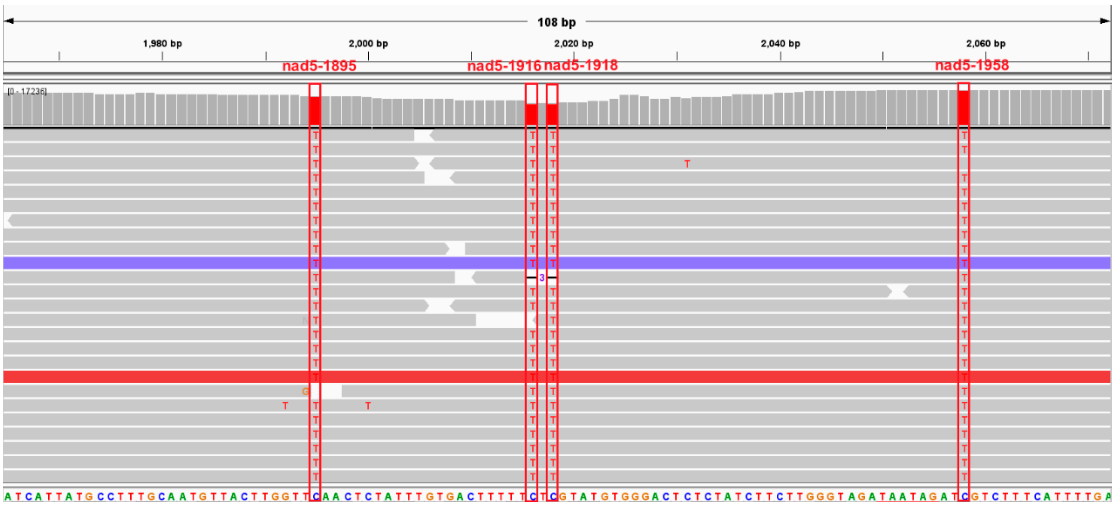

U.Mapping of RNA-seq reads to the coding sequence of *nad6*.

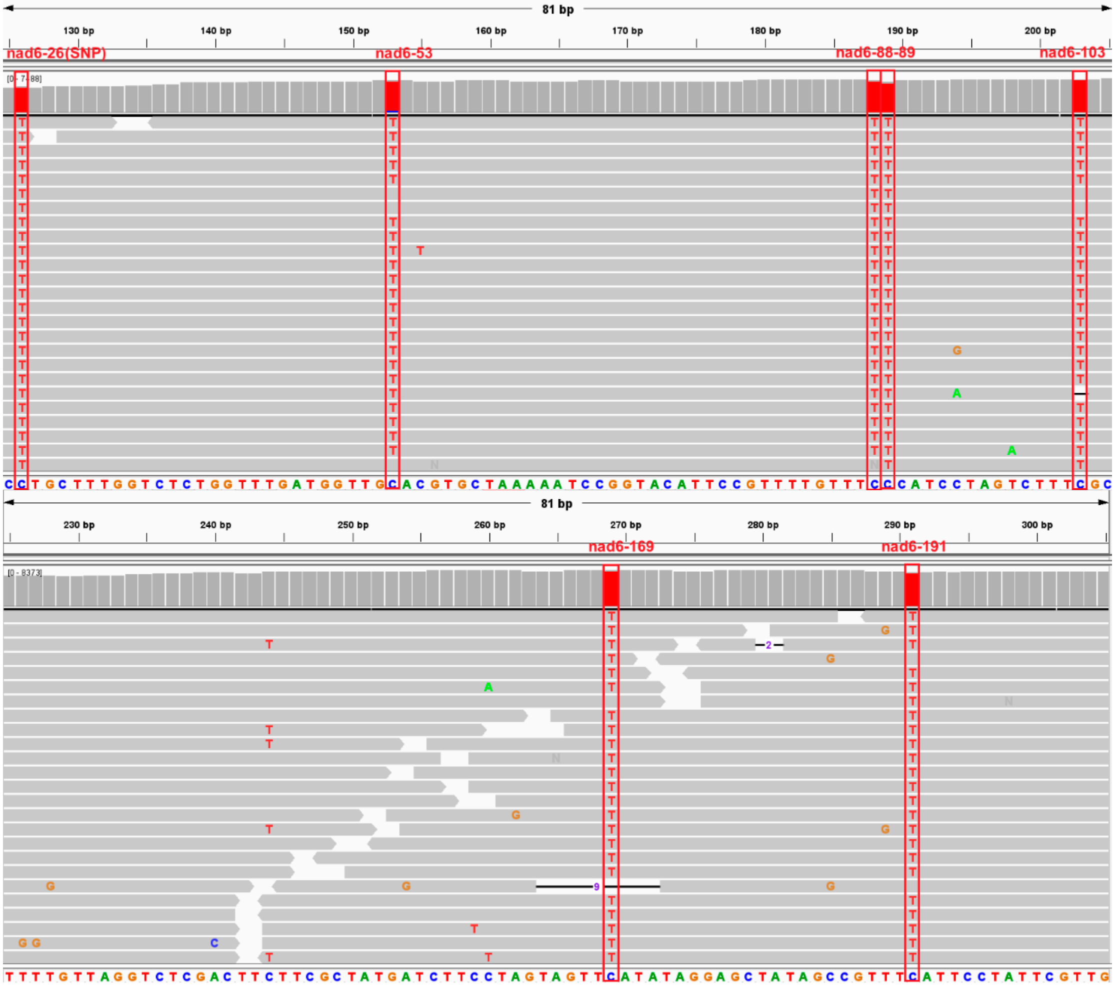

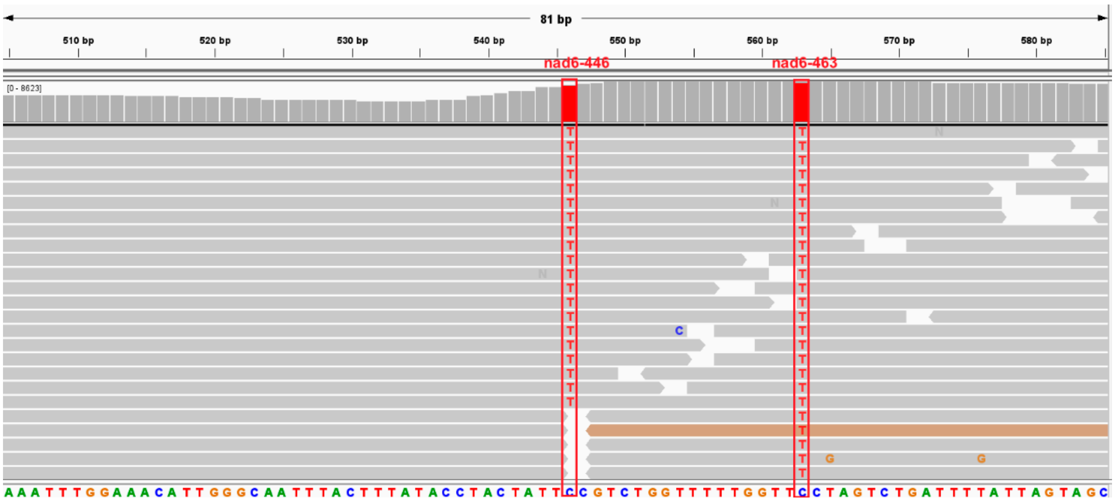

V. Mapping of RNA-seq reads to the coding sequence of *nad7*.

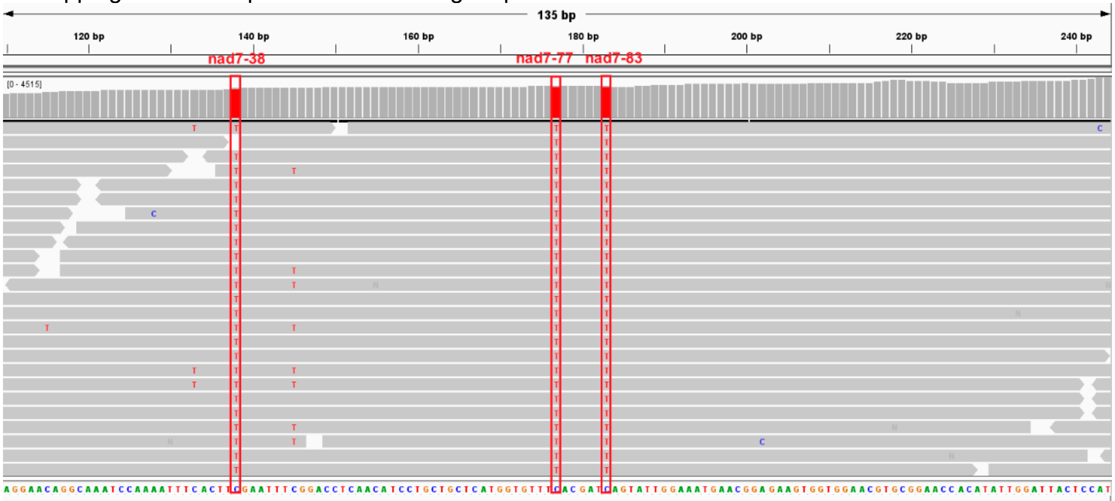

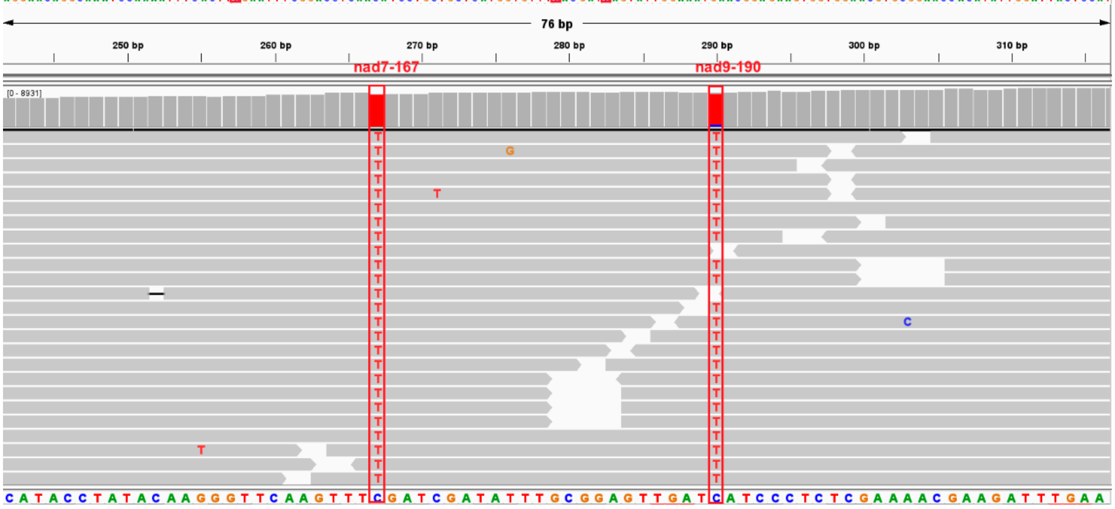

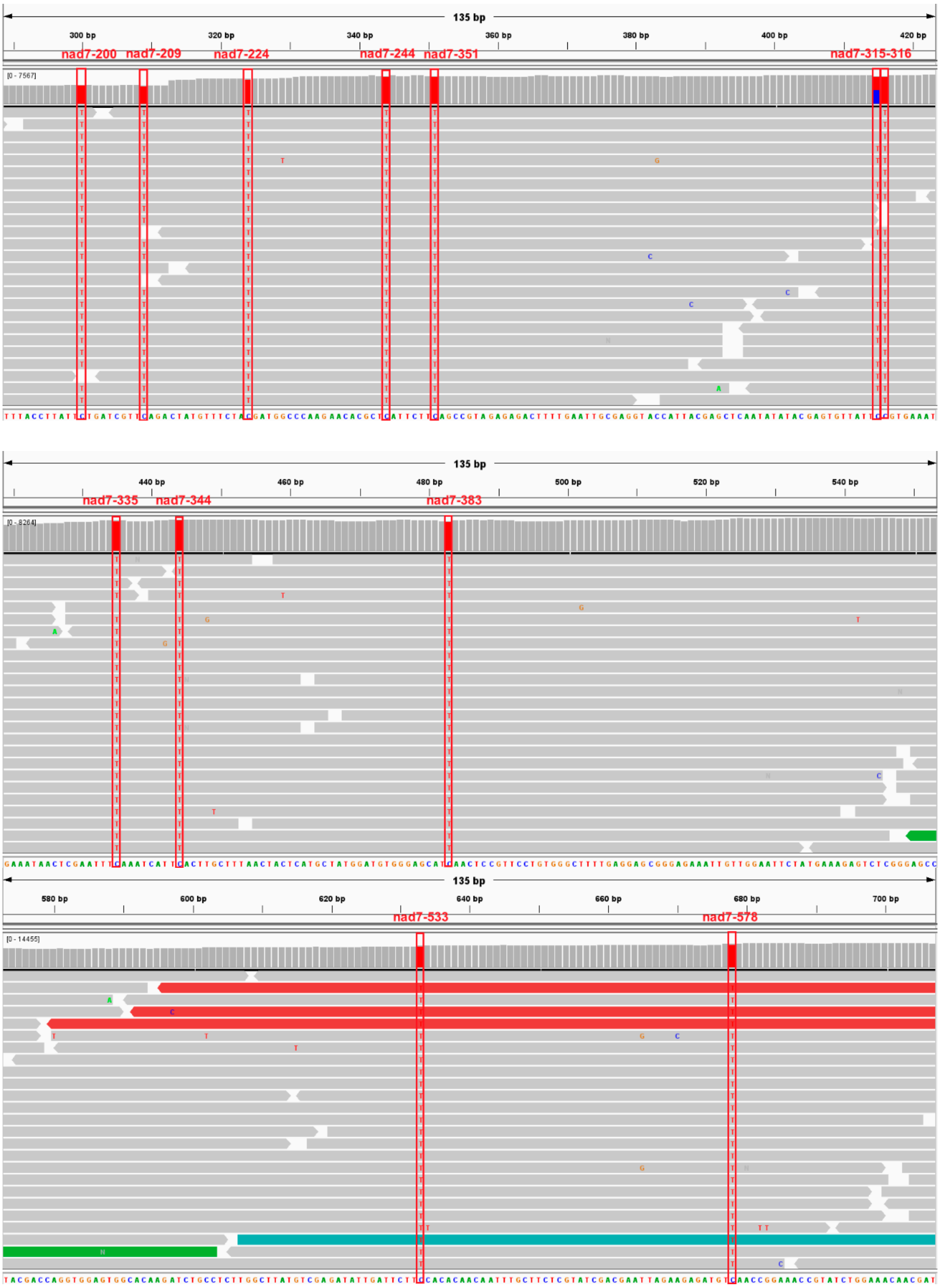
135 bp
300 bp
320 bp
340 bp
360 bp
380 bp
400 bp
420 bp
nad7-200
nad7-209
nad7-224
nad7-244
nad7-351
nad7-315-316
[0 - 7567]
135 bp
440 bp
460 bp
480 bp
500 bp
520 bp
540 bp
nad7-335
nad7-344
nad7-383
[0 - 8264]
135 bp
580 bp
600 bp
620 bp
640 bp
660 bp
680 bp
700 bp
nad7-533
nad7-578
[0 - 14455]

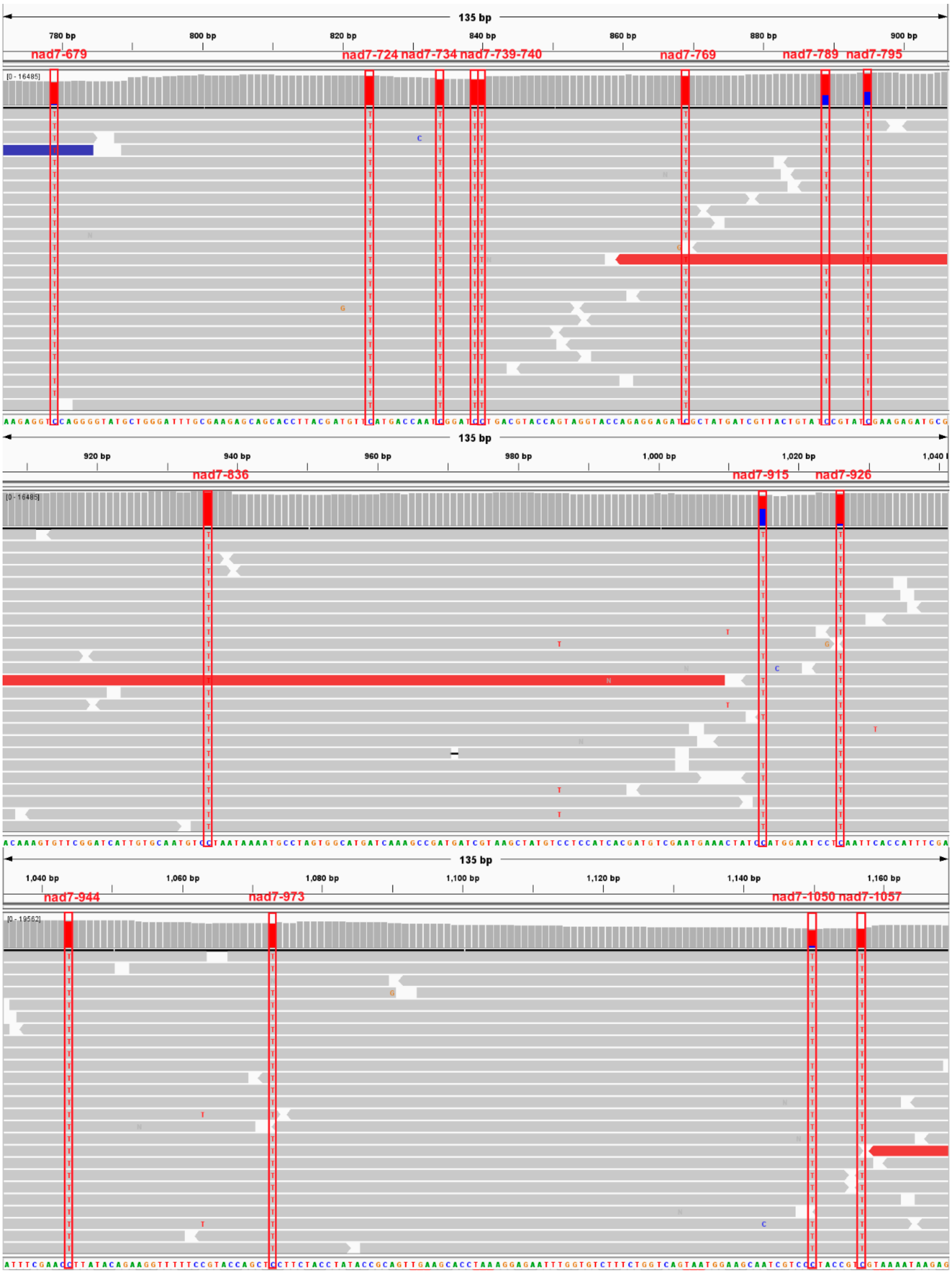
135 bp
780 bp
800 bp
820 bp
840 bp
860 bp
880 bp
900 bp
nad7-679
nad7-724
nad7-734
nad7-739-740
nad7-769
nad7-789
nad7-795
[0 - 16485]
135 bp
920 bp
940 bp
960 bp
980 bp
1,000 bp
1,020 bp
nad7-836
nad7-915
nad7-926
[0 - 16485]
135 bp
1,040 bp
1,060 bp
1,080 bp
1,100 bp
1,120 bp
1,140 bp
1,160 bp
nad7-944
nad7-973
nad7-1050
nad7-1057
[0 - 19562]

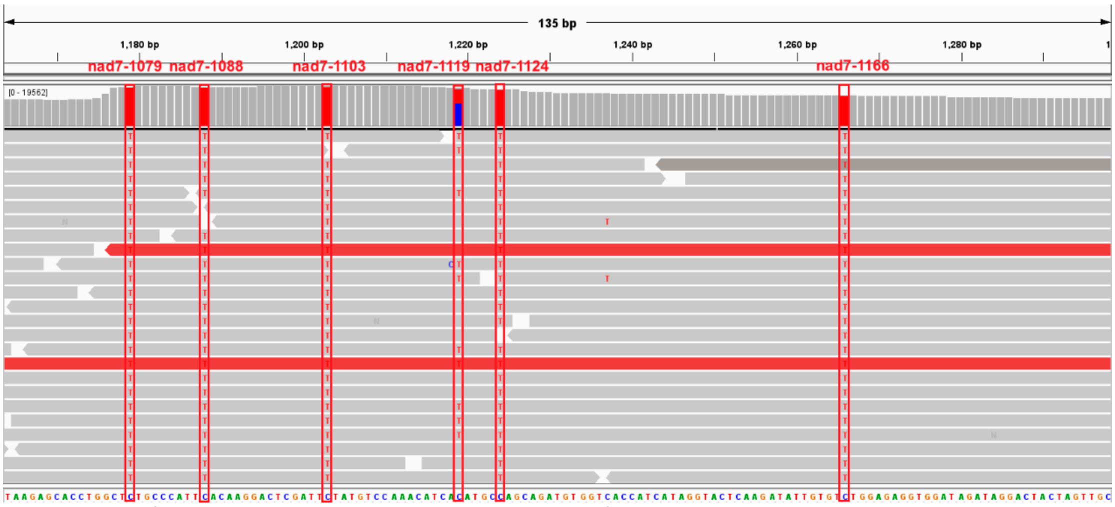

W. Mapping of RNA-seq reads to the coding sequence of *nad9*.

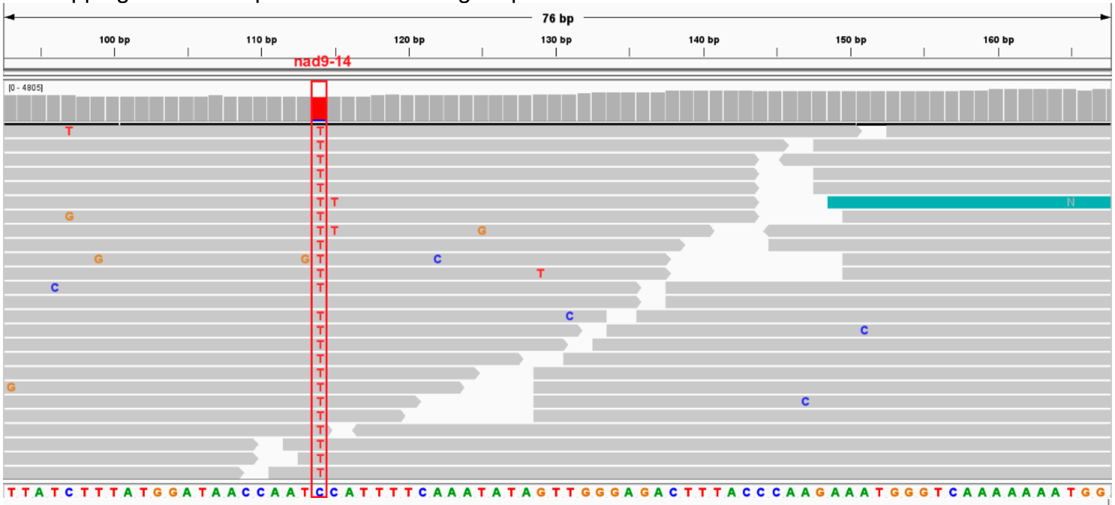

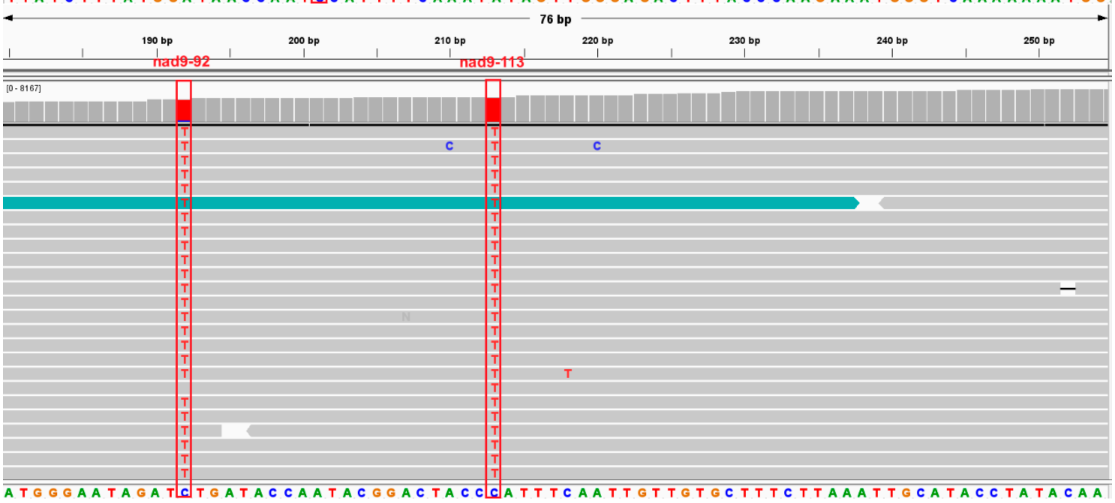

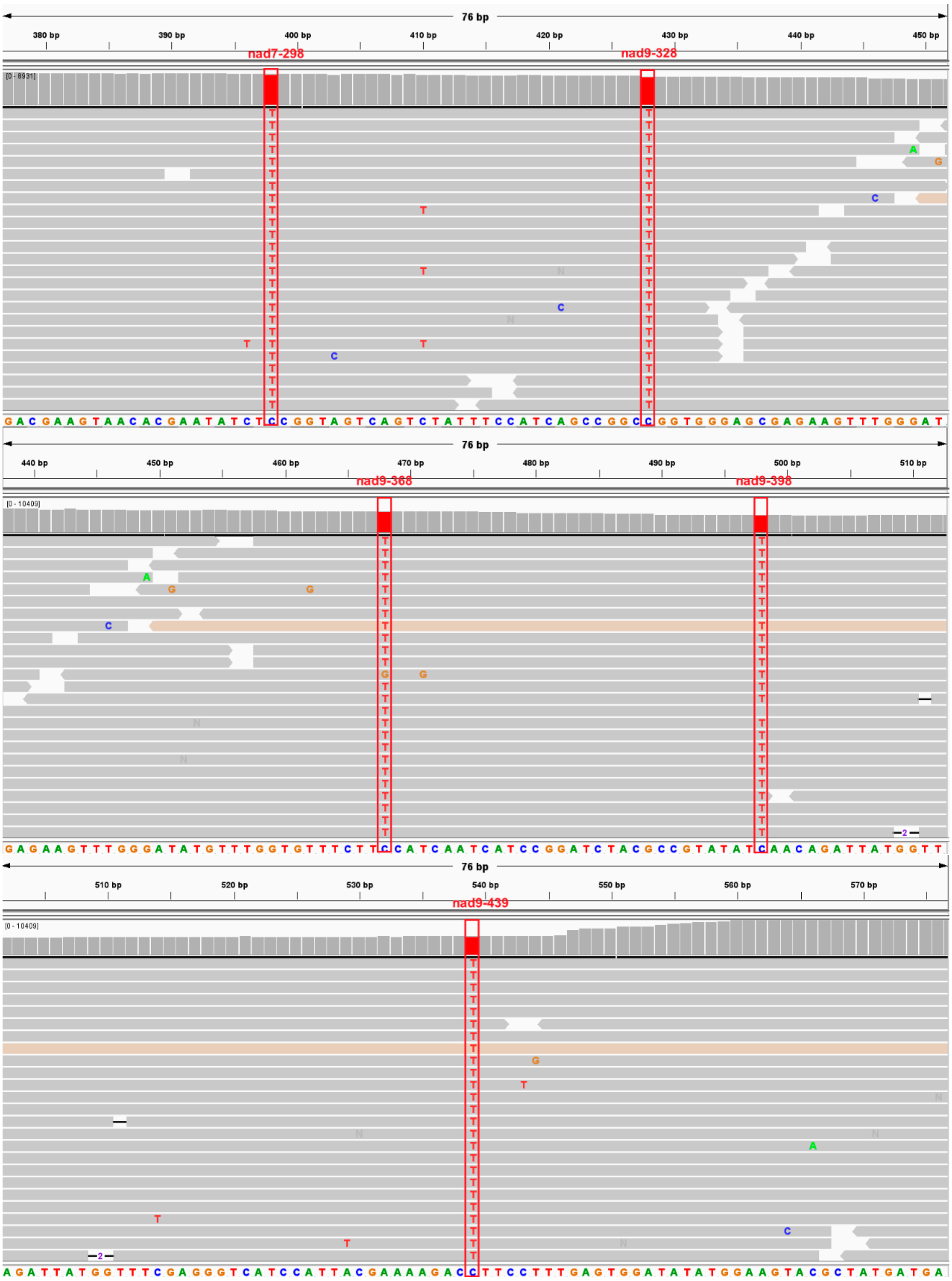

76 bp
380 bp
390 bp
400 bp
410 bp
420 bp
430 bp
440 bp
450 bp
nad7-298
nad9-328
[0 - 8931]
76 bp
440 bp
450 bp
460 bp
470 bp
480 bp
490 bp
500 bp
510 bp
nad9-368
nad9-398
[0 - 10409]
76 bp
510 bp
520 bp
530 bp
540 bp
550 bp
560 bp
570 bp
nad9-439
[0 - 10409]

X. Mapping of RNA-seq reads to the coding sequence of *rpl2*.

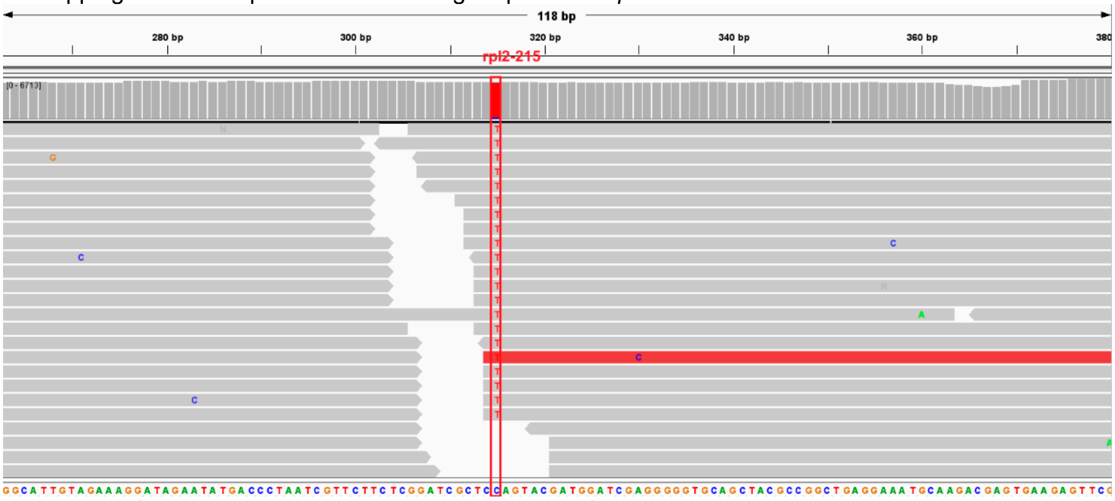

Y.Mapping of RNA-seq reads to the coding sequence of *rpl5*.

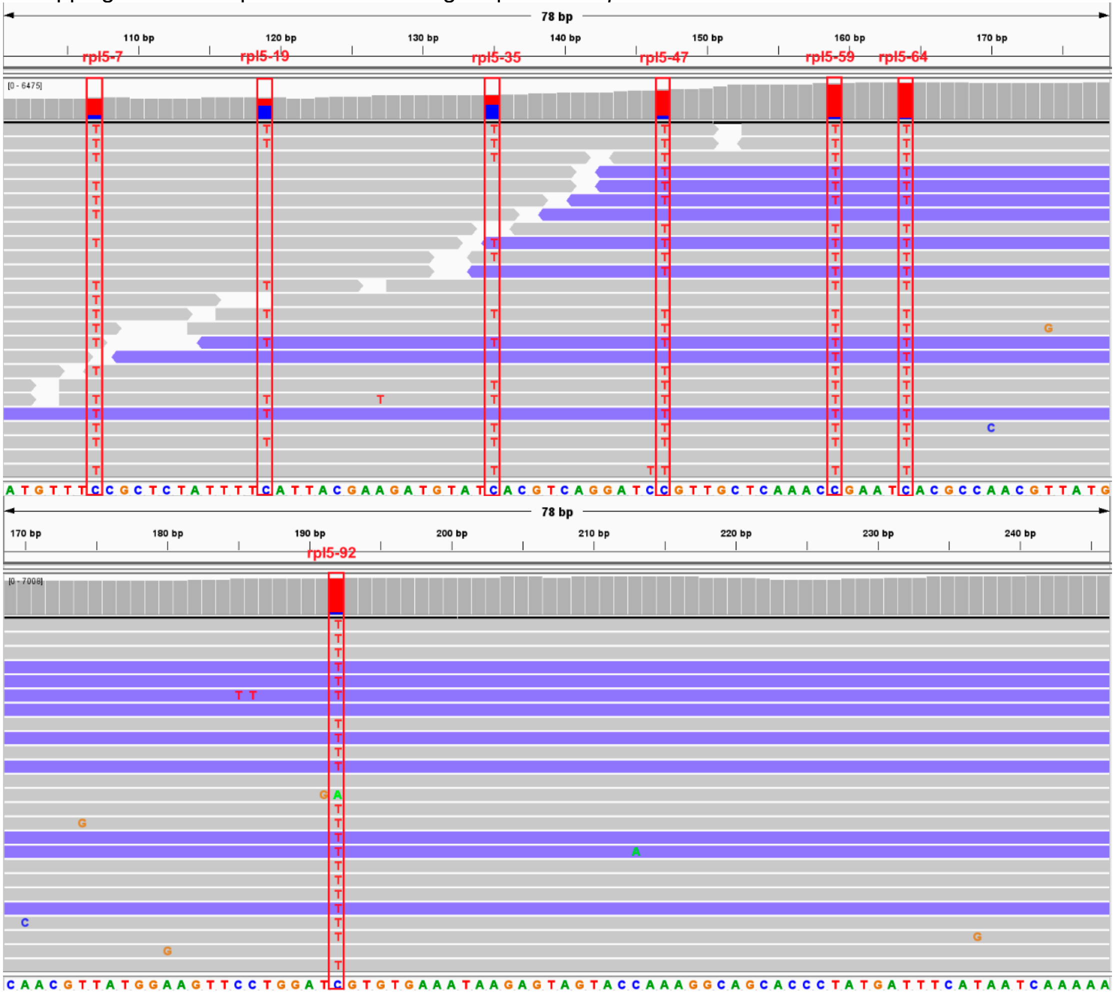

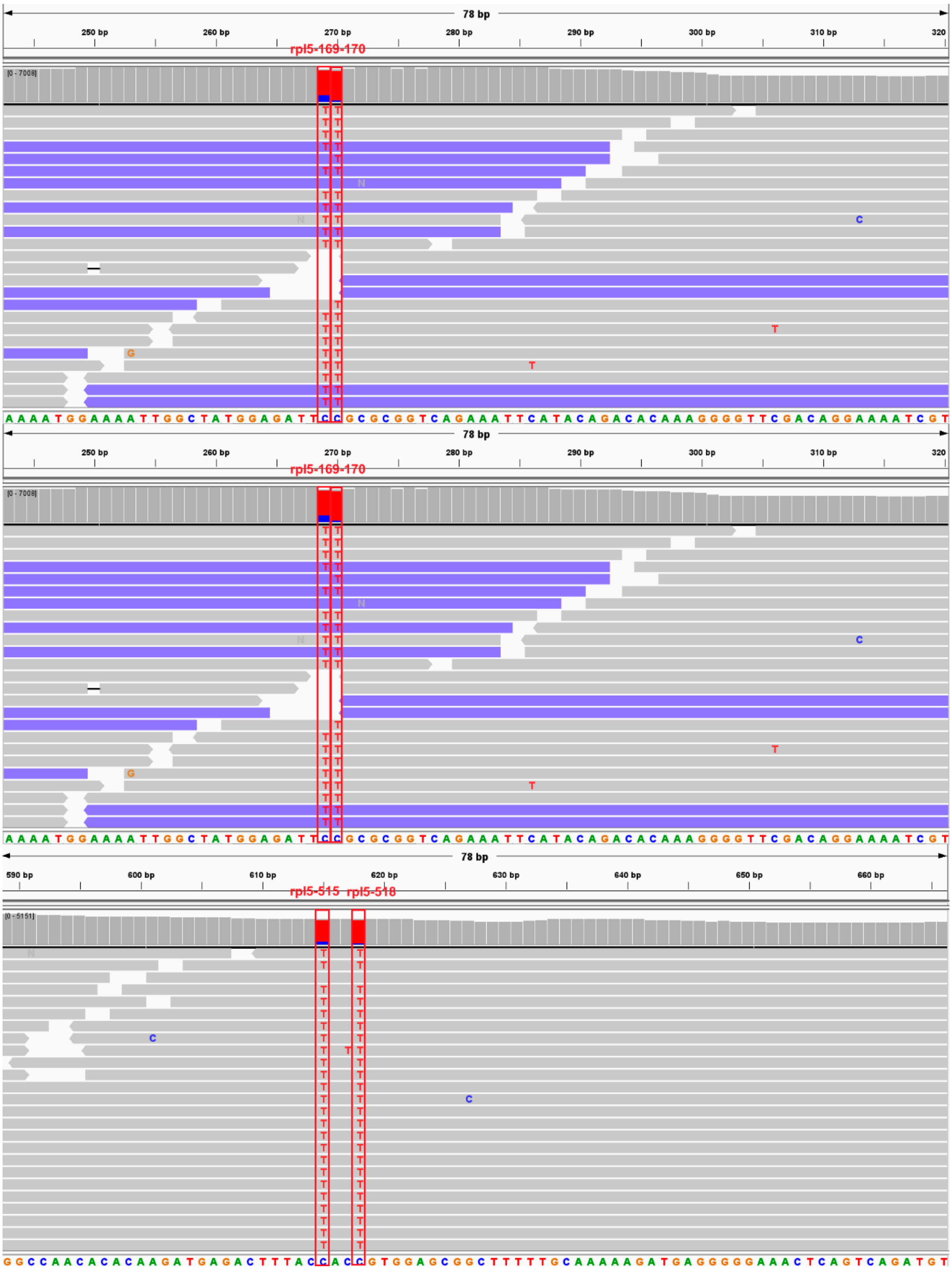
78 bp
250 bp
260 bp
270 bp
280 bp
290 bp
300 bp
310 bp
320
rpl5-169-170
[0 - 7008]
78 bp
250 bp
260 bp
270 bp
280 bp
290 bp
300 bp
310 bp
320
rpl5-169-170
[0 - 7008]
78 bp
590 bp
600 bp
610 bp
620 bp
630 bp
640 bp
650 bp
660 bp
rpl5-515
rpl5-518
[0 - 5151]

Z. Mapping of RNA-seq reads to the coding sequence of *rpl10*.

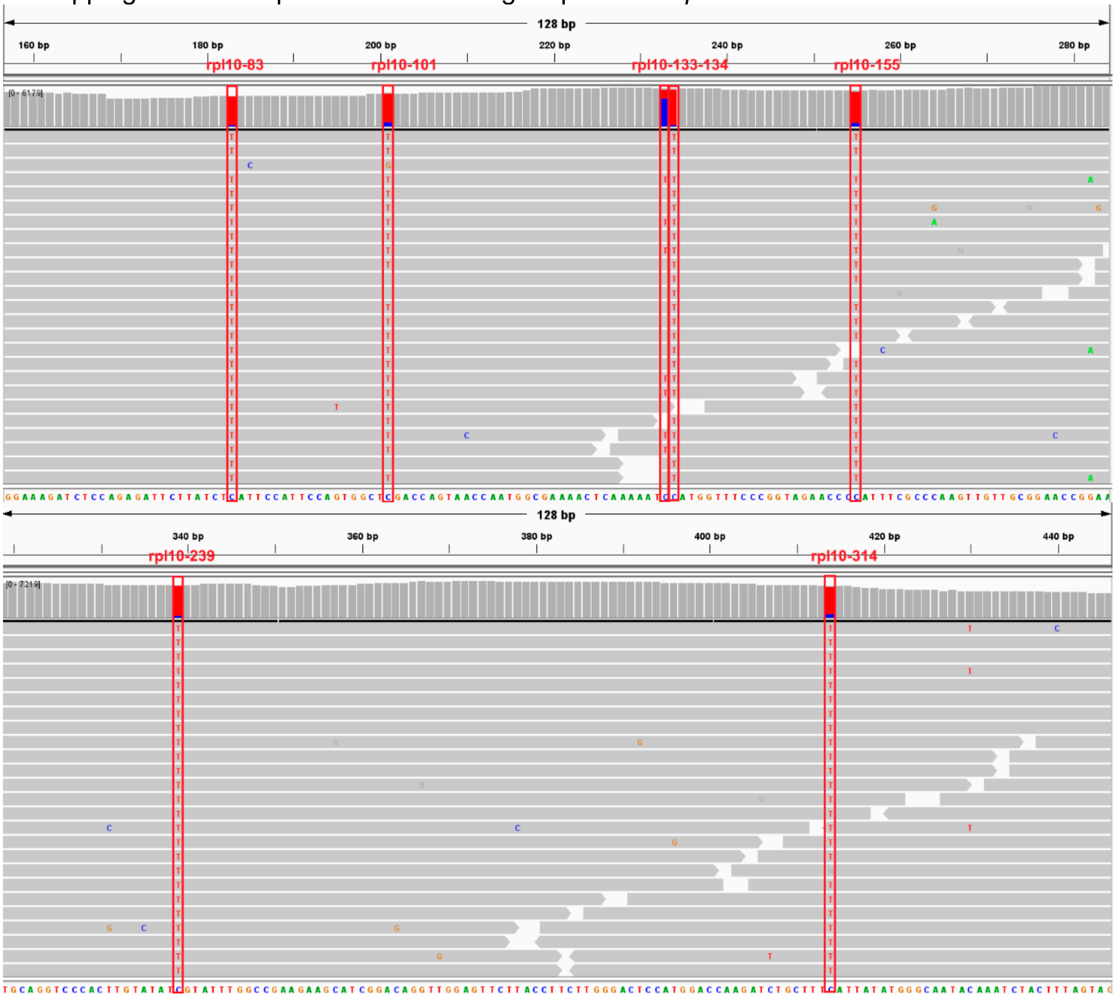

A1. Mapping of RNA-seq reads to the coding sequence of *rpl16.*

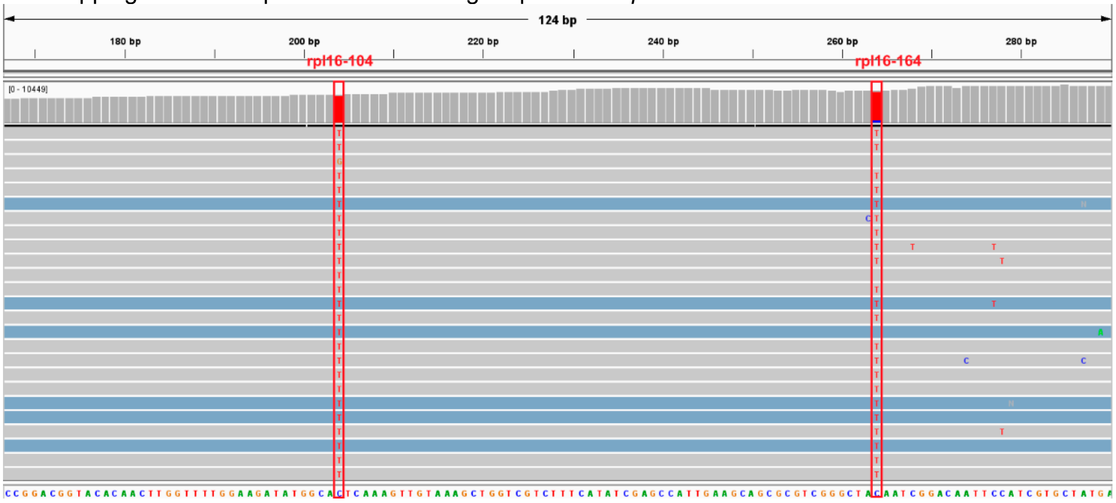

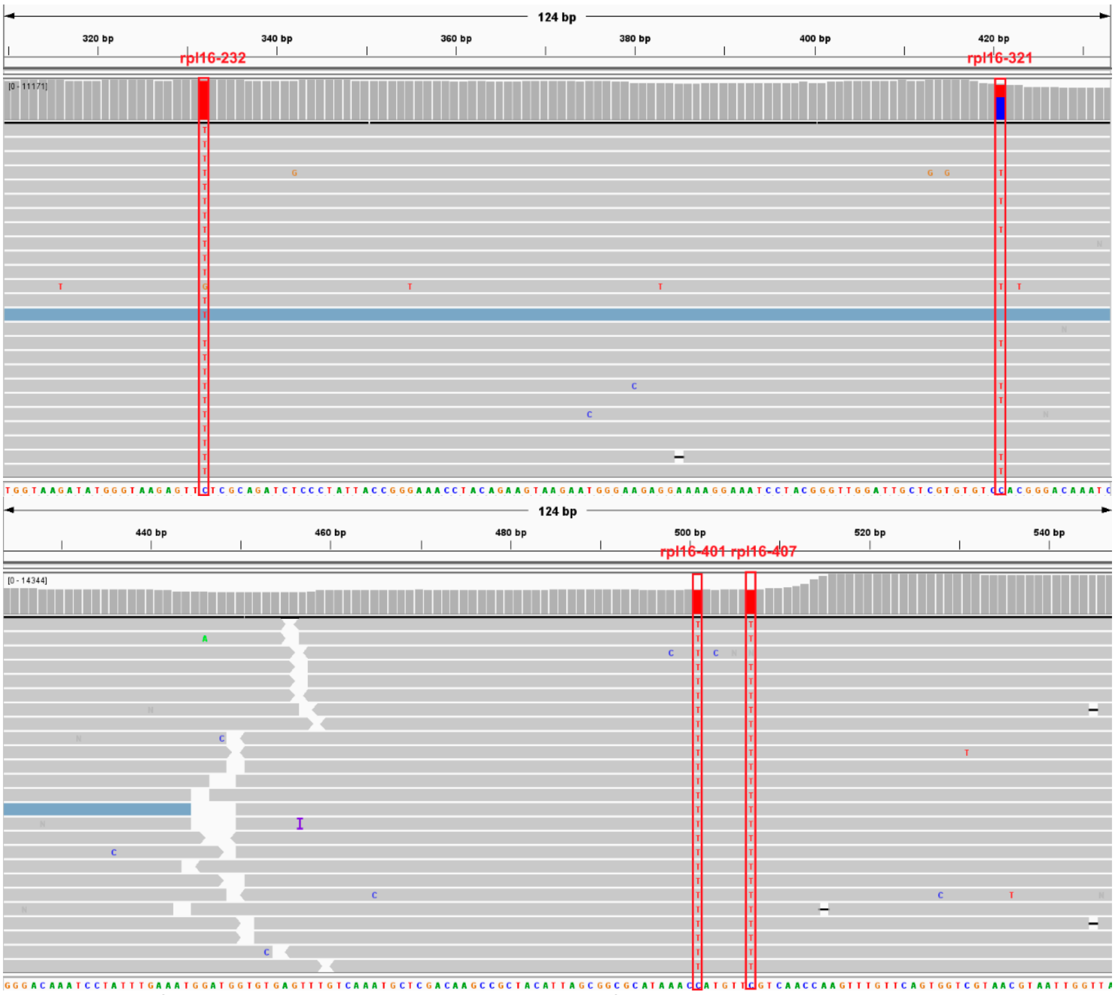

B1. Mapping of RNA-seq reads to the coding sequence of *rps1*.

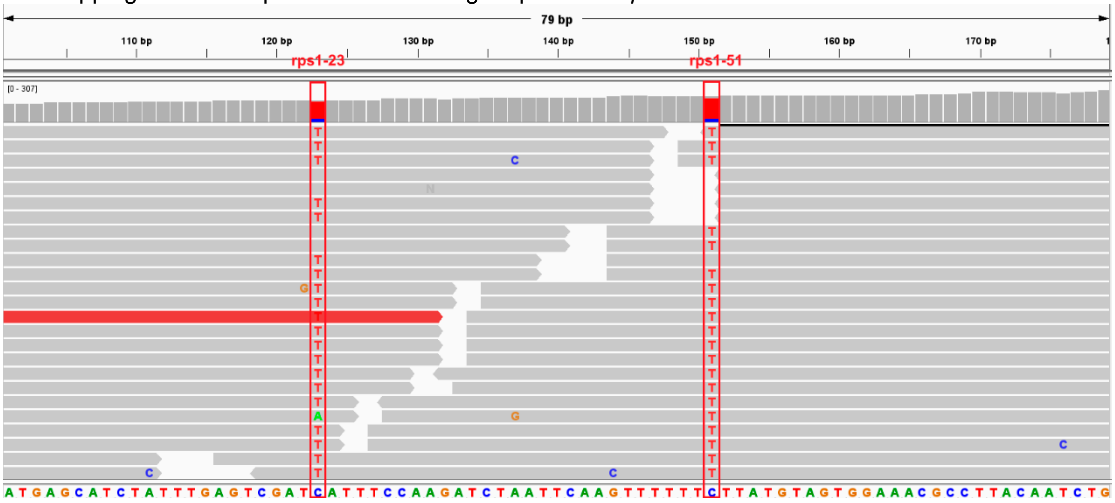

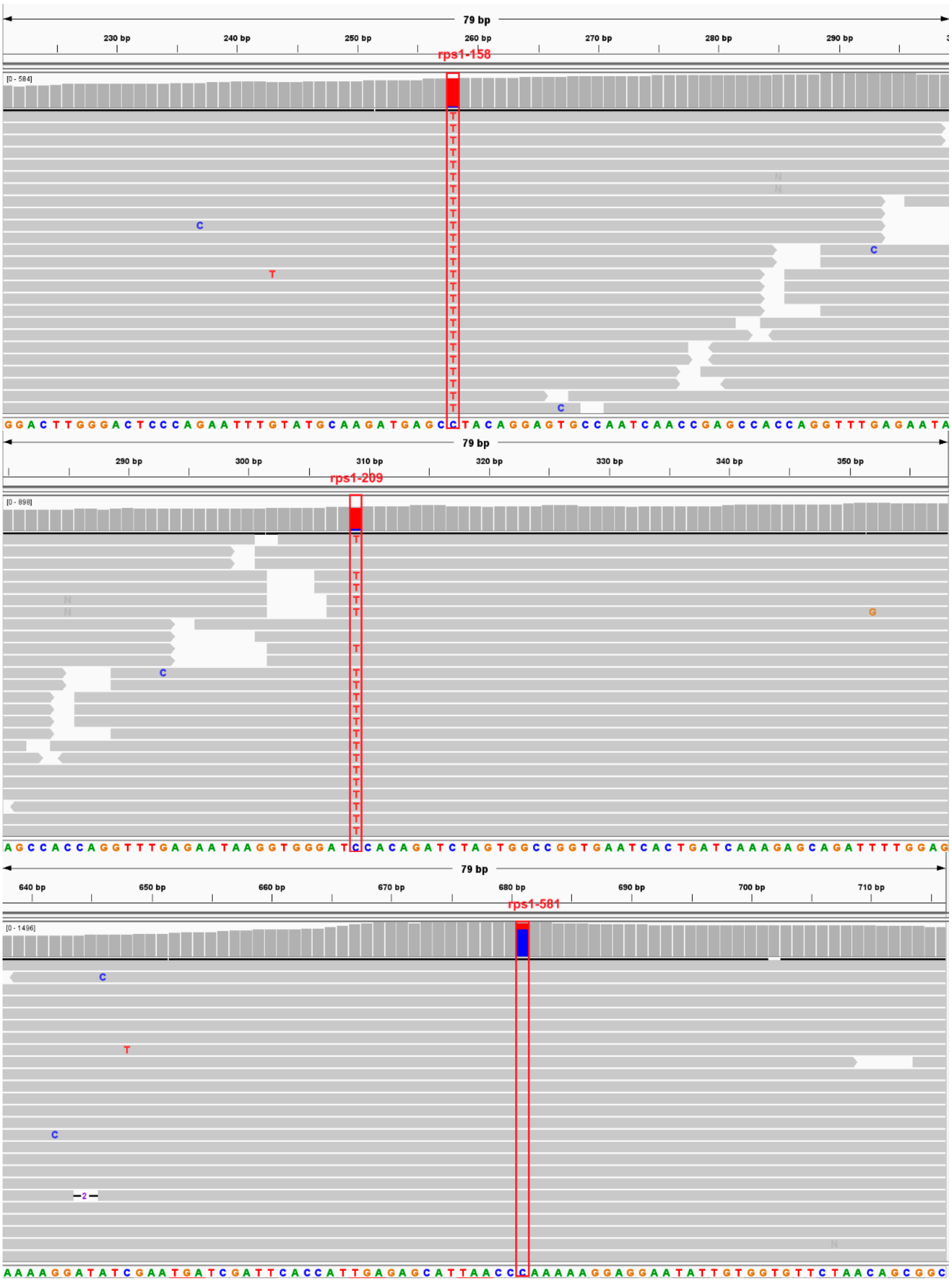
79 bp
230 bp
240 bp
250 bp
260 bp
270 bp
280 bp
290 bp
rps1-158
[0 - 584]
79 bp
290 bp
300 bp
310 bp
320 bp
330 bp
340 bp
350 bp
rps1-209
[0 - 898]
79 bp
640 bp
650 bp
660 bp
670 bp
680 bp
690 bp
700 bp
710 bp
rps1-581
[0 - 1496]

C1. Mapping of RNA-seq reads to the coding sequence of *rps3*.

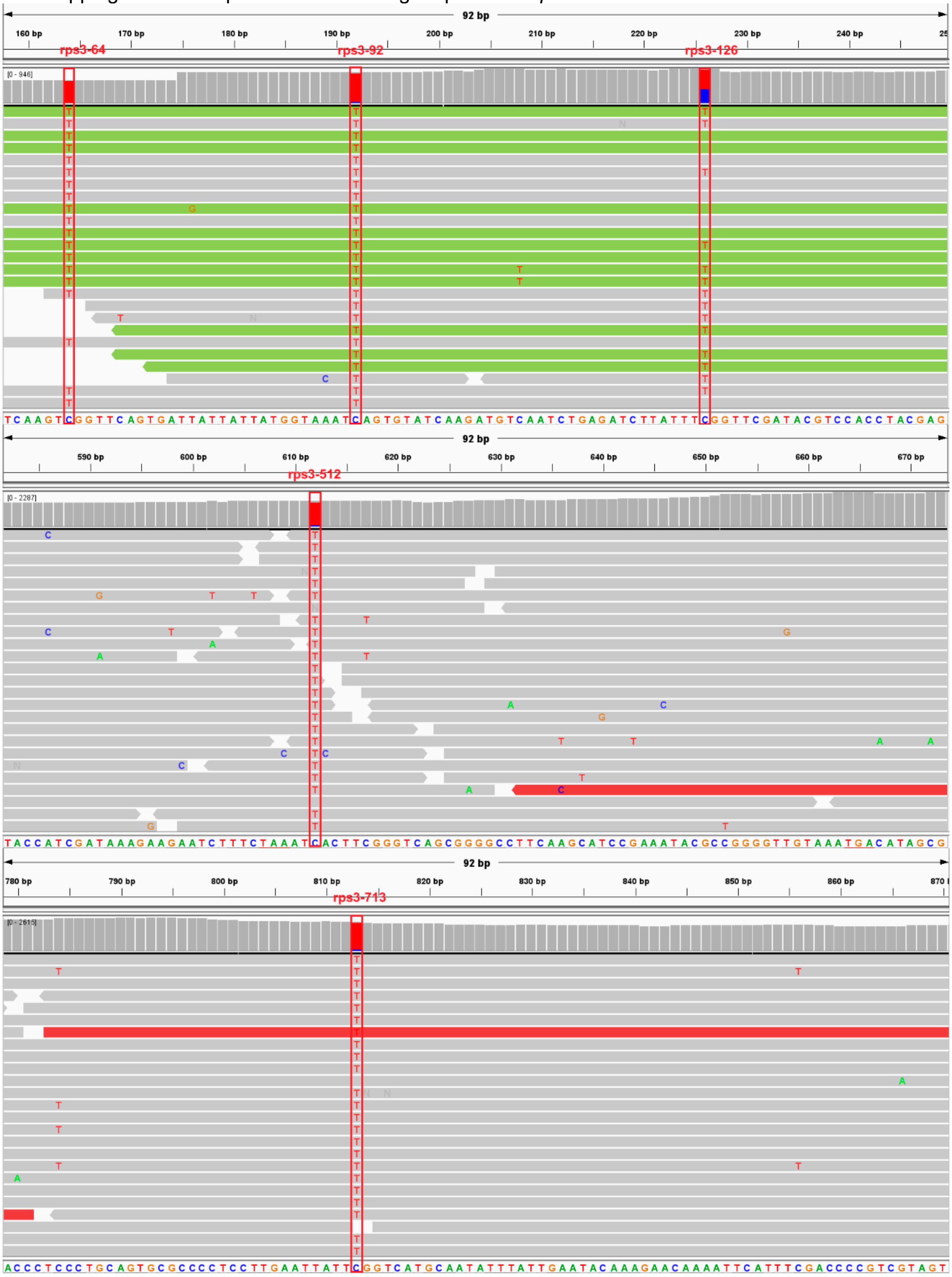

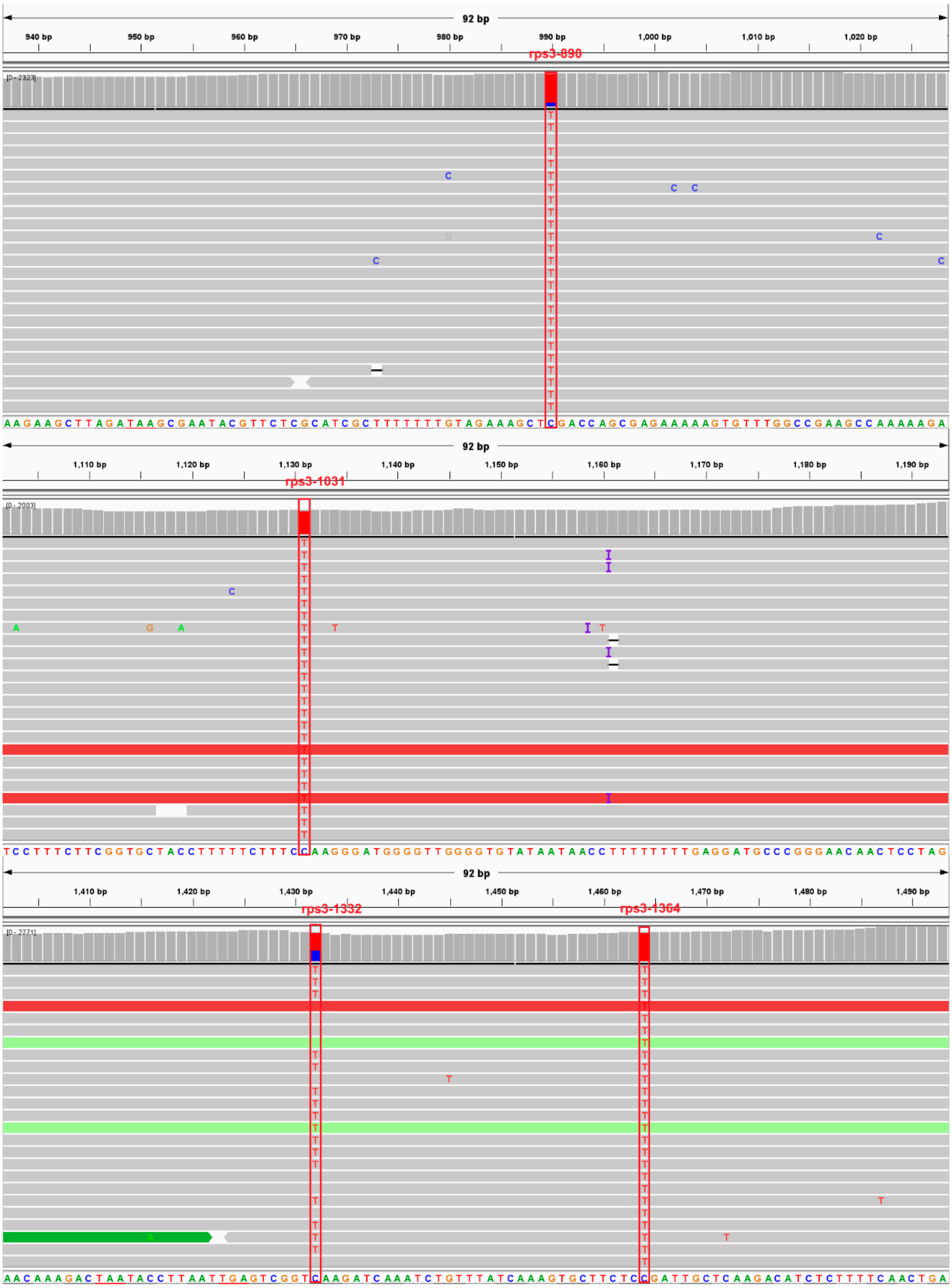
92 bp
940 bp
950 bp
960 bp
970 bp
980 bp
990 bp
1,000 bp
1,010 bp
1,020 bp
rps3-890
[0 - 2323]
92 bp
1,110 bp
1,120 bp
1,130 bp
1,140 bp
1,150 bp
1,160 bp
1,170 bp
1,180 bp
1,190 bp
rps3-1031
[0 - 2003]
92 bp
1,410 bp
1,420 bp
1,430 bp
1,440 bp
1,450 bp
1,460 bp
1,470 bp
1,480 bp
1,490 bp
rps3-1332
rps3-1364
[0 - 2771]

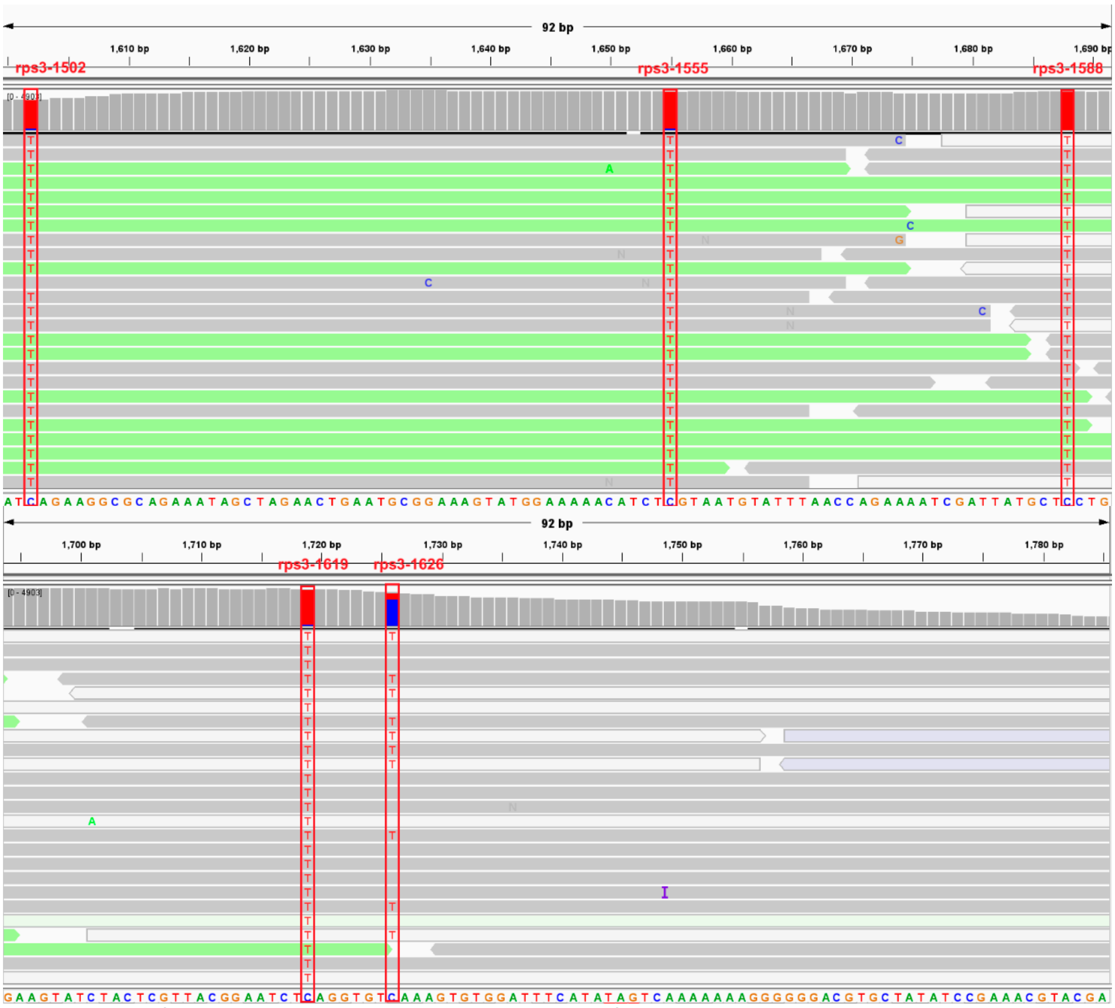

D1. Mapping of RNA-seq reads to the coding sequence of *rps4*.

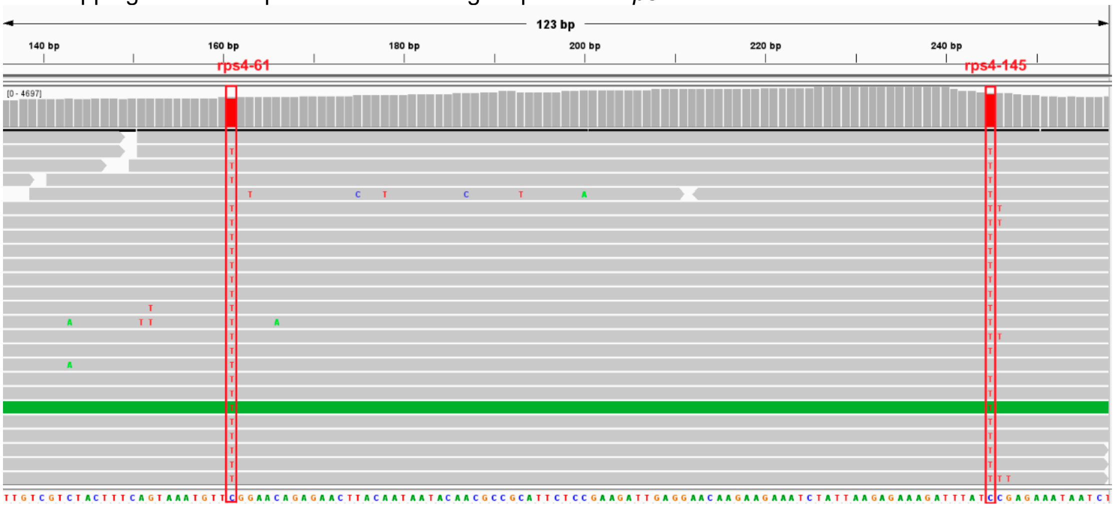

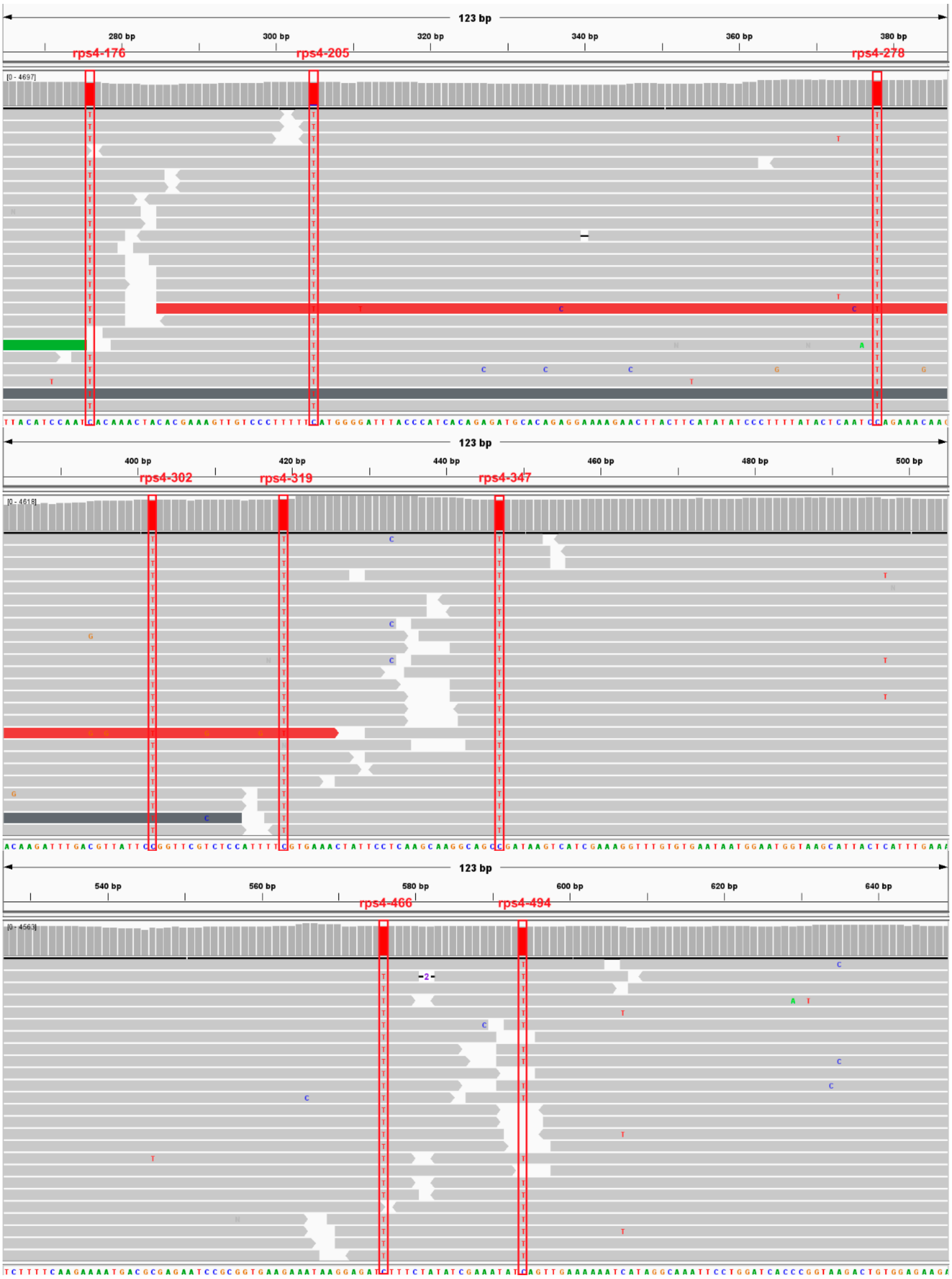
123 bp
280 bp
300 bp
320 bp
340 bp
360 bp
380 bp
rps4-176
rps4-205
rps4-278
123 bp
400 bp
420 bp
440 bp
460 bp
480 bp
500 bp
rps4-302
rps4-319
rps4-347
123 bp
540 bp
560 bp
580 bp
600 bp
620 bp
640 bp
rps4-466
rps4-494

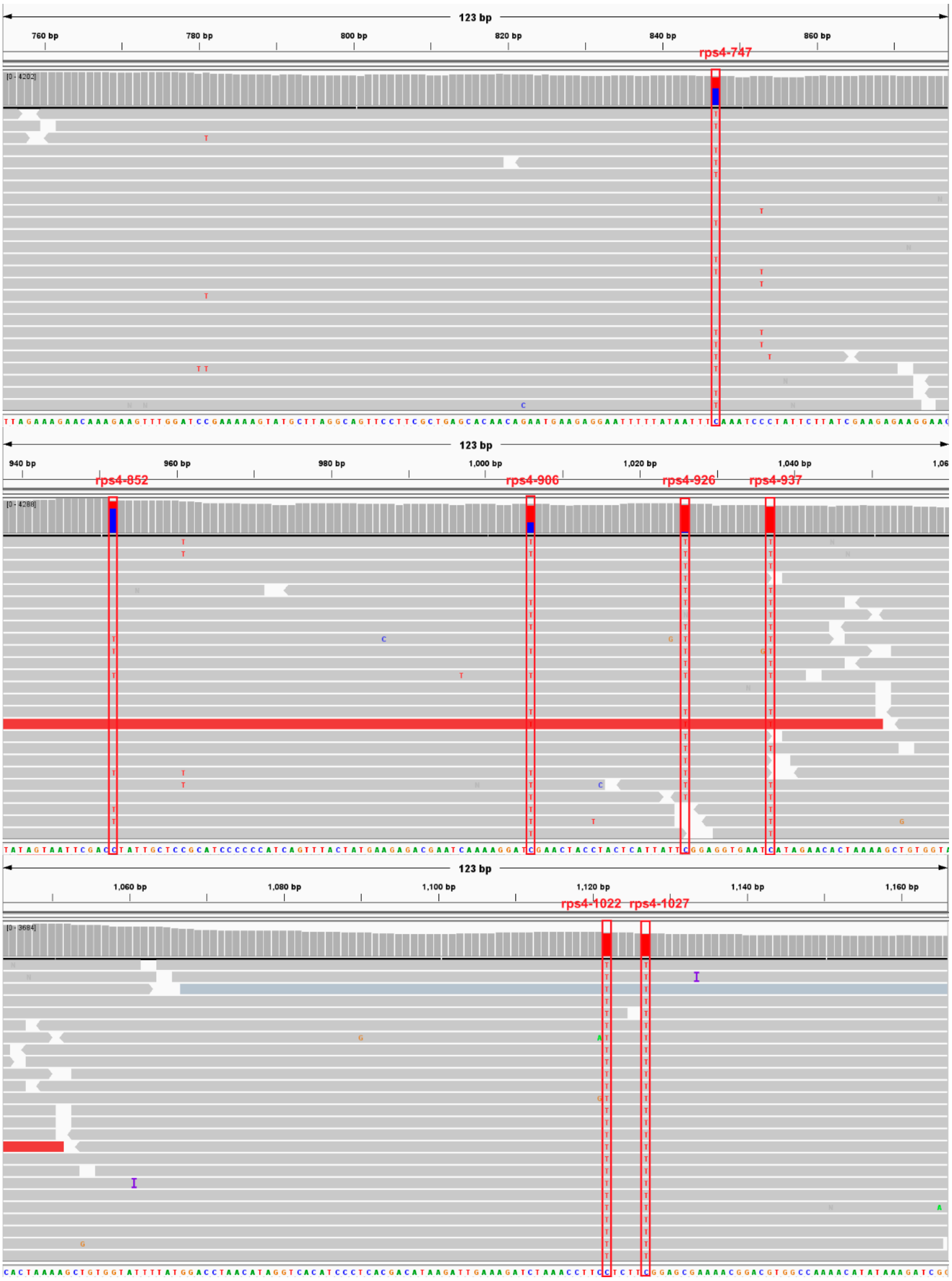
123 bp
760 bp
780 bp
800 bp
820 bp
840 bp
860 bp
rps4-747
[0 - 4202]
123 bp
940 bp
960 bp
980 bp
1,000 bp
1,020 bp
1,040 bp
rps4-852
rps4-906
rps4-926
rps4-937
[0 - 4288]
123 bp
1,060 bp
1,080 bp
1,100 bp
1,120 bp
1,140 bp
1,160 bp
rps4-1022
rps4-1027
[0 - 3684]

E1. Mapping of RNA-seq reads to the coding sequence of *rps10.*

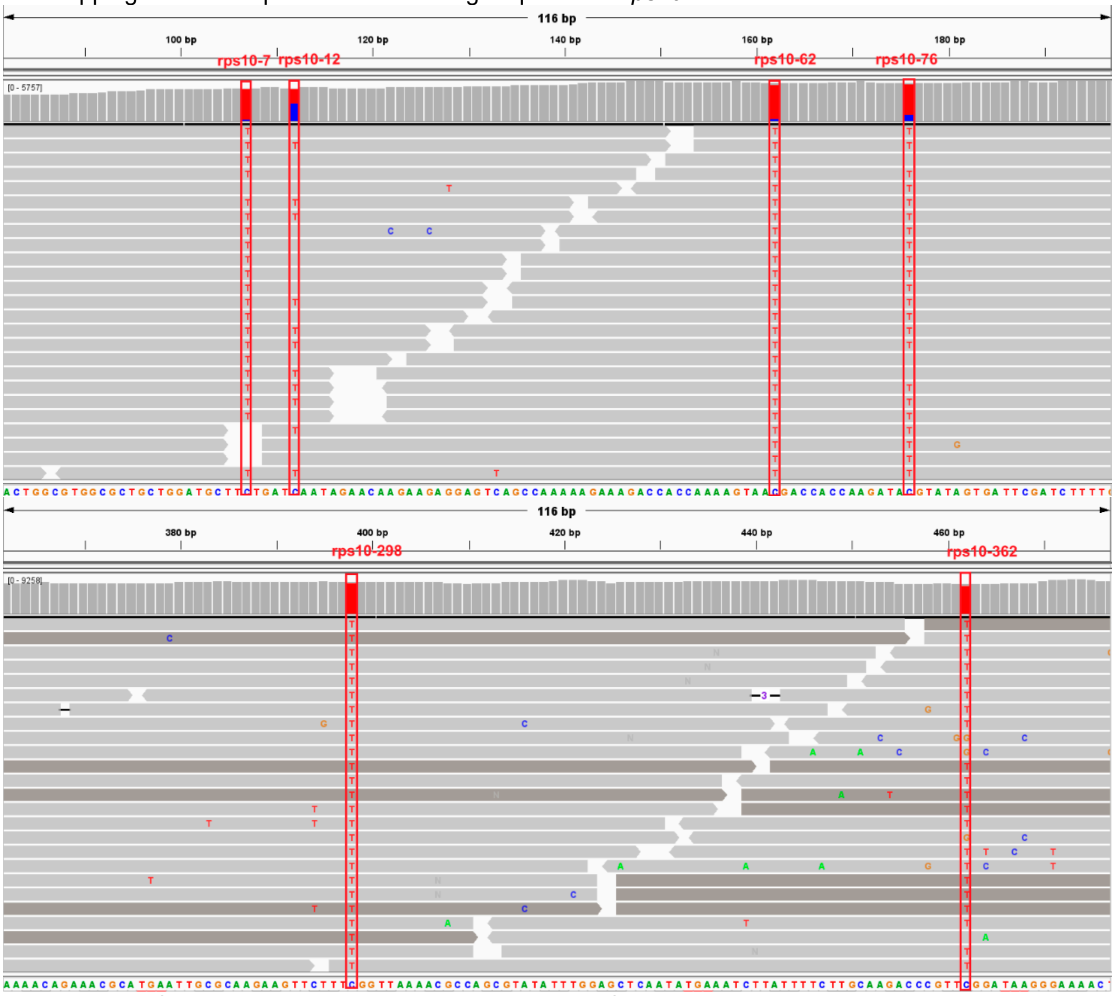

F1. Mapping of RNA-seq reads to the coding sequence of *rps12.*

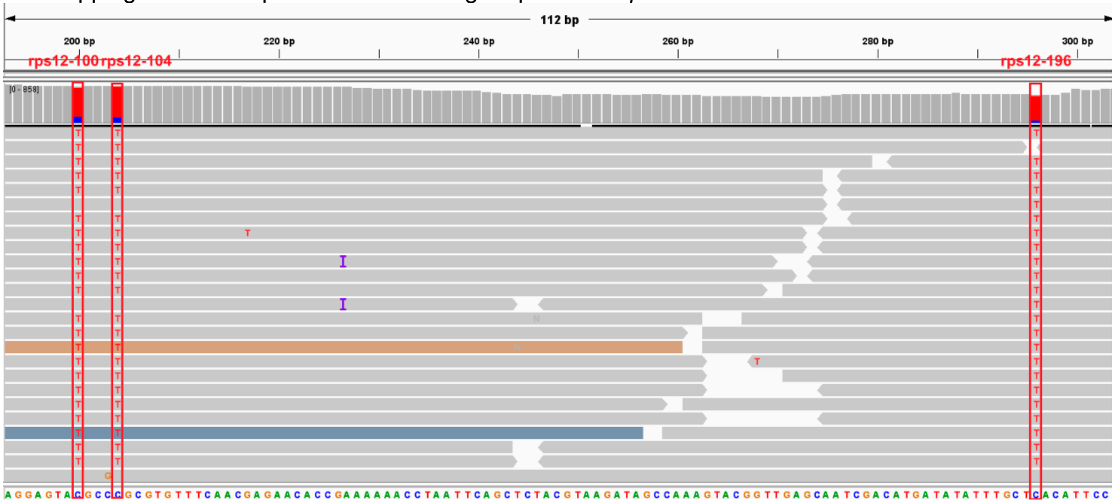

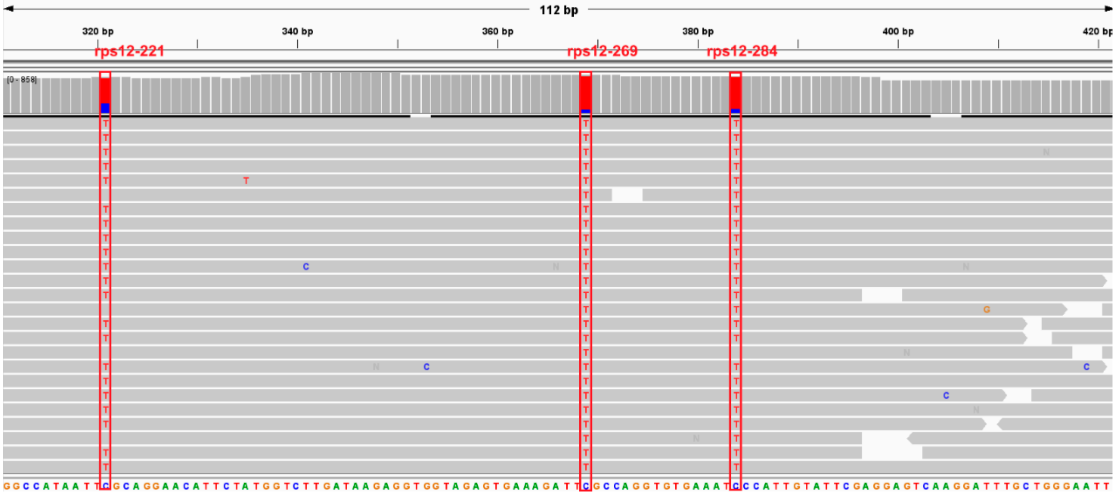
112 bp
320 bp
340 bp
360 bp
380 bp
400 bp
420 bp
rps12-221
rps12-269
rps12-284
[0 - 858]

G1 Mapping of RNA-seq reads to the coding sequence of *rps19.*

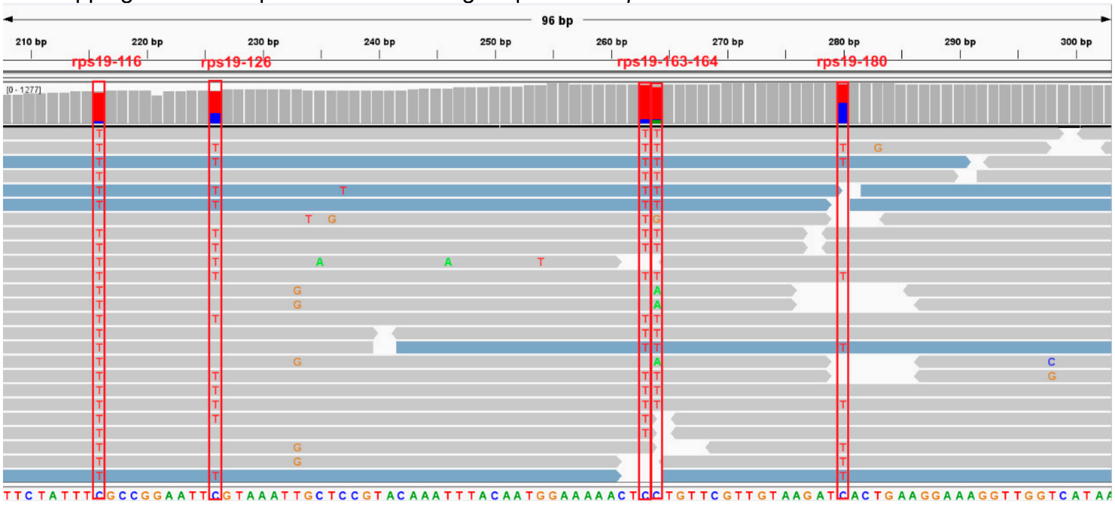

H1 Mapping of RNA-seq reads to the coding sequence of *sdh4.*

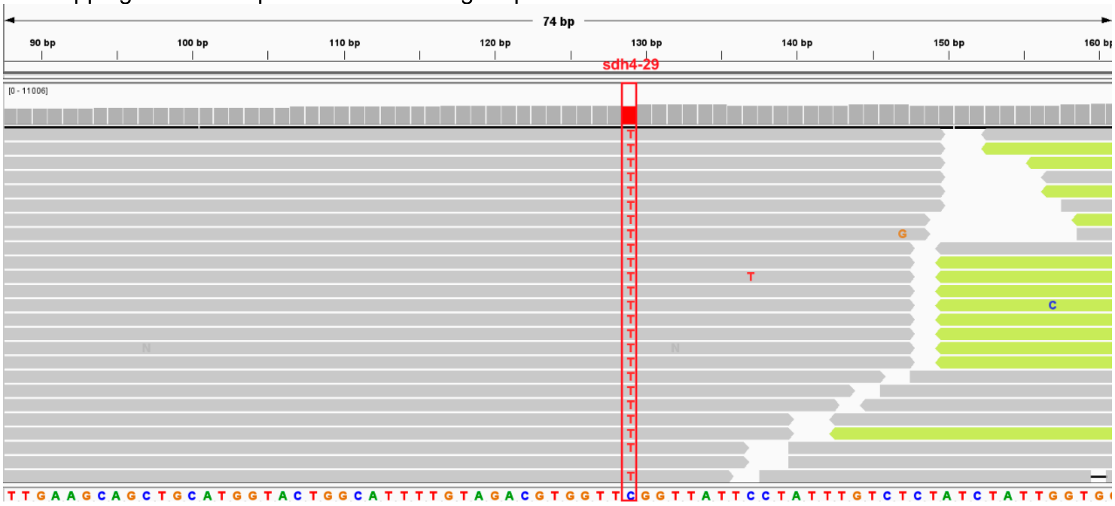

Supplement: Supplementary file 1 [file ijms-26-07093-s001.zip › ijms-3691009-supplementary.pdf]
